# A Set of 17 microRNAs Common for Brain and Cerebrospinal Fluid Differentiates Primary Central Nervous System Lymphoma from Non-Malignant Brain Tumors

**DOI:** 10.3390/biom11091395

**Published:** 2021-09-21

**Authors:** Maria Sromek, Grzegorz Rymkiewicz, Agnieszka Paziewska, Lukasz Michal Szafron, Maria Kulecka, Michalina Zajdel, Mariusz Kulinczak, Michalina Dabrowska, Aneta Balabas, Zbigniew Bystydzienski, Magdalena Chechlinska, Jan Konrad Siwicki

**Affiliations:** 1 Department of Cancer Biology, Maria Sklodowska-Curie National Research Institute of Oncology, 02-781 Warsaw, Poland; Maria.Sromek@pib-nio.pl (M.S.); lukszafron@gmail.com (L.M.S.); Michalina.Zajdel@pib-nio.pl (M.Z.); Mariusz.Kulinczak@pib-nio.pl (M.K.); 2 Flow Cytometry Laboratory, Department of Pathology and Laboratory Diagnostics, Maria Sklodowska-Curie National Research Institute of Oncology, 02-781 Warsaw, Poland; Grzegorz.Rymkiewicz@pib-nio.pl (G.R.); Zbigniew.Bystydzienski@pib-nio.pl (Z.B.); 3 Department of Gastroenterology Hepatology and Clinical Oncology, Centre of Postgraduate Medical Education, 02-781 Warsaw, Poland; apaziewska@cmkp.edu.pl (A.P.); mkulecka@cmkp.edu.pl (M.K.); Michalina.Dabrowska@pib-nio.pl (M.D.); Aneta.Balabas@pib-nio.pl (A.B.); 4 Department of Genetics, Maria Sklodowska-Curie National Research Institute of Oncology, 02-781 Warsaw, Poland; 5 Faculty of Medical and Health Sciences, Institute of Health Sciences, Siedlce University of Natural Sciences and Humanities, 08-110 Siedlce, Poland

**Keywords:** primary central nervous system lymphoma, CNS DLBCL, microRNA, miRNA, miR, next-generation sequencing, cerebrospinal fluid

## Abstract

The diagnosis of primary central nervous system (CNS) lymphoma, which is predominantly of the diffuse large B-cell lymphoma type (CNS DLBCL), is challenging. MicroRNAs (miRs) are gene expression-regulating non-coding RNAs that are potential biomarkers. We aimed to distinguish miR expression patterns differentiating CNS DLBCL and non-malignant CNS diseases with tumor presentation (n-ML). Next generation sequencing-based miR profiling of cerebrospinal fluids (CSFs) and brain tumors was performed. Sample source-specific (CSF vs. brain tumor) miR patterns were revealed. Even so, a set of 17 miRs differentiating CNS DLBCL from n-ML, no matter if assessed in CSF or in a tumor, was identified. Along with the results of pathway analyses, this suggests their pathogenic role in CNS DLBCL. A combination of just four of those miRs (miR-16-5p, miR-21-5p, miR-92a-3p, and miR-423-5p), assessed in CSFs, discriminated CNS DLBCL from n-ML samples with 100% specificity and 67.0% sensitivity. Analyses of paired CSF-tumor samples from patients with CNS DLBCL showed significantly lower CSF levels of miR-26a, and higher CSF levels of miR-15a-5p, miR-15b-5p, miR-19a-3p, miR-106b-3p, miR-221-3p, and miR-423-5p. Noteworthy, the same miRs belonged to the abovementioned set differentiating CNS DLBCL from non-malignant CNS diseases. Our results not only add to the basic knowledge, but also hold significant translational potential.

## 1. Introduction

Primary central nervous system (CNS) lymphoma (PCNSL) is a malignant extranodal form of aggressive B-cell non-Hodgkin lymphoma (B-NHL), predominantly of the histological subtype of primary diffuse large B-cell lymphoma (CNS DLBCL). PCNSL shares some clinical symptoms with a number of non-neoplastic neurological disorders, and in imaging exams, inflammatory and other non-neoplastic lesions often present as tumors resembling PCNSL [1,2,3]. In everyday diagnostic practice, cases with unspecific presentations of brain disorders qualify for time-consuming enhanced diagnostics, while they often require acute treatment (e.g., due to cerebral edema). According to the WHO criteria, the ultimate diagnosis of PCNSL requires histopathological and immunohistochemical (IHC) examination of brain biopsy material [3]. However, the invasive procedure of stereotactic brain biopsy carries a risk of major complications, including intracranial hemorrhage. In some cases, stereotactic brain biopsy cannot be performed due to the inaccessible location of the lesion; in many cases, because of a common initial steroid therapy resulting in vanishing tumors, HP/IHC examination of brain biopsy samples are inconclusive [4,5]. Complementary diagnostic tools, including neuroimaging and cytological and flow cytometry examination of the cerebrospinal fluid (CSF), are also available, but PCNSL diagnosis remains challenging [1]. There is a need to develop new diagnostic methods because fast and reliable diagnosis of PCNSL is a prerequisite for prompt and proper treatment and strongly influences patients’ outcomes. Several CSF markers, including IL-10, CXCL13 [6,7], and neopterin [8], have been proposed for PCNSL diagnosis; however, their diagnostic value has not been confirmed in clinical practice [9]. microRNAs (miRs), small, non-coding RNA molecules that regulate gene expression, have emerged as promising biomarkers, also in lymphoid malignancies and neurologic diseases [10,11]. The utility of CSF miR-21, miR-19b, and miR-92a as PCNSL markers has been suggested [12]. We have recently confirmed [13] that the miR-21, miR-19b, and miR-92a levels were significantly higher in CSFs of patients with CNS DLBCL than of patients with non-malignant brain tumors; however, the diagnostic accuracy of those miRs was found to be lower than previously suggested. We also demonstrated that CNS DLBCL CSFs and the relevant brain biopsy samples are characterized by specific, different miR profiles [13].

Our current study based on next-generation sequencing (NGS) explores the miRNome in brain biopsies and CSF samples, to develop miR signatures that differentiate PCNSL from non-neoplastic CNS diseases.

## 2. Materials and Methods

### 2.1. Patients and Samples

Consecutive CSFs and brain tumor samples analyzed in the study were collected for routine diagnostic purposes from patients with the initial clinical and/or MRI presentation suggesting PCNSL and ultimately diagnosed with CNS DLBCL or non-malignant brain diseases (n-ML). Patients were diagnosed and consulted/treated at the Maria Sklodowska-Curie National Research Institute of Oncology in Warsaw (Table 1 and Table 2).

CSF samples of patients with n-ML were collected in the neurological departments of Warsaw hospitals, for routine flow cytometry (FCM) diagnosis performed at the Flow Cytometry Laboratory, Department of Pathology and Laboratory Diagnostics at the Maria Sklodowska-Curie National Research Institute of Oncology in Warsaw.

CSF samples were obtained via lumbar puncture from patients with the initial clinical and/or MRI presentation suggesting PCNSL, and subsequently diagnosed with CNS DLBCL (n = 9, 6 women/3 men, median age 51, range 31–73) or with n-ML (n = 12, 7 women/5 men, median age 39, range 16–81) (Table 1 and Table 2). The CSF samples were centrifuged at 170× *g*, to recover the cells for the routine cytological and FCM examinations. The leftover supernatants were centrifuged at 500× *g* for 10 min at 20 °C, aliquoted in 400 μL volumes, and stored at −70 °C.

Brain tumor samples were obtained by stereotactic biopsy or surgical resection of the CNS tumors of patients with the initial clinical and/or MRI presentation suggesting PCNSL, and subsequently diagnosed with CNS DLBCL (n = 10, 7 women/3 men, median age 53, range 31–73) or with n-ML (n = 10, 5 women/5 men, median age 40.5, range 28–65) (Table 1 and Table 2).

Formalin-fixed, paraffin-embedded samples were prepared by routine methods. For HP examination, hematoxylin and eosin staining was performed. For IHC, tissue sections were incubated with the diluted antibodies for 1 h in an autostainer (Dako, Carpinteria, CA, USA) following the antigen-retrieval technique, if necessary, using the EnVision™ Detection Systems FLEX kit (Dako Corp., Carpinteria, CA, USA, code K 8000) and monoclonal antibodies (MoAbs) specific for CD20, CD10, BCL6, MUM1, BCL2, CD5, and Ki-67, as previously described [13]. A reaction for CD20, BCL6, MUM1, and CD5 was considered positive if at least 20% of the CNS DLBCL cells showed staining, while for CD10 if any cell showed staining. Cases with a 100% IHC positivity of tumor cells were evaluated as positive (+). Patterns of BCL2 staining were divided into 3 categories: (–) “negative”—lack of BCL2 on CNS DLBCL cells; (+) “positive”—expression of BCL2 on CNS DLBCL cells, comparable/lower than in the surrounding small T lymphocytes; and (++)h—strong BCL2 staining on CNS DLBCL cells, higher than in the background cells. A reaction was considered positive if at least 20% of the CNS DLBCL cells showed the signal of BCL2. 

For the Ki-67 index assessment, 200 cells were counted under HPF (×400), in each case.

Immunophenotyping of CSF samples was performed by FCM. Concentrated cells isolated from CSFs by centrifugation were incubated with a panel of MoAbs (for the staining procedure, see [14], and for a list of MoAbs, see [13]). The expression of B-cell antigens CD(45/19/20/10/HLADR), T-cell antigens CD(45/3/4/8/5/43), and macrophage antigens (CD14/CD4/43/HLADR) was quantified on FACSCalibur and FACSCanto II cytometers (Becton Dickinson, BD, San Jose, CA, USA) and samples were categorized into three groups, according to the percentages of positive cells: (−), no expression (<20% of positive neoplastic cells); (+/−), expression in ≥20%<100% of cells; and (+), expression in 100% of cells. Simultaneously, cytological smears were stained with May–Grünwald–Giemsa for morphological evaluation.

The final diagnosis of CNS DLBCL and n-ML considered histopathological criteria and IHC examination according to the 2016 WHO classification [15,16], and included immunohistochemical subgroups, CD5 positive, germinal-center B-cell (GCB) type, and activated B-cell (ABC) type, distinguished by the Hans algorithm; i.e., based on CD10, BCL6, and multiple myeloma oncogene-1 (MUM1) expression, as proposed in the WHO 2008 classification [17]. CNS DLBCLs were classified as ABC (6 cases), GCB (3 cases), and CD5 positive (1 case). The clinical and patomorphological characteristics of the patients are presented in Table 1. 

The study was conducted in accordance with the Declaration of Helsinki, and the protocol was approved by the Ethics Committee of the Maria Sklodowska-Curie National Research Institute of Oncology (April/2011–January/2012).

### 2.2. RNA Extraction

Total RNA was isolated from CSF and tumor samples according to the protocols described earlier by Zajdel et al. [13]. Briefly, total RNA was isolated from CSF samples with the Gene Matrix Universal RNA/miRNA Purification Kit (EURx, Gdansk, Poland), according to the manufacturer’s instructions. Ten 20-μm-thick sections of each formalin-fixed, paraffin-embedded tissue (FFPET) sample were cut with a disposable blade. Total RNA was extracted using the RecoverAll™ Total Nucleic Acid Isolation Kit (Applied Biosystems, Carlsbad, CA 92008 USA), according to the manufacturer’s recommendations. RNA concentration and quality were measured with the NanoDrop ND 1000 Spectrophotometer (NanoDrop Technologies, Wilmington, DE 19810 USA).

### 2.3. Next-Generation Sequencing

miR libraries were prepared with an Ion Total RNA-Seq Kit v2 and Ion Xpress RNA-Seq BC01-16 Kit (ThermoFisher, Scientific Carlsbad, CA 92008 USA), according to the manufacturer’s protocol, cleaned with Qiagen Gel Extraction Kit (Qiagen, Düsseldorf, Germany), and checked for concentration and quality on Bioanalyzer 2100, using the High Sensitivity DNA Analysis Kit (Agilent, Santa Clara, CA 95051 USA).

The generated amplicons, of equal concentration for each sample, were sequenced on the Ion Proton (Thermo Fisher Scientific, Carlsbad, CA 92008 USA) platform using Ion PI ™ Hi-Q ™ Chef Kit and Ion PI ™ Chip Kit v3.

### 2.4. Quantitative Real-Time PCR

The reverse transcription reaction was performed with the TaqMan Advanced miR cDNA Synthesis Kit (Applied Biosystems CN A28007, Carlsbad, CA 92008 USA). The specific TaqMan Advanced miR Assay (Applied Biosystems CN A25576, Carlsbad, CA 92008 USA) was used to measure miRs specified in Table S1. The internal controls were miR-24-3p for tumor samples and miR-24-3p and miR-23a-3p for CSF samples based on the NormFinder algorithm. 

Quantitative real-time polymerase chain reaction (qPCR) was performed using the TaqMan Fast Advanced Master Mix (Applied Biosystems, Carlsbad, CA 92008 USA) on a 7500 Fast Real-Time PCR System (Applied Biosystems, Carlsbad, CA 92008 USA). All PCR reactions were carried out in triplicates, at a final volume of 10 μL. The data were analyzed with the 7500 Software v.2.0.6 (Applied Biosystems, Carlsbad, CA 92008 USA) and the relative miRs quantities were calculated with the 2^−∆Ct^ method. 

### 2.5. Statistical Analysis

The miRDeep2 application: (https://www.mdc-berlin.de/content/mirdeep2-documentation, accessed on 22 September 2020) was applied to map NGS reads to the human genome hg19, to quantify the known miRs (miRBase V21/22) and to predict novel miRs. Differential miR expression was analyzed with the use of the DESeq2 package; the significance threshold was set at 0.05 after the Benjamini–Hochberg correction (q). The obtained gene expression data were normalized (according to the internal DESeq2 normalization mechanisms) and, after log2-transformation, used for the principal component analysis (PCA) and hierarchical clustering in the R environment, to identify similarities in miR expression patterns between the samples.

The lists of genes with expression levels significantly altered between the studied groups were subsequently used in ontological analyses performed with the miR enrichment analysis and annotation tool (miEAA, https://ccb-compute2.cs.uni-saarland.de/mieaa2, accessed on 1 December 2019). This tool was employed to perform the over-representation analysis of mature miRs in 28 different categories (Table S2). The enrichment analysis was performed with the nonparametric Kolmogorov–Smirnov (KS) test. The minimum hits per sub-category threshold equaled 2. The term was qualified as enriched if the KS test p-value after the Benjamini–Hochberg adjustment was lower than 0.05. The enriched ontological terms were visualized in word clouds, and the statistical significance of each term along with the over-represented miRs are shown as heatmaps. Finally, miRs of potential importance for differentiating CNS DLBCL and benign brain lesions were analyzed by the Pearson’s correlation test to evaluate the relationship between their levels in tumors and the corresponding CSFs in CNS DLBCL patients.

The differences in miR expression levels between samples revealed at the RT-qPCR verification were assessed by the Mann–Whitney U test.

Receiver Operator Characteristic (ROC) analysis was performed to quantify the accuracy of the miR profiling to discriminate between the CNS DLBCL and n-ML samples.

## 3. Results

### 3.1. PCA Analyses

**Figure 1:**
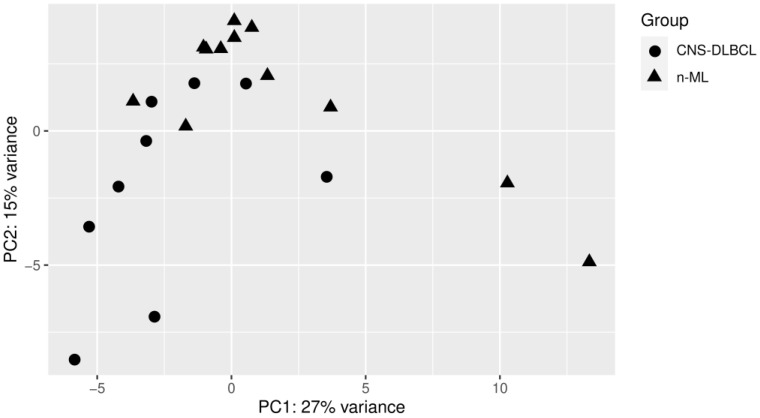
PCA of the cerebrospinal fluid samples from patients with primary diffuse large B-cell lymphoma of the central nervous system (CNS DLBCL, n = 9) and non-malignant diseases (n-ML, n = 12).

**Figure 2:**
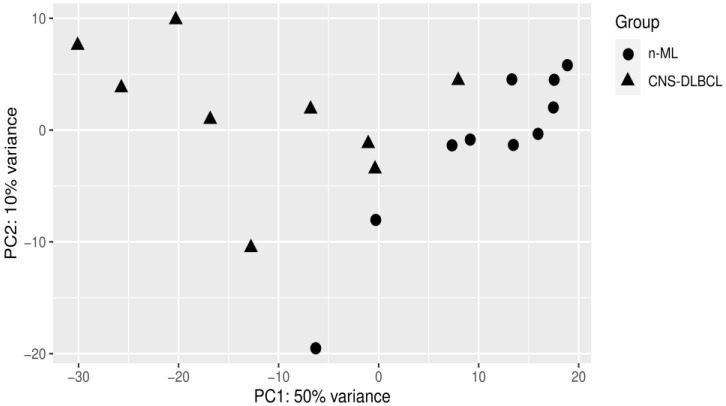
PCA of the tumor samples from patients with primary diffuse large B-cell lymphoma of the central nervous system (CNS DLBCL, n = 10) and with non-malignant CNS diseases (n-ML, n = 10).

PCA analysis showed that the miR levels clustered samples according to their malignant vs. non-malignant origin from patients with non-malignant vs. malignant CNS tumors. The segregation was much more evident for the n-ML and CNS DLBCL tumor samples than for the CSF samples (Figure 1 and Figure 2).

### 3.2. CNS DLBCL-Specific CSF miR Profile

The NGS analysis of all 21 CSF samples identified a total number of 406 miRs of 2588 miR sequences recorded in the miRBase v21. Eighteen CSF miRs were significantly differentially expressed between the two groups of patients, including 15 (miR-15a-5p, miR-15b-5p, miR-16-5p, miR-17-3p, miR-19a-3p, miR-19b-3p, miR-20a-5p, miR-21-5p, miR-25-3p, miR-92a-3p, miR-106b-3p, miR-148a-3p, miR-210-3p, miR-221-3p, and miR-423-5p) miRs with higher and 3 miRs (miR-9-3p, miR-9-5p, and miR-26a-5p) with lower expression levels in CNS DLBCL than in non-neoplastic CNS diseases (Table S3).

### 3.3. CNS DLBCL-Specific miR Profile of Brain Tumors

A total of 349 miRs out of 2588 miR sequences recorded in the miRBase v21 were detected in 20 FFPET samples of CNS tumors, 10 from CNS DLBCL, and 10 from n-ML. Significantly different expression of 205 miRs was shown between patients with CNS DLBCL and with n-ML, including 87 with a higher expression and 118 with a lower expression in CNS DLBCL (Table 3 and Table S3).

### 3.4. A Common set of CSF and Tumor miRs Differentially Expressed between CNS DLBCL and Non-Malignant CNS Diseases

**Figure 3:**
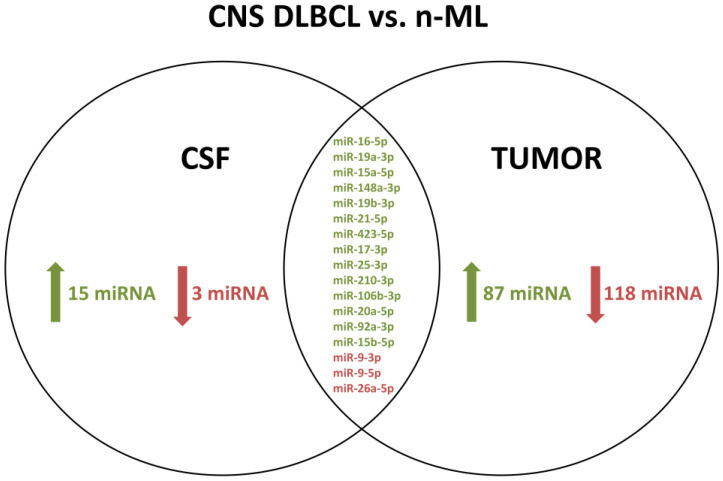
A common set of cerebrospinal fluid (CSF) and tumor microRNAs differentially expressed between patients with primary diffuse large B-cell lymphoma of the central nervous system (CNS DLBCL) and non-malignant CNS diseases (n-ML). Red and green mark miRNAs down- or upregulated in CNS DLBCL vs n-ML.

Seventeen miRs differentially expressed between patients with CNS DLBCL and those with n-ML, were common for both CSF and tumor samples. Those included 14 miRs (miR-15a-5p, miR-15b-5p, miR-16-5p, miR-17-3p, miR-19a-3p, miR-19b-3p, miR-20a-5p, miR-21-5p, miR-25-3p, miR-92a-3p, miR-106b-3p, miR-148a-3p, miR-210-3p, and miR-423-5p) with a higher expression in malignant vs. n-ML samples, and 3 miRs (miR-9-3p, miR-9-5p, and miR-26a-5p) with a lower expression (Table 3, Figure 3). Noteworthy, only one miR of the CNS DLBCL-specific CSF profile, namely, miR-221-3p, does not belong to the common set of 17 miRs.

### 3.5. miR Profiles in Paired CSF/Brain Samples from Patients with CNS DLBCL

The analysis of nine paired CSF and brain tumor samples from patients with CNS DLBCL revealed 624 miRs to be expressed at a significantly lower level, and 79 miRs at a significantly higher level in CSF than in brain tumor samples. Noteworthy, among those expressed at a lower level in the CSFs, there was miR-26a-5p, and those expressed at a higher level in CSFs included 5 miRs, miR-15a-5p, -15b-5p, -19a-3p, -106b-3p, and -423-5p, which significantly differentiated CNS DLBCL vs. non-malignant CNS disease, no matter if assessed in CSF or in a tumor.

### 3.6. New, Previously Unannotated micoRNA Molecules

NGS analysis revealed five new miRs not yet included in the miRBase v22. However, none of them significantly differentiated malignant and benign lesions, four were detected in all or in the majority of tumor biopsies, while their occurrence in CSFs was much less frequent (Table S4).

### 3.7. RT-qPCR Validation of the NGS Results

In order to verify the NGS data, we focused on the set of 17 miRs differentially expressed between patients with CNS DLBCL and with non-malignant CNS diseases in both CSF and tumor samples. miR expression was verified by RT-qPCR in all tumor brain tumor samples and CSFs from patients with CNS DLBCL and with non-malignant CNS diseases included in the NGS analysis. The expression levels of all except two miRs (miR-9-3p and miR-26a-5p) were confirmed to significantly differentiate CNS DLBCL and non-malignant brain tumor samples. In CSFs, the levels of five miRs were confirmed to differentiate CNS DLBCL from n-ML (miR-16-5p, miR-92a-3p, miR-106b-3p, miR-423-5p, and miR-21-5p that nearly reached the significance threshold) (Table 3). It needs to be emphasized that RT-qPCR validation of the NGS results is commonly qualified as challenging, as the RT-qPCR technique, although routinely used, is regarded as not sufficient to validate NGS results. Significant discrepancies between NGS and RT-qPCR results have been observed in other studies involving cerebrospinal fluids [18,19]. Considering the above, the validation we achieved should be regarded as more than satisfactory. 

### 3.8. ROC Analyses

**Figure 4:**
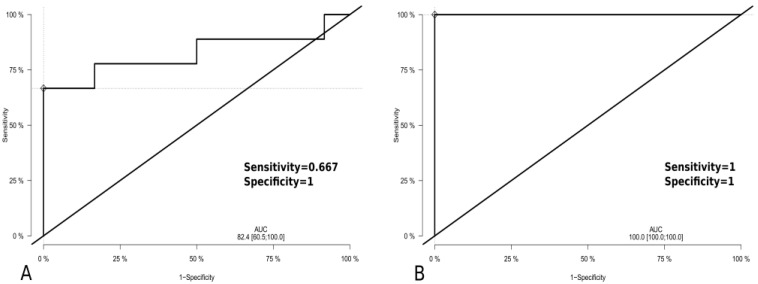
ROC analysis of the performance of a combined miR-16-5p, miR-21-5p, miR-92a-3p and miR-423-5p expression to discriminate primary diffuse large B-cell lymphoma of the central nervous system (CNS DLBCL) and non-malignant CNS lesions (n-ML) in (**A**) CSFs (CNS DLBCL, n = 9; n-ML, n = 12) and (**B**) tumor samples (CNS DLBCL, n = 10; non-ML, n = 10).

As shown by ROC analyses each of the 17 miRs differentially expressed between patients with CNS DLBCL and with non-malignant CNS diseases in both CSF and tumor samples showed a high discrimination power when assessed in tumor samples AUC > 80), while when assessed in CSFs, 10 of them reached AUC > 70 (Table 3). Combining miR-16-5p, miR-21-5p, miR-92a-3p, and miR-423-5p (selected out of those that were positively verified by the RT-qPCR) in the ROC analysis resulted in a high power of discrimination between CNS DLBCL and n-ML samples, 100% specificity, 100% sensitivity, and AUC = 100 for tumor samples; and 100% specificity, 67.0% sensitivity, and AUC = 82.4 for CSF samples (Figure 4).

### 3.9. Functional Analyses of CNS DLBCL-Specific CSF and Tumor miR Profiles

We performed comprehensive ontological analyses of the CNS DLBCL-specific miR profiles of CSFs and tumor samples using the miR enrichment analysis and an annotation tool (miEAA, https://ccb-compute2.cs.uni-saarland.de/mieaa2, accessed on 1 December 2019).

#### 3.9.1. Bioinformatic Analysis of CNS DLBCL-Specific CSF miR Profile

Ontological analysis of the CNS DLBCL-specific CSF miR profile (18 miRs) revealed the biological processes that were regulated by this set of miRs. *Positive regulation of cellular metabolic process*, *organelle organization* and *positive regulation of cellular biosynthetic process* were the top biological processes. Of all processes revealed, 11 were related to cellular metabolism (Database: miRPathDB GO Biological process) (Table S5).

The set of CNS DLBCL-specific CSF miRs was linked, among others, to nervous system diseases, glioblastoma, brain injuries, lymphomas of different cell of origin, and lymphoproliferative disorders (Database: Diseases MNDR) (Table S6).

Next, with the use of the SM2miR database of small molecules’ effects on miR expression [20], we identified a number of small molecules/drugs that potentially can significantly influence the expression level of the set of 18 CSF-derived CNS DLBCL-specific miRs. The identified small molecules/drugs included various epigenetic drugs: histone deacetylase inhibitors (HDACi), e.g., *LAQ824 (dacinostat)* and *ITF2357 (givinostat)*; DNA methylation inhibitors, such as *Decitabine*, *Azacitidine*, and *Temozolomide*; and the bioactive compounds *curcumin*, *marine fungal metabolite 1386A, 3,3-diindolylmethane BR-DIM*, and *ginsenoside Rh2* (Table S7).

Thereafter, using the miRandola database, which classifies extracellular non-coding RNAs according to their form and source, the set of CNS DLBCL-specific CSF miRs were found to be predominantly *microparticle-associated*, *binding with Argonaute 2 protein* and *microvesicle-derived*. According to the miRPathDB GO Cellular component database, the CSF-derived CNS DLBCL-specific miRs were localized mainly in *vesicles*, *cytoplasm*, *nuclear body*, and *protein-containing complex.* Another database, miRWalk Organs, identified *lymphocytes*, *endothelial cells*, and *brain*, among others, to be the sources of these miRs (Table S8). 

We also showed (Table S9) that the analyzed set of CSF miRs may directly influence many important cellular processes, *miR mediated inhibition of translation* and *negative regulation of cell population proliferation* (GO Annotations database) in particular, and may also act indirectly, by influencing the function of key genes involved in signaling pathways or important cellular processes, such as *neuron apoptotic process* and *anaphase-promoting complex binding* (GO Annotation indirect database).

In order to determine whether the CNS DLBCL-specific miRs are linked to particular signaling pathways, we used the miRPathDB Reactome and miRPathDB KEGG databases. The miRPathDB Reactome database revealed several significantly enriched pathways, e.g., *PTEN regulation, PIP3-activated AKT signaling, transcriptional regulation by TP53, signaling by TGF-beta family members, pre-NOTCH expression and processing, estrogen-dependent gene expression, ESR-mediated signaling*, and *regulation of RUNX1 expression and activity* (Table S10). The analysis of the CSF miR set based on the miRPathDB KEGG database identified significant participation of these miRs in *pathways in cancer*, *p53 signaling pathway*, *cell cycle*, and in the *FoxO-, neurotrophin-, TNF-, Wnt-*, and *ErbB-signaling pathways,* among others (Table S10). 

By employing the miRTarBase database designated to define miR–target interactions, we identified 1688 target genes related to the analyzed group of CNS DLBCL-specific CSF miRs and largely linked to important cancer signaling pathways. There was a significant abundance of miRs related to genes responsible not only for basic signaling pathways and cellular processes, often disturbed in carcinogenesis (*TFB1M, NFKB1, GIT2, PTEN,* and *RASA1*), but also to genes involved in ion transport (*SLC9A6* and *ITPR1*), cell communication (*DICER1, DENND6A,* and *ARCN1*), and adhesion (*DENND6A, RASSF5,* and *BTBD7*). Other identified genes are directly related to the functioning of B lymphocytes (*NFKB1, POU2AF1,* and *RASSF5*) or the pathogenesis of DLBCL (*NFKB1, MAP2K3 CHEK1, DDX3X,* and *NOTCH2*) (Table S11). This set of genes also included some of the abovementioned genes, e.g., *PTEN* and *NOTCH.*

#### 3.9.2. Bioinformatic Analysis of CNS DLBCL-Specific Tumor miR Profile

Ontological analysis (miRPathDB GO Biological process database) of 205 brain tumor miRs, with significantly different expression between CNS DLBCL and non-malignant CNS diseases, showed that these miRs are involved in many cellular processes, with *cellular protein modification process, immune system process, positive regulation of metabolic process, response to organic substance and growth factor, negative regulation of cell death*, and *intracellular signal transduction* on top of the list (Table S12).

Next, we used the Mammalian ncRNA-Disease Repository database (MNDR), designated to extract the miR–disease associations, and found that the CNS DLBCL-specific tumor miR profile is linked to many cancers, including *lymphoma, B-cell lymphoma*, *Burkitt lymphoma*, as well as to diseases of the CNS, including *neurodegenerative disease*, *amyotrophic lateral sclerosis*, and *brain disease* (Table S13).

SM2miR database-based analysis revealed that the expression level of brain tumor-derived CNS DLBCL-specific miRs might be significantly modified by several epigenetic drugs, including DNA methylation inhibitors, e.g., *Decitabine*, *Azacitidine*, and *Temozolomide*, as well as histone deacetylase inhibitors (HDACi), e.g., *LAQ824 (Dacinostat)*, *ITF2357 (Givinostat)*, *Vorinostat (SAHA)*, and *Trichostatin A (TSA)*. Small molecules with a potentially significant influence on these miRs’ expression levels include arsenic trioxide and the following bioactive compounds: *Aidi injection (extracts from Radix Ginseng, Astragaloside, Eleutherococcus senticosus*, and *Cantharidin*), bioactive compound from *Panax ginseng (ginsenoside Rh2)*, *marine fungal metabolite 1386A*, and *curcumin* (Table S7).

According to the Tissue Atlas database, the analyzed CNS DLBCL-specific miRs were classified as expressed primarily in *brain, spinal cord*, and *dura mater*. The miRandola database classified the analyzed miRs mainly as *circulating* (Table S14). 

Next, we found that the analyzed 205 miR profile is linked to various molecular functions, such as *negative regulation of anoikis, neuron apoptotic process, cerebral cortex development*, and *epithelial to mesenchymal transition* (GO Annotations indirect database). We also found that those 205 CNS DLBCL-specific miRs participate in the regulation of a number of signaling pathways, including *FoxO, HIF-1*, *PI3K-Akt*, *TGF-beta*, *mTOR*, *ErbB*, and *TP53* (miRPathDB KEGG database) (Table S15).

Analyses employing the miRPathDB Reactome database confirmed that the CNS DLBCL-specific miR set is significantly linked to the following pathways: *PIP3 activated AKT signaling*, *TGF-beta receptor complex*, and *transcriptional regulation by TP53 pathways*, as well as *PTEN regulation*, *interleukin-4* and *interleukin-13 signaling*, *signaling by nuclear receptors*, and *TP53 regulates metabolic genes* (Table S15).

The analysis of miR-target interactions with the use of the miRTarBase database revealed a set of 3023 genes that most strongly interact with the identified group of CNS DLBCL-specific miRs (Table S11). These genes are responsible for many important cellular processes (e.g., proliferation and apoptosis) and are associated with the pathogenesis of DLBCL, *BCL2, RUNX2, NOTCH2, MYC, APC, BMPR2, PTEN, IL6, CAMTA1*, and *PRKAR1A* among them. The identified set of genes also includes *ARCN1, BTBD7*, and *PTEN*, strongly related to the CNS DLBCL-specific CSF miRs (Table S11).

## 4. Discussion

We present the first NGS-based study examining CSF and brain biopsy miRNomes, in order to identify patterns differentiating patients with CNS DLBCL from those with non-malignant CNS diseases.

In line with our previous RTq-PCR-based study that focused on seven miRs [13], we revealed CNS DLBCL-specific miR profiles that are different for CSF and brain biopsy samples. Still, we identified here a set of 17 miRs that differentiates CNS DLBCL from non-malignant CNS tumors, no matter if assessed in CSF or brain biopsy samples, which implies their biomarker potential. Combined levels of just four of those miRs presented a high power of discrimination between the CNS DLBCL and n-ML samples. 

Molecular discordance between CSF and brain samples has been reported in other diseases. In sporadic Creutzfeldt–Jakob disease, there was no correlation between altered miR profiles in CSF and pathologically affected brain regions, while in Alzheimer’s disease a limited correlation has been shown [21,22,23]. Even the established Alzheimer disease marker, amyloid-β 42, presented an increased brain deposition and decreased CSF and plasma levels [24]. In cancer patients, the profiles of circulating miRs have been shown not necessarily to reflect their expression in tumor samples [25,26,27,28,29,30,31]. The lack of correlation between the disease-specific miR levels in body fluids and the matched tumor biopsies is not clearly understood, and may be associated with the systemic effects of cancer progression [32]. A multi-organ origin of most plasma-circulating miRs has also been suggested [33]. CSF miRs may also derive from different cell types, such as those associated with the ventricular choroid plexus, ventricular system, the subarachnoid space, and spinal cord, while miRs detected in tumor biopsy specimens may be regarded as originating mainly from cancer cells [34]. Disease-related CSF miR profiles may also be biased by miRs that, encapsulated in exosomes, may cross the blood–brain barrier (BBB) [35] or brain-derived miRs that reach the circulation due to a BBB dysfunction which frequently accompanies CNS cancers and neurodegenerative diseases [36]. In addition, it has been demonstrated that miRs can be selectively secreted by or retained in normal or malignant cells [37,38,39,40]. As shown in a recent study in healthy donors, brain tissues and CSF exosomes differ in miR profiles, suggesting a selective secretion of miRs by brain tissues [39]. 

Our NGS analysis of paired CSF and brain biopsy samples from CNS DLBCL patients demonstrated significantly lower CSF miR-26a levels, and significantly higher CSF levels of miR-15a-5p, miR-15b-5p, miR-19a-3p, miR-106b-3p, miR-221-3p, and miR-423-5p. Noteworthy, we also found that the levels of exactly the same miRs differentiated CNS DLBCL from non-malignant CNS diseases, no matter if assessed in CSF or in a tumor. These CNS DLBCL-specific changes in miR content in CSFs and brain biopsies may suggest their role in the pathogenesis of brain diseases.

The tumor suppressor activity of miR-26a and its downregulation has been documented in many malignancies, including lung [41], breast [42], nasopharyngeal [43], gastric cancers [44], prostate cancers [45], melanoma [46], and Burkitt lymphoma [47]. Noteworthy, a widespread MYC-induced repression of miRs (miR-26a included) contributes to the pathogenesis of *MYC*-driven aggressive B-NHLs [48], and *MYC* expression has been demonstrated in 70–90% of CNS DLBCL cases [49,50,51]. The CNS DLBCL-specific miR-26a downregulation that we present here is also in line with the previous studies suggesting an *MYC*-miR-26a-*EZH2* positive feedback loop in aggressive B-NHLs [52].

Our data suggest an oncogenic role of miR-15a-5p, miR-19a-3p, miR-106b-3p, and miR-423-5p. Significantly increased levels of serum miR-15a-5p have been shown in DLBCL patients [53,54], and a link between an increased miR-15a-5p expression and neuroblastoma progression has been suggested based on studies involving clinical samples and cell lines [55,56]. Increased miR-19a-3p expression and its oncogenic role has been demonstrated in multiple myeloma [57], hepatocellular carcinoma [58], ovarian cancer [59], and osteosarcoma [60]. Elevated circulating miR-106b-3p levels have been found in pancreatic cancer [61], colorectal cancer [62], and hepatocellular carcinoma [63], and in esophageal squamous cell carcinoma cells miR-106b-3p expression has been found to be increased and to induce malignant features [64].

With respect to miR-423-5p, its upregulation in glioma has been associated with enhanced growth, migration, neurosphere formation, invasion, and resistance to temozolomide [65], while in lung cancer it has been linked to brain metastases [66]. In addition, increased serum exosome miR-423-5p levels have been associated with the promotion of gastric cancer growth and metastasis [67].

We also found significantly higher CSF miR-221-3p levels to differentiate CNS DLBCL from non-malignant CNS diseases. In CNS DLBCL patients, miR-221-3p expression was also higher in CSFs than in tumor brain biopsies. Noteworthy, miR-221-3p upregulation has been reported in ABC DLBCL [68], and CNS DLBCL mostly belongs to the ABC subgroup [69,70]. Exosomal miR-221-3p in breast cancer [71] and glioma [72] has been implicated in drug resistance.

Ontological analyses demonstrated significant associations between the CNS DLBCL-specific set of miRs (CSF- and/or brain biopsy-derived) with CNS diseases and various DLBCL subtypes as well as with numerous biological processes, pathways, and molecular functions, the latter including *Anaphase-promoting complex binding*. Interestingly, a recent study pointed to anaphase-promoting complex as a new promising treatment target in DLBCL and mantle cell lymphoma [73]. Concordantly, other analyses of miR–target interactions in CSFs and brain biopsies of CNS DLBCL patients identified a significant abundance of miRs related to the genes responsible for functioning of B lymphocytes and the pathogenesis of B-NHLs, among others. Taken together, these findings support the relevance of the identified set of miRs to the underlying molecular CNS DLBCL pathogenesis and point to the potential of those miRs as diagnostic biomarkers.

Many biological processes regulated by the CSF- and brain biopsy-derived CNS DLBCL-specific set of miRs were found to be related to cellular metabolism. This is in line with the previously postulated tumor metabolism-dependent shaping of the B-NHL microenvironment, which influence tumor progression [74].

Moreover, we revealed that the expression of miRs of the identified CNS DLBCL-specific profiles may be affected by a group of small molecules/drugs and bioactive substances, including arsenic trioxide and epigenetic drugs (histone deacetylase inhibitors (HDACis), LAQ824 (dacinostat), ITF2357 (givinostat), Vorinostat, DNA methyltransferase inhibitors (DNMTis), Decitabine, Azacitidine, and Temozolomide). The combination of arsenic trioxide with other compounds proved efficient in various hematological and lymphoid malignancies, including acute promyelocytic leukemia [75], primary effusion lymphoma [76], and adult T-cell leukemia/lymphoma [77]. HDACis and DNMTis demonstrated promising anticancer activities in both hematological and lymphoid malignancies and solid tumors [78,79] and Vorinostat (SAHA) has been approved for treating primary cutaneous T-cell lymphoma [80]. Therapeutic efficacy of HDACis and DNMTis is still under investigation, especially in combination with other cancer drugs [81,82,83,84,85,86]. Interestingly, it has recently been found that HDAC enhanced the therapeutic effects of methotrexate in PCNSL [87]. The identified miR profiles provide suggestions on potential new therapeutic options for PCNSL.

## 5. Conclusions

We discovered specific patterns of CSF miRs and brain tumor miRs differentiating CNS DLBCL from n-ML. A set of 17 miRs, no matter if assessed in CSF or in a tumor, differentiated CNS DLBCL from non-malignant CNS diseases. These miRs are probably linked to the pathogenesis of CNS DLBCL and have biomarker potential. Assessment of a few selected miRs in a CSF might provide a less invasive alternative to brain biopsy and might serve as a diagnostic tool for patients who do not qualify for brain biopsy.

Further studies are necessary to validate the biomarker potential of miRs on an independent set of samples, and to assess the diagnostic power of miRs in patients following a common initial steroid treatment known to hinder PCNSL diagnosis based on stereotactic brain biopsy [4,5] because of vanishing tumors.

## Figures and Tables

**Table 1:** Pathomorphological characteristics of the CNS DLBCL samples. Cerebrospinal fluids (F), n = 9; brain tumor samples (T), n = 10.

Sample ID CSF/Tumor	Patient Age/Sex	Histopathology	IHC	
			Cell of Origin	BCL2 Expression
F1/T1	63/f	CNS DLBCL	ABC	(++)h
F2/T2	36/m	CNS DLBCL	GCB	(++)h
F3/T3	55/m	CNS DLBCL	ABC	(++)h
F4/T4	73/f	CNS DLBCL	ABC	(+)
F5/T5	51/f	CNS DLBCL	ABC	(+)
F6/T6	56/f	IVLBCL with CNS involvement	GCB	(−)
F7/T7	49/f	CNS DLBCL	ABC	(++)h
F8/T8	31/f	CNS DLBCL	GCB	(+)
F9/T9	42/m	CNS DLBCL	ABC	(+)
–/T10	63/f	CNS DLBCL	CD5(+)	(+)

m, male; f, female; histopathology: primary histopathological diagnosis of central nervous system (CNS) tumor; IHC, immunohistochemical examination; cell of origin: GCB, germinal center B-cell type; ABC, activated B-cell type (non-GCB); CD5(+), CD5 positive immunohistochemical subgroup; BCL2 expression: (++)h, higher BCL2 staining than in T lymphocytes; (+) BCL2 staining similar to or weaker than in T lymphocytes; (−) no BCL2 staining in neoplastic cells; IVLBCL, intravascular large B-cell lymphoma.

**Table 2:** Pathomorphological characteristics of samples from patients with non-malignant CNS diseases. Cerebrospinal fluid (FN, fluid non-malignant), n = 12; tumor (TN, tumor non-malignant), n = 10.

Sample ID	Age/Sex	ICD10 (Diagnosis)/HP	Cytology
**CSFs**			
1FN	40/m	G35 (SM)	L, M
2FN	81/f	D43.1 (meningioma)	L, M
3FN	48/f	G37.9	L, M
4FN	30/f	G35 (SM)	L, M, Neu
5FN	36/m	G37.9	L
6FN	48/m	G04.9 (ADEM)	L, M
7FN	59/m	G04.8	L
8FN	49/f	D33.1	L, M, E
9FN	16/f	G04.0	E, D
10FN	31/f	D33	L, M
11FN	38/m	I67.7	L, M, E
12FN	37/f	D33/reaction process. gliosis	L, M
**Tumors**			
11TN	38/m	D33/reaction process. gliosis	-
12TN	35/f	reaction process. gliosis	-
13TN	41/f	hematoma. reaction process	-
14TN	40/m	Gliosis	-
15TN	54/f	hematoma	-
16TN	65/m	reaction process	-
17TN	59/m	cerebral hemispheres tissue	-
18TN	28/f	focal cortical dysplasia (FCD) type IIB	-
19TC	30/f	ischemic necrosis. gliosis	-
20TC	47/m	Hematoma	-

m, male; f, female. ICD-10, clinical diagnosis of non-neoplastic CNS tumors according to the International Classification of Diseases. Tenth Revision. Clinical Modification. Diseases of the nervous system codes: D33, Benign neoplasm of brain and other parts of central nervous system; D33.1, Benign neoplasm of brain. infratentorial; D43.1, Neoplasm of uncertain behavior of brain. infratentorial; G04.0, Encephalomyelitis disseminatus acuta; G04.8, Other encephalitis. myelitis and encephalomyelitis; G04.9, Encephalitis. myelitis and encephalomyelitis, unspecified; ADEM, Acute Disseminated Encephalomyelitis; G35, Multiple sclerosis (SM); G37.9, Demyelinating disease of central nervous system, unspecified; I67.7, Central nervous system vasculitis (cerebral NEC); HP, histopathological diagnosis of non-neoplastic CNS tumor; cytological smears: L, lymphocytes; M, macrophages; D debris; E erythrocytes; Neu, neutrophils.

**Table 3:** Seventeen miRs common for cerebrospinal fluid and tumor samples, significantly differentiating CNS DLBCL and n-ML tumors. RT-qPCR validation of the NGS results.

miRs	Tumor Samples						CSF Samples					
	NGS			RT-qPCR			NGS			RT-qPCR		
	q*-Value	FC (CNS DLBCL /n-ML)	ROC (AUC)	Expression Level in CNS DLBCL (Median)	Expression Level in n-ML (Median)	*p*-Value	q-Value	FC (CNS DLBCL /n-ML)	ROC (AUC)	Expression Level in CNS DLBCL (Median)	Expression Level in n-ML (Median)	*p*-Value
**miR-9-3p**	***p* < 0.001**	0.24	0.86	0.15	0.69	0.0643	**0.0165**	0.14	0.84	0.26	0.39	0.3030
**miR-9-5p**	***p* < 0.001**	0.22	0.89	1.33	3.09	**0.0455**	**0.0299**	0.15	0.79	0.54	0.77	0.6965
**miR-15a-5p**	**0.0132**	2.05	0.81	1.49	0.56	***p* < 0.001**	**0.0095**	5.31	0.58	2.44	1.86	0.4532
**miR-15b-5p**	***p* < 0.001**	5.23	0.87	0.67	0.26	**0.0022**	**0.0198**	5.24	0.79	1.09	0.81	0.2713
**miR-16-5p**	***p* < 0.001**	2.57	0.93	4.20	1.36	***p* < 0.001**	**0.0067**	4.34	0.72	5.86	1.65	**0.0251**
**miR-17-3p**	***p* < 0.001**	5.37	0.9	0.07	0.02	***p* < 0.001**	**0.0198**	16.11	0.73	0.09	0.02	0.0601
**miR-19a-3p**	***p* < 0.001**	6.61	0.96	0.05	0.01	***p* < 0.001**	***p* < 0.001**	4.79	0.96	0.20	0.11	0.1902
**miR-19b-3p**	***p* < 0.001**	5.06	0.95	0.14	0.03	**0.0022**	**0.0095**	3.16	0.73	0.86	0.29	0.5961
**miR-20a-5p**	***p* < 0.001**	8.96	0.98	0.39	0.07	***p* < 0.001**	**0.0334**	5.71	0.68	0.36	0.15	0.1902
**miR-21-5p**	**0.0311**	2.78	0.83	4.86	0.52	**0.0022**	**0.0165**	3.40	0.76	5.90	3.77	**0.0512**
**miR-25-3p**	***p* < 0.001**	3.45	0.83	0.71	0.21	***p* < 0.001**	**0.0260**	12.46	0.37	0.60	0.36	0.1260
**miR-26a-5p**	**0.0097**	0.65	0.89	2.45	3.30	0.1211	**0.0337**	0.40	0.76	1.12	1.39	0.4122
**miR-92a-3p**	***p* < 0.001**	5.77	0.98	1.98	0.53	**0.001**	**0.0219**	6.08	0.61	6.76	0.88	**0.0357**
**miR-106b-3p**	***p* < 0.001**	7.09	1	0.02	0.01	**0.0073**	**0.0253**	19.51	0.32	0.05	0.02	**0.0466**
**miR-148a-3p**	**0.0010**	6.21	0.89	0.39	0.04	***p* < 0.001**	**0.0095**	9.45	0.70	0.57	0.71	0.6965
**miR-210-3p**	***p* < 0.001**	4.17	0.84	0.12	0.05	**0.0036**	**0.0198**	10.48	0.68	0.17	0.14	0.3030
**miR-423-5p**	**0.0125**	1.80	0.85	1.75	0.63	**0.0057**	**0.0115**	4.15	0.68	2.44	1.29	**0.0424**

CNS DLBCL, Central nervous system primary diffuse large B-cell lymphoma; n-ML, non-malignant brain lesions; CSF, cerebrospinal fluid; FC, fold change; ROC, Receiver Operating Characteristic; AUC, Area Under Curve; * *p*-value after the Benjamini–Hochberg adjustment.

## Supplementary Materials

The following are available online at https://www.mdpi.com/article/10.3390/biom11091395/s1, Table S1: A list of miRNAs verified by RT qPCR and TaqMan Advanced miRNA Assay; Table S2: Databases used for the microRNA enrichment analysis and by the annotation tool (miEAA); Table S3: A list of CSF and tumor miRs significantly differentiating CNS DLBCL and n-ML diseases; Table S4: Newly identified miRNAs; Table S5: CNS DLBCL-specific CSF microRNAs and biological processes; Table S6: CNS DLBCL-specific CSF microRNAs and diseases; Table S7: Drugs potentially affecting CNS DLBCL-specific CSF/tumor miRNAs; Table S8: Sources of CNS DLBCL-specific CSF microRNAs; Table S9: Molecular functions of CNS DLBCL-specific CSF microRNAs; Table S10: CNS DLBCL-specific CSF microRNAs and signaling pathways; Table S11: Top genes strongly associated with the CNS DLBCL-specific miRNA set; Table S12: CNS DLBCL-specific tumor microRNAs and biological processes; Table S13: CNS DLBCL-specific FFPET microRNAs and diseases. Lymphoma and central nervous system diseases are marked bold; Table S14: Forms and sources of CNS DLBCL-specific tumor microRNAs; Table S15: Molecular functions of CNS DLBCL-specific tumor microRNAs.
