# Supplementary material for: A Set of 17 microRNAs Common for Brain and Cerebrospinal Fluid Differentiates Primary Central Nervous System Lymphoma from Non-Malignant Brain Tumors
Source: Biomolecules. 2021 Sep 21;11(9):1395. doi: 10.3390/biom11091395 (PMC8466839; doi:10.3390/biom11091395)
Supplement: Supplementary file 1 [file biomolecules-11-01395-s001.zip › Table S1.pdf]

Supplementary Table S1

A list of miRNAs verified by RT-qPCR (TaqMan Advanced miRNA Assay).

**TaqMan Advanced miRNA Assay**	**ID number Applied Biosystems CN A25576**
miR-9-5p	ID 478214_mir
miR-9-3p*	ID 478211_mir
miR-15a-5p	ID 477858_mir
miR-15b-5p	ID 478313_mir
miR-16-5p	ID 477860_mir
miR-17-3p	ID 477932_mir
miR-19a-3p	ID 479228_mir
miR-19b-3p	ID 478264_mir
miR-20a-5p	ID 478586_mir
miR-21-5p	ID 477973_mir
miR-25-3p	ID 477994_mir
miR-26a-5p	ID 477995_mir
miR-92a-3p	ID 477827_mir
miR-106b-3p	ID 477866_mir
miR-148a-3p	ID 477814_mir
miR-210-3p	ID 477970_mir
miR-221-3p	ID 477981_mir
miR-423-5p	ID 478090_mir
**Internal control for tumor samples**	
miR-24-3p	ID 477992_mir
**Internal controls for CSF samples**	
miR-23a-3p	ID 478532_mir
miR-24-3p	ID 477992_mir
