# Supplementary material for: A Set of 17 microRNAs Common for Brain and Cerebrospinal Fluid Differentiates Primary Central Nervous System Lymphoma from Non-Malignant Brain Tumors
Source: Biomolecules. 2021 Sep 21;11(9):1395. doi: 10.3390/biom11091395 (PMC8466839; doi:10.3390/biom11091395)
Supplement: Supplementary file 1 [file biomolecules-11-01395-s001.zip › Table S10.pdf]

Supplementary Table S10

CNS DLBCL-specific CSF microRNAs and signaling pathways. Processes specific for carcinogenesis and lymphoma-related disorders, are marked bold.

Reactome (miRPathDB)

No	subcategory	q-value*	number of miRs observed	hsa-miRs
**1**	**PTEN Regulation**	**8.495E-07**	**11**	**19a-3p; 16-5p; 148a-3p; 15a-5p; 19b-3p; 221-3p; 21-5p; 9-3p; 92a-3p; 20a-5p; 26a-5p**
2	Intracellular signaling by second messengers	2.889E-06	12	19a-3p; 16-5p; 148a-3p; 15a-5p; 19b-3p; 221-3p; 21-5p; 9-3p; 15b-5p; 92a-3p; 20a-5p; 26a-5p
**3**	**PIP3 activates AKT signaling**	**2.889E-06**	**12**	**19a-3p; 16-5p; 148a-3p; 15a-5p; 19b-3p; 221-3p; 21-5p; 9-3p; 15b-5p; 92a-3p; 20a-5p; 26a-5p**
**4**	**Transcriptional Regulation by TP53**	**2.889E-06**	**12**	**19a-3p; 16-5p; 148a-3p; 15a-5p; 19b-3p; 221-3p; 21-5p; 15b-5p; 17-3p; 92a-3p; 20a-5p; 26a-5p**
**5**	**Signal Transduction**	**7.223E-06**	**12**	**19a-3p; 16-5p; 148a-3p; 15a-5p; 19b-3p; 221-3p; 21-5p; 9-3p; 15b-5p; 9-5p; 20a-5p; 26a-5p**
6	Immune System	3.434E-05	10	19a-3p; 16-5p; 148a-3p; 15a-5p; 221-3p; 21-5p; 9-3p; 15b-5p; 17-3p; 20a-5p
7	Oxidative Stress Induced Senescence	5.562E-05	11	19a-3p; 16-5p; 15a-5p; 221-3p; 423-5p; 21-5p; 9-3p; 15b-5p; 92a-3p; 20a-5p; 26a-5p
**8**	**Signaling by TGF-beta family members**	**6.878E-05**	**9**	**19a-3p; 16-5p; 148a-3p; 15a-5p; 19b-3p; 21-5p; 15b-5p; 92a-3p; 20a-5p**
**9**	**Pre-NOTCH Expression and Processing**	**9.273E-05**	**9**	**16-5p; 148a-3p; 15a-5p; 221-3p; 9-3p; 15b-5p; 92a-3p; 9-5p; 20a-5p**
10	Estrogen-dependent gene expression	0.0001196	10	19a-3p; 16-5p; 148a-3p; 15a-5p; 221-3p; 21-5p; 15b-5p; 17-3p; 92a-3p; 26a-5p
11	Intra-Golgi and retrograde Golgi-to-ER traffic	0.0001196	5	16-5p; 148a-3p; 15a-5p; 15b-5p; 92a-3p
12	ESR-mediated signaling	0.0001264	11	19a-3p; 16-5p; 148a-3p; 15a-5p; 221-3p; 21-5p; 15b-5p; 17-3p; 92a-3p; 9-5p; 26a-5p
13	Deubiquitination	0.0001283	8	19a-3p; 16-5p; 15a-5p; 19b-3p; 221-3p; 15b-5p; 92a-3p; 20a-5p
**14**	**Regulation of RUNX1 Expression and Activity**	**0.0001283**	**10**	**19a-3p; 16-5p; 148a-3p; 15a-5p; 19b-3p; 221-3p; 21-5p; 15b-5p; 20a-5p; 26a-5p**
**15**	**Mitotic G1-G1S phases**	**0.0001313**	**8**	**16-5p; 15a-5p; 221-3p; 423-5p; 15b-5p; 92a-3p; 20a-5p; 26a-5p**
16	Vesicle-mediated transport	0.0002082	7	19a-3p; 16-5p; 148a-3p; 15a-5p; 15b-5p; 92a-3p; 20a-5p

KEGG (miRPathDB)

No	subcategory	q-value	number of miRs observed	hsa-miRs
1	Prostate cancer	9.729E-05	13	19a-3p; 16-5p; 148a-3p; 15a-5p; 221-3p; 21-5p; 9-3p; 15b-5p; 92a-3p; 25-3p; 9-5p; 20a-5p; 26a-5p
**2**	**MicroRNAs in cancer**	**0.0001047**	**14**	**19a-3p; 16-5p; 148a-3p; 15a-5p; 221-3p; 21-5p; 9-3p; 15b-5p; 17-3p; 92a-3p; 25-3p; 9-5p; 20a-5p; 26a-5p**
3	Small cell lung cancer	0.0001047	10	19a-3p; 16-5p; 148a-3p; 15a-5p; 221-3p; 21-5p; 9-3p; 15b-5p; 20a-5p; 26a-5p
4	Viral carcinogenesis	0.0001047	11	19a-3p; 16-5p; 148a-3p; 15a-5p; 221-3p; 423-5p; 9-3p; 15b-5p; 92a-3p; 25-3p; 26a-5p
5	Hepatitis B	0.0004127	11	19a-3p; 16-5p; 148a-3p; 15a-5p; 221-3p; 21-5p; 9-3p; 15b-5p; 25-3p; 20a-5p; 26a-5p
**6**	**Pathways in cancer**	**0.0005077**	**12**	**19a-3p; 16-5p; 148a-3p; 15a-5p; 221-3p; 21-5p; 9-3p; 15b-5p; 92a-3p; 9-5p; 20a-5p; 26a-5p**
7	HTLV-I infection	0.0006449	9	19a-3p; 16-5p; 148a-3p; 15a-5p; 221-3p; 21-5p; 15b-5p; 9-5p; 26a-5p

8	Melanoma	0.0006449	10	19a-3p; 16-5p; 15a-5p; 221-3p; 21-5p; 9-3p; 15b-5p; 25-3p; 20a-5p; 26a-5p
**9**	**p53 signaling pathway**	**0.0006449**	**10**	**19a-3p; 16-5p; 148a-3p; 15a-5p; 19b-3p; 221-3p; 21-5p; 15b-5p; 20a-5p; 26a-5p**
10	Bladder cancer	0.0008959	9	19a-3p; 16-5p; 148a-3p; 221-3p; 21-5p; 9-3p; 15b-5p; 25-3p; 20a-5p
**11**	**Cell cycle**	**0.0008959**	**10**	**16-5p; 148a-3p; 15a-5p; 221-3p; 423-5p; 21-5p; 15b-5p; 92a-3p; 20a-5p; 26a-5p**
12	Influenza A	0.0008959	6	19a-3p; 16-5p; 15a-5p; 221-3p; 21-5p; 15b-5p
13	Toxoplasmosis	0.0009062	6	19a-3p; 148a-3p; 15a-5p; 21-5p; 9-3p; 15b-5p
14	Epstein-Barr virus infection	0.0009714	8	19a-3p; 16-5p; 148a-3p; 15a-5p; 221-3p; 21-5p; 15b-5p; 92a-3p
15	Acute myeloid leukemia	0.0014885	7	19a-3p; 16-5p; 148a-3p; 15a-5p; 221-3p; 9-3p; 15b-5p
16	Chronic myeloid leukemia	0.0014885	10	19a-3p; 16-5p; 148a-3p; 15a-5p; 221-3p; 21-5p; 9-3p; 15b-5p; 20a-5p; 26a-5p
17	Measles	0.0014885	7	19a-3p; 16-5p; 15a-5p; 221-3p; 21-5p; 15b-5p; 26a-5p
18	Pancreatic cancer	0.0014885	9	19a-3p; 16-5p; 148a-3p; 15a-5p; 221-3p; 21-5p; 9-3p; 15b-5p; 20a-5p
19	Hippo signaling pathway	0.0028012	7	19a-3p; 16-5p; 148a-3p; 15a-5p; 221-3p; 21-5p; 15b-5p
20	Non-small cell lung cancer	0.0029488	8	19a-3p; 16-5p; 15a-5p; 221-3p; 21-5p; 9-3p; 15b-5p; 26a-5p
21	Prolactin signaling pathway	0.0029488	7	19a-3p; 19b-3p; 221-3p; 21-5p; 9-3p; 15b-5p; 26a-5p
22	Colorectal cancer	0.0046909	8	19a-3p; 16-5p; 148a-3p; 15a-5p; 221-3p; 21-5p; 15b-5p; 20a-5p
**23**	**FoxO signaling pathway**	**0.0046909**	**9**	**19a-3p; 16-5p; 148a-3p; 15a-5p; 221-3p; 21-5p; 15b-5p; 9-5p; 26a-5p**
24	Insulin signaling pathway	0.0050551	6	19a-3p; 16-5p; 15a-5p; 9-3p; 15b-5p; 26a-5p
**25**	**Neurotrophin signaling pathway**	**0.0050551**	**7**	**19a-3p; 16-5p; 148a-3p; 15a-5p; 21-5p; 9-3p; 15b-5p**
**26**	**PI3K-Akt signaling pathway**	**0.0065548**	**8**	**19a-3p; 16-5p; 148a-3p; 15a-5p; 221-3p; 21-5p; 15b-5p; 26a-5p**
27	Oocyte meiosis	0.0065806	5	16-5p; 15a-5p; 9-3p; 15b-5p; 26a-5p
**28**	**TNF signaling pathway**	**0.0065806**	**5**	**19a-3p; 15a-5p; 221-3p; 21-5p; 9-3p**
29	Endometrial cancer	0.0105322	7	19a-3p; 15a-5p; 221-3p; 21-5p; 9-3p; 15b-5p; 25-3p
**30**	**Ubiquitin mediated proteolysis**	**0.0114871**	**3**	**16-5p; 15a-5p; 15b-5p**
31	Glioma	0.0115167	8	19a-3p; 16-5p; 221-3p; 21-5p; 9-3p; 15b-5p; 20a-5p; 26a-5p
32	Non-alcoholic fatty liver disease NAFLD	0.0115167	4	19a-3p; 148a-3p; 221-3p; 21-5p
33	Proteoglycans in cancer	0.0116473	8	19a-3p; 16-5p; 148a-3p; 221-3p; 21-5p; 9-3p; 15b-5p; 26a-5p
34	Leishmaniasis	0.0116859	3	21-5p; 9-3p; 15b-5p
**35**	**RNA degradation**	**0.0116859**	**3**	**19a-3p; 148a-3p; 92a-3p**
36	Renal cell carcinoma	0.0142412	6	19a-3p; 16-5p; 15a-5p; 21-5p; 9-3p; 15b-5p
**37**	**Wnt signaling pathway**	**0.0147174**	**5**	**19a-3p; 148a-3p; 15a-5p; 15b-5p; 26a-5p**
**38**	**TGF-beta signaling pathway**	**0.0174426**	**5**	**19a-3p; 148a-3p; 21-5p; 15b-5p; 20a-5p**
39	Shigellosis	0.017775	4	19a-3p; 148a-3p; 15a-5p; 9-3p
40	Pathogenic Escherichia coli infection	0.0188539	4	221-3p; 423-5p; 92a-3p; 25-3p
41	Chagas disease American trypanosomiasis	0.0199664	4	19a-3p; 21-5p; 9-3p; 15b-5p
**42**	**ErbB signaling pathway**	**0.0203374**	**6**	**19a-3p; 16-5p; 15a-5p; 221-3p; 9-3p; 15b-5p**
43	Tuberculosis	0.0277519	3	221-3p; 21-5p; 9-3p
44	T cell receptor signaling pathway	0.03574	4	19a-3p; 15a-5p; 9-3p; 15b-5p
45	Focal adhesion	0.049707	6	19a-3p; 16-5p; 148a-3p; 15a-5p; 221-3p; 15b-5p
46	Hepatitis C	0.049707	4	19a-3p; 148a-3p; 221-3p; 9-3p
47	Toll-like receptor signaling pathway	0.049707	3	19a-3p; 21-5p; 9-3p

*p-value after the Benjamini-Hochberg adjustment
