# Supplementary material for: A Set of 17 microRNAs Common for Brain and Cerebrospinal Fluid Differentiates Primary Central Nervous System Lymphoma from Non-Malignant Brain Tumors
Source: Biomolecules. 2021 Sep 21;11(9):1395. doi: 10.3390/biom11091395 (PMC8466839; doi:10.3390/biom11091395)
Supplement: Supplementary file 1 [file biomolecules-11-01395-s001.zip › Table S11.pdf]

Supplementary Table S11

Top genes strongly associated with the CNS DLBCL-specific miRNA set.

Target genes (miRTarBase)

CSF miRNAs

No	GENES	q-value*	number of miRs observed	hsa-miRs	Biological processes/Molecular function
1	*MCM3AP-AS1*	9.97443E-08	5	19a-3p; 16-5p; 15a-5p; 19b-3p; 15b-5p	lncRNA
2	*SLC9A6*	9.97443E-08	6	19a-3p; 16-5p; 15a-5p; 19b-3p; 21-5p; 15b-5p	Ion transport. regulation of neurotrophin TRK receptor and brain-derived neurotrophic factor receptor signaling pathway. synapse organization
3	*TFB1M*	9.97443E-08	5	19a-3p; 16-5p; 15a-5p; 19b-3p; 15b-5p	mitochondrial methyltransferase. rRNA modification. mitochondrion organization
4	*ITPR1*	1.34207E-07	7	19a-3p; 16-5p; 15a-5p; 19b-3p; 15b-5p; 92a-3p; 25-3p	Ion transport. inositol phosphate-mediated signaling. apoptotic process. response to hypoxia
5	*C2orf42*	1.95905E-07	5	19a-3p; 16-5p; 15a-5p; 19b-3p; 15b-5p	lncRNA
6	*RCOR1*	1.95905E-07	8	19a-3p; 16-5p; 15a-5p; 19b-3p; 9-3p; 15b-5p; 25-3p; 26a-5p	neural cell differentiation. negative regulation of transcription. DNA-templated. histone H4 deacetylation
7	*DICER1*	2.00163E-07	9	19a-3p; 16-5p; 148a-3p; 15a-5p; 19b-3p; 221-3p; 21-5p; 15b-5p; 9-5p	RNA-induced silencing complex (RISC) - regulation of transcription. NIK/NF-kappaB signaling. nerve development. positive regulation of myelination. negative regulation of TNF production
8	*NFKB1*	3.76805E-07	6	16-5p; 15a-5p; 21-5p; 9-3p; 92a-3p; 9-5p	proliferation and survival of normal and pathological B cells. negative regulation of transcription. apoptotic process. stress-activated MAPK cascade and positive regulation of canonical Wnt signaling pathway
9	*GIT2*	6.50888E-07	5	19a-3p; 19b-3p; 210-3p; 92a-3p; 26a-5p	GTPase-activating protein. regulation of G protein-coupled receptor signaling pathway
10	*PTEN*	6.50888E-07	10	19a-3p; 19b-3p; 221-3p; 21-5p; 17-3p; 92a-3p; 106b-3p; 25-3p; 20a-5p; 26a-5p	PI3K-AKT-mTOR. regulation of ERK1 and ERK2 cascade. Wnt signaling pathway. regulation of B cell apoptotic process. neuron-neuron synaptic transmission; CNS development. myelin maintenance; cell migration. proliferation. differentiation
11	*DENND6A*	1.78784E-06	7	19a-3p; 16-5p; 15a-5p; 19b-3p; 15b-5p; 210-3p; 92a-3p	endocytic recycling pathway. positive regulation of cell-cell adhesion mediated by cadherin
12	*DDX3Y*	0.000001846	6	19a-3p; 16-5p; 15a-5p; 19b-3p; 221-3p; 15b-5p	cell differentiation. cellular growth and division. alteration of RNA secondary structure: embryogenesis. spermatogenesis
13	*RASA1*	0.000001846	5	19a-3p; 19b-3p; 21-5p; 92a-3p; 26a-5p	RAS/MAPK signaling pathway .negative regulation of cell-matrix adhesion. negative regulation of (neuron) apoptotic process. regulation of GTPase activity
14	*ARCN1*	2.27629E-06	6	16-5p; 15a-5p; 15b-5p; 92a-3p; 20a-5p; 26a-5p	vesicle structure or vesicle trafficking.
15	*RASSF5*	2.56697E-06	5	19a-3p; 16-5p; 15a-5p; 19b-3p; 15b-5p	tumor suppressor. lymphocyte adhesion. negative regulation of cell population proliferation. positive regulation of protein ubiquitination. apoptotic process
16	*POU2AF1*	2.8472E-06	4	16-5p; 15a-5p; 15b-5p; 210-3p	response of B-cells to antigens. formation of germinal centers (GC B cell differentiation). positive regulation of interleukin-6 production. positive regulation of transcription by RNA polymerase II
17	*BTBD7*	4.07289E-06	6	19a-3p; 19b-3p; 21-5p; 92a-3p; 25-3p; 20a-5p	reducing cell-cell adhesion

18	*MAP2K3*	5.33787E-06	7	19a-3p; 16-5p; 15a-5p; 19b-3p; 21-5p; 15b-5p; 20a-5p	MAP kinase-mediated signaling cascade (proliferation. differentiation. transcription regulation and development)
19	*SMAD7*	5.33787E-06	7	16-5p; 15a-5p; 21-5p; 15b-5p; 92a-3p; 25-3p; 20a-5p	TGFBR1 degradation
20	*CHEK1*	5.85529E-06	7	19a-3p; 16-5p; 15a-5p; 19b-3p; 15b-5p; 92a-3p; 26a-5p	inhibition of DNA damage detection and repair
21	*DDX3X*	6.03052E-06	9	19a-3p; 16-5p; 15a-5p; 19b-3p; 21-5p; 15b-5p; 92a-3p; 25-3p; 26a-5p	NFKB signaling pathway
22	*SLC9A1*	6.23677E-06	6	19a-3p; 16-5p; 15a-5p; 19b-3p; 15b-5p; 92a-3p	plasma membrane transporter. regulating pH homeostasis. cell migration and cell volume and tumor growth.
23	*CPEB3*	6.72728E-06	7	16-5p; 15a-5p; 21-5p; 15b-5p; 92a-3p; 25-3p; 26a-5p	RNA-binding protein (translational repressor)
24	*NOTCH2*	7.37889E-06	7	16-5p; 15a-5p; 9-3p; 15b-5p; 92a-3p; 25-3p; 9-5p	NOTCH signaling pathway
25	*ANKIB1*	8.02984E-06	5	19a-3p; 19b-3p; 92a-3p; 25-3p; 20a-5p	positive regulation of proteasomal ubiquitin-dependent protein catabolic process
26	*TIMP3*	8.02984E-06	5	16-5p; 221-3p; 423-5p; 21-5p; 17-3p	tissue inhibitor of metalloproteinases
27	*AP3M1*	9.36439E-06	6	16-5p; 15a-5p; 423-5p; 21-5p; 15b-5p; 25-3p	intracellular protein transport. endocytosis. anterograde axonal and synaptic vesicle transport. vesicle-mediated transport
28	*CLIC1*	0.0000109	4	221-3p; 92a-3p; 106b-3p; 25-3p	regulation of ion transmembrane transport and cell cycle
29	*NOL4L*	0.0000109	4	16-5p; 15a-5p; 15b-5p; 92a-3p	Nucleolar Protein 4 Like

tumor miRNAs

No	GENES	q-value	number of miRs observed	hsa-miRs	Biological processes/Molecular function
1	*BCL2*	1.96921E-17	35	17-5p; 182-5p; 20a-5p; 96-5p; 18a-5p; 200c-3p; 497-5p; 181c-5p; 15b-5p; 9-5p; 16-5p; 192-5p; 376c-3p; 451a; 125a-5p; 195-5p; 143-3p; hsa-let-7a-5p; 135a-5p; 148a-3p; 30b-5p; 98-5p; 15a-5p; 139-5p; 33b-5p; 181d-5p; 7-5p; 204-5p; 494-3p; 21-5p; 136-5p; 153-3p; 211-5p; 125b-5p; 126-3p	apoptosis. lymphoid progenitor cell differentiation. B cell homeostasis
2	*ARCN1*	7.39538E-12	18	92a-3p; 17-5p; 20a-5p; hsa-let-7e-5p; 93-5p; 106b-5p; 15b-5p; 130b-3p; 16-5p; 195-5p; 30c-5p; 98-5p; 15a-5p; 26a-5p; 1-3p; hsa-let-7b-5p; 20b-5p; 770-5p	vesicle-mediated transport
3	*RUNX2*	7.39538E-12	19	155-5p; 497-5p; 30d-5p; 376c-3p; 628-3p; 124-3p; 30a-5p; 195-5p; 203a-3p; 218-5p; 135a-5p; 30c-5p; 30b-5p; 484; 433-3p; 204-5p; 30a-3p; 23b-3p; 221-5p	cell (neuron) differentiation. proliferation. maturation; regulation of transcription. DNA-templated regulation of FGFR signaling pathway
4	*NOTCH2*	1.51097E-11	26	92a-3p; 17-5p; 182-5p; 155-5p; 96-5p; hsa-let-7c-5p; 183-5p; 744-5p; 130b-5p; 497-5p; 146a-5p; 15b-5p; 25-3p; 9-5p; 16-5p; 9-3p; 191-5p; 195-5p; 543; 29b-1-5p; 1248; 15a-5p; 106a-5p; 1-3p; 27b-3p; 23b-3p	NOTCH signaling pathway

5	*MYC*	1.75462E-11	39	92a-3p; 17-5p; 20a-5p; 155-5p; 19a-3p; 19b-1-5p; hsa-let-7c-5p; hsa-let-7e-5p; 92a-1-5p; 744-5p; 16-2-3p; 93-5p; 106b-5p; 25-3p; 16-5p; 30d-5p; 186-5p; 451a; 125a-5p; hsa-let-7a-5p; 135a-5p; 423-5p; 487b-3p; 148a-3p; 30c-5p; 1248; 130a-3p; 98-5p; 129-2-3p; 323a-3p; 33b-5p; 378a-3p; 7-5p; 494-3p; hsa-let-7b-5p; 21-5p; 30a-3p; 145-5p; 92b-3p	proto-oncogene. cell proliferation. apoptosis. regulation of transcription. DNA-templated. MAPK cascade. Notch signaling pathway
6	*APC*	5.46947E-11	15	155-5p; 142-3p; 106b-5p; 210-3p; 192-5p; 186-5p; 218-5p; 135a-5p; 148a-3p; 129-5p; 106a-5p; 27a-3p; 21-5p; 590-3p; 125b-5p	tumor suppressor gene; regulation of cell migration. differentiation. adhesion; cell cycle arrest; positive regulation of cell death; negative regulation of Wnt signaling pathway
7	*EZH2*	2.52009E-10	22	hsa-let-7c-5p; hsa-let-7e-5p; 1260b; 1260a; 93-5p; 25-3p; 30d-5p; 124-3p; hsa-let-7a-5p; 144-3p; 101-5p; 484; 98-5p; 26a-5p; 320c; 138-5p; 532-3p; 340-3p; 27a-3p; hsa-let-7b-5p; 126-3p; 92b-3p	epigenetic regulation
8	*PRPF8*	6.05297E-10	17	92a-3p; 17-5p; hsa-let-7c-5p; hsa-let-7e-5p; 93-5p; 25-3p; 16-5p; 381-3p; 186-5p; 218-5p; 93-3p; 484; 106a-5p; 532-3p; 409-3p; 27b-3p; hsa-let-7b-5p	RNA/mRNA splicing. cellular response to tumor necrosis factor
9	*BMPR2*	9.73221E-10	23	92a-3p; 17-5p; 20a-5p; 19a-3p; 93-5p; 19b-3p; 181c-5p; 106b-5p; 3615; 192-5p; 128-3p; 135a-5p; 93-3p; 130a-3p; 181d-5p; 7-5p; 27b-3p; 27a-3p; 32-5p; 21-5p; 20b-5p; 153-3p; 92b-3p	TGF beta receptor. BMP signaling pathway. cell proliferation (endothelial) growth. migration and differentiation. brain development
10	*AGO1*	1.2065E-09	35	92a-3p; 17-5p; 20a-5p; 19a-3p; hsa-let-7c-5p; hsa-let-7e-5p; 744-5p; 93-5p; 19b-3p; 106b-5p; 130b-3p; 25-3p; 30d-5p; 628-3p; 186-5p; 31-5p; 195-5p; hsa-let-7a-5p; 218-5p; 30c-5p; 99a-5p; 93-3p; 484; 149-5p; 194-5p; 26a-5p; 106a-5p; 423-3p; 320c; 1-3p; 378a-3p; 138-5p; 769-3p; hsa-let-7b-5p; 20b-5p	RNA-induced silencing complex (RISC)
11	*BTBD7*	1.2065E-09	17	92a-3p; 17-5p; 20a-5p; 19a-3p; 142-3p; 93-5p; 19b-3p; 106b-5p; 25-3p; 30d-5p; 30a-5p; 218-5p; 30c-5p; 1248; 30b-5p; 21-5p; 20b-5p	reducing cell-cell adhesion
12	*PTEN*	1.4554E-09	36	92a-3p; 106b-3p; 17-5p; 182-5p; 20a-5p; 155-5p; 18a-5p; 19a-3p; 17-3p; 142-5p; 92a-1-5p; 93-5p; 200c-3p; 19b-3p; 181c-5p; 106b-5p; 130b-3p; 25-3p; 486-5p; 425-5p; 128-3p; 543; 21-3p; 144-3p; 130a-3p; 376a-5p; 26a-5p; 106a-5p; 382-5p; 494-3p; 32-5p; 21-5p; 20b-5p; 153-3p; 23b-3p; 92b-3p	PI3K-AKT-mTOR signaling pathway. regulation of ERK1 and ERK2 cascade. Wnt signaling pathway. regulation of B cell apoptotic process. neuron-neuron synaptic transmission; CNS development. myelin maintenance; cell migration. proliferation. differentiation
13	*IL6*	3.89154E-09	15	155-5p; hsa-let-7c-5p; 142-3p; 146a-5p; 9-5p; 124-3p; 451a; 203a-3p; hsa-let-7a-5p; 98-5p; 149-5p; 26a-5p; 106a-5p; 1-3p; 136-5p	JAK-STAT signaling pathway. positive regulation of TNF. VEGF. positive regulation of epithelial to mesenchymal transition. maintenance of blood-brain barrier. regulation of cell migration. proliferation. differentiation. apoptotic process. translation. positive regulation of B cell activation. regulation of microglial cell activation and glial cell proliferation. neuroinflammatory response
14	*CAMTA1*	5.51732E-09	12	17-5p; 20a-5p; 155-5p; 96-5p; 19a-3p; 93-5p; 19b-3p; 106b-5p; 124-3p; 218-5p; 129-5p; 20b-5p	transcription factor and tumor suppressor

15	*PRKAR1A*	5.51732E-09	19	17-5p; 20a-5p; 155-5p; 96-5p; 1246; 93-5p; 106b-5p; 25-3p; 16-5p; 192-5p; 30d-5p; 30a-5p; 30c-5p; 30b-5p; 106a-5p; 323a-3p; 204-5p; 20b-5p; 1296-5p	cAMP signaling pathways

*p-value after the Benjamini-Hochberg adjustment
