# Supplementary material for: A Set of 17 microRNAs Common for Brain and Cerebrospinal Fluid Differentiates Primary Central Nervous System Lymphoma from Non-Malignant Brain Tumors
Source: Biomolecules. 2021 Sep 21;11(9):1395. doi: 10.3390/biom11091395 (PMC8466839; doi:10.3390/biom11091395)
Supplement: Supplementary file 1 [file biomolecules-11-01395-s001.zip › Table S12.pdf]

Supplementary Table S12

CNS DLBCL-specific tumor microRNAs and biological processes.

MiRPathDB GO Biological process				
**No**	**subcategory**	**q-value***	**number of miRs observed**	**hsa-miRs**
1	cellular protein modification process	8.223E-22	71	92a-3p; 17-5p; 182-5p; 20a-5p; 155-5p; 96-5p; 18a-5p; 19a-3p; hsa-let-7c-5p; 17-3p; 183-5p; 142-3p; 744-5p; 93-5p; 200c-3p; 19b-3p; 582-5p; 497-5p; 146a-5p; 181c-5p; 106b-5p; 15b-5p; 130b-3p; 210-3p; 9-5p; 16-5p; 192-5p; 30d-5p; 124-3p; 30a-5p; 486-3p; 186-5p; 9-3p; 31-5p; 451a; 191-5p; 125a-5p; 195-5p; 203a-3p; 128-3p; hsa-let-7a-5p; 218-5p; 135a-5p; 543; 487b-3p; 148a-3p; 30c-5p; 30b-5p; 132-3p; 130a-3p; 98-5p; 625-5p; 15a-5p; 376a-5p; 26a-5p; 323a-3p; 7-5p; 138-5p; 27b-3p; 27a-3p; 494-3p; 32-5p; 21-5p; 20b-5p; 340-5p; 153-3p; 454-3p; 145-5p; 125b-5p; 126-3p; 92b-3p
2	protein modification process	8.223E-22	71	92a-3p; 17-5p; 182-5p; 20a-5p; 155-5p; 96-5p; 18a-5p; 19a-3p; hsa-let-7c-5p; 17-3p; 183-5p; 142-3p; 744-5p; 93-5p; 200c-3p; 19b-3p; 582-5p; 497-5p; 146a-5p; 181c-5p; 106b-5p; 15b-5p; 130b-3p; 210-3p; 9-5p; 16-5p; 192-5p; 30d-5p; 124-3p; 30a-5p; 486-3p; 186-5p; 9-3p; 31-5p; 451a; 191-5p; 125a-5p; 195-5p; 203a-3p; 128-3p; hsa-let-7a-5p; 218-5p; 135a-5p; 543; 487b-3p; 148a-3p; 30c-5p; 30b-5p; 132-3p; 130a-3p; 98-5p; 625-5p; 15a-5p; 376a-5p; 26a-5p; 323a-3p; 7-5p; 138-5p; 27b-3p; 27a-3p; 494-3p; 32-5p; 21-5p; 20b-5p; 340-5p; 153-3p; 454-3p; 145-5p; 125b-5p; 126-3p; 92b-3p
3	immune system process	1.814E-21	53	92a-3p; 17-5p; 20a-5p; 155-5p; 96-5p; 18a-5p; 19a-3p; 93-5p; 200c-3p; 582-5p; 497-5p; 146a-5p; 106b-5p; 15b-5p; 130b-3p; 25-3p; 9-5p; 16-5p; 30d-5p; 376c-3p; 124-3p; 30a-5p; 9-3p; 31-5p; 451a; 125a-5p; 195-5p; 128-3p; 218-5p; 135a-5p; 543; 148a-3p; 30c-5p; 30b-5p; 130a-3p; 15a-5p; 106a-5p; 139-5p; 320c; 1-3p; 33b-5p; 378a-3p; 138-5p; 27b-3p; 204-5p; 27a-3p; 494-3p; 21-5p; 23b-3p; 145-5p; 125b-5p; 126-3p; 1296-5p
4	positive regulation of metabolic process	1.814E-21	93	92a-3p; 106b-3p; 17-5p; 182-5p; 20a-5p; 155-5p; 96-5p; 18a-5p; 1246; 19a-3p; hsa-let-7c-5p; 17-3p; 183-5p; 142-3p; 92a-1-5p; 744-5p; 93-5p; 200c-3p; 19b-3p; 497-5p; 146a-5p; 181c-5p; 106b-5p; 15b-5p; 130b-3p; 210-3p; 9-5p; 16-5p; 192-5p; 30d-5p; 381-3p; 376c-3p; 124-3p; 30a-5p; 410-3p; 486-3p; 186-5p; 9-3p; 31-5p; 625-3p; 451a; 505-3p; 491-5p; 125a-5p; 195-5p; 203a-3p; 128-3p; 143-3p; hsa-let-7a-5p; 218-5p; 135a-5p; 423-5p; 543; 148a-3p; 656-3p; 30c-5p; 30b-5p; 99a-5p; 144-3p; 101-5p; 484; 130a-3p; 134-5p; 935; 98-5p; 149-5p; 495-3p; 15a-5p; 26a-5p; 106a-5p; 139-5p; 323a-3p; 423-3p; 425-3p; 320c; 1-3p; 378a-3p; 138-5p; 187-3p; 27b-3p; 204-5p; 27a-3p; 494-3p; 369-3p; hsa-let-7b-5p; 21-5p; 20b-5p; 30a-3p; 153-3p; 145-5p; 125b-5p; 126-3p; 1296-5p
5	positive regulation of cellular metabolic process	3.511E-21	88	92a-3p; 106b-3p; 17-5p; 182-5p; 20a-5p; 155-5p; 96-5p; 18a-5p; 1246; 19a-3p; hsa-let-7c-5p; 17-3p; 183-5p; 142-3p; 92a-1-5p; 744-5p; 93-5p; 200c-3p; 19b-3p; 497-5p; 146a-5p; 106b-5p; 15b-5p; 130b-3p; 25-3p; 210-3p; 9-5p; 16-5p; 192-5p; 30d-5p; 381-3p; 376c-3p; 124-3p; 30a-5p; 410-3p; 486-3p; 186-5p; 9-3p; 31-5p; 451a; 505-3p; 491-5p; 125a-5p; 195-5p; 203a-3p; 128-3p; 143-3p; hsa-let-7a-5p; 218-5p; 135a-5p; 423-5p; 543; 148a-3p; 656-3p; 30c-5p; 30b-5p; 99a-5p; 144-3p; 101-5p; 130a-3p; 134-5p; 98-5p; 149-5p; 625-5p; 495-3p; 15a-5p; 26a-5p; 106a-5p; 139-5p; 323a-3p; 425-3p; 320c; 378a-3p; 138-5p; 187-3p; 27b-3p; 204-5p; 27a-3p; 494-3p; hsa-let-7b-5p; 21-5p; 20b-5p; 30a-3p; 153-3p; 145-5p; 125b-5p; 126-3p; 1296-5p

6	protein metabolic process	3.511E-21	67	92a-3p; 106b-3p; 17-5p; 182-5p; 20a-5p; 155-5p; 96-5p; 18a-5p; 19a-3p; hsa-let-7c-5p; 17-3p; 183-5p; 142-3p; 744-5p; 93-5p; 200c-3p; 19b-3p; 497-5p; 146a-5p; 181c-5p; 15b-5p; 130b-3p; 25-3p; 16-5p; 30d-5p; 124-3p; 30a-5p; 486-3p; 186-5p; 31-5p; 625-3p; 451a; 191-5p; 125a-5p; 195-5p; 203a-3p; 128-3p; hsa-let-7a-5p; 218-5p; 423-5p; 543; 487b-3p; 148a-3p; 30c-5p; 30b-5p; 99a-5p; 132-3p; 484; 130a-3p; 149-5p; 625-5p; 15a-5p; 26a-5p; 423-3p; 590-5p; 7-5p; 138-5p; 27b-3p; 27a-3p; 494-3p; 32-5p; hsa-let-7b-5p; 21-5p; 145-5p; 125b-5p; 126-3p; 92b-3p
7	regulation of protein metabolic process	7.597E-21	77	92a-3p; 17-5p; 182-5p; 155-5p; 96-5p; 18a-5p; 19a-3p; hsa-let-7c-5p; 17-3p; 183-5p; 142-3p; 92a-1-5p; 744-5p; 93-5p; 200c-3p; 19b-3p; 582-5p; 497-5p; 146a-5p; 181c-5p; 106b-5p; 15b-5p; 130b-3p; 25-3p; 9-5p; 16-5p; 30d-5p; 376c-3p; 124-3p; 30a-5p; 486-3p; 186-5p; 9-3p; 31-5p; 625-3p; 451a; 191-5p; 125a-5p; 195-5p; 203a-3p; 128-3p; hsa-let-7a-5p; 218-5p; 423-5p; 487b-3p; 148a-3p; 30c-5p; 144-3p; 101-5p; 132-3p; 484; 130a-3p; 149-5p; 625-5p; 495-3p; 15a-5p; 194-5p; 26a-5p; 106a-5p; 423-3p; 320c; 1-3p; 378a-3p; 590-5p; 7-5p; 138-5p; 27b-3p; 204-5p; 27a-3p; 494-3p; hsa-let-7b-5p; 21-5p; 30a-3p; 145-5p; 125b-5p; 126-3p; 92b-3p
8	response to organic substance	7.597E-21	72	92a-3p; 17-5p; 182-5p; 20a-5p; 155-5p; 96-5p; 18a-5p; 19a-3p; hsa-let-7c-5p; 17-3p; 183-5p; 142-3p; 744-5p; 93-5p; 200c-3p; 582-5p; 497-5p; 146a-5p; 106b-5p; 15b-5p; 130b-3p; 25-3p; 9-5p; 16-5p; 381-3p; 124-3p; 30a-5p; 186-5p; 9-3p; 451a; 191-5p; 125a-5p; 195-5p; 203a-3p; 369-5p; 128-3p; 143-3p; 218-5p; 135a-5p; 148a-3p; 30c-5p; 30b-5p; 144-3p; 31-3p; 132-3p; 484; 130a-3p; 98-5p; 15a-5p; 194-5p; 26a-5p; 296-5p; 106a-5p; 423-3p; 320c; 1-3p; 33b-5p; 378a-3p; 138-5p; 27b-3p; 204-5p; 27a-3p; 494-3p; 32-5p; 21-5p; 30a-3p; 153-3p; 23b-3p; 145-5p; 125b-5p; 126-3p; 92b-3p
9	response to growth factor	2.348E-20	60	92a-3p; 17-5p; 182-5p; 155-5p; 96-5p; 18a-5p; 19a-3p; hsa-let-7c-5p; 183-5p; 744-5p; 200c-3p; 19b-3p; 497-5p; 146a-5p; 181c-5p; 15b-5p; 130b-3p; 25-3p; 9-5p; 16-5p; 376c-3p; 124-3p; 30a-5p; 186-5p; 625-3p; 451a; 125a-5p; 195-5p; 203a-3p; 128-3p; 218-5p; 135a-5p; 487b-3p; 148a-3p; 144-3p; 31-3p; 132-3p; 130a-3p; 495-3p; 15a-5p; 194-5p; 26a-5p; 296-5p; 106a-5p; 1-3p; 33b-5p; 378a-3p; 590-5p; 138-5p; 27b-3p; 204-5p; 27a-3p; 494-3p; 32-5p; 21-5p; 30a-3p; 23b-3p; 145-5p; 125b-5p; 126-3p
10	cellular protein metabolic process	4.534E-20	68	92a-3p; 17-5p; 182-5p; 20a-5p; 155-5p; 96-5p; 18a-5p; 19a-3p; hsa-let-7c-5p; 17-3p; 183-5p; 142-3p; 744-5p; 93-5p; 200c-3p; 19b-3p; 497-5p; 146a-5p; 181c-5p; 106b-5p; 15b-5p; 130b-3p; 25-3p; 16-5p; 30d-5p; 124-3p; 30a-5p; 486-3p; 186-5p; 31-5p; 625-3p; 451a; 191-5p; 125a-5p; 195-5p; 203a-3p; 128-3p; hsa-let-7a-5p; 218-5p; 423-5p; 543; 487b-3p; 148a-3p; 30c-5p; 30b-5p; 99a-5p; 101-5p; 132-3p; 484; 130a-3p; 98-5p; 149-5p; 625-5p; 15a-5p; 26a-5p; 423-3p; 7-5p; 138-5p; 27b-3p; 27a-3p; 494-3p; 32-5p; hsa-let-7b-5p; 21-5p; 145-5p; 125b-5p; 126-3p; 92b-3p
11	negative regulation of cell death	4.534E-20	59	17-5p; 182-5p; 155-5p; 96-5p; hsa-let-7c-5p; 183-5p; 92a-1-5p; 744-5p; 200c-3p; 497-5p; 146a-5p; 181c-5p; 15b-5p; 9-5p; 16-5p; 376c-3p; 124-3p; 410-3p; 31-5p; 625-3p; 451a; 191-5p; 491-5p; 125a-5p; 195-5p; 203a-3p; 128-3p; 143-3p; hsa-let-7a-5p; 218-5p; 135a-5p; 543; 487b-3p; 148a-3p; 144-3p; 98-5p; 149-5p; 495-3p; 15a-5p; 26a-5p; 106a-5p; 139-5p; 425-3p; 1-3p; 33b-5p; 138-5p; 127-3p; 27b-3p; 204-5p; 27a-3p; 494-3p; 21-5p; 30a-3p; 153-3p; 23b-3p; 145-5p; 125b-5p; 126-3p; 92b-3p

12	intracellular signal transduction	8.081E-20	59	92a-3p; 17-5p; 182-5p; 20a-5p; 155-5p; 96-5p; 19a-3p; hsa-let-7c-5p; 183-5p; 744-5p; 93-5p; 200c-3p; 19b-3p; 497-5p; 146a-5p; 106b-5p; 15b-5p; 130b-3p; 9-5p; 16-5p; 381-3p; 376c-3p; 124-3p; 186-5p; 31-5p; 625-3p; 451a; 125a-5p; 195-5p; 203a-3p; 128-3p; 218-5p; 135a-5p; 148a-3p; 30c-5p; 99a-5p; 144-3p; 132-3p; 130a-3p; 98-5p; 625-5p; 15a-5p; 194-5p; 26a-5p; 106a-5p; 139-5p; 1-3p; 138-5p; 27b-3p; 204-5p; 27a-3p; 494-3p; 21-5p; 20b-5p; 153-3p; 23b-3p; 145-5p; 125b-5p; 126-3p
13	regulation of cellular protein metabolic process	1.28E-19	75	92a-3p; 17-5p; 182-5p; 20a-5p; 155-5p; 96-5p; 18a-5p; 19a-3p; hsa-let-7c-5p; 17-3p; 183-5p; 142-3p; 744-5p; 93-5p; 200c-3p; 19b-3p; 582-5p; 497-5p; 146a-5p; 181c-5p; 106b-5p; 15b-5p; 130b-3p; 25-3p; 9-5p; 16-5p; 376c-3p; 124-3p; 30a-5p; 410-3p; 486-3p; 186-5p; 31-5p; 625-3p; 451a; 125a-5p; 195-5p; 203a-3p; 128-3p; hsa-let-7a-5p; 218-5p; 487b-3p; 148a-3p; 30c-5p; 144-3p; 101-5p; 132-3p; 484; 130a-3p; 149-5p; 625-5p; 495-3p; 15a-5p; 194-5p; 26a-5p; 106a-5p; 423-3p; 320c; 1-3p; 378a-3p; 590-5p; 7-5p; 138-5p; 27b-3p; 204-5p; 27a-3p; 494-3p; 369-3p; hsa-let-7b-5p; 21-5p; 30a-3p; 145-5p; 125b-5p; 126-3p; 92b-3p
14	negative regulation of programmed cell death	1.946E-19	57	17-5p; 182-5p; 155-5p; 96-5p; hsa-let-7c-5p; 183-5p; 92a-1-5p; 744-5p; 200c-3p; 497-5p; 146a-5p; 181c-5p; 15b-5p; 9-5p; 16-5p; 376c-3p; 124-3p; 30a-5p; 410-3p; 31-5p; 625-3p; 451a; 191-5p; 491-5p; 125a-5p; 195-5p; 203a-3p; 128-3p; 143-3p; hsa-let-7a-5p; 218-5p; 135a-5p; 543; 487b-3p; 148a-3p; 98-5p; 149-5p; 495-3p; 15a-5p; 26a-5p; 106a-5p; 139-5p; 425-3p; 1-3p; 33b-5p; 138-5p; 127-3p; 27b-3p; 204-5p; 27a-3p; 21-5p; 30a-3p; 153-3p; 23b-3p; 145-5p; 125b-5p; 126-3p
15	biosynthetic process	2.531E-19	84	92a-3p; 17-5p; 182-5p; 20a-5p; 155-5p; 96-5p; 18a-5p; 19a-3p; hsa-let-7c-5p; 17-3p; hsa-let-7e-5p; 1260b; 92a-1-5p; 744-5p; 93-5p; 200c-3p; 19b-3p; 582-5p; 497-5p; 146a-5p; 181c-5p; 106b-5p; 15b-5p; 130b-3p; 25-3p; 9-5p; 425-5p; 16-5p; 192-5p; 30d-5p; 381-3p; 30a-5p; 410-3p; 486-3p; 186-5p; 9-3p; 31-5p; 625-3p; 451a; 191-5p; 125a-5p; 195-5p; 203a-3p; 128-3p; 143-3p; hsa-let-7a-5p; 218-5p; 135a-5p; 148a-3p; 30c-5p; 30b-5p; 99a-5p; 144-3p; 484; 130a-3p; 935; 98-5p; 149-5p; 495-3p; 15a-5p; 26a-5p; 106a-5p; 423-3p; 425-3p; 144-5p; 181d-5p; 378a-3p; 138-5p; 27b-3p; 204-5p; 27a-3p; 494-3p; 32-5p; hsa-let-7b-5p; 21-5p; 454-3p; 23b-3p; 331-5p; 145-5p; 125b-5p; 126-3p; 92b-3p; 770-5p; 1296-5p
16	organelle organization	3.286E-19	60	92a-3p; 106b-3p; 17-5p; 182-5p; 20a-5p; 155-5p; 96-5p; 18a-5p; 19a-3p; hsa-let-7c-5p; hsa-let-7e-5p; 92a-1-5p; 744-5p; 93-5p; 200c-3p; 19b-3p; 181c-5p; 106b-5p; 15b-5p; 130b-3p; 25-3p; 9-5p; 16-5p; 192-5p; 30d-5p; 30a-5p; 186-5p; 125a-5p; 195-5p; 203a-3p; 128-3p; 218-5p; 423-5p; 543; 148a-3p; 30c-5p; 30b-5p; 484; 130a-3p; 149-5p; 495-3p; 15a-5p; 26a-5p; 106a-5p; 423-3p; 1-3p; 181d-5p; 138-5p; 187-3p; 27b-3p; 27a-3p; hsa-let-7b-5p; 21-5p; 20b-5p; 153-3p; 99b-3p; 23b-3p; 126-3p; 92b-3p; 1296-5p
17	positive regulation of cellular biosynthetic process	3.709E-19	78	92a-3p; 17-5p; 182-5p; 20a-5p; 155-5p; 18a-5p; 19a-3p; hsa-let-7c-5p; 17-3p; 92a-1-5p; 744-5p; 93-5p; 200c-3p; 19b-3p; 497-5p; 146a-5p; 106b-5p; 15b-5p; 130b-3p; 25-3p; 210-3p; 9-5p; 16-5p; 30d-5p; 381-3p; 124-3p; 30a-5p; 410-3p; 486-3p; 186-5p; 9-3p; 31-5p; 451a; 491-5p; 125a-5p; 195-5p; 203a-3p; 128-3p; hsa-let-7a-5p; 218-5p; 135a-5p; 423-5p; 543; 148a-3p; 656-3p; 30c-5p; 30b-5p; 99a-5p; 144-3p; 484; 130a-3p; 134-5p; 98-5p; 149-5p; 495-3p; 15a-5p; 26a-5p; 106a-5p; 139-5p; 323a-3p; 425-3p; 320c; 378a-3p; 138-5p; 187-3p; 27b-3p; 204-5p; 27a-3p; 494-3p; hsa-let-7b-5p; 21-5p; 20b-5p; 153-3p; 23b-3p; 145-5p; 125b-5p; 126-3p; 1296-5p

18	posttranscriptional regulation of gene expression	3.709E-19	59	92a-3p; 17-5p; 182-5p; 20a-5p; 155-5p; 96-5p; 18a-5p; 19a-3p; 17-3p; hsa-let-7e-5p; 744-5p; 93-5p; 19b-3p; 497-5p; 106b-5p; 15b-5p; 130b-3p; 25-3p; 16-5p; 30a-5p; 410-3p; 486-3p; 186-5p; 625-3p; 451a; 505-3p; 125a-5p; 195-5p; 203a-3p; 128-3p; hsa-let-7a-5p; 218-5p; 148a-3p; 30c-5p; 99a-5p; 93-3p; 484; 130a-3p; 149-5p; 15a-5p; 26a-5p; 106a-5p; 423-3p; 320c; 378a-3p; 590-5p; 138-5p; 27b-3p; 27a-3p; 494-3p; hsa-let-7b-5p; 21-5p; 20b-5p; 454-3p; 23b-3p; 145-5p; 125b-5p; 92b-3p; 1296-5p
19	negative regulation of apoptotic process	3.864E-19	55	17-5p; 182-5p; 155-5p; 96-5p; hsa-let-7c-5p; 183-5p; 92a-1-5p; 744-5p; 200c-3p; 497-5p; 146a-5p; 181c-5p; 15b-5p; 9-5p; 16-5p; 376c-3p; 124-3p; 30a-5p; 31-5p; 625-3p; 451a; 191-5p; 491-5p; 125a-5p; 195-5p; 203a-3p; 128-3p; 143-3p; hsa-let-7a-5p; 218-5p; 135a-5p; 543; 487b-3p; 148a-3p; 98-5p; 149-5p; 495-3p; 15a-5p; 26a-5p; 106a-5p; 139-5p; 1-3p; 33b-5p; 378a-3p; 138-5p; 127-3p; 27b-3p; 27a-3p; 21-5p; 30a-3p; 153-3p; 23b-3p; 145-5p; 125b-5p; 126-3p
20	nitrogen compound metabolic process	4.321E-19	84	92a-3p; 106b-3p; 17-5p; 20a-5p; 155-5p; 96-5p; 18a-5p; 1246; 19a-3p; hsa-let-7c-5p; 17-3p; 183-5p; hsa-let-7e-5p; 92a-1-5p; 744-5p; 93-5p; 200c-3p; 19b-3p; 582-5p; 497-5p; 146a-5p; 766-3p; 181c-5p; 106b-5p; 15b-5p; 130b-3p; 25-3p; 210-3p; 9-5p; 16-5p; 192-5p; 30d-5p; 381-3p; 30a-5p; 186-5p; 31-5p; 625-3p; 451a; 125a-5p; 195-5p; 203a-3p; 128-3p; 143-3p; hsa-let-7a-5p; 218-5p; 135a-5p; 423-5p; 543; 148a-3p; 30c-5p; 30b-5p; 99a-5p; 144-3p; 31-3p; 484; 130a-3p; 935; 98-5p; 149-5p; 625-5p; 15a-5p; 26a-5p; 106a-5p; 139-5p; 423-3p; 144-5p; 181d-5p; 378a-3p; 138-5p; 27b-3p; 27a-3p; 494-3p; 32-5p; hsa-let-7b-5p; 21-5p; 20b-5p; 454-3p; 23b-3p; 145-5p; 125b-5p; 126-3p; 92b-3p; 770-5p; 1296-5p
21	cellular metabolic process	9.242E-19	84	92a-3p; 106b-3p; 17-5p; 20a-5p; 155-5p; 96-5p; 18a-5p; 19a-3p; hsa-let-7c-5p; 17-3p; 183-5p; hsa-let-7e-5p; 1260b; 92a-1-5p; 744-5p; 93-5p; 200c-3p; 19b-3p; 582-5p; 497-5p; 766-3p; 181c-5p; 106b-5p; 15b-5p; 130b-3p; 25-3p; 210-3p; 9-5p; 425-5p; 30d-5p; 381-3p; 30a-5p; 186-5p; 9-3p; 31-5p; 625-3p; 451a; 505-3p; 191-5p; 491-5p; 125a-5p; 195-5p; 203a-3p; 128-3p; 143-3p; hsa-let-7a-5p; 218-5p; 148a-3p; 30c-5p; 30b-5p; 99a-5p; 144-3p; 31-3p; 484; 130a-3p; 935; 98-5p; 149-5p; 495-3p; 15a-5p; 26a-5p; 106a-5p; 423-3p; 425-3p; 320c; 1-3p; 181d-5p; 378a-3p; 138-5p; 27b-3p; 27a-3p; 494-3p; 32-5p; hsa-let-7b-5p; 21-5p; 20b-5p; 340-5p; 454-3p; 23b-3p; 145-5p; 125b-5p; 126-3p; 92b-3p; 1296-5p
22	cellular macromolecule biosynthetic process	9.633E-19	93	92a-3p; 17-5p; 182-5p; 20a-5p; 155-5p; 96-5p; 18a-5p; 1246; 19a-3p; hsa-let-7c-5p; 17-3p; 183-5p; hsa-let-7e-5p; 92a-1-5p; 744-5p; 93-5p; 200c-3p; 19b-3p; 582-5p; 497-5p; 146a-5p; 766-3p; 181c-5p; 106b-5p; 15b-5p; 130b-3p; 25-3p; 9-5p; 425-5p; 16-5p; 192-5p; 30d-5p; 381-3p; 30a-5p; 410-3p; 486-3p; 186-5p; 9-3p; 31-5p; 625-3p; 505-3p; 191-5p; 125a-5p; 195-5p; 203a-3p; 128-3p; 143-3p; hsa-let-7a-5p; 218-5p; 135a-5p; 423-5p; 543; 148a-3p; 656-3p; 30c-5p; 30b-5p; 99a-5p; 144-3p; 484; 130a-3p; 134-5p; 935; 98-5p; 149-5p; 495-3p; 15a-5p; 26a-5p; 106a-5p; 423-3p; 425-3p; 320c; 144-5p; 181d-5p; 378a-3p; 138-5p; 27b-3p; 204-5p; 27a-3p; 494-3p; 376b-3p; 32-5p; hsa-let-7b-5p; 21-5p; 20b-5p; 454-3p; 23b-3p; 331-5p; 145-5p; 125b-5p; 126-3p; 92b-3p; 770-5p; 1296-5p

23	cell cycle	1.181E-18	75	92a-3p; 17-5p; 182-5p; 20a-5p; 155-5p; 96-5p; 18a-5p; 1246; 19a-3p; hsa-let-7c-5p; 183-5p; hsa-let-7e-5p; 92a-1-5p; 744-5p; 93-5p; 200c-3p; 19b-3p; 497-5p; 146a-5p; 181c-5p; 106b-5p; 15b-5p; 130b-3p; 25-3p; 9-5p; 16-5p; 192-5p; 376c-3p; 124-3p; 30a-5p; 186-5p; 625-3p; 451a; 191-5p; 125a-5p; 195-5p; 203a-3p; 128-3p; hsa-let-7a-5p; 218-5p; 487b-3p; 148a-3p; 21-3p; 30c-5p; 30b-5p; 99a-5p; 93-3p; 484; 130a-3p; 149-5p; 495-3p; 15a-5p; 26a-5p; 106a-5p; 323a-3p; 423-3p; 1-3p; 181d-5p; 378a-3p; 138-5p; 187-3p; 885-5p; 27b-3p; 27a-3p; 494-3p; hsa-let-7b-5p; 21-5p; 20b-5p; 340-5p; 454-3p; 145-5p; 125b-5p; 126-3p; 92b-3p; 1296-5p
24	macromolecule metabolic process	1.306E-18	88	92a-3p; 106b-3p; 17-5p; 20a-5p; 155-5p; 96-5p; 18a-5p; 1246; 19a-3p; hsa-let-7c-5p; 17-3p; 183-5p; hsa-let-7e-5p; 92a-1-5p; 744-5p; 93-5p; 200c-3p; 19b-3p; 582-5p; 497-5p; 146a-5p; 766-3p; 181c-5p; 106b-5p; 15b-5p; 130b-3p; 25-3p; 210-3p; 9-5p; 16-5p; 192-5p; 30d-5p; 381-3p; 30a-5p; 186-5p; 31-5p; 625-3p; 451a; 505-3p; 125a-5p; 195-5p; 203a-3p; 128-3p; hsa-let-7a-5p; 218-5p; 135a-5p; 423-5p; 543; 148a-3p; 30c-5p; 30b-5p; 99a-5p; 144-3p; 31-3p; 484; 130a-3p; 935; 98-5p; 149-5p; 625-5p; 495-3p; 15a-5p; 26a-5p; 106a-5p; 139-5p; 423-3p; 425-3p; 320c; 144-5p; 181d-5p; 378a-3p; 590-5p; 138-5p; 27b-3p; 27a-3p; 494-3p; 32-5p; hsa-let-7b-5p; 21-5p; 20b-5p; 454-3p; 23b-3p; 145-5p; 125b-5p; 126-3p; 92b-3p; 770-5p; 1296-5p
25	regulation of cellular process	1.487E-18	89	92a-3p; 17-5p; 182-5p; 20a-5p; 155-5p; 96-5p; 18a-5p; 1246; 19a-3p; hsa-let-7c-5p; 17-3p; 92a-1-5p; 744-5p; 93-5p; 200c-3p; 19b-3p; 582-5p; 497-5p; 146a-5p; 181c-5p; 106b-5p; 15b-5p; 130b-3p; 25-3p; 210-3p; 9-5p; 16-5p; 192-5p; 30d-5p; 381-3p; 376a-3p; 376c-3p; 124-3p; 30a-5p; 410-3p; 486-3p; 186-5p; 31-5p; 625-3p; 451a; 505-3p; 491-5p; 125a-5p; 195-5p; 203a-3p; 128-3p; hsa-let-7a-5p; 218-5p; 135a-5p; 148a-3p; 29b-1-5p; 129-1-3p; 656-3p; 30c-5p; 30b-5p; 99a-5p; 144-3p; 130a-3p; 98-5p; 149-5p; 495-3p; 15a-5p; 194-5p; 129-2-3p; 26a-5p; 106a-5p; 139-5p; 423-3p; 1-3p; 181d-5p; 378a-3p; 138-5p; 27b-3p; 204-5p; 27a-3p; 494-3p; 32-5p; 21-5p; 20b-5p; 30a-3p; 454-3p; 23b-3p; 211-5p; 145-5p; 125b-5p; 126-3p; 92b-3p; 770-5p; 1296-5p
26	programmed cell death	2.297E-18	65	92a-3p; 17-5p; 182-5p; 20a-5p; 155-5p; 96-5p; 18a-5p; 19a-3p; hsa-let-7c-5p; 183-5p; 92a-1-5p; 744-5p; 93-5p; 200c-3p; 19b-3p; 582-5p; 497-5p; 146a-5p; 181c-5p; 106b-5p; 15b-5p; 130b-3p; 210-3p; 9-5p; 16-5p; 376c-3p; 124-3p; 30a-5p; 9-3p; 31-5p; 451a; 491-5p; 125a-5p; 195-5p; 203a-3p; 128-3p; 143-3p; 218-5p; 135a-5p; 148a-3p; 30c-5p; 484; 130a-3p; 935; 98-5p; 149-5p; 495-3p; 15a-5p; 26a-5p; 106a-5p; 139-5p; 1-3p; 378a-3p; 138-5p; 127-3p; 27b-3p; 27a-3p; 494-3p; 21-5p; 20b-5p; 30a-3p; 153-3p; 145-5p; 125b-5p; 126-3p
27	cellular response to growth factor stimulus	2.34E-18	54	92a-3p; 182-5p; 155-5p; 96-5p; 18a-5p; 19a-3p; hsa-let-7c-5p; 183-5p; 744-5p; 200c-3p; 19b-3p; 497-5p; 146a-5p; 181c-5p; 15b-5p; 130b-3p; 25-3p; 9-5p; 376c-3p; 124-3p; 30a-5p; 186-5p; 451a; 125a-5p; 195-5p; 203a-3p; 128-3p; 143-3p; 218-5p; 135a-5p; 144-3p; 31-3p; 132-3p; 130a-3p; 495-3p; 15a-5p; 194-5p; 26a-5p; 296-5p; 106a-5p; 1-3p; 378a-3p; 590-5p; 138-5p; 27b-3p; 204-5p; 27a-3p; 32-5p; 21-5p; 30a-3p; 23b-3p; 145-5p; 125b-5p; 126-3p

28	cellular response to organic substance	2.34E-18	71	92a-3p; 17-5p; 182-5p; 155-5p; 96-5p; 18a-5p; 19a-3p; hsa-let-7c-5p; 183-5p; 142-3p; 744-5p; 93-5p; 200c-3p; 582-5p; 497-5p; 146a-5p; 106b-5p; 15b-5p; 130b-3p; 25-3p; 9-5p; 16-5p; 30d-5p; 381-3p; 376c-3p; 124-3p; 30a-5p; 9-3p; 31-5p; 625-3p; 451a; 125a-5p; 195-5p; 203a-3p; 369-5p; 128-3p; 143-3p; 218-5p; 135a-5p; 148a-3p; 30c-5p; 30b-5p; 144-3p; 31-3p; 132-3p; 130a-3p; 98-5p; 15a-5p; 194-5p; 26a-5p; 296-5p; 106a-5p; 320c; 1-3p; 33b-5p; 378a-3p; 7-5p; 138-5p; 27b-3p; 204-5p; 27a-3p; 494-3p; 32-5p; 21-5p; 30a-3p; 153-3p; 23b-3p; 145-5p; 125b-5p; 126-3p; 92b-3p
29	regulation of programmed cell death	2.416E-18	63	92a-3p; 17-5p; 182-5p; 20a-5p; 155-5p; 96-5p; 18a-5p; 19a-3p; hsa-let-7c-5p; 183-5p; 92a-1-5p; 744-5p; 200c-3p; 497-5p; 146a-5p; 15b-5p; 130b-3p; 210-3p; 9-5p; 16-5p; 376c-3p; 124-3p; 30a-5p; 9-3p; 625-3p; 451a; 191-5p; 125a-5p; 195-5p; 203a-3p; 128-3p; 218-5p; 135a-5p; 423-5p; 543; 148a-3p; 29b-1-5p; 30c-5p; 30b-5p; 130a-3p; 935; 98-5p; 495-3p; 15a-5p; 26a-5p; 106a-5p; 139-5p; 1-3p; 33b-5p; 378a-3p; 138-5p; 127-3p; 27b-3p; 204-5p; 27a-3p; 21-5p; 20b-5p; 30a-3p; 153-3p; 23b-3p; 145-5p; 125b-5p; 126-3p
30	regulation of apoptotic process	2.538E-18	61	92a-3p; 17-5p; 182-5p; 20a-5p; 155-5p; 96-5p; 18a-5p; 19a-3p; hsa-let-7c-5p; 183-5p; 92a-1-5p; 744-5p; 93-5p; 200c-3p; 497-5p; 146a-5p; 106b-5p; 15b-5p; 130b-3p; 210-3p; 9-5p; 16-5p; 376c-3p; 124-3p; 30a-5p; 625-3p; 451a; 125a-5p; 195-5p; 203a-3p; 128-3p; 218-5p; 135a-5p; 423-5p; 543; 148a-3p; 29b-1-5p; 30c-5p; 30b-5p; 130a-3p; 935; 98-5p; 495-3p; 15a-5p; 26a-5p; 106a-5p; 1-3p; 33b-5p; 378a-3p; 138-5p; 127-3p; 27b-3p; 204-5p; 27a-3p; 21-5p; 20b-5p; 30a-3p; 153-3p; 145-5p; 125b-5p; 126-3p
31	positive regulation of macromolecule biosynthetic process	3.004E-18	77	92a-3p; 17-5p; 182-5p; 20a-5p; 155-5p; 18a-5p; 19a-3p; hsa-let-7c-5p; 17-3p; 92a-1-5p; 744-5p; 93-5p; 200c-3p; 19b-3p; 497-5p; 146a-5p; 106b-5p; 15b-5p; 130b-3p; 25-3p; 9-5p; 16-5p; 30d-5p; 381-3p; 124-3p; 30a-5p; 410-3p; 486-3p; 186-5p; 9-3p; 31-5p; 451a; 491-5p; 125a-5p; 195-5p; 203a-3p; 128-3p; hsa-let-7a-5p; 218-5p; 135a-5p; 423-5p; 543; 148a-3p; 656-3p; 30c-5p; 30b-5p; 99a-5p; 144-3p; 484; 130a-3p; 134-5p; 98-5p; 149-5p; 495-3p; 15a-5p; 26a-5p; 296-5p; 106a-5p; 139-5p; 425-3p; 320c; 378a-3p; 138-5p; 187-3p; 27b-3p; 204-5p; 27a-3p; 494-3p; hsa-let-7b-5p; 21-5p; 20b-5p; 153-3p; 23b-3p; 145-5p; 125b-5p; 126-3p; 1296-5p
32	regulation of organelle organization	3.923E-18	54	92a-3p; 17-5p; 20a-5p; 155-5p; 18a-5p; 19a-3p; 142-3p; 744-5p; 93-5p; 200c-3p; 497-5p; 146a-5p; 106b-5p; 15b-5p; 130b-3p; 25-3p; 210-3p; 9-5p; 16-5p; 376c-3p; 30a-5p; 186-5p; 9-3p; 31-5p; 625-3p; 451a; 491-5p; 125a-5p; 195-5p; 203a-3p; 128-3p; 218-5p; 135a-5p; 148a-3p; 30c-5p; 484; 130a-3p; 149-5p; 15a-5p; 487a-3p; 26a-5p; 323a-3p; 1-3p; 138-5p; 127-3p; 27a-3p; 494-3p; hsa-let-7b-5p; 21-5p; 153-3p; 99b-3p; 145-5p; 126-3p; 92b-3p
33	apoptotic process	7.341E-18	64	92a-3p; 17-5p; 182-5p; 20a-5p; 155-5p; 96-5p; 18a-5p; 19a-3p; hsa-let-7c-5p; 183-5p; 92a-1-5p; 744-5p; 93-5p; 200c-3p; 19b-3p; 582-5p; 497-5p; 146a-5p; 181c-5p; 106b-5p; 15b-5p; 130b-3p; 210-3p; 9-5p; 16-5p; 376c-3p; 124-3p; 30a-5p; 9-3p; 31-5p; 451a; 125a-5p; 195-5p; 203a-3p; 128-3p; 143-3p; 218-5p; 135a-5p; 148a-3p; 30c-5p; 484; 130a-3p; 935; 98-5p; 149-5p; 495-3p; 15a-5p; 26a-5p; 106a-5p; 139-5p; 1-3p; 378a-3p; 138-5p; 127-3p; 27b-3p; 27a-3p; 21-5p; 20b-5p; 30a-3p; 153-3p; 23b-3p; 145-5p; 125b-5p; 126-3p

34	cell death	4.056E-17	62	92a-3p; 17-5p; 182-5p; 20a-5p; 155-5p; 96-5p; 18a-5p; 19a-3p; hsa-let-7c-5p; 183-5p; 92a-1-5p; 744-5p; 93-5p; 200c-3p; 19b-3p; 582-5p; 497-5p; 146a-5p; 181c-5p; 106b-5p; 15b-5p; 130b-3p; 210-3p; 9-5p; 16-5p; 376c-3p; 124-3p; 30a-5p; 9-3p; 31-5p; 451a; 491-5p; 125a-5p; 195-5p; 203a-3p; 128-3p; 143-3p; 218-5p; 135a-5p; 148a-3p; 30c-5p; 130a-3p; 935; 98-5p; 495-3p; 15a-5p; 26a-5p; 106a-5p; 139-5p; 1-3p; 378a-3p; 138-5p; 127-3p; 27b-3p; 27a-3p; 21-5p; 20b-5p; 30a-3p; 153-3p; 145-5p; 125b-5p; 126-3p
35	negative regulation of cell cycle	4.978E-17	58	92a-3p; 17-5p; 182-5p; 20a-5p; 155-5p; 96-5p; 18a-5p; 1246; 19a-3p; hsa-let-7c-5p; 183-5p; 142-3p; 92a-1-5p; 744-5p; 93-5p; 200c-3p; 19b-3p; 146a-5p; 106b-5p; 130b-3p; 25-3p; 16-5p; 376c-3p; 124-3p; 30a-5p; 186-5p; 451a; 191-5p; 125a-5p; 203a-3p; 128-3p; hsa-let-7a-5p; 135a-5p; 487b-3p; 148a-3p; 30c-5p; 30b-5p; 144-3p; 130a-3p; 98-5p; 149-5p; 495-3p; 15a-5p; 26a-5p; 106a-5p; 423-3p; 138-5p; 885-5p; 27b-3p; 27a-3p; 494-3p; hsa-let-7b-5p; 21-5p; 20b-5p; 23b-3p; 145-5p; 125b-5p; 126-3p
36	cellular macromolecule catabolic process	6.508E-17	52	92a-3p; 17-5p; 182-5p; 20a-5p; 155-5p; 18a-5p; hsa-let-7c-5p; 17-3p; 183-5p; hsa-let-7e-5p; 744-5p; 93-5p; 582-5p; 497-5p; 106b-5p; 15b-5p; 130b-3p; 25-3p; 16-5p; 30a-5p; 410-3p; 186-5p; 625-3p; 505-3p; 125a-5p; 195-5p; 203a-3p; 128-3p; hsa-let-7a-5p; 218-5p; 543; 148a-3p; 30c-5p; 99a-5p; 484; 935; 149-5p; 15a-5p; 376a-5p; 26a-5p; 139-5p; 423-3p; 181d-5p; 378a-3p; 27b-3p; 27a-3p; hsa-let-7b-5p; 21-5p; 20b-5p; 125b-5p; 92b-3p; 1296-5p

*p-value after the Benjamini-Hochberg adjustment
