# Supplementary material for: A Set of 17 microRNAs Common for Brain and Cerebrospinal Fluid Differentiates Primary Central Nervous System Lymphoma from Non-Malignant Brain Tumors
Source: Biomolecules. 2021 Sep 21;11(9):1395. doi: 10.3390/biom11091395 (PMC8466839; doi:10.3390/biom11091395)
Supplement: Supplementary file 1 [file biomolecules-11-01395-s001.zip › Table S13.pdf]

Supplementary Table S13

CNS DLBCL-specific FFPET microRNAs and diseases. Lymphoma and central nervous system diseases are marked bold.

No	subcategory	q-value*	number of miRs observed	hsa-miRs
		Diseases (MNDR)		
1	osteosarcoma	1.47E-85	165	92a-3p; 106b-3p; 17-5p; 182-5p; 20a-5p; 155-5p; 96-5p; 18a-5p; 19a-3p; 19b-1-5p; hsa-let-7c-5p; 17-3p; 142-5p; 183-5p; 15b-3p; hsa-let-7e-5p; 142-3p; 1260a; 744-5p; 16-2-3p; 93-5p; 200c-3p; 19b-3p; 582-5p; 130b-5p; 497-5p; 146a-5p; 766-3p; 181c-5p; 106b-5p; 15b-5p; 130b-3p; 25-3p; 210-3p; 9-5p; 486-5p; 425-5p; 16-5p; 154-5p; 192-5p; 30d-5p; 381-3p; 1185-5p; 376a-3p; 376c-3p; 628-3p; 124-3p; 30a-5p; 186-5p; 9-3p; 127-5p; 299-5p; 31-5p; 625-3p; 451a; 539-5p; 769-5p; 505-3p; 191-5p; 491-5p; 125a-5p; 181c-3p; 195-5p; 203a-3p; 379-5p; 128-3p; 143-3p; hsa-let-7a-5p; 218-5p; 135a-5p; 423-5p; 543; 485-3p; 26b-3p; 487b-3p; 148a-3p; 21-3p; 129-5p; 29b-1-5p; 129-1-3p; 30c-5p; 30b-5p; 99a-5p; 628-5p; 144-3p; 654-3p; 31-3p; 93-3p; 329-3p; 132-3p; 484; 130a-3p; 134-5p; 935; 98-5p; 149-5p; 382-3p; 409-5p; 758-3p; 495-3p; 15a-5p; 487a-3p; 95-3p; 551b-3p; 361-3p; 194-5p; 411-5p; 433-3p; 26a-5p; 296-5p; 106a-5p; 139-5p; 323a-3p; 423-3p; 425-3p; 1-3p; 132-5p; 335-3p; 144-5p; 33b-5p; 181d-5p; 18b-5p; 378a-3p; 629-5p; 590-5p; 432-5p; 7-5p; 138-5p; 127-3p; 409-3p; 339-5p; 382-5p; 885-5p; 27b-3p; 340-3p; 204-5p; 27a-3p; 383-5p; 769-3p; 494-3p; 411-3p; 376b-3p; 139-3p; 32-5p; 21-5p; 590-3p; 20b-5p; 136-5p; 340-5p; 153-3p; 99b-3p; 338-5p; 454-3p; 23b-3p; 331-5p; 28-3p; 145-5p; 370-3p; 125b-5p; 221-5p; 99b-5p; 126-3p; 92b-3p; 770-5p; 1249-3p
2	carcinoma. ductal. breast	5E-74	155	92a-3p; 17-5p; 182-5p; 20a-5p; 155-5p; 96-5p; 18a-5p; 19a-3p; 19b-1-5p; hsa-let-7c-5p; 17-3p; 142-5p; 183-5p; 15b-3p; hsa-let-7e-5p; 142-3p; 92a-1-5p; 16-2-3p; 93-5p; 200c-3p; 19b-3p; 130b-5p; 497-5p; 766-3p; 181c-5p; 106b-5p; 15b-5p; 130b-3p; 3615; 99a-3p; 210-3p; 9-5p; 486-5p; 425-5p; 16-5p; 125b-2-3p; 154-5p; 192-5p; 381-3p; 376c-3p; 30a-5p; 410-3p; 186-5p; 9-3p; 127-5p; 299-5p; 625-3p; 655-3p; 451a; 539-5p; 769-5p; 505-3p; 191-5p; 136-3p; 154-3p; 491-5p; 125a-5p; 181c-3p; 195-5p; 203a-3p; 369-5p; 379-5p; 128-3p; 143-3p; 218-5p; 543; 485-3p; 26b-3p; 487b-3p; 148a-3p; 21-3p; 129-5p; 29b-1-5p; 656-3p; 378c; 30c-5p; 30b-5p; 99a-5p; 628-5p; 144-3p; 654-3p; 101-5p; 93-3p; 329-3p; 132-3p; 484; 130a-3p; 3613-5p; 134-5p; 98-5p; 625-5p; 409-5p; 758-3p; 495-3p; 15a-5p; 376a-5p; 551b-3p; 194-5p; 411-5p; 433-3p; 26a-5p; 296-5p; 106a-5p; 139-5p; 323a-3p; 423-3p; 425-3p; 1-3p; 3065-5p; 335-3p; 144-5p; 33b-5p; 181d-5p; 378a-3p; 629-5p; 590-5p; 487a-5p; 432-5p; 7-5p; 320d; 127-3p; 532-3p; 187-3p; 409-3p; 382-5p; 340-3p; 204-5p; 27a-3p; 769-3p; 494-3p; 411-3p; 376b-3p; 139-3p; 32-5p; hsa-let-7b-5p; 21-5p; 590-3p; 20b-5p; 136-5p; 30a-3p; 340-5p; 99b-3p; 454-3p; 23b-3p; 331-5p; 28-3p; 145-5p; 370-3p; 125b-5p; 99b-5p; 126-3p; 323b-3p; 92b-3p; 1296-5p; 1249-3p
3	bladder urothelial carcinoma	5E-74	153	92a-3p; 106b-3p; 17-5p; 182-5p; 20a-5p; 96-5p; 18a-5p; 19a-3p; 19b-1-5p; hsa-let-7c-5p; 17-3p; 4521; 183-5p; 15b-3p; 142-3p; 92a-1-5p; 16-2-3p; 93-5p; 200c-3p; 19b-3p; 582-5p; 130b-5p; 497-5p; 766-3p; 181c-5p; 106b-5p; 15b-5p; 130b-3p; 25-3p; 3615; 99a-3p; 210-3p; 486-5p; 425-5p; 16-5p; 125b-2-3p; 154-5p; 192-5p; 30d-5p; 381-3p; 376a-3p; 376c-3p; 30a-5p; 410-3p; 127-5p; 299-5p; 31-5p; 655-3p; 451a; 539-5p; 505-3p; 191-5p; 136-3p; 154-3p; 125a-5p; 181c-3p; 195-5p; 203a-3p; 369-5p; 379-5p; 143-3p; hsa-let-7a-5p; 218-5p; 423-5p; 543; 485-3p; 26b-3p; 487b-3p; 148a-3p; 21-3p; 129-5p; 656-3p; 378c; 30c-5p; 30b-5p; 99a-5p; 628-5p; 144-3p; 654-3p; 31-3p; 93-3p; 329-3p; 132-3p; 484; 130a-3p; 3613-5p; 134-5p; 98-5p; 149-5p; 409-5p; 758-3p; 495-3p; 15a-5p; 376a-5p; 487a-3p; 551b-3p; 194-5p; 411-5p; 433-3p; 26a-5p; 106a-5p; 139-5p; 423-3p; 425-3p; 320c; 1-3p; 132-5p; 3065-5p; 335-3p; 144-5p; 378a-3p; 629-5p; 590-5p; 487a-5p; 432-5p; 138-5p; 127-3p; 532-3p; 187-3p; 409-3p; 339-5p; 382-5p; 27b-3p; 340-3p; 204-5p; 27a-3p; 769-3p; 494-3p; 369-3p; 411-3p; 502-3p; 376b-3p; 139-3p; 32-5p; 21-5p; 590-3p; 136-5p; 30a-3p; 340-5p; 378d; 338-5p; 454-3p; 23b-3p; 331-5p; 28-3p; 145-5p; 370-3p; 504-5p; 125b-5p; 221-5p; 126-3p; 92b-3p; 1249-3p

4	renal clear cell carcinoma	5E-74	158	92a-3p; 106b-3p; 17-5p; 182-5p; 20a-5p; 155-5p; 96-5p; 18a-5p; 19a-3p; 19b-1-5p; hsa-let-7c-5p; 17-3p; 142-5p; 183-5p; 15b-3p; hsa-let-7e-5p; 142-3p; 744-5p; 16-2-3p; 93-5p; 200c-3p; 19b-3p; 130b-5p; 497-5p; 146a-5p; 766-3p; 181c-5p; 106b-5p; 15b-5p; 130b-3p; 25-3p; 3615; 99a-3p; 210-3p; 9-5p; 486-5p; 425-5p; 16-5p; 154-5p; 192-5p; 30d-5p; 381-3p; 376c-3p; 124-3p; 30a-5p; 486-3p; 186-5p; 127-5p; 31-5p; 625-3p; 451a; 539-5p; 769-5p; 505-3p; 191-5p; 136-3p; 125a-5p; 181c-3p; 195-5p; 203a-3p; 369-5p; 379-5p; 128-3p; 143-3p; hsa-let-7a-5p; 218-5p; 135a-5p; 485-3p; 26b-3p; 487b-3p; 148a-3p; 21-3p; 129-5p; 29b-1-5p; 378c; 30c-5p; 1248; 30b-5p; 99a-5p; 628-5p; 144-3p; 654-3p; 101-5p; 93-3p; 484; 130a-3p; 3613-5p; 134-5p; 935; 149-5p; 625-5p; 409-5p; 758-3p; 495-3p; 15a-5p; 95-3p; 551b-3p; 361-3p; 194-5p; 411-5p; 26a-5p; 296-5p; 106a-5p; 139-5p; 323a-3p; 423-3p; 320c; 1-3p; 132-5p; 3065-5p; 335-3p; 144-5p; 33b-5p; 181d-5p; 378a-3p; 629-5p; 590-5p; 320d; 138-5p; 127-3p; 532-3p; 187-3p; 339-5p; 382-5p; 885-5p; 27b-3p; 340-3p; 204-5p; 27a-3p; 494-3p; 502-3p; 139-3p; 32-5p; hsa-let-7b-5p; 21-5p; 590-3p; 20b-5p; 136-5p; 30a-3p; 340-5p; 378d; 153-3p; 99b-3p; 338-5p; 454-3p; 23b-3p; 331-5p; 28-3p; 145-5p; 370-3p; 125b-5p; 221-5p; 99b-5p; 126-3p; 323b-3p; 92b-3p; 1296-5p; 1249-3p
5	lung cancer	2.74E-73	184	92a-3p; 106b-3p; 17-5p; 182-5p; 20a-5p; 155-5p; 96-5p; 18a-5p; 1246; 19a-3p; hsa-let-7c-5p; 17-3p; 1290; 142-5p; 183-5p; hsa-let-7e-5p; 142-3p; 92a-1-5p; 744-5p; 873-5p; 93-5p; 200c-3p; 19b-3p; 582-5p; 130b-5p; 497-5p; 146a-5p; 766-3p; 181c-5p; 106b-5p; 15b-5p; 130b-3p; 25-3p; 99a-3p; 210-3p; 9-5p; 486-5p; 425-5p; 16-5p; 125b-2-3p; 154-5p; 192-5p; 30d-5p; 381-3p; 376a-3p; 376c-3p; 628-3p; 1285-3p; 124-3p; 30a-5p; 486-3p; 186-5p; 9-3p; 127-5p; 299-5p; 31-5p; 625-3p; 655-3p; 451a; 539-5p; 769-5p; 505-3p; 191-5p; 154-3p; 491-5p; 125a-5p; 181c-3p; 195-5p; 203a-3p; 369-5p; 379-5p; 128-3p; 485-5p; 143-3p; hsa-let-7a-5p; 218-5p; 135a-5p; 423-5p; 543; 485-3p; 26b-3p; 487b-3p; 148a-3p; 129-5p; 29b-1-5p; 129-1-3p; 30c-5p; 30b-5p; 99a-5p; 380-3p; 628-5p; 144-3p; 654-3p; 101-5p; 31-3p; 93-3p; 132-3p; 484; 130a-3p; 134-5p; 935; 98-5p; 149-5p; 625-5p; 409-5p; 758-3p; 495-3p; 15a-5p; 376a-5p; 487a-3p; 95-3p; 551b-3p; 361-3p; 194-5p; 411-5p; 433-3p; 129-2-3p; 26a-5p; 296-5p; 106a-5p; 139-5p; 323a-3p; 423-3p; 425-3p; 1-3p; 132-5p; 144-5p; 33b-5p; 181d-5p; 18b-5p; 378a-3p; 629-5p; 590-5p; 487a-5p; 432-5p; 7-5p; 320d; 138-5p; 127-3p; 532-3p; 187-3p; 409-3p; 339-5p; 382-5p; 885-5p; 27b-3p; 340-3p; 204-5p; 27a-3p; 383-5p; 769-3p; 494-3p; 369-3p; 502-3p; 376b-3p; 139-3p; 32-5p; hsa-let-7b-5p; 21-5p; 590-3p; 20b-5p; 136-5p; 30a-3p; 340-5p; 153-3p; 99b-3p; 338-5p; 454-3p; 23b-3p; 331-5p; 28-3p; 211-5p; 145-5p; 370-3p; 504-5p; 125b-5p; 92b-5p; 221-5p; 99b-5p; 126-3p; 92b-3p; 770-5p; 1296-5p; 1249-3p
6	prostate adenocarcinoma	6.09E-73	145	92a-3p; 17-5p; 182-5p; 20a-5p; 96-5p; 18a-5p; 19a-3p; 19b-1-5p; hsa-let-7c-5p; 17-3p; 183-5p; 15b-3p; hsa-let-7e-5p; 92a-1-5p; 16-2-3p; 93-5p; 200c-3p; 19b-3p; 582-5p; 130b-5p; 766-3p; 181c-5p; 106b-5p; 15b-5p; 130b-3p; 25-3p; 99a-3p; 210-3p; 9-5p; 486-5p; 425-5p; 125b-2-3p; 154-5p; 192-5p; 30d-5p; 381-3p; 376c-3p; 30a-5p; 410-3p; 186-5p; 9-3p; 127-5p; 299-5p; 625-3p; 655-3p; 451a; 539-5p; 505-3p; 191-5p; 136-3p; 154-3p; 491-5p; 125a-5p; 181c-3p; 203a-3p; 379-5p; 128-3p; 143-3p; hsa-let-7a-5p; 218-5p; 135a-5p; 423-5p; 487b-3p; 148a-3p; 21-3p; 129-5p; 29b-1-5p; 378c; 30c-5p; 30b-5p; 99a-5p; 628-5p; 144-3p; 654-3p; 101-5p; 31-3p; 93-3p; 132-3p; 484; 130a-3p; 3613-5p; 134-5p; 98-5p; 149-5p; 758-3p; 495-3p; 15a-5p; 95-3p; 194-5p; 411-5p; 26a-5p; 296-5p; 106a-5p; 139-5p; 323a-3p; 423-3p; 425-3p; 1-3p; 3065-5p; 144-5p; 33b-5p; 181d-5p; 18b-5p; 378a-3p; 629-5p; 590-5p; 432-5p; 7-5p; 320d; 127-3p; 532-3p; 187-3p; 409-3p; 339-5p; 27b-3p; 340-3p; 204-5p; 27a-3p; 383-5p; 769-3p; 494-3p; 369-3p; 411-3p; 139-3p; 32-5p; hsa-let-7b-5p; 21-5p; 20b-5p; 136-5p; 30a-3p; 340-5p; 378d; 153-3p; 99b-3p; 338-5p; 454-3p; 23b-3p; 145-5p; 370-3p; 221-5p; 99b-5p; 126-3p; 323b-3p; 1296-5p; 1249-3p

7	pancreatic adenocarcinoma	6.81E-71	158	92a-3p; 106b-3p; 17-5p; 182-5p; 20a-5p; 155-5p; 18a-5p; 1246; 19b-1-5p; hsa-let-7c-5p; 17-3p; 142-5p; 183-5p; 15b-3p; hsa-let-7e-5p; 142-3p; 1260b; 92a-1-5p; 744-5p; 16-2-3p; 93-5p; 130b-5p; 497-5p; 146a-5p; 766-3p; 106b-5p; 15b-5p; 130b-3p; 25-3p; 210-3p; 486-5p; 425-5p; 16-5p; 125b-2-3p; 154-5p; 192-5p; 30d-5p; 1185-5p; 376a-3p; 376c-3p; 628-3p; 1285-3p; 30a-5p; 410-3p; 486-3p; 186-5p; 127-5p; 299-5p; 31-5p; 625-3p; 655-3p; 451a; 539-5p; 769-5p; 505-3p; 191-5p; 136-3p; 154-3p; 125a-5p; 181c-3p; 203a-3p; 369-5p; 379-5p; 128-3p; 485-5p; 143-3p; 135a-5p; 423-5p; 543; 485-3p; 26b-3p; 487b-3p; 148a-3p; 21-3p; 129-5p; 29b-1-5p; 129-1-3p; 656-3p; 378c; 30b-5p; 99a-5p; 380-3p; 628-5p; 144-3p; 654-3p; 101-5p; 31-3p; 93-3p; 329-3p; 132-3p; 484; 134-5p; 935; 98-5p; 382-3p; 409-5p; 495-3p; 15a-5p; 376a-5p; 487a-3p; 95-3p; 551b-3p; 194-5p; 411-5p; 433-3p; 129-2-3p; 106a-5p; 139-5p; 323a-3p; 423-3p; 132-5p; 3065-5p; 335-3p; 144-5p; 181d-5p; 18b-5p; 378a-3p; 629-5p; 590-5p; 487a-5p; 432-5p; 7-5p; 320d; 138-5p; 127-3p; 532-3p; 187-3p; 409-3p; 339-5p; 382-5p; 204-5p; 769-3p; 494-3p; 369-3p; 411-3p; 502-3p; 139-3p; hsa-let-7b-5p; 21-5p; 590-3p; 136-5p; 30a-3p; 153-3p; 99b-3p; 338-5p; 454-3p; 23b-3p; 331-5p; 28-3p; 145-5p; 370-3p; 125b-5p; 221-5p; 99b-5p; 126-3p; 323b-3p; 92b-3p; 770-5p
8	papillary renal cell carcinoma	7.51E-71	156	92a-3p; 106b-3p; 17-5p; 182-5p; 20a-5p; 155-5p; 96-5p; 18a-5p; 19a-3p; 19b-1-5p; hsa-let-7c-5p; 17-3p; 142-5p; 183-5p; 15b-3p; hsa-let-7e-5p; 142-3p; 744-5p; 16-2-3p; 93-5p; 200c-3p; 19b-3p; 130b-5p; 497-5p; 146a-5p; 766-3p; 181c-5p; 106b-5p; 15b-5p; 130b-3p; 25-3p; 3615; 99a-3p; 210-3p; 9-5p; 486-5p; 425-5p; 16-5p; 125b-2-3p; 154-5p; 192-5p; 30d-5p; 381-3p; 376c-3p; 628-3p; 30a-5p; 410-3p; 486-3p; 186-5p; 127-5p; 299-5p; 31-5p; 625-3p; 451a; 539-5p; 769-5p; 505-3p; 191-5p; 136-3p; 491-5p; 125a-5p; 181c-3p; 195-5p; 203a-3p; 369-5p; 379-5p; 128-3p; 143-3p; 218-5p; 135a-5p; 423-5p; 485-3p; 487b-3p; 148a-3p; 21-3p; 129-5p; 29b-1-5p; 30b-5p; 99a-5p; 628-5p; 144-3p; 654-3p; 101-5p; 31-3p; 93-3p; 132-3p; 484; 3613-5p; 134-5p; 935; 98-5p; 625-5p; 409-5p; 758-3p; 495-3p; 15a-5p; 551b-3p; 361-3p; 194-5p; 411-5p; 26a-5p; 296-5p; 106a-5p; 139-5p; 323a-3p; 423-3p; 425-3p; 320c; 1-3p; 132-5p; 3065-5p; 144-5p; 33b-5p; 181d-5p; 629-5p; 590-5p; 432-5p; 127-3p; 532-3p; 187-3p; 409-3p; 339-5p; 382-5p; 885-5p; 27b-3p; 340-3p; 204-5p; 27a-3p; 769-3p; 494-3p; 369-3p; 502-3p; 139-3p; hsa-let-7b-5p; 21-5p; 590-3p; 20b-5p; 136-5p; 340-5p; 378d; 153-3p; 99b-3p; 338-5p; 454-3p; 23b-3p; 331-5p; 28-3p; 211-5p; 145-5p; 370-3p; 125b-5p; 126-3p; 323b-3p; 92b-3p; 1296-5p; 1249-3p
9	glioblastoma	7.36E-70	189	92a-3p; 106b-3p; 17-5p; 182-5p; 20a-5p; 155-5p; 96-5p; 18a-5p; 1246; 19a-3p; 19b-1-5p; hsa-let-7c-5p; 17-3p; 1290; 142-5p; 183-5p; 15b-3p; hsa-let-7e-5p; 142-3p; 1260a; 92a-1-5p; 744-5p; 873-5p; 93-5p; 200c-3p; 19b-3p; 582-5p; 130b-5p; 497-5p; 146a-5p; 181c-5p; 106b-5p; 15b-5p; 130b-3p; 25-3p; 99a-3p; 210-3p; 9-5p; 486-5p; 425-5p; 16-5p; 125b-2-3p; 154-5p; 192-5p; 30d-5p; 381-3p; 1185-5p; 376a-3p; 376c-3p; 628-3p; 124-3p; 30a-5p; 410-3p; 486-3p; 186-5p; 9-3p; 299-5p; 31-5p; 625-3p; 655-3p; 451a; 539-5p; 505-3p; 191-5p; 154-3p; 491-5p; 125a-5p; 181c-3p; 195-5p; 203a-3p; 369-5p; 379-5p; 128-3p; 485-5p; 143-3p; hsa-let-7a-5p; 218-5p; 4286; 135a-5p; 423-5p; 543; 485-3p; 26b-3p; 487b-3p; 148a-3p; 21-3p; 129-5p; 129-1-3p; 656-3p; 30c-5p; 1248; 30b-5p; 99a-5p; 380-3p; 628-5p; 144-3p; 654-3p; 93-3p; 329-3p; 132-3p; 484; 130a-3p; 134-5p; 935; 98-5p; 149-5p; 382-3p; 625-5p; 378i; 409-5p; 495-3p; 15a-5p; 376a-5p; 487a-3p; 95-3p; 551b-3p; 361-3p; 194-5p; 411-5p; 433-3p; 129-2-3p; 26a-5p; 296-5p; 106a-5p; 139-5p; 323a-3p; 423-3p; 425-3p; 320c; 1-3p; 132-5p; 144-5p; 181d-5p; 18b-5p; 378a-3p; 629-5p; 432-5p; 7-5p; 124-5p; 320d; 138-5p; 127-3p; 532-3p; 187-3p; 409-3p; 339-5p; 382-5p; 885-5p; 27b-3p; 340-3p; 204-5p; 27a-3p; 383-5p; 769-3p; 494-3p; 369-3p; 411-3p; 502-3p; 376b-3p; 139-3p; 32-5p; hsa-let-7b-5p; 21-5p; 590-3p; 20b-5p; 136-5p; 30a-3p; 340-5p; 378d; 153-3p; 99b-3p; 338-5p; 454-3p; 23b-3p; 28-3p; 211-5p; 145-5p; 370-3p; 504-5p; 125b-5p; 92b-5p; 221-5p; 99b-5p; 126-3p; 323b-3p; 92b-3p; 770-5p; 1296-5p; 1249-3p

10	head and neck squamous cell carcinoma	2.41E-68	153	92a-3p; 106b-3p; 17-5p; 182-5p; 20a-5p; 155-5p; 96-5p; 18a-5p; 19a-3p; 19b-1-5p; hsa-let-7c-5p; 4521; 183-5p; 15b-3p; hsa-let-7e-5p; 142-3p; 92a-1-5p; 16-2-3p; 93-5p; 200c-3p; 19b-3p; 130b-5p; 497-5p; 766-3p; 181c-5p; 106b-5p; 15b-5p; 4449; 130b-3p; 25-3p; 3615; 99a-3p; 210-3p; 9-5p; 486-5p; 425-5p; 16-5p; 125b-2-3p; 154-5p; 381-3p; 376a-3p; 376c-3p; 628-3p; 30a-5p; 410-3p; 486-3p; 186-5p; 127-5p; 299-5p; 31-5p; 655-3p; 451a; 539-5p; 769-5p; 505-3p; 191-5p; 136-3p; 154-3p; 491-5p; 125a-5p; 195-5p; 203a-3p; 369-5p; 379-5p; 128-3p; 143-3p; 218-5p; 423-5p; 543; 26b-3p; 487b-3p; 148a-3p; 21-3p; 29b-1-5p; 656-3p; 378c; 30c-5p; 30b-5p; 99a-5p; 144-3p; 654-3p; 101-5p; 31-3p; 93-3p; 329-3p; 132-3p; 484; 130a-3p; 3613-5p; 134-5p; 98-5p; 149-5p; 382-3p; 758-3p; 495-3p; 15a-5p; 376a-5p; 487a-3p; 95-3p; 361-3p; 411-5p; 433-3p; 26a-5p; 296-5p; 106a-5p; 139-5p; 423-3p; 1-3p; 144-5p; 378a-3p; 590-5p; 487a-5p; 432-5p; 7-5p; 320d; 138-5p; 127-3p; 532-3p; 187-3p; 409-3p; 339-5p; 382-5p; 27b-3p; 204-5p; 27a-3p; 769-3p; 494-3p; 369-3p; 411-3p; 502-3p; 376b-3p; 139-3p; 32-5p; 21-5p; 136-5p; 30a-3p; 340-5p; 378d; 153-3p; 99b-3p; 338-5p; 454-3p; 23b-3p; 331-5p; 28-3p; 145-5p; 370-3p; 125b-5p; 221-5p; 99b-5p; 126-3p; 323b-3p; 92b-3p
11	breast neoplasms	1.78E-65	181	92a-3p; 106b-3p; 17-5p; 182-5p; 20a-5p; 155-5p; 96-5p; 18a-5p; 1246; 19a-3p; 19b-1-5p; hsa-let-7c-5p; 17-3p; 1290; 142-5p; 183-5p; 15b-3p; hsa-let-7e-5p; 142-3p; 1260a; 92a-1-5p; 744-5p; 16-2-3p; 873-5p; 93-5p; 200c-3p; 19b-3p; 582-5p; 497-5p; 146a-5p; 766-3p; 181c-5p; 106b-5p; 15b-5p; 130b-3p; 25-3p; 99a-3p; 210-3p; 9-5p; 486-5p; 425-5p; 16-5p; 125b-2-3p; 154-5p; 192-5p; 30d-5p; 381-3p; 1185-5p; 376a-3p; 376c-3p; 628-3p; 124-3p; 30a-5p; 410-3p; 486-3p; 9-3p; 127-5p; 299-5p; 31-5p; 625-3p; 655-3p; 451a; 539-5p; 769-5p; 505-3p; 191-5p; 136-3p; 154-3p; 125a-5p; 181c-3p; 195-5p; 203a-3p; 369-5p; 379-5p; 128-3p; 143-3p; hsa-let-7a-5p; 218-5p; 135a-5p; 423-5p; 543; 485-3p; 26b-3p; 487b-3p; 148a-3p; 21-3p; 129-5p; 29b-1-5p; 656-3p; 30c-5p; 1248; 30b-5p; 99a-5p; 380-3p; 144-3p; 654-3p; 101-5p; 31-3p; 93-3p; 329-3p; 132-3p; 484; 130a-3p; 134-5p; 935; 98-5p; 149-5p; 625-5p; 409-5p; 758-3p; 495-3p; 15a-5p; 376a-5p; 487a-3p; 95-3p; 551b-3p; 361-3p; 194-5p; 411-5p; 129-2-3p; 26a-5p; 296-5p; 106a-5p; 139-5p; 323a-3p; 423-3p; 425-3p; 320c; 1-3p; 132-5p; 335-3p; 144-5p; 33b-5p; 181d-5p; 18b-5p; 378a-3p; 629-5p; 590-5p; 432-5p; 7-5p; 124-5p; 320d; 138-5p; 127-3p; 532-3p; 187-3p; 409-3p; 339-5p; 382-5p; 27b-3p; 204-5p; 27a-3p; 383-5p; 769-3p; 494-3p; 502-3p; 139-3p; 32-5p; hsa-let-7b-5p; 21-5p; 590-3p; 20b-5p; 136-5p; 30a-3p; 340-5p; 153-3p; 99b-3p; 338-5p; 454-3p; 331-5p; 28-3p; 145-5p; 370-3p; 504-5p; 125b-5p; 92b-5p; 221-5p; 99b-5p; 126-3p; 92b-3p; 770-5p
12	thyroid cancer	5.73E-65	111	92a-3p; 106b-3p; 17-5p; 182-5p; 20a-5p; 155-5p; 96-5p; 18a-5p; 19a-3p; 19b-1-5p; 142-5p; 183-5p; 15b-3p; hsa-let-7e-5p; 142-3p; 16-2-3p; 93-5p; 200c-3p; 19b-3p; 497-5p; 146a-5p; 181c-5p; 15b-5p; 25-3p; 210-3p; 9-5p; 486-5p; 16-5p; 154-5p; 30d-5p; 381-3p; 376a-3p; 376c-3p; 124-3p; 30a-5p; 410-3p; 9-3p; 299-5p; 31-5p; 451a; 539-5p; 191-5p; 136-3p; 154-3p; 125a-5p; 195-5p; 203a-3p; 369-5p; 379-5p; 485-5p; 143-3p; hsa-let-7a-5p; 218-5p; 543; 485-3p; 487b-3p; 148a-3p; 129-5p; 129-1-3p; 656-3p; 144-3p; 654-3p; 101-5p; 31-3p; 329-3p; 132-3p; 130a-3p; 149-5p; 625-5p; 409-5p; 758-3p; 495-3p; 15a-5p; 376a-5p; 487a-3p; 551b-3p; 411-5p; 433-3p; 129-2-3p; 26a-5p; 106a-5p; 323a-3p; 132-5p; 335-3p; 144-5p; 18b-5p; 629-5p; 432-5p; 7-5p; 138-5p; 127-3p; 409-3p; 339-5p; 382-5p; 27b-3p; 204-5p; 27a-3p; 369-3p; 376b-3p; hsa-let-7b-5p; 21-5p; 20b-5p; 136-5p; 30a-3p; 153-3p; 23b-3p; 145-5p; 125b-5p; 92b-5p; 99b-5p; 126-3p
13	uterine corpus endometrial carcinoma	1.35E-64	146	92a-3p; 106b-3p; 17-5p; 182-5p; 155-5p; 96-5p; 18a-5p; 19a-3p; hsa-let-7c-5p; 142-5p; 183-5p; 15b-3p; 142-3p; 744-5p; 16-2-3p; 93-5p; 200c-3p; 19b-3p; 582-5p; 130b-5p; 497-5p; 146a-5p; 766-3p; 181c-5p; 106b-5p; 15b-5p; 4449; 130b-3p; 25-3p; 3615; 99a-3p; 210-3p; 9-5p; 425-5p; 16-5p; 125b-2-3p; 154-5p; 192-5p; 381-3p; 376c-3p; 410-3p; 486-3p; 9-3p; 127-5p; 299-5p; 31-5p; 655-3p; 451a; 539-5p; 769-5p; 505-3p; 191-5p; 136-3p; 154-3p; 125a-5p; 181c-3p; 195-5p; 203a-3p; 369-5p; 379-5p; 128-3p; 143-3p; hsa-let-7a-5p; 218-5p; 485-3p; 26b-3p; 487b-3p; 21-3p; 129-5p; 29b-1-5p; 378c; 30b-5p; 99a-5p; 628-5p; 654-3p; 101-5p; 31-3p; 93-3p; 132-3p; 484; 130a-3p; 3613-5p; 134-5p; 382-3p; 409-5p; 758-3p; 495-3p; 15a-5p; 95-3p; 551b-3p; 361-3p; 194-5p; 411-5p; 433-3p; 26a-5p; 296-5p; 106a-5p; 139-5p; 423-3p; 425-3p; 1-3p; 132-5p; 3065-5p; 144-5p; 33b-5p; 18b-5p; 378a-3p; 629-5p; 590-5p; 432-5p; 7-5p; 127-3p; 187-3p; 409-3p; 339-5p; 382-5p; 885-5p; 27b-3p; 340-3p; 204-5p; 769-3p; 494-3p; 369-3p; 411-3p; 502-3p; 376b-3p; 139-3p; 32-5p; hsa-let-7b-5p; 21-5p; 590-3p; 20b-5p; 136-5p; 153-3p; 99b-3p; 454-3p; 23b-3p;

				28-3p; 145-5p; 370-3p; 125b-5p; 92b-5p; 221-5p; 126-3p; 92b-3p; 1296-5p
14	Alzheimers disease	1.73E-64	153	92a-3p; 17-5p; 182-5p; 20a-5p; 155-5p; 96-5p; 18a-5p; 19a-3p; 17-3p; 142-5p; 183-5p; hsa-let-7e-5p; 142-3p; 1260a; 744-5p; 16-2-3p; 873-5p; 93-5p; 200c-3p; 19b-3p; 582-5p; 497-5p; 146a-5p; 766-3p; 181c-5p; 106b-5p; 15b-5p; 4449; 130b-3p; 25-3p; 210-3p; 9-5p; 486-5p; 425-5p; 16-5p; 154-5p; 192-5p; 30d-5p; 381-3p; 376a-3p; 376c-3p; 628-3p; 1285-3p; 124-3p; 30a-5p; 410-3p; 486-3p; 186-5p; 9-3p; 127-5p; 299-5p; 31-5p; 451a; 769-5p; 505-3p; 191-5p; 136-3p; 491-5p; 125a-5p; 195-5p; 203a-3p; 379-5p; 128-3p; 485-5p; 143-3p; hsa-let-7a-5p; 218-5p; 135a-5p; 423-5p; 543; 485-3p; 26b-3p; 487b-3p; 148a-3p; 129-5p; 30c-5p; 30b-5p; 99a-5p; 380-3p; 144-3p; 101-5p; 329-3p; 132-3p; 130a-3p; 134-5p; 98-5p; 149-5p; 409-5p; 758-3p; 495-3p; 15a-5p; 487a-3p; 95-3p; 551b-3p; 361-3p; 194-5p; 411-5p; 433-3p; 129-2-3p; 26a-5p; 296-5p; 106a-5p; 139-5p; 323a-3p; 423-3p; 425-3p; 320c; 1-3p; 132-5p; 144-5p; 33b-5p; 181d-5p; 18b-5p; 378a-3p; 629-5p; 590-5p; 487a-5p; 432-5p; 7-5p; 320d; 138-5p; 127-3p; 187-3p; 339-5p; 382-5p; 885-5p; 27b-3p; 204-5p; 27a-3p; 383-5p; 494-3p; 369-3p; 502-3p; 32-5p; 21-5p; 590-3p; 20b-5p; 136-5p; 30a-3p; 340-5p; 153-3p; 338-5p; 454-3p; 23b-3p; 331-5p; 28-3p; 211-5p; 145-5p; 370-3p; 125b-5p; 99b-5p; 126-3p; 92b-3p
15	cervical squamous cell carcinoma	5.27E-63	107	92a-3p; 106b-3p; 17-5p; 182-5p; 96-5p; 18a-5p; 19a-3p; hsa-let-7c-5p; 17-3p; 142-5p; 183-5p; 15b-3p; 142-3p; 16-2-3p; 93-5p; 200c-3p; 19b-3p; 130b-5p; 497-5p; 146a-5p; 181c-5p; 106b-5p; 15b-5p; 130b-3p; 25-3p; 99a-3p; 210-3p; 9-5p; 486-5p; 425-5p; 16-5p; 125b-2-3p; 154-5p; 30d-5p; 381-3p; 376a-3p; 376c-3p; 124-3p; 410-3p; 186-5p; 299-5p; 31-5p; 451a; 539-5p; 136-3p; 125a-5p; 195-5p; 203a-3p; 379-5p; 143-3p; hsa-let-7a-5p; 218-5p; 423-5p; 543; 487b-3p; 148a-3p; 21-3p; 129-5p; 656-3p; 30c-5p; 30b-5p; 99a-5p; 654-3p; 101-5p; 93-3p; 132-3p; 484; 3613-5p; 134-5p; 149-5p; 409-5p; 758-3p; 495-3p; 15a-5p; 411-5p; 433-3p; 26a-5p; 296-5p; 106a-5p; 139-5p; 320c; 1-3p; 33b-5p; 181d-5p; 378a-3p; 629-5p; 487a-5p; 432-5p; 127-3p; 339-5p; 204-5p; 27a-3p; 494-3p; 411-3p; 502-3p; 376b-3p; 139-3p; 32-5p; 21-5p; 20b-5p; 23b-3p; 28-3p; 145-5p; 370-3p; 125b-5p; 92b-3p; 1296-5p
16	hepatocellular carcinoma	7.44E-63	191	92a-3p; 106b-3p; 17-5p; 182-5p; 20a-5p; 155-5p; 96-5p; 18a-5p; 1246; 19a-3p; 19b-1-5p; hsa-let-7c-5p; 17-3p; 1290; 142-5p; 183-5p; 15b-3p; hsa-let-7e-5p; 142-3p; 1260a; 92a-1-5p; 744-5p; 16-2-3p; 93-5p; 200c-3p; 19b-3p; 582-5p; 130b-5p; 497-5p; 146a-5p; 766-3p; 181c-5p; 106b-5p; 15b-5p; 130b-3p; 25-3p; 3615; 99a-3p; 210-3p; 9-5p; 486-5p; 425-5p; 16-5p; 125b-2-3p; 154-5p; 192-5p; 30d-5p; 381-3p; 376a-3p; 376c-3p; 628-3p; 124-3p; 30a-5p; 410-3p; 486-3p; 186-5p; 9-3p; 127-5p; 299-5p; 31-5p; 625-3p; 655-3p; 451a; 539-5p; 769-5p; 505-3p; 191-5p; 136-3p; 154-3p; 491-5p; 125a-5p; 195-5p; 203a-3p; 369-5p; 379-5p; 128-3p; 485-5p; 143-3p; hsa-let-7a-5p; 218-5p; 135a-5p; 423-5p; 543; 485-3p; 26b-3p; 487b-3p; 148a-3p; 21-3p; 129-5p; 29b-1-5p; 378c; 30c-5p; 1248; 30b-5p; 99a-5p; 144-3p; 654-3p; 101-5p; 93-3p; 329-3p; 132-3p; 484; 130a-3p; 3613-5p; 134-5p; 935; 98-5p; 149-5p; 625-5p; 409-5p; 758-3p; 495-3p; 15a-5p; 376a-5p; 487a-3p; 95-3p; 551b-3p; 361-3p; 194-5p; 411-5p; 433-3p; 129-2-3p; 26a-5p; 296-5p; 106a-5p; 139-5p; 323a-3p; 423-3p; 425-3p; 320c; 1-3p; 132-5p; 335-3p; 144-5p; 33b-5p; 181d-5p; 18b-5p; 378a-3p; 629-5p; 590-5p; 432-5p; 7-5p; 138-5p; 127-3p; 532-3p; 187-3p; 409-3p; 339-5p; 382-5p; 885-5p; 27b-3p; 340-3p; 204-5p; 27a-3p; 383-5p; 769-3p; 494-3p; 369-3p; 411-3p; 502-3p; 376b-3p; 139-3p; 32-5p; hsa-let-7b-5p; 21-5p; 590-3p; 20b-5p; 136-5p; 30a-3p; 340-5p; 378d; 153-3p; 99b-3p; 338-5p; 454-3p; 23b-3p; 331-5p; 28-3p; 211-5p; 145-5p; 370-3p; 504-5p; 125b-

				5p; 92b-5p; 221-5p; 99b-5p; 126-3p; 92b-3p; 770-5p; 1296-5p; 1249-3p
17	gastric adenocarcinoma	6.09E-61	139	92a-3p; 106b-3p; 17-5p; 182-5p; 20a-5p; 155-5p; 96-5p; 18a-5p; 19a-3p; 19b-1-5p; hsa-let-7c-5p; 17-3p; 142-5p; 183-5p; 15b-3p; hsa-let-7e-5p; 142-3p; 92a-1-5p; 744-5p; 16-2-3p; 93-5p; 200c-3p; 19b-3p; 582-5p; 130b-5p; 497-5p; 146a-5p; 766-3p; 181c-5p; 106b-5p; 15b-5p; 130b-3p; 25-3p; 99a-3p; 210-3p; 9-5p; 486-5p; 425-5p; 16-5p; 125b-2-3p; 154-5p; 192-5p; 30d-5p; 381-3p; 376c-3p; 628-3p; 30a-5p; 410-3p; 486-3p; 186-5p; 9-3p; 127-5p; 299-5p; 655-3p; 451a; 539-5p; 191-5p; 136-3p; 154-3p; 491-5p; 125a-5p; 195-5p; 203a-3p; 369-5p; 379-5p; 143-3p; hsa-let-7a-5p; 218-5p; 135a-5p; 543; 485-3p; 487b-3p; 21-3p; 129-5p; 29b-1-5p; 378c; 30c-5p; 30b-5p; 99a-5p; 628-5p; 144-3p; 654-3p; 101-5p; 93-3p; 132-3p; 484; 130a-3p; 3613-5p; 935; 149-5p; 382-3p; 758-3p; 495-3p; 15a-5p; 376a-5p; 95-3p; 551b-3p; 194-5p; 411-5p; 433-3p; 26a-5p; 106a-5p; 139-5p; 423-3p; 1-3p; 132-5p; 335-3p; 144-5p; 378a-3p; 590-5p; 432-5p; 7-5p; 138-5p; 127-3p; 532-3p; 187-3p; 382-5p; 27b-3p; 340-3p; 204-5p; 411-3p; 502-3p; 376b-3p; 139-3p; hsa-let-7b-5p; 21-5p; 590-3p; 20b-5p; 136-5p; 30a-3p; 378d; 153-3p; 338-5p; 23b-3p; 28-3p; 145-5p; 125b-5p; 221-5p; 1296-5p
18	lung adenocarcinoma	1.04E-60	175	106b-3p; 17-5p; 182-5p; 20a-5p; 155-5p; 96-5p; 18a-5p; 1246; 19a-3p; 19b-1-5p; hsa-let-7c-5p; 17-3p; 4521; 1290; 142-5p; 183-5p; hsa-let-7e-5p; 142-3p; 1260a; 744-5p; 16-2-3p; 93-5p; 200c-3p; 19b-3p; 582-5p; 130b-5p; 497-5p; 146a-5p; 181c-5p; 106b-5p; 15b-5p; 130b-3p; 25-3p; 3615; 99a-3p; 210-3p; 9-5p; 486-5p; 425-5p; 16-5p; 125b-2-3p; 154-5p; 192-5p; 30d-5p; 381-3p; 376c-3p; 628-3p; 1285-3p; 124-3p; 30a-5p; 410-3p; 486-3p; 186-5p; 9-3p; 127-5p; 31-5p; 625-3p; 655-3p; 451a; 539-5p; 769-5p; 505-3p; 191-5p; 136-3p; 154-3p; 491-5p; 125a-5p; 195-5p; 203a-3p; 379-5p; 128-3p; 143-3p; hsa-let-7a-5p; 218-5p; 135a-5p; 423-5p; 543; 485-3p; 26b-3p; 487b-3p; 148a-3p; 21-3p; 129-5p; 29b-1-5p; 378c; 30c-5p; 1248; 30b-5p; 99a-5p; 628-5p; 144-3p; 654-3p; 101-5p; 31-3p; 93-3p; 132-3p; 484; 130a-3p; 3613-5p; 134-5p; 935; 98-5p; 149-5p; 625-5p; 409-5p; 758-3p; 15a-5p; 95-3p; 551b-3p; 361-3p; 194-5p; 411-5p; 129-2-3p; 26a-5p; 296-5p; 106a-5p; 139-5p; 323a-3p; 423-3p; 425-3p; 320c; 1-3p; 132-5p; 335-3p; 144-5p; 33b-5p; 18b-5p; 378a-3p; 629-5p; 590-5p; 432-5p; 7-5p; 320d; 138-5p; 127-3p; 187-3p; 409-3p; 339-5p; 382-5p; 885-5p; 204-5p; 27a-3p; 383-5p; 769-3p; 494-3p; 411-3p; 502-3p; 139-3p; 32-5p; hsa-let-7b-5p; 21-5p; 20b-5p; 136-5p; 30a-3p; 340-5p; 378d; 153-3p; 99b-3p; 338-5p; 454-3p; 23b-3p; 28-3p; 211-5p; 145-5p; 370-3p; 504-5p; 125b-5p; 221-5p; 99b-5p; 126-3p; 323b-3p; 92b-3p; 770-5p; 1296-5p; 1249-3p

19	breast cancer	1.09E-60	199	92a-3p; 106b-3p; 17-5p; 182-5p; 20a-5p; 155-5p; 96-5p; 18a-5p; 1246; 19a-3p; 19b-1-5p; hsa-let-7c-5p; 17-3p; 1290; 142-5p; 183-5p; 15b-3p; hsa-let-7e-5p; 142-3p; 1260a; 92a-1-5p; 744-5p; 16-2-3p; 873-5p; 93-5p; 200c-3p; 19b-3p; 582-5p; 130b-5p; 497-5p; 146a-5p; 766-3p; 181c-5p; 106b-5p; 15b-5p; 4449; 130b-3p; 25-3p; 99a-3p; 210-3p; 9-5p; 486-5p; 425-5p; 16-5p; 125b-2-3p; 154-5p; 192-5p; 30d-5p; 381-3p; 1185-5p; 376a-3p; 376c-3p; 628-3p; 1285-3p; 124-3p; 30a-5p; 410-3p; 486-3p; 186-5p; 9-3p; 127-5p; 299-5p; 31-5p; 625-3p; 655-3p; 451a; 539-5p; 769-5p; 505-3p; 191-5p; 136-3p; 154-3p; 491-5p; 125a-5p; 181c-3p; 195-5p; 203a-3p; 369-5p; 379-5p; 128-3p; 485-5p; 143-3p; hsa-let-7a-5p; 218-5p; 135a-5p; 423-5p; 543; 485-3p; 26b-3p; 487b-3p; 148a-3p; 21-3p; 129-5p; 29b-1-5p; 129-1-3p; 656-3p; 30c-5p; 1248; 30b-5p; 99a-5p; 380-3p; 628-5p; 144-3p; 654-3p; 101-5p; 31-3p; 93-3p; 329-3p; 132-3p; 484; 130a-3p; 3613-5p; 134-5p; 935; 98-5p; 149-5p; 625-5p; 409-5p; 758-3p; 495-3p; 15a-5p; 376a-5p; 487a-3p; 95-3p; 551b-3p; 361-3p; 194-5p; 411-5p; 433-3p; 129-2-3p; 26a-5p; 296-5p; 106a-5p; 139-5p; 323a-3p; 423-3p; 425-3p; 320c; 1-3p; 132-5p; 335-3p; 144-5p; 33b-5p; 181d-5p; 18b-5p; 378a-3p; 629-5p; 590-5p; 487a-5p; 432-5p; 7-5p; 124-5p; 320d; 138-5p; 127-3p; 532-3p; 187-3p; 409-3p; 339-5p; 382-5p; 885-5p; 27b-3p; 340-3p; 204-5p; 27a-3p; 383-5p; 769-3p; 494-3p; 369-3p; 411-3p; 502-3p; 376b-3p; 139-3p; 32-5p; hsa-let-7b-5p; 21-5p; 590-3p; 20b-5p; 136-5p; 30a-3p; 340-5p; 153-3p; 99b-3p; 338-5p; 454-3p; 23b-3p; 331-5p; 28-3p; 211-5p; 145-5p; 370-3p; 504-5p; 125b-5p; 92b-5p; 221-5p; 99b-5p; 126-3p; 92b-3p; 770-5p
20	carcinoma. renal cell	4.97E-60	136	92a-3p; 106b-3p; 182-5p; 155-5p; 96-5p; 18a-5p; 19a-3p; hsa-let-7c-5p; 17-3p; 142-5p; 183-5p; 15b-3p; 142-3p; 92a-1-5p; 744-5p; 16-2-3p; 93-5p; 200c-3p; 19b-3p; 582-5p; 130b-5p; 497-5p; 146a-5p; 766-3p; 181c-5p; 106b-5p; 15b-5p; 130b-3p; 25-3p; 9-5p; 486-5p; 425-5p; 16-5p; 154-5p; 192-5p; 30d-5p; 381-3p; 376c-3p; 628-3p; 30a-5p; 410-3p; 486-3p; 127-5p; 299-5p; 451a; 539-5p; 769-5p; 505-3p; 191-5p; 136-3p; 125a-5p; 181c-3p; 195-5p; 203a-3p; 379-5p; 143-3p; hsa-let-7a-5p; 218-5p; 135a-5p; 423-5p; 485-3p; 26b-3p; 487b-3p; 21-3p; 29b-1-5p; 30c-5p; 30b-5p; 99a-5p; 144-3p; 654-3p; 101-5p; 93-3p; 132-3p; 130a-3p; 3613-5p; 134-5p; 935; 98-5p; 149-5p; 625-5p; 409-5p; 758-3p; 495-3p; 15a-5p; 95-3p; 361-3p; 194-5p; 411-5p; 26a-5p; 106a-5p; 323a-3p; 423-3p; 425-3p; 320c; 1-3p; 132-5p; 335-3p; 144-5p; 33b-5p; 181d-5p; 18b-5p; 378a-3p; 629-5p; 590-5p; 432-5p; 127-3p; 187-3p; 409-3p; 382-5p; 885-5p; 27b-3p; 340-3p; 204-5p; 27a-3p; 369-3p; 502-3p; 139-3p; hsa-let-7b-5p; 21-5p; 590-3p; 20b-5p; 136-5p; 30a-3p; 340-5p; 153-3p; 338-5p; 23b-3p; 331-5p; 145-5p; 370-3p; 221-5p; 99b-5p; 126-3p; 323b-3p; 92b-3p; 1249-3p
21	synovial sarcoma	1.07E-59	97	92a-3p; 106b-3p; 17-5p; 20a-5p; 155-5p; 18a-5p; 19a-3p; 142-5p; hsa-let-7e-5p; 142-3p; 200c-3p; 19b-3p; 146a-5p; 181c-5p; 106b-5p; 130b-3p; 99a-3p; 210-3p; 9-5p; 486-5p; 16-5p; 125b-2-3p; 154-5p; 376a-3p; 376c-3p; 628-3p; 30a-5p; 410-3p; 486-3p; 127-5p; 31-5p; 625-3p; 769-5p; 191-5p; 154-3p; 125a-5p; 369-5p; 379-5p; 485-5p; 143-3p; hsa-let-7a-5p; 218-5p; 543; 485-3p; 487b-3p; 148a-3p; 380-3p; 654-3p; 93-3p; 329-3p; 132-3p; 130a-3p; 134-5p; 98-5p; 149-5p; 409-5p; 495-3p; 15a-5p; 487a-3p; 95-3p; 551b-3p; 411-5p; 433-3p; 26a-5p; 296-5p; 423-3p; 425-3p; 1-3p; 181d-5p; 378a-3p; 432-5p; 7-5p; 127-3p; 409-3p; 382-5p; 204-5p; 27a-3p; 769-3p; 494-3p; 411-3p; 376b-3p; 21-5p; 20b-5p; 136-5p; 30a-3p; 340-5p; 99b-3p; 338-5p; 454-3p; 211-5p; 145-5p; 370-3p; 125b-5p; 92b-5p; 99b-5p; 126-3p; 92b-3p

22	leukemia	6.94E-59	177	92a-3p; 106b-3p; 17-5p; 182-5p; 20a-5p; 155-5p; 96-5p; 18a-5p; 19a-3p; 19b-1-5p; hsa-let-7c-5p; 17-3p; 142-5p; 183-5p; 15b-3p; hsa-let-7e-5p; 142-3p; 92a-1-5p; 744-5p; 16-2-3p; 873-5p; 93-5p; 200c-3p; 19b-3p; 582-5p; 497-5p; 146a-5p; 766-3p; 181c-5p; 106b-5p; 15b-5p; 130b-3p; 25-3p; 210-3p; 9-5p; 486-5p; 425-5p; 16-5p; 125b-2-3p; 154-5p; 192-5p; 30d-5p; 381-3p; 376a-3p; 376c-3p; 628-3p; 124-3p; 30a-5p; 410-3p; 486-3p; 186-5p; 9-3p; 299-5p; 31-5p; 655-3p; 451a; 539-5p; 769-5p; 191-5p; 154-3p; 491-5p; 125a-5p; 181c-3p; 195-5p; 203a-3p; 369-5p; 379-5p; 128-3p; 485-5p; 143-3p; hsa-let-7a-5p; 218-5p; 135a-5p; 423-5p; 543; 485-3p; 26b-3p; 487b-3p; 148a-3p; 129-5p; 29b-1-5p; 129-1-3p; 30c-5p; 30b-5p; 99a-5p; 628-5p; 144-3p; 101-5p; 31-3p; 93-3p; 329-3p; 132-3p; 484; 130a-3p; 134-5p; 935; 98-5p; 149-5p; 625-5p; 409-5p; 758-3p; 495-3p; 15a-5p; 376a-5p; 487a-3p; 95-3p; 551b-3p; 361-3p; 194-5p; 411-5p; 433-3p; 129-2-3p; 26a-5p; 296-5p; 106a-5p; 139-5p; 323a-3p; 423-3p; 425-3p; 320c; 1-3p; 132-5p; 144-5p; 33b-5p; 181d-5p; 18b-5p; 378a-3p; 629-5p; 590-5p; 487a-5p; 432-5p; 7-5p; 124-5p; 320d; 138-5p; 127-3p; 532-3p; 187-3p; 409-3p; 339-5p; 382-5p; 885-5p; 27b-3p; 204-5p; 27a-3p; 383-5p; 769-3p; 494-3p; 369-3p; 411-3p; 502-3p; 376b-3p; 139-3p; 32-5p; hsa-let-7b-5p; 21-5p; 590-3p; 20b-5p; 136-5p; 30a-3p; 340-5p; 153-3p; 338-5p; 454-3p; 23b-3p; 331-5p; 28-3p; 211-5p; 145-5p; 370-3p; 125b-5p; 92b-5p; 99b-5p; 126-3p; 92b-3p; 770-5p; 1296-5p
23	vascular disease	2.16E-58	183	92a-3p; 106b-3p; 17-5p; 182-5p; 20a-5p; 155-5p; 96-5p; 18a-5p; 19a-3p; 19b-1-5p; 17-3p; 142-5p; 183-5p; 15b-3p; 142-3p; 92a-1-5p; 744-5p; 16-2-3p; 873-5p; 93-5p; 200c-3p; 19b-3p; 582-5p; 130b-5p; 497-5p; 146a-5p; 766-3p; 181c-5p; 106b-5p; 15b-5p; 130b-3p; 25-3p; 99a-3p; 210-3p; 9-5p; 486-5p; 425-5p; 16-5p; 125b-2-3p; 154-5p; 192-5p; 30d-5p; 381-3p; 376a-3p; 376c-3p; 628-3p; 124-3p; 30a-5p; 410-3p; 486-3p; 186-5p; 9-3p; 127-5p; 299-5p; 31-5p; 625-3p; 655-3p; 451a; 539-5p; 769-5p; 505-3p; 191-5p; 136-3p; 154-3p; 491-5p; 125a-5p; 181c-3p; 195-5p; 203a-3p; 369-5p; 379-5p; 128-3p; 485-5p; 143-3p; hsa-let-7a-5p; 218-5p; 135a-5p; 423-5p; 543; 485-3p; 26b-3p; 487b-3p; 148a-3p; 21-3p; 129-5p; 29b-1-5p; 129-1-3p; 30c-5p; 30b-5p; 99a-5p; 380-3p; 628-5p; 144-3p; 654-3p; 101-5p; 31-3p; 93-3p; 329-3p; 132-3p; 484; 130a-3p; 134-5p; 935; 98-5p; 149-5p; 625-5p; 409-5p; 758-3p; 495-3p; 15a-5p; 376a-5p; 487a-3p; 95-3p; 551b-3p; 361-3p; 194-5p; 411-5p; 433-3p; 129-2-3p; 26a-5p; 296-5p; 106a-5p; 139-5p; 323a-3p; 423-3p; 425-3p; 1-3p; 132-5p; 335-3p; 144-5p; 33b-5p; 18b-5p; 629-5p; 590-5p; 487a-5p; 432-5p; 7-5p; 124-5p; 138-5p; 127-3p; 532-3p; 187-3p; 409-3p; 339-5p; 382-5p; 885-5p; 27b-3p; 340-3p; 204-5p; 27a-3p; 383-5p; 769-3p; 494-3p; 369-3p; 411-3p; 502-3p; 376b-3p; 139-3p; 32-5p; 21-5p; 590-3p; 20b-5p; 136-5p; 30a-3p; 340-5p; 153-3p; 99b-3p; 338-5p; 454-3p; 23b-3p; 331-5p; 28-3p; 211-5p; 145-5p; 370-3p; 504-5p; 125b-5p; 92b-5p; 221-5p; 99b-5p; 126-3p; 92b-3p; 770-5p
24	lung small cell carcinoma	5.58E-58	118	92a-3p; 106b-3p; 17-5p; 182-5p; 20a-5p; 155-5p; 96-5p; 18a-5p; 1246; 19a-3p; 19b-1-5p; 17-3p; 1290; 142-5p; 183-5p; 15b-3p; 142-3p; 1260a; 16-2-3p; 873-5p; 93-5p; 200c-3p; 19b-3p; 497-5p; 146a-5p; 106b-5p; 15b-5p; 130b-3p; 25-3p; 210-3p; 9-5p; 486-5p; 425-5p; 16-5p; 125b-2-3p; 30d-5p; 381-3p; 376c-3p; 124-3p; 30a-5p; 410-3p; 9-3p; 31-5p; 451a; 539-5p; 769-5p; 505-3p; 191-5p; 491-5p; 181c-3p; 195-5p; 203a-3p; 379-5p; 128-3p; 143-3p; hsa-let-7a-5p; 218-5p; 135a-5p; 543; 485-3p; 487b-3p; 148a-3p; 21-3p; 129-5p; 29b-1-5p; 129-1-3p; 30c-5p; 30b-5p; 99a-5p; 628-5p; 144-3p; 654-3p; 101-5p; 31-3p; 329-3p; 130a-3p; 134-5p; 98-5p; 149-5p; 625-5p; 758-3p; 495-3p; 15a-5p; 376a-5p; 95-3p; 551b-3p; 361-3p; 433-3p; 26a-5p; 106a-5p; 139-5p; 323a-3p; 1-3p; 335-3p; 144-5p; 181d-5p; 18b-5p; 7-5p; 138-5p; 409-3p; 885-5p; 340-3p; 27a-3p; 383-5p; 494-3p; 376b-3p; 139-3p; 21-5p; 20b-5p; 30a-3p; 340-5p; 153-3p; 99b-3p; 454-3p; 145-5p; 125b-5p; 221-5p; 126-3p

25	lung squamous cell carcinoma	1.46E-57	156	92a-3p; 106b-3p; 17-5p; 182-5p; 20a-5p; 155-5p; 96-5p; 18a-5p; 19a-3p; 19b-1-5p; hsa-let-7c-5p; 17-3p; 4521; 1290; 142-5p; 183-5p; 15b-3p; hsa-let-7e-5p; 142-3p; 92a-1-5p; 16-2-3p; 93-5p; 200c-3p; 19b-3p; 582-5p; 130b-5p; 497-5p; 146a-5p; 766-3p; 181c-5p; 106b-5p; 15b-5p; 4449; 130b-3p; 25-3p; 3615; 99a-3p; 210-3p; 9-5p; 486-5p; 425-5p; 16-5p; 125b-2-3p; 192-5p; 30d-5p; 381-3p; 30a-5p; 410-3p; 186-5p; 9-3p; 127-5p; 31-5p; 625-3p; 451a; 539-5p; 769-5p; 505-3p; 191-5p; 136-3p; 491-5p; 125a-5p; 181c-3p; 195-5p; 203a-3p; 379-5p; 128-3p; 485-5p; 143-3p; hsa-let-7a-5p; 218-5p; 135a-5p; 26b-3p; 148a-3p; 21-3p; 129-5p; 378c; 30c-5p; 30b-5p; 99a-5p; 380-3p; 628-5p; 144-3p; 101-5p; 31-3p; 93-3p; 329-3p; 132-3p; 484; 130a-3p; 134-5p; 98-5p; 149-5p; 625-5p; 409-5p; 15a-5p; 551b-3p; 361-3p; 194-5p; 26a-5p; 106a-5p; 139-5p; 323a-3p; 423-3p; 425-3p; 320c; 1-3p; 132-5p; 3065-5p; 335-3p; 144-5p; 33b-5p; 181d-5p; 18b-5p; 378a-3p; 629-5p; 590-5p; 432-5p; 7-5p; 320d; 138-5p; 409-3p; 339-5p; 382-5p; 27b-3p; 340-3p; 27a-3p; 383-5p; 769-3p; 494-3p; 369-3p; 502-3p; 139-3p; 32-5p; hsa-let-7b-5p; 21-5p; 590-3p; 20b-5p; 136-5p; 30a-3p; 340-5p; 378d; 99b-3p; 338-5p; 454-3p; 23b-3p; 28-3p; 211-5p; 145-5p; 370-3p; 504-5p; 125b-5p; 221-5p; 99b-5p; 126-3p; 323b-3p; 1249-3p
26	progesterone-receptor positive breast cancer	2.13E-57	169	92a-3p; 106b-3p; 17-5p; 182-5p; 20a-5p; 155-5p; 96-5p; 18a-5p; 19a-3p; 19b-1-5p; hsa-let-7c-5p; 17-3p; 1290; 142-5p; 183-5p; 15b-3p; hsa-let-7e-5p; 142-3p; 1260a; 744-5p; 873-5p; 93-5p; 200c-3p; 497-5p; 146a-5p; 766-3p; 181c-5p; 106b-5p; 15b-5p; 130b-3p; 25-3p; 210-3p; 9-5p; 486-5p; 425-5p; 16-5p; 125b-2-3p; 154-5p; 192-5p; 381-3p; 1185-5p; 376a-3p; 376c-3p; 628-3p; 124-3p; 30a-5p; 410-3p; 486-3p; 9-3p; 127-5p; 299-5p; 31-5p; 625-3p; 655-3p; 451a; 539-5p; 769-5p; 505-3p; 191-5p; 136-3p; 154-3p; 125a-5p; 181c-3p; 195-5p; 203a-3p; 369-5p; 379-5p; 128-3p; 143-3p; hsa-let-7a-5p; 218-5p; 135a-5p; 423-5p; 543; 485-3p; 487b-3p; 148a-3p; 21-3p; 129-5p; 29b-1-5p; 656-3p; 30c-5p; 1248; 30b-5p; 99a-5p; 380-3p; 654-3p; 101-5p; 31-3p; 93-3p; 329-3p; 132-3p; 484; 130a-3p; 134-5p; 935; 98-5p; 149-5p; 625-5p; 409-5p; 758-3p; 495-3p; 15a-5p; 376a-5p; 487a-3p; 95-3p; 551b-3p; 361-3p; 194-5p; 411-5p; 433-3p; 129-2-3p; 26a-5p; 296-5p; 106a-5p; 139-5p; 423-3p; 425-3p; 320c; 1-3p; 132-5p; 335-3p; 144-5p; 33b-5p; 181d-5p; 378a-3p; 629-5p; 590-5p; 432-5p; 7-5p; 124-5p; 320d; 138-5p; 127-3p; 532-3p; 187-3p; 409-3p; 339-5p; 382-5p; 27b-3p; 204-5p; 383-5p; 769-3p; 494-3p; 502-3p; 139-3p; 32-5p; hsa-let-7b-5p; 21-5p; 590-3p; 20b-5p; 30a-3p; 340-5p; 153-3p; 99b-3p; 338-5p; 454-3p; 331-5p; 28-3p; 145-5p; 370-3p; 504-5p; 125b-5p; 92b-5p; 221-5p; 99b-5p; 126-3p; 92b-3p; 770-5p
27	thyroid carcinoma	4.01E-57	142	92a-3p; 106b-3p; 17-5p; 182-5p; 20a-5p; 155-5p; 96-5p; 18a-5p; 19a-3p; 19b-1-5p; hsa-let-7c-5p; 17-3p; 142-5p; 183-5p; 15b-3p; hsa-let-7e-5p; 142-3p; 92a-1-5p; 744-5p; 16-2-3p; 200c-3p; 19b-3p; 130b-5p; 497-5p; 146a-5p; 181c-5p; 106b-5p; 130b-3p; 25-3p; 3615; 210-3p; 9-5p; 486-5p; 425-5p; 16-5p; 154-5p; 192-5p; 30d-5p; 381-3p; 376c-3p; 628-3p; 30a-5p; 410-3p; 486-3p; 186-5p; 127-5p; 299-5p; 31-5p; 625-3p; 655-3p; 451a; 539-5p; 769-5p; 136-3p; 154-3p; 125a-5p; 181c-3p; 195-5p; 203a-3p; 369-5p; 379-5p; 128-3p; 143-3p; 218-5p; 485-3p; 26b-3p; 148a-3p; 21-3p; 129-5p; 29b-1-5p; 378c; 30c-5p; 30b-5p; 99a-5p; 628-5p; 144-3p; 654-3p; 31-3p; 132-3p; 130a-3p; 3613-5p; 134-5p; 935; 98-5p; 149-5p; 625-5p; 409-5p; 758-3p; 495-3p; 15a-5p; 95-3p; 551b-3p; 361-3p; 411-5p; 26a-5p; 106a-5p; 139-5p; 323a-3p; 423-3p; 425-3p; 1-3p; 132-5p; 3065-5p; 335-3p; 144-5p; 33b-5p; 181d-5p; 629-5p; 590-5p; 7-5p; 320d; 138-5p; 127-3p; 532-3p; 187-3p; 409-3p; 382-5p; 27b-3p; 340-3p; 204-5p; 369-3p; 502-3p; 139-3p; hsa-let-7b-5p; 21-5p; 20b-5p; 136-5p; 30a-3p; 153-3p; 99b-3p; 338-5p; 23b-3p; 331-5p; 28-3p; 145-5p; 125b-5p; 221-5p; 99b-5p; 126-3p; 92b-3p; 1296-5p; 1249-3p

28	progesterone-receptor negative breast cancer	8.06E-55	162	17-5p; 182-5p; 20a-5p; 155-5p; 96-5p; 18a-5p; 1246; 19a-3p; 19b-1-5p; hsa-let-7c-5p; 17-3p; 1290; 142-5p; 183-5p; 15b-3p; hsa-let-7e-5p; 142-3p; 1260a; 744-5p; 16-2-3p; 873-5p; 93-5p; 200c-3p; 19b-3p; 582-5p; 497-5p; 146a-5p; 766-3p; 181c-5p; 106b-5p; 15b-5p; 130b-3p; 25-3p; 99a-3p; 210-3p; 9-5p; 486-5p; 425-5p; 16-5p; 125b-2-3p; 154-5p; 192-5p; 30d-5p; 381-3p; 1185-5p; 376a-3p; 376c-3p; 628-3p; 124-3p; 30a-5p; 410-3p; 486-3p; 9-3p; 127-5p; 299-5p; 31-5p; 625-3p; 655-3p; 451a; 539-5p; 769-5p; 191-5p; 136-3p; 154-3p; 125a-5p; 195-5p; 203a-3p; 369-5p; 379-5p; 128-3p; 143-3p; hsa-let-7a-5p; 218-5p; 135a-5p; 423-5p; 543; 485-3p; 26b-3p; 487b-3p; 148a-3p; 21-3p; 29b-1-5p; 656-3p; 30c-5p; 1248; 30b-5p; 99a-5p; 380-3p; 144-3p; 654-3p; 101-5p; 31-3p; 329-3p; 132-3p; 484; 130a-3p; 134-5p; 935; 98-5p; 149-5p; 625-5p; 409-5p; 758-3p; 495-3p; 15a-5p; 376a-5p; 487a-3p; 95-3p; 551b-3p; 194-5p; 411-5p; 129-2-3p; 26a-5p; 296-5p; 106a-5p; 139-5p; 425-3p; 320c; 1-3p; 132-5p; 335-3p; 144-5p; 18b-5p; 378a-3p; 629-5p; 590-5p; 432-5p; 7-5p; 320d; 127-3p; 187-3p; 409-3p; 339-5p; 382-5p; 340-3p; 204-5p; 27a-3p; 383-5p; 769-3p; 502-3p; 139-3p; 32-5p; hsa-let-7b-5p; 21-5p; 590-3p; 20b-5p; 136-5p; 30a-3p; 340-5p; 99b-3p; 338-5p; 454-3p; 28-3p; 145-5p; 370-3p; 504-5p; 125b-5p; 92b-5p; 221-5p; 99b-5p; 126-3p; 92b-3p
29	oral squamous cell carcinoma	1.53E-54	137	92a-3p; 106b-3p; 17-5p; 20a-5p; 155-5p; 96-5p; 18a-5p; 1246; 19a-3p; hsa-let-7c-5p; 17-3p; 1290; 142-5p; 183-5p; 15b-3p; hsa-let-7e-5p; 142-3p; 93-5p; 200c-3p; 19b-3p; 497-5p; 146a-5p; 106b-5p; 15b-5p; 130b-3p; 25-3p; 99a-3p; 210-3p; 9-5p; 486-5p; 16-5p; 125b-2-3p; 154-5p; 192-5p; 30d-5p; 381-3p; 376a-3p; 376c-3p; 628-3p; 124-3p; 30a-5p; 410-3p; 486-3p; 9-3p; 299-5p; 31-5p; 655-3p; 191-5p; 136-3p; 125a-5p; 195-5p; 203a-3p; 369-5p; 379-5p; 128-3p; 485-5p; 143-3p; hsa-let-7a-5p; 218-5p; 135a-5p; 423-5p; 543; 26b-3p; 487b-3p; 148a-3p; 21-3p; 656-3p; 30c-5p; 30b-5p; 99a-5p; 628-5p; 144-3p; 101-5p; 31-3p; 329-3p; 132-3p; 484; 130a-3p; 134-5p; 935; 98-5p; 149-5p; 625-5p; 495-3p; 15a-5p; 376a-5p; 95-3p; 411-5p; 26a-5p; 296-5p; 106a-5p; 139-5p; 320c; 1-3p; 144-5p; 33b-5p; 18b-5p; 378a-3p; 590-5p; 432-5p; 7-5p; 138-5p; 127-3p; 532-3p; 187-3p; 409-3p; 339-5p; 382-5p; 885-5p; 27b-3p; 204-5p; 27a-3p; 383-5p; 769-3p; 494-3p; 369-3p; 411-3p; 139-3p; 32-5p; hsa-let-7b-5p; 21-5p; 20b-5p; 136-5p; 30a-3p; 340-5p; 153-3p; 99b-3p; 454-3p; 23b-3p; 331-5p; 211-5p; 145-5p; 370-3p; 125b-5p; 221-5p; 126-3p; 92b-3p
30	colon adenocarcinoma	1.8E-54	124	92a-3p; 106b-3p; 17-5p; 182-5p; 20a-5p; 96-5p; 18a-5p; 19a-3p; 19b-1-5p; hsa-let-7c-5p; 17-3p; 142-5p; 183-5p; 15b-3p; hsa-let-7e-5p; 142-3p; 92a-1-5p; 744-5p; 200c-3p; 19b-3p; 582-5p; 130b-5p; 497-5p; 146a-5p; 766-3p; 181c-5p; 106b-5p; 15b-5p; 130b-3p; 25-3p; 3615; 486-5p; 16-5p; 125b-2-3p; 192-5p; 30d-5p; 30a-5p; 186-5p; 127-5p; 625-3p; 451a; 769-5p; 505-3p; 191-5p; 136-3p; 491-5p; 125a-5p; 195-5p; 203a-3p; 379-5p; 143-3p; hsa-let-7a-5p; 218-5p; 423-5p; 485-3p; 148a-3p; 21-3p; 129-5p; 29b-1-5p; 378c; 30c-5p; 30b-5p; 654-3p; 101-5p; 132-3p; 484; 130a-3p; 98-5p; 149-5p; 625-5p; 409-5p; 758-3p; 15a-5p; 95-3p; 361-3p; 194-5p; 433-3p; 26a-5p; 296-5p; 106a-5p; 139-5p; 323a-3p; 423-3p; 425-3p; 320c; 1-3p; 335-3p; 144-5p; 181d-5p; 18b-5p; 378a-3p; 590-5p; 487a-5p; 432-5p; 127-3p; 532-3p; 409-3p; 339-5p; 382-5p; 340-3p; 204-5p; 27a-3p; 494-3p; 369-3p; 376b-3p; 139-3p; 32-5p; hsa-let-7b-5p; 21-5p; 136-5p; 30a-3p; 99b-3p; 338-5p; 454-3p; 23b-3p; 28-3p; 145-5p; 370-3p; 125b-5p; 99b-5p; 126-3p; 92b-3p; 1296-5p; 1249-3p
31	carcinoma	4.43E-54	170	92a-3p; 106b-3p; 17-5p; 182-5p; 20a-5p; 155-5p; 96-5p; 18a-5p; 19a-3p; 19b-1-5p; 17-3p; 142-5p; 183-5p; 15b-3p; 142-3p; 92a-1-5p; 744-5p; 873-5p; 93-5p; 200c-3p; 19b-3p; 582-5p; 497-5p; 146a-5p; 181c-5p; 106b-5p; 15b-5p; 130b-3p; 25-3p; 99a-3p; 210-3p; 9-5p; 486-5p; 425-5p; 16-5p; 125b-2-3p; 154-5p; 192-5p; 30d-5p; 381-3p; 376a-3p; 376c-3p; 124-3p; 30a-5p; 410-3p; 486-3p; 186-5p; 9-3p; 127-5p; 299-5p; 31-5p; 655-3p; 451a; 539-5p; 769-5p; 505-3p; 191-5p; 136-3p; 491-5p; 125a-5p; 181c-3p; 195-5p; 203a-3p; 369-5p; 379-5p; 128-3p; 485-5p; 143-3p; hsa-let-7a-5p; 218-5p; 135a-5p; 423-5p; 543; 485-3p; 26b-3p; 487b-3p; 148a-3p; 21-3p; 129-5p; 29b-1-5p; 129-1-3p; 30c-5p; 30b-5p; 99a-5p; 628-5p; 144-3p; 654-3p; 101-5p; 31-3p; 93-3p; 329-3p; 132-3p; 484; 130a-3p; 134-5p; 935; 98-5p; 149-5p; 625-5p; 409-5p; 758-3p; 495-3p; 15a-5p; 376a-5p; 487a-3p; 95-3p; 551b-3p; 361-3p; 194-5p; 411-5p; 433-3p; 129-2-3p; 26a-5p; 296-5p; 106a-5p; 139-5p; 323a-3p; 423-3p; 320c; 1-3p; 132-5p; 33b-5p; 18b-5p; 629-5p; 590-5p; 487a-5p; 432-5p; 7-5p; 124-5p; 320d; 138-5p; 127-3p; 532-3p; 187-3p; 409-3p; 339-5p; 382-5p; 885-5p; 27b-3p; 204-5p; 27a-3p; 383-5p; 769-3p; 494-3p; 369-3p; 411-3p; 502-3p; 376b-3p; 139-3p; 32-5p; 21-5p; 590-3p; 20b-5p; 30a-3p; 340-5p; 153-3p; 338-5p; 454-3p; 23b-3p; 331-5p; 28-3p;

				211-5p; 145-5p; 370-3p; 504-5p; 125b-5p; 92b-5p; 126-3p; 92b-3p; 770-5p
32	amyotrophic lateral sclerosis	1.06E-53	128	4488; 92a-3p; 106b-3p; 17-5p; 20a-5p; 155-5p; 18a-5p; 1246; 19a-3p; hsa-let-7c-5p; 1290; 3960; 142-5p; hsa-let-7e-5p; 142-3p; 744-5p; 873-5p; 93-5p; 19b-3p; 582-5p; 146a-5p; 766-3p; 181c-5p; 106b-5p; 15b-5p; 130b-3p; 25-3p; 210-3p; 9-5p; 16-5p; 154-5p; 192-5p; 381-3p; 376a-3p; 124-3p; 127-5p; 299-5p; 31-5p; 625-3p; 655-3p; 451a; 539-5p; 505-3p; 491-5p; 125a-5p; 195-5p; 369-5p; 379-5p; 128-3p; 485-5p; 143-3p; hsa-let-7a-5p; 218-5p; 135a-5p; 423-5p; 543; 485-3p; 487b-3p; 148a-3p; 129-5p; 30c-5p; 30b-5p; 99a-5p; 380-3p; 654-3p; 329-3p; 132-3p; 484; 130a-3p; 3613-5p; 134-5p; 98-5p; 149-5p; 625-5p; 409-5p; 758-3p; 495-3p; 15a-5p; 487a-3p; 551b-3p; 194-5p; 433-3p; 129-2-3p; 26a-5p; 296-5p; 106a-5p; 139-5p; 323a-3p; 425-3p; 1-3p; 132-5p; 18b-5p; 629-5p; 487a-5p; 432-5p; 7-5p; 138-5p; 127-3p; 532-3p; 409-3p; 339-5p; 382-5p; 885-5p; 27b-3p; 204-5p; 27a-3p; 383-5p; 494-3p; 369-3p; 502-3p; 139-3p; hsa-let-7b-5p; 21-5p; 20b-5p; 340-5p; 153-3p; 99b-3p; 338-5p; 454-3p; 23b-3p; 331-5p; 28-3p; 211-5p; 145-5p; 370-3p; 504-5p; 125b-5p; 99b-5p
33	medulloblastoma	2.49E-52	118	92a-3p; 106b-3p; 17-5p; 182-5p; 20a-5p; 155-5p; 96-5p; 18a-5p; 19a-3p; hsa-let-7c-5p; 17-3p; 1290; 142-5p; 183-5p; hsa-let-7e-5p; 142-3p; 1260b; 1260a; 93-5p; 200c-3p; 19b-3p; 582-5p; 497-5p; 146a-5p; 766-3p; 181c-5p; 106b-5p; 130b-3p; 25-3p; 210-3p; 9-5p; 486-5p; 425-5p; 16-5p; 154-5p; 192-5p; 30d-5p; 376a-3p; 376c-3p; 124-3p; 30a-5p; 410-3p; 186-5p; 9-3p; 31-5p; 451a; 539-5p; 191-5p; 491-5p; 125a-5p; 181c-3p; 195-5p; 203a-3p; 379-5p; 128-3p; 143-3p; hsa-let-7a-5p; 218-5p; 4286; 135a-5p; 26b-3p; 487b-3p; 148a-3p; 21-3p; 129-5p; 30c-5p; 30b-5p; 132-3p; 130a-3p; 134-5p; 935; 98-5p; 149-5p; 495-3p; 15a-5p; 361-3p; 194-5p; 433-3p; 26a-5p; 106a-5p; 139-5p; 323a-3p; 425-3p; 320c; 1-3p; 132-5p; 144-5p; 33b-5p; 181d-5p; 432-5p; 7-5p; 138-5p; 127-3p; 187-3p; 409-3p; 382-5p; 27b-3p; 204-5p; 27a-3p; 383-5p; 494-3p; 139-3p; 32-5p; hsa-let-7b-5p; 21-5p; 20b-5p; 136-5p; 340-5p; 153-3p; 338-5p; 23b-3p; 145-5p; 370-3p; 125b-5p; 92b-5p; 221-5p; 99b-5p; 126-3p
34	melanoma	2.49E-52	154	92a-3p; 17-5p; 182-5p; 20a-5p; 155-5p; 96-5p; 18a-5p; 19a-3p; hsa-let-7c-5p; 17-3p; 1290; 142-5p; 183-5p; 15b-3p; hsa-let-7e-5p; 142-3p; 92a-1-5p; 744-5p; 873-5p; 93-5p; 200c-3p; 19b-3p; 582-5p; 130b-5p; 497-5p; 146a-5p; 766-3p; 181c-5p; 106b-5p; 15b-5p; 130b-3p; 25-3p; 99a-3p; 210-3p; 9-5p; 486-5p; 425-5p; 16-5p; 125b-2-3p; 154-5p; 192-5p; 30d-5p; 381-3p; 1185-5p; 376a-3p; 376c-3p; 1285-3p; 124-3p; 30a-5p; 410-3p; 486-3p; 186-5p; 9-3p; 299-5p; 31-5p; 655-3p; 451a; 539-5p; 191-5p; 154-3p; 491-5p; 125a-5p; 195-5p; 203a-3p; 369-5p; 379-5p; 128-3p; 485-5p; 143-3p; hsa-let-7a-5p; 218-5p; 135a-5p; 485-3p; 26b-3p; 487b-3p; 148a-3p; 21-3p; 129-5p; 30c-5p; 1248; 30b-5p; 99a-5p; 628-5p; 144-3p; 101-5p; 329-3p; 132-3p; 484; 130a-3p; 134-5p; 98-5p; 149-5p; 625-5p; 409-5p; 758-3p; 495-3p; 15a-5p; 487a-3p; 95-3p; 194-5p; 411-5p; 433-3p; 26a-5p; 296-5p; 106a-5p; 139-5p; 323a-3p; 423-3p; 425-3p; 1-3p; 132-5p; 335-3p; 33b-5p; 181d-5p; 18b-5p; 378a-3p; 629-5p; 590-5p; 7-5p; 138-5p; 127-3p; 409-3p; 339-5p; 382-5p; 885-5p; 27b-3p; 204-5p; 27a-3p; 383-5p; 769-3p; 494-3p; 369-3p; 32-5p; hsa-let-7b-5p; 21-5p; 20b-5p; 136-5p; 30a-3p; 340-5p; 338-5p; 454-3p; 23b-3p; 211-5p; 145-5p; 370-3p; 504-5p; 125b-5p; 92b-5p; 221-5p; 99b-5p; 126-3p; 770-5p; 1296-5p; 1249-3p

35	**lymphoma**	4.12E-52	145	92a-3p; 106b-3p; 17-5p; 182-5p; 20a-5p; 155-5p; 96-5p; 18a-5p; 19a-3p; 19b-1-5p; 17-3p; 142-5p; 183-5p; 15b-3p; 142-3p; 92a-1-5p; 744-5p; 16-2-3p; 873-5p; 93-5p; 200c-3p; 19b-3p; 582-5p; 130b-5p; 497-5p; 146a-5p; 181c-5p; 106b-5p; 15b-5p; 130b-3p; 25-3p; 9-5p; 486-5p; 425-5p; 16-5p; 192-5p; 30d-5p; 381-3p; 376a-3p; 376c-3p; 30a-5p; 410-3p; 486-3p; 186-5p; 9-3p; 127-5p; 299-5p; 31-5p; 625-3p; 451a; 539-5p; 769-5p; 505-3p; 191-5p; 154-3p; 125a-5p; 181c-3p; 195-5p; 203a-3p; 379-5p; 128-3p; 143-3p; hsa-let-7a-5p; 485-3p; 26b-3p; 487b-3p; 148a-3p; 21-3p; 29b-1-5p; 129-1-3p; 30c-5p; 30b-5p; 99a-5p; 628-5p; 144-3p; 654-3p; 101-5p; 93-3p; 132-3p; 484; 130a-3p; 134-5p; 98-5p; 149-5p; 625-5p; 409-5p; 758-3p; 15a-5p; 487a-3p; 95-3p; 551b-3p; 361-3p; 194-5p; 411-5p; 433-3p; 129-2-3p; 26a-5p; 296-5p; 106a-5p; 139-5p; 423-3p; 425-3p; 1-3p; 132-5p; 18b-5p; 378a-3p; 629-5p; 590-5p; 7-5p; 124-5p; 138-5p; 532-3p; 339-5p; 382-5p; 27b-3p; 340-3p; 204-5p; 27a-3p; 769-3p; 494-3p; 411-3p; 502-3p; 139-3p; 32-5p; hsa-let-7b-5p; 21-5p; 590-3p; 20b-5p; 30a-3p; 340-5p; 99b-3p; 338-5p; 454-3p; 23b-3p; 28-3p; 211-5p; 145-5p; 370-3p; 125b-5p; 92b-5p; 221-5p; 99b-5p; 126-3p; 92b-3p; 770-5p
36	**Burkitt lymphoma**	1.65E-51	95	92a-3p; 106b-3p; 17-5p; 182-5p; 20a-5p; 155-5p; 96-5p; 18a-5p; 19a-3p; hsa-let-7c-5p; 17-3p; 142-5p; 183-5p; hsa-let-7e-5p; 142-3p; 93-5p; 200c-3p; 19b-3p; 130b-5p; 497-5p; 146a-5p; 181c-5p; 106b-5p; 15b-5p; 130b-3p; 25-3p; 210-3p; 9-5p; 486-5p; 16-5p; 30d-5p; 376c-3p; 124-3p; 30a-5p; 410-3p; 31-5p; 191-5p; 136-3p; 154-3p; 181c-3p; 203a-3p; 128-3p; 143-3p; hsa-let-7a-5p; 218-5p; 543; 485-3p; 148a-3p; 129-5p; 30c-5p; 30b-5p; 99a-5p; 144-3p; 93-3p; 132-3p; 484; 130a-3p; 134-5p; 98-5p; 15a-5p; 95-3p; 361-3p; 194-5p; 129-2-3p; 26a-5p; 106a-5p; 139-5p; 323a-3p; 423-3p; 1-3p; 132-5p; 335-3p; 378a-3p; 7-5p; 124-5p; 138-5p; 127-3p; 187-3p; 409-3p; 339-5p; 27b-3p; 204-5p; 27a-3p; 383-5p; 494-3p; hsa-let-7b-5p; 21-5p; 136-5p; 30a-3p; 340-5p; 153-3p; 145-5p; 125b-5p; 99b-5p; 126-3p
37	**brain disease**	1.74E-51	166	92a-3p; 106b-3p; 17-5p; 182-5p; 20a-5p; 155-5p; 96-5p; 18a-5p; 19a-3p; 19b-1-5p; 17-3p; 142-5p; 183-5p; 15b-3p; 142-3p; 92a-1-5p; 744-5p; 873-5p; 93-5p; 200c-3p; 19b-3p; 582-5p; 130b-5p; 497-5p; 146a-5p; 181c-5p; 106b-5p; 15b-5p; 130b-3p; 25-3p; 99a-3p; 210-3p; 9-5p; 486-5p; 425-5p; 16-5p; 125b-2-3p; 154-5p; 192-5p; 30d-5p; 381-3p; 376a-3p; 376c-3p; 628-3p; 124-3p; 30a-5p; 410-3p; 486-3p; 186-5p; 127-5p; 299-5p; 31-5p; 655-3p; 451a; 769-5p; 505-3p; 191-5p; 136-3p; 154-3p; 491-5p; 125a-5p; 181c-3p; 195-5p; 369-5p; 379-5p; 128-3p; 485-5p; 143-3p; hsa-let-7a-5p; 218-5p; 135a-5p; 423-5p; 543; 485-3p; 487b-3p; 148a-3p; 21-3p; 129-5p; 30c-5p; 30b-5p; 99a-5p; 628-5p; 144-3p; 654-3p; 101-5p; 31-3p; 93-3p; 329-3p; 132-3p; 484; 130a-3p; 134-5p; 935; 98-5p; 149-5p; 625-5p; 409-5p; 758-3p; 495-3p; 15a-5p; 376a-5p; 487a-3p; 95-3p; 551b-3p; 361-3p; 194-5p; 411-5p; 433-3p; 129-2-3p; 26a-5p; 296-5p; 106a-5p; 139-5p; 323a-3p; 423-3p; 425-3p; 1-3p; 144-5p; 33b-5p; 18b-5p; 629-5p; 590-5p; 487a-5p; 432-5p; 7-5p; 124-5p; 138-5p; 127-3p; 532-3p; 187-3p; 409-3p; 339-5p; 382-5p; 885-5p; 27b-3p; 204-5p; 27a-3p; 383-5p; 769-3p; 494-3p; 369-3p; 502-3p; 376b-3p; 139-3p; 32-5p; 21-5p; 590-3p; 20b-5p; 136-5p; 30a-3p; 340-5p; 153-3p; 338-5p; 454-3p; 23b-3p; 331-5p; 211-5p; 145-5p; 370-3p; 504-5p; 125b-5p; 92b-5p; 99b-5p; 126-3p; 92b-3p; 770-5p
38	**neurodegenerative disease**	6.57E-51	169	92a-3p; 106b-3p; 17-5p; 182-5p; 20a-5p; 155-5p; 96-5p; 18a-5p; 19a-3p; 19b-1-5p; 17-3p; 142-5p; 183-5p; 15b-3p; 142-3p; 92a-1-5p; 744-5p; 873-5p; 93-5p; 200c-3p; 19b-3p; 582-5p; 130b-5p; 497-5p; 146a-5p; 181c-5p; 15b-5p; 130b-3p; 25-3p; 210-3p; 9-5p; 486-5p; 425-5p; 16-5p; 125b-2-3p; 154-5p; 192-5p; 30d-5p; 381-3p; 376a-3p; 376c-3p; 628-3p; 124-3p; 30a-5p; 410-3p; 486-3p; 186-5p; 9-3p; 127-5p; 299-5p; 31-5p; 655-3p; 451a; 769-5p; 505-3p; 191-5p; 136-3p; 154-3p; 491-5p; 125a-5p; 181c-3p; 195-5p; 203a-3p; 369-5p; 379-5p; 128-3p; 485-5p; 143-3p; hsa-let-7a-5p; 218-5p; 135a-5p; 423-5p; 543; 485-3p; 26b-3p; 487b-3p; 148a-3p; 21-3p; 129-5p; 29b-1-5p; 30c-5p; 30b-5p; 99a-5p; 628-5p; 144-3p; 654-3p; 101-5p; 31-3p; 93-3p; 329-3p; 132-3p; 484; 130a-3p; 134-5p; 935; 98-5p; 149-5p; 625-5p; 409-5p; 758-3p; 495-3p; 15a-5p; 376a-5p; 487a-3p; 95-3p; 551b-3p; 361-3p; 194-5p; 411-5p; 433-3p; 129-2-3p; 26a-5p; 296-5p; 106a-5p; 139-5p; 323a-3p; 423-3p; 1-3p; 335-3p; 144-5p; 33b-5p; 629-5p; 590-5p; 487a-5p; 432-5p; 7-5p; 124-5p; 138-5p; 532-3p; 187-3p; 409-3p; 339-5p; 382-5p; 885-5p; 27b-3p; 204-5p; 27a-3p; 383-5p; 769-3p; 494-3p; 369-3p; 502-3p; 376b-3p; 139-3p; 32-5p; 21-5p; 590-3p; 20b-5p; 136-5p; 30a-3p; 340-5p; 153-3p; 99b-3p; 338-5p; 454-3p; 23b-3p; 331-5p; 28-3p; 211-5p; 145-5p; 370-3p; 504-5p; 125b-5p; 92b-5p; 221-5p; 99b-5p; 126-3p; 92b-3p; 770-5p

39	rectum adenocarcinoma	8.6E-51	117	92a-3p; 106b-3p; 17-5p; 182-5p; 20a-5p; 155-5p; 96-5p; 19a-3p; 19b-1-5p; hsa-let-7c-5p; 17-3p; 142-5p; 183-5p; 15b-3p; 142-3p; 744-5p; 16-2-3p; 19b-3p; 582-5p; 497-5p; 146a-5p; 766-3p; 106b-5p; 15b-5p; 130b-3p; 25-3p; 3615; 486-5p; 425-5p; 16-5p; 125b-2-3p; 192-5p; 30d-5p; 381-3p; 30a-5p; 186-5p; 127-5p; 31-5p; 625-3p; 451a; 539-5p; 769-5p; 505-3p; 136-3p; 491-5p; 125a-5p; 195-5p; 203a-3p; 379-5p; 143-3p; 218-5p; 423-5p; 485-3p; 26b-3p; 148a-3p; 21-3p; 129-5p; 378c; 30c-5p; 1248; 30b-5p; 101-5p; 31-3p; 132-3p; 484; 130a-3p; 98-5p; 149-5p; 382-3p; 409-5p; 758-3p; 15a-5p; 376a-5p; 361-3p; 194-5p; 433-3p; 26a-5p; 296-5p; 106a-5p; 139-5p; 323a-3p; 423-3p; 1-3p; 3065-5p; 335-3p; 144-5p; 181d-5p; 378a-3p; 590-5p; 487a-5p; 432-5p; 127-3p; 532-3p; 339-5p; 27a-3p; 769-3p; 369-3p; 411-3p; 139-3p; 32-5p; hsa-let-7b-5p; 21-5p; 20b-5p; 136-5p; 30a-3p; 99b-3p; 338-5p; 28-3p; 145-5p; 370-3p; 125b-5p; 99b-5p; 126-3p; 323b-3p; 92b-3p; 1296-5p; 1249-3p
40	adrenocortical carcinoma	1.89E-50	89	92a-3p; 17-5p; 182-5p; 20a-5p; 155-5p; 18a-5p; 19a-3p; 142-5p; 183-5p; 15b-3p; 142-3p; 93-5p; 200c-3p; 19b-3p; 497-5p; 146a-5p; 15b-5p; 130b-3p; 9-5p; 486-5p; 154-5p; 192-5p; 381-3p; 376a-3p; 376c-3p; 124-3p; 30a-5p; 410-3p; 127-5p; 299-5p; 31-5p; 655-3p; 539-5p; 136-3p; 154-3p; 491-5p; 125a-5p; 195-5p; 369-5p; 379-5p; 143-3p; hsa-let-7a-5p; 218-5p; 543; 485-3p; 487b-3p; 148a-3p; 656-3p; 378c; 99a-5p; 380-3p; 654-3p; 329-3p; 132-3p; 484; 130a-3p; 134-5p; 98-5p; 382-3p; 409-5p; 758-3p; 495-3p; 15a-5p; 376a-5p; 487a-3p; 411-5p; 433-3p; 139-5p; 323a-3p; 33b-5p; 487a-5p; 432-5p; 127-3p; 409-3p; 382-5p; 27a-3p; 494-3p; 369-3p; 411-3p; 502-3p; 376b-3p; 21-5p; 136-5p; 340-5p; 23b-3p; 145-5p; 370-3p; 126-3p; 323b-3p
41	urinary bladder cancer	2.34E-50	88	106b-3p; 17-5p; 182-5p; 20a-5p; 155-5p; 96-5p; 19a-3p; hsa-let-7c-5p; 142-5p; 183-5p; 142-3p; 744-5p; 93-5p; 200c-3p; 19b-3p; 497-5p; 146a-5p; 106b-5p; 15b-5p; 130b-3p; 25-3p; 210-3p; 9-5p; 425-5p; 16-5p; 125b-2-3p; 154-5p; 192-5p; 381-3p; 376a-3p; 376c-3p; 124-3p; 30a-5p; 186-5p; 299-5p; 31-5p; 451a; 195-5p; 203a-3p; 379-5p; 143-3p; hsa-let-7a-5p; 218-5p; 135a-5p; 487b-3p; 148a-3p; 21-3p; 129-5p; 30c-5p; 30b-5p; 99a-5p; 144-3p; 654-3p; 132-3p; 130a-3p; 134-5p; 495-3p; 15a-5p; 194-5p; 26a-5p; 139-5p; 423-3p; 320c; 1-3p; 181d-5p; 432-5p; 7-5p; 127-3p; 532-3p; 409-3p; 27b-3p; 340-3p; 204-5p; 27a-3p; 494-3p; 502-3p; 139-3p; 21-5p; 20b-5p; 136-5p; 30a-3p; 23b-3p; 28-3p; 145-5p; 125b-5p; 221-5p; 99b-5p; 126-3p
42	**B-cell lymphoma**	3.04E-48	103	92a-3p; 106b-3p; 17-5p; 182-5p; 20a-5p; 155-5p; 96-5p; 18a-5p; 19a-3p; hsa-let-7c-5p; 17-3p; 142-5p; 183-5p; hsa-let-7e-5p; 142-3p; 1260a; 93-5p; 200c-3p; 19b-3p; 582-5p; 146a-5p; 181c-5p; 106b-5p; 15b-5p; 130b-3p; 25-3p; 210-3p; 9-5p; 486-5p; 16-5p; 192-5p; 30d-5p; 376a-3p; 376c-3p; 124-3p; 30a-5p; 186-5p; 9-3p; 31-5p; 451a; 191-5p; 125a-5p; 195-5p; 203a-3p; 143-3p; hsa-let-7a-5p; 218-5p; 148a-3p; 129-5p; 29b-1-5p; 30c-5p; 30b-5p; 99a-5p; 144-3p; 130a-3p; 134-5p; 98-5p; 15a-5p; 95-3p; 551b-3p; 194-5p; 26a-5p; 296-5p; 106a-5p; 139-5p; 323a-3p; 423-3p; 320c; 1-3p; 18b-5p; 378a-3p; 432-5p; 7-5p; 320d; 138-5p; 127-3p; 532-3p; 187-3p; 339-5p; 27b-3p; 204-5p; 27a-3p; 494-3p; 369-3p; 502-3p; 32-5p; hsa-let-7b-5p; 21-5p; 20b-5p; 136-5p; 30a-3p; 153-3p; 338-5p; 454-3p; 23b-3p; 211-5p; 145-5p; 504-5p; 125b-5p; 221-5p; 99b-5p; 126-3p; 770-5p
43	malignant glioma	1.16E-46	166	92a-3p; 106b-3p; 17-5p; 182-5p; 20a-5p; 155-5p; 96-5p; 18a-5p; 1246; 19a-3p; hsa-let-7c-5p; 17-3p; 142-5p; 183-5p; hsa-let-7e-5p; 142-3p; 1260b; 1260a; 744-5p; 93-5p; 200c-3p; 19b-3p; 582-5p; 497-5p; 146a-5p; 766-3p; 181c-5p; 106b-5p; 15b-5p; 130b-3p; 25-3p; 210-3p; 9-5p; 486-5p; 425-5p; 16-5p; 154-5p; 192-5p; 30d-5p; 381-3p; 376a-3p; 376c-3p; 628-3p; 1285-3p; 124-3p; 30a-5p; 410-3p; 186-5p; 9-3p; 299-5p; 31-5p; 655-3p; 451a; 539-5p; 769-5p; 505-3p; 191-5p; 154-3p; 491-5p; 125a-5p; 181c-3p; 195-5p; 203a-3p; 369-5p; 379-5p; 128-3p; 485-5p; 143-3p; hsa-let-7a-5p; 218-5p; 4286; 135a-5p; 423-5p; 543; 485-3p; 487b-3p; 148a-3p; 21-3p; 129-5p; 29b-1-5p; 129-1-3p; 656-3p; 30c-5p; 1248; 30b-5p; 99a-5p; 380-3p; 144-3p; 31-3p; 329-3p; 132-3p; 484; 130a-3p; 134-5p; 98-5p; 149-5p; 625-5p; 409-5p; 758-3p; 495-3p; 15a-5p; 376a-5p; 487a-3p; 95-3p; 551b-3p; 194-5p; 411-5p; 433-3p; 129-2-3p; 26a-5p; 296-5p; 106a-5p; 139-5p; 323a-3p; 423-3p; 425-3p; 320c; 1-3p; 33b-5p; 181d-5p; 18b-5p; 378a-3p; 629-5p; 590-5p; 487a-5p; 432-5p; 7-5p; 138-5p; 127-3p; 187-3p; 409-3p; 339-5p; 382-5p; 885-5p; 27b-3p; 204-5p; 27a-3p; 383-5p; 769-3p; 494-3p; 369-3p; 376b-3p; 32-5p; hsa-let-7b-5p; 21-5p; 590-3p; 20b-5p; 136-5p; 30a-3p; 340-5p; 153-3p; 338-5p; 454-3p; 23b-3p; 28-3p; 211-5p; 145-5p; 370-3p; 504-5p; 125b-5p; 221-5p; 99b-5p; 126-3p; 92b-3p; 770-5p; 1296-5p

*p-value after the Benjamini-Hochberg adjustment
