# Supplementary material for: A Set of 17 microRNAs Common for Brain and Cerebrospinal Fluid Differentiates Primary Central Nervous System Lymphoma from Non-Malignant Brain Tumors
Source: Biomolecules. 2021 Sep 21;11(9):1395. doi: 10.3390/biom11091395 (PMC8466839; doi:10.3390/biom11091395)
Supplement: Supplementary file 1 [file biomolecules-11-01395-s001.zip › Table S14.pdf]

Supplementary Table S14

Forms and sources of CNS DLBCL-specific tumor microRNAs.

		Extracellular RNA forms (miRandola)		
**No**	**subcategory**	**q-value***	**number of miRs observed**	**hsa-miRs**
1	circulating	1.2505E-05	150	92a-3p; 17-5p; 182-5p; 20a-5p; 155-5p; 18a-5p; 1246; 19a-3p; hsa-let-7c-5p; 17-3p; 1290; 142-5p; 183-5p; 15b-3p; hsa-let-7e-5p; 142-3p; 1260a; 92a-1-5p; 744-5p; 16-2-3p; 93-5p; 200c-3p; 19b-3p; 497-5p; 146a-5p; 766-3p; 181c-5p; 106b-5p; 15b-5p; 130b-3p; 25-3p; 210-3p; 9-5p; 486-5p; 425-5p; 16-5p; 125b-2-3p; 154-5p; 192-5p; 30d-5p; 381-3p; 376a-3p; 376c-3p; 628-3p; 1285-3p; 124-3p; 30a-5p; 410-3p; 486-3p; 186-5p; 9-3p; 299-5p; 31-5p; 625-3p; 451a; 505-3p; 191-5p; 491-5p; 125a-5p; 195-5p; 203a-3p; 379-5p; 128-3p; 485-5p; 143-3p; hsa-let-7a-5p; 218-5p; 4286; 423-5p; 543; 485-3p; 487b-3p; 148a-3p; 21-3p; 29b-1-5p; 129-1-3p; 30c-5p; 1248; 30b-5p; 99a-5p; 628-5p; 144-3p; 654-3p; 132-3p; 484; 130a-3p; 134-5p; 98-5p; 149-5p; 625-5p; 495-3p; 15a-5p; 95-3p; 551b-3p; 361-3p; 194-5p; 411-5p; 433-3p; 26a-5p; 296-5p; 106a-5p; 139-5p; 323a-3p; 423-3p; 425-3p; 320c; 1-3p; 335-3p; 144-5p; 33b-5p; 181d-5p; 18b-5p; 378a-3p; 629-5p; 590-5p; 432-5p; 7-5p; 138-5p; 127-3p; 532-3p; 409-3p; 339-5p; 382-5p; 885-5p; 27b-3p; 340-3p; 204-5p; 27a-3p; 383-5p; 769-3p; 494-3p; 369-3p; 376b-3p; 139-3p; 32-5p; hsa-let-7b-5p; 21-5p; 20b-5p; 136-5p; 340-5p; 454-3p; 23b-3p; 28-3p; 145-5p; 125b-5p; 92b-5p; 99b-5p; 126-3p; 92b-3p; 1249-3p
2	exosome	0.0060744	140	92a-3p; 17-5p; 182-5p; 20a-5p; 155-5p; 96-5p; 18a-5p; 1246; 19a-3p; 19b-1-5p; hsa-let-7c-5p; 17-3p; 1290; 142-5p; 183-5p; 15b-3p; hsa-let-7e-5p; 142-3p; 744-5p; 16-2-3p; 93-5p; 200c-3p; 19b-3p; 582-5p; 146a-5p; 766-3p; 181c-5p; 106b-5p; 15b-5p; 130b-3p; 25-3p; 210-3p; 9-5p; 486-5p; 425-5p; 16-5p; 125b-2-3p; 192-5p; 30d-5p; 376a-3p; 628-3p; 124-3p; 30a-5p; 186-5p; 9-3p; 31-5p; 625-3p; 451a; 769-5p; 505-3p; 191-5p; 491-5p; 125a-5p; 181c-3p; 195-5p; 203a-3p; 379-5p; 128-3p; 485-5p; hsa-let-7a-5p; 218-5p; 135a-5p; 423-5p; 487b-3p; 148a-3p; 21-3p; 129-5p; 378c; 30c-5p; 30b-5p; 99a-5p; 628-5p; 31-3p; 93-3p; 132-3p; 484; 130a-3p; 134-5p; 98-5p; 149-5p; 625-5p; 15a-5p; 95-3p; 551b-3p; 361-3p; 194-5p; 433-3p; 26a-5p; 296-5p; 106a-5p; 139-5p; 423-3p; 425-3p; 320c; 132-5p; 3065-5p; 33b-5p; 181d-5p; 18b-5p; 378a-3p; 629-5p; 590-5p; 432-5p; 7-5p; 320d; 138-5p; 127-3p; 532-3p; 187-3p; 339-5p; 382-5p; 885-5p; 27b-3p; 340-3p; 27a-3p; 769-3p; 494-3p; 369-3p; 502-3p; 139-3p; 32-5p; hsa-let-7b-5p; 21-5p; 20b-5p; 136-5p; 30a-3p; 340-5p; 153-3p; 99b-3p; 454-3p; 23b-3p; 28-3p; 145-5p; 370-3p; 125b-5p; 221-5p; 99b-5p; 126-3p; 92b-3p; 770-5p
3	microparticle	0.0060744	6	92a-3p; 106b-3p; 19b-3p; 25-3p; 143-3p; 21-5p
4	microvesicle	0.0060744	40	92a-3p; 17-5p; 20a-5p; hsa-let-7c-5p; 17-3p; 142-5p; 142-3p; 93-5p; 200c-3p; 19b-3p; 181c-5p; 15b-5p; 9-5p; 486-5p; 16-5p; 30d-5p; 410-3p; 31-5p; 451a; 191-5p; 125a-5p; hsa-let-7a-5p; 21-3p; 99a-5p; 26a-5p; 106a-5p; 323a-3p; 423-3p; 1-3p; 432-5p; 382-5p; 204-5p; 27a-3p; 383-5p; hsa-let-7b-5p; 21-5p; 20b-5p; 23b-3p; 145-5p; 125b-5p
5	Ago2	0.0061846	53	92a-3p; 17-5p; 20a-5p; 18a-5p; 19a-3p; 19b-1-5p; 142-5p; 93-5p; 200c-3p; 19b-3p; 146a-5p; 766-3p; 130b-3p; 25-3p; 99a-3p; 210-3p; 486-5p; 425-5p; 16-5p; 192-5p; 30d-5p; 376a-3p; 376c-3p; 30a-5p; 186-5p; 9-3p; 451a; 143-3p; 423-5p; 485-3p; 26b-3p; 148a-3p; 99a-5p; 144-3p; 654-3p; 93-3p; 130a-3p; 15a-5p; 194-5p; 296-5p; 106a-5p; 425-3p; 18b-5p; 378a-3p; 7-5p; 27b-3p; 204-5p; 27a-3p; hsa-let-7b-5p; 21-5p; 20b-5p; 340-5p; 770-5p

Expressed in tissue (Tissue Atlas)				
**No**	**subcategory**	**q-value**	**number of miRs observed**	**hsa-miRs**
**1**	brain	1.3414E-09	171	4488; 92a-3p; 17-5p; 20a-5p; 155-5p; 96-5p; 1246; 19a-3p; hsa-let-7c-5p; 17-3p; 1290; 3960; 142-5p; hsa-let-7e-5p; 142-3p; 1260b; 1260a; 744-5p; 873-5p; 93-5p; 19b-3p; 582-5p; 497-5p; 146a-5p; 766-3p; 181c-5p; 106b-5p; 15b-5p; 4449; 130b-3p; 25-3p; 210-3p; 9-5p; 486-5p; 425-5p; 16-5p; 125b-2-3p; 154-5p; 192-5p; 30d-5p; 381-3p; 1185-5p; 376a-3p; 376c-3p; 628-3p; 1285-3p; 124-3p; 30a-5p; 410-3p; 186-5p; 9-3p; 127-5p; 299-5p; 31-5p; 625-3p; 451a; 539-5p; 769-5p; 505-3p; 136-3p; 154-3p; 491-5p; 125a-5p; 181c-3p; 195-5p; 203a-3p; 369-5p; 379-5p; 128-3p; 485-5p; 143-3p; hsa-let-7a-5p; 218-5p; 4286; 135a-5p; 423-5p; 543; 485-3p; 487b-3p; 148a-3p; 129-5p; 129-1-3p; 656-3p; 378c; 30c-5p; 30b-5p; 99a-5p; 628-5p; 144-3p; 654-3p; 101-5p; 31-3p; 329-3p; 132-3p; 484; 130a-3p; 134-5p; 98-5p; 149-5p; 625-5p; 378i; 409-5p; 758-3p; 495-3p; 15a-5p; 376a-5p; 487a-3p; 95-3p; 551b-3p; 361-3p; 194-5p; 411-5p; 433-3p; 129-2-3p; 26a-5p; 296-5p; 139-5p; 323a-3p; 423-3p; 425-3p; 320c; 1-3p; 132-5p; 3065-5p; 335-3p; 181d-5p; 378a-3p; 590-5p; 432-5p; 7-5p; 124-5p; 320d; 138-5p; 127-3p; 532-3p; 409-3p; 382-5p; 885-5p; 27b-3p; 340-3p; 204-5p; 27a-3p; 383-5p; 769-3p; 494-3p; 369-3p; 502-3p; 376b-3p; 139-3p; 32-5p; hsa-let-7b-5p; 21-5p; 20b-5p; 136-5p; 30a-3p; 340-5p; 153-3p; 99b-3p; 338-5p; 454-3p; 23b-3p; 211-5p; 145-5p; 370-3p; 125b-5p; 99b-5p; 126-3p; 92b-3p; 770-5p; 1296-5p; 1249-3p
**2**	spinal cord	1.3414E-09	170	4488; 92a-3p; 17-5p; 20a-5p; 155-5p; 96-5p; 18a-5p; 1246; 19a-3p; hsa-let-7c-5p; 17-3p; 1290; 3960; 142-5p; 183-5p; hsa-let-7e-5p; 142-3p; 1260b; 1260a; 744-5p; 873-5p; 93-5p; 19b-3p; 582-5p; 497-5p; 146a-5p; 766-3p; 181c-5p; 106b-5p; 15b-5p; 4449; 130b-3p; 25-3p; 3615; 99a-3p; 210-3p; 9-5p; 486-5p; 425-5p; 16-5p; 125b-2-3p; 154-5p; 192-5p; 30d-5p; 381-3p; 1185-5p; 376a-3p; 376c-3p; 628-3p; 1285-3p; 124-3p; 30a-5p; 410-3p; 186-5p; 9-3p; 127-5p; 299-5p; 31-5p; 625-3p; 451a; 539-5p; 769-5p; 505-3p; 136-3p; 154-3p; 125a-5p; 181c-3p; 195-5p; 369-5p; 379-5p; 128-3p; 143-3p; hsa-let-7a-5p; 218-5p; 4286; 135a-5p; 423-5p; 543; 485-3p; 487b-3p; 148a-3p; 21-3p; 129-5p; 129-1-3p; 656-3p; 378c; 30c-5p; 30b-5p; 99a-5p; 628-5p; 144-3p; 654-3p; 101-5p; 31-3p; 329-3p; 132-3p; 484; 130a-3p; 134-5p; 98-5p; 149-5p; 625-5p; 378i; 758-3p; 495-3p; 15a-5p; 376a-5p; 487a-3p; 95-3p; 551b-3p; 361-3p; 194-5p; 411-5p; 129-2-3p; 26a-5p; 296-5p; 139-5p; 323a-3p; 423-3p; 425-3p; 320c; 1-3p; 132-5p; 144-5p; 181d-5p; 378a-3p; 590-5p; 432-5p; 7-5p; 124-5p; 320d; 138-5p; 127-3p; 532-3p; 409-3p; 339-5p; 382-5p; 885-5p; 27b-3p; 340-3p; 204-5p; 27a-3p; 383-5p; 769-3p; 494-3p; 502-3p; 376b-3p; 139-3p; 32-5p; hsa-let-7b-5p; 21-5p; 20b-5p; 136-5p; 30a-3p; 340-5p; 378d; 153-3p; 99b-3p; 338-5p; 454-3p; 23b-3p; 211-5p; 145-5p; 370-3p; 125b-5p; 99b-5p; 126-3p; 770-5p; 1296-5p; 1249-3p
3	epididymis	0.00011923	158	4488; 92a-3p; 17-5p; 182-5p; 20a-5p; 155-5p; 96-5p; 1246; 19a-3p; hsa-let-7c-5p; 17-3p; 1290; 3960; 142-5p; 183-5p; hsa-let-7e-5p; 142-3p; 1260b; 1260a; 16-2-3p; 93-5p; 200c-3p; 19b-3p; 582-5p; 497-5p; 146a-5p; 766-3p; 181c-5p; 106b-5p; 15b-5p; 4449; 130b-3p; 25-3p; 210-3p; 486-5p; 425-5p; 16-5p; 125b-2-3p; 154-5p; 192-5p; 30d-5p; 381-3p; 1185-5p; 376a-3p; 376c-3p; 1285-3p; 30a-5p; 410-3p; 186-5p; 9-3p; 299-5p; 31-5p; 625-3p; 451a; 539-5p; 769-5p; 505-3p; 136-3p; 125a-5p; 195-5p; 203a-3p; 369-5p; 379-5p; 128-3p; 143-3p; hsa-let-7a-5p; 218-5p; 4286; 135a-5p; 423-5p; 543; 487b-3p; 148a-3p; 129-5p; 29b-1-5p; 129-1-3p; 656-3p; 378c; 30c-5p; 30b-5p; 99a-5p; 628-5p; 144-3p; 654-3p; 101-5p; 31-3p; 329-3p; 132-3p; 484; 130a-3p; 134-5p; 98-5p; 149-5p; 625-5p; 378i; 758-3p; 495-3p; 15a-5p; 376a-5p; 95-3p; 551b-3p; 361-3p; 194-5p; 411-5p; 129-2-3p; 26a-5p; 296-5p; 139-5p; 423-3p; 425-3p; 320c; 1-3p; 132-5p; 335-3p; 144-5p; 181d-5p; 18b-5p; 378a-3p; 590-5p; 432-5p; 7-5p; 320d; 127-3p; 532-3p; 409-3p; 382-5p; 885-5p; 27b-3p; 340-3p; 204-5p; 27a-3p; 769-3p; 494-3p; 369-3p; 502-3p; 376b-3p; 139-3p; 32-5p; hsa-let-7b-5p; 21-5p; 20b-5p; 136-5p; 30a-3p; 340-5p; 378d; 99b-3p; 454-3p; 23b-3p; 211-5p; 145-5p; 370-3p; 125b-5p; 221-5p; 99b-5p; 126-3p; 92b-3p; 770-5p; 1249-3p

4	dura mater	0.00048845	154	4488; 92a-3p; 17-5p; 20a-5p; 155-5p; 96-5p; 1246; 19a-3p; hsa-let-7c-5p; 17-3p; 1290; 3960; 142-5p; hsa-let-7e-5p; 142-3p; 1260b; 1260a; 16-2-3p; 873-5p; 93-5p; 19b-3p; 582-5p; 497-5p; 146a-5p; 766-3p; 181c-5p; 106b-5p; 15b-5p; 4449; 130b-3p; 25-3p; 210-3p; 9-5p; 486-5p; 425-5p; 16-5p; 125b-2-3p; 154-5p; 192-5p; 30d-5p; 381-3p; 376a-3p; 376c-3p; 1285-3p; 124-3p; 30a-5p; 410-3p; 186-5p; 9-3p; 299-5p; 625-3p; 451a; 539-5p; 769-5p; 505-3p; 136-3p; 154-3p; 125a-5p; 181c-3p; 195-5p; 369-5p; 379-5p; 128-3p; 143-3p; hsa-let-7a-5p; 218-5p; 4286; 135a-5p; 423-5p; 543; 485-3p; 487b-3p; 148a-3p; 21-3p; 129-5p; 29b-1-5p; 129-1-3p; 656-3p; 378c; 30c-5p; 30b-5p; 99a-5p; 144-3p; 654-3p; 101-5p; 329-3p; 132-3p; 484; 130a-3p; 134-5p; 98-5p; 149-5p; 625-5p; 378i; 495-3p; 15a-5p; 376a-5p; 95-3p; 551b-3p; 361-3p; 194-5p; 129-2-3p; 26a-5p; 296-5p; 139-5p; 323a-3p; 423-3p; 425-3p; 320c; 1-3p; 132-5p; 335-3p; 181d-5p; 378a-3p; 590-5p; 432-5p; 7-5p; 320d; 138-5p; 127-3p; 409-3p; 382-5p; 885-5p; 27b-3p; 340-3p; 204-5p; 27a-3p; 383-5p; 769-3p; 494-3p; 369-3p; 502-3p; 139-3p; hsa-let-7b-5p; 21-5p; 20b-5p; 136-5p; 30a-3p; 340-5p; 378d; 153-3p; 99b-3p; 338-5p; 23b-3p; 28-3p; 211-5p; 145-5p; 370-3p; 125b-5p; 99b-5p; 126-3p; 92b-3p; 770-5p; 1249-3p
5	skin	0.0004892	160	4488; 92a-3p; 17-5p; 20a-5p; 155-5p; 96-5p; 18a-5p; 1246; 19a-3p; hsa-let-7c-5p; 17-3p; 1290; 3960; 142-5p; 183-5p; 15b-3p; hsa-let-7e-5p; 142-3p; 1260b; 1260a; 16-2-3p; 93-5p; 200c-3p; 19b-3p; 582-5p; 130b-5p; 497-5p; 146a-5p; 766-3p; 181c-5p; 106b-5p; 15b-5p; 130b-3p; 25-3p; 210-3p; 9-5p; 486-5p; 425-5p; 16-5p; 125b-2-3p; 154-5p; 192-5p; 30d-5p; 381-3p; 1185-5p; 376a-3p; 376c-3p; 1285-3p; 124-3p; 30a-5p; 410-3p; 186-5p; 9-3p; 299-5p; 625-3p; 451a; 539-5p; 769-5p; 505-3p; 136-3p; 154-3p; 125a-5p; 195-5p; 203a-3p; 369-5p; 379-5p; 128-3p; 143-3p; hsa-let-7a-5p; 218-5p; 4286; 135a-5p; 423-5p; 485-3p; 26b-3p; 487b-3p; 148a-3p; 21-3p; 129-5p; 29b-1-5p; 129-1-3p; 656-3p; 378c; 30c-5p; 30b-5p; 99a-5p; 144-3p; 654-3p; 101-5p; 329-3p; 132-3p; 484; 130a-3p; 3613-5p; 134-5p; 98-5p; 149-5p; 625-5p; 378i; 495-3p; 15a-5p; 376a-5p; 95-3p; 551b-3p; 361-3p; 194-5p; 129-2-3p; 26a-5p; 296-5p; 139-5p; 423-3p; 425-3p; 320c; 1-3p; 132-5p; 335-3p; 181d-5p; 378a-3p; 590-5p; 432-5p; 7-5p; 320d; 127-3p; 532-3p; 409-3p; 382-5p; 885-5p; 27b-3p; 340-3p; 204-5p; 27a-3p; 383-5p; 769-3p; 494-3p; 369-3p; 502-3p; 139-3p; 32-5p; hsa-let-7b-5p; 21-5p; 20b-5p; 136-5p; 30a-3p; 340-5p; 378d; 99b-3p; 338-5p; 454-3p; 23b-3p; 28-3p; 211-5p; 145-5p; 370-3p; 125b-5p; 221-5p; 99b-5p; 126-3p; 770-5p; 1296-5p; 1249-3p
6	thyroid	0.00054428	158	4488; 92a-3p; 17-5p; 20a-5p; 155-5p; 96-5p; 18a-5p; 1246; 19a-3p; hsa-let-7c-5p; 17-3p; 1290; 3960; 142-5p; 183-5p; 15b-3p; hsa-let-7e-5p; 142-3p; 1260b; 1260a; 16-2-3p; 873-5p; 93-5p; 200c-3p; 19b-3p; 582-5p; 130b-5p; 497-5p; 146a-5p; 766-3p; 181c-5p; 106b-5p; 15b-5p; 4449; 130b-3p; 25-3p; 210-3p; 9-5p; 486-5p; 425-5p; 16-5p; 125b-2-3p; 154-5p; 192-5p; 30d-5p; 381-3p; 1185-5p; 376a-3p; 376c-3p; 1285-3p; 124-3p; 30a-5p; 186-5p; 9-3p; 299-5p; 31-5p; 625-3p; 451a; 539-5p; 769-5p; 505-3p; 136-3p; 125a-5p; 195-5p; 203a-3p; 369-5p; 128-3p; 143-3p; hsa-let-7a-5p; 218-5p; 4286; 135a-5p; 423-5p; 543; 485-3p; 26b-3p; 148a-3p; 21-3p; 129-5p; 29b-1-5p; 129-1-3p; 378c; 30c-5p; 30b-5p; 99a-5p; 628-5p; 144-3p; 654-3p; 101-5p; 31-3p; 329-3p; 132-3p; 484; 130a-3p; 3613-5p; 134-5p; 98-5p; 149-5p; 625-5p; 378i; 495-3p; 15a-5p; 376a-5p; 95-3p; 551b-3p; 361-3p; 194-5p; 129-2-3p; 26a-5p; 296-5p; 139-5p; 425-3p; 320c; 1-3p; 132-5p; 335-3p; 144-5p; 181d-5p; 18b-5p; 378a-3p; 590-5p; 432-5p; 7-5p; 320d; 138-5p; 127-3p; 532-3p; 409-3p; 885-5p; 27b-3p; 340-3p; 204-5p; 27a-3p; 769-3p; 494-3p; 502-3p; 139-3p; 32-5p; hsa-let-7b-5p; 21-5p; 20b-5p; 136-5p; 30a-3p; 340-5p; 378d; 99b-3p; 338-5p; 454-3p; 23b-3p; 211-5p; 145-5p; 370-3p; 125b-5p; 221-5p; 99b-5p; 126-3p; 1296-5p; 1249-3p

7	liver	0.0108343	124	4488; 92a-3p; 17-5p; 20a-5p; 155-5p; 1246; 19a-3p; hsa-let-7c-5p; 17-3p; 1290; 3960; 142-5p; 15b-3p; 142-3p; 1260b; 1260a; 16-2-3p; 93-5p; 19b-3p; 130b-5p; 497-5p; 146a-5p; 766-3p; 106b-5p; 15b-5p; 4449; 130b-3p; 25-3p; 3615; 210-3p; 486-5p; 425-5p; 16-5p; 125b-2-3p; 154-5p; 192-5p; 30d-5p; 381-3p; 376a-3p; 376c-3p; 1285-3p; 30a-5p; 186-5p; 299-5p; 625-3p; 451a; 539-5p; 769-5p; 505-3p; 125a-5p; 195-5p; 128-3p; 143-3p; hsa-let-7a-5p; 4286; 135a-5p; 423-5p; 485-3p; 148a-3p; 21-3p; 129-5p; 29b-1-5p; 129-1-3p; 656-3p; 30c-5p; 30b-5p; 99a-5p; 144-3p; 654-3p; 329-3p; 484; 130a-3p; 3613-5p; 134-5p; 149-5p; 378i; 15a-5p; 361-3p; 194-5p; 129-2-3p; 26a-5p; 296-5p; 139-5p; 423-3p; 425-3p; 320c; 335-3p; 181d-5p; 378a-3p; 590-5p; 432-5p; 320d; 127-3p; 409-3p; 382-5p; 885-5p; 27b-3p; 340-3p; 204-5p; 27a-3p; 383-5p; 769-3p; 494-3p; 139-3p; hsa-let-7b-5p; 21-5p; 20b-5p; 136-5p; 30a-3p; 340-5p; 378d; 99b-3p; 338-5p; 23b-3p; 28-3p; 211-5p; 145-5p; 370-3p; 125b-5p; 126-3p; 92b-3p; 770-5p; 1296-5p; 1249-3p
8	lymph node	0.0108343	115	4488; 92a-3p; 17-5p; 20a-5p; 155-5p; 96-5p; 1246; 19a-3p; hsa-let-7c-5p; 1290; 3960; 142-5p; hsa-let-7e-5p; 142-3p; 1260b; 1260a; 93-5p; 200c-3p; 19b-3p; 497-5p; 146a-5p; 766-3p; 106b-5p; 15b-5p; 4449; 130b-3p; 25-3p; 3615; 210-3p; 486-5p; 425-5p; 16-5p; 125b-2-3p; 192-5p; 30d-5p; 381-3p; 628-3p; 1285-3p; 30a-5p; 186-5p; 9-3p; 31-5p; 625-3p; 451a; 539-5p; 491-5p; 125a-5p; 195-5p; 203a-3p; 143-3p; hsa-let-7a-5p; 4286; 423-5p; 485-3p; 148a-3p; 21-3p; 129-5p; 29b-1-5p; 129-1-3p; 378c; 30c-5p; 30b-5p; 99a-5p; 628-5p; 144-3p; 484; 130a-3p; 134-5p; 149-5p; 378i; 15a-5p; 95-3p; 361-3p; 194-5p; 129-2-3p; 26a-5p; 296-5p; 423-3p; 425-3p; 320c; 335-3p; 181d-5p; 378a-3p; 590-5p; 432-5p; 320d; 138-5p; 127-3p; 409-3p; 382-5p; 885-5p; 27b-3p; 204-5p; 27a-3p; 383-5p; 769-3p; 494-3p; 139-3p; hsa-let-7b-5p; 21-5p; 20b-5p; 136-5p; 99b-3p; 338-5p; 23b-3p; 211-5p; 145-5p; 370-3p; 125b-5p; 92b-5p; 126-3p; 92b-3p; 770-5p; 1296-5p; 1249-3p
9	tunica albuginea	0.0108343	150	4488; 92a-3p; 17-5p; 20a-5p; 155-5p; 96-5p; 1246; 19a-3p; hsa-let-7c-5p; 17-3p; 1290; 3960; 142-5p; 15b-3p; hsa-let-7e-5p; 142-3p; 1260b; 1260a; 16-2-3p; 873-5p; 93-5p; 200c-3p; 19b-3p; 582-5p; 130b-5p; 497-5p; 146a-5p; 766-3p; 181c-5p; 106b-5p; 15b-5p; 130b-3p; 25-3p; 210-3p; 486-5p; 425-5p; 16-5p; 125b-2-3p; 154-5p; 192-5p; 30d-5p; 381-3p; 1185-5p; 376a-3p; 376c-3p; 1285-3p; 30a-5p; 410-3p; 186-5p; 299-5p; 625-3p; 655-3p; 451a; 539-5p; 769-5p; 505-3p; 136-3p; 154-3p; 125a-5p; 195-5p; 369-5p; 379-5p; 128-3p; 143-3p; hsa-let-7a-5p; 218-5p; 4286; 135a-5p; 423-5p; 543; 485-3p; 26b-3p; 487b-3p; 148a-3p; 21-3p; 129-5p; 29b-1-5p; 129-1-3p; 30c-5p; 30b-5p; 99a-5p; 628-5p; 144-3p; 654-3p; 101-5p; 329-3p; 132-3p; 484; 130a-3p; 3613-5p; 134-5p; 98-5p; 149-5p; 378i; 409-5p; 758-3p; 495-3p; 15a-5p; 376a-5p; 551b-3p; 361-3p; 194-5p; 129-2-3p; 26a-5p; 296-5p; 139-5p; 425-3p; 320c; 1-3p; 132-5p; 335-3p; 144-5p; 33b-5p; 181d-5p; 378a-3p; 590-5p; 432-5p; 7-5p; 320d; 127-3p; 532-3p; 409-3p; 382-5p; 885-5p; 27b-3p; 340-3p; 204-5p; 27a-3p; 383-5p; 769-3p; 494-3p; 369-3p; 502-3p; 376b-3p; 139-3p; hsa-let-7b-5p; 21-5p; 20b-5p; 136-5p; 30a-3p; 340-5p; 378d; 23b-3p; 28-3p; 211-5p; 145-5p; 125b-5p; 99b-5p; 126-3p; 1249-3p
10	muscle	0.0166504	162	92a-3p; 17-5p; 20a-5p; 155-5p; 96-5p; 1246; 19a-3p; hsa-let-7c-5p; 17-3p; 1290; 3960; 142-5p; 15b-3p; hsa-let-7e-5p; 142-3p; 1260b; 1260a; 16-2-3p; 93-5p; 19b-3p; 582-5p; 130b-5p; 497-5p; 146a-5p; 766-3p; 181c-5p; 106b-5p; 15b-5p; 4449; 130b-3p; 25-3p; 210-3p; 9-5p; 486-5p; 425-5p; 16-5p; 125b-2-3p; 154-5p; 192-5p; 30d-5p; 381-3p; 1185-5p; 376a-3p; 376c-3p; 628-3p; 1285-3p; 30a-5p; 410-3p; 486-3p; 186-5p; 299-5p; 625-3p; 655-3p; 451a; 539-5p; 769-5p; 505-3p; 136-3p; 154-3p; 125a-5p; 195-5p; 369-5p; 379-5p; 128-3p; 143-3p; hsa-let-7a-5p; 218-5p; 4286; 135a-5p; 423-5p; 543; 485-3p; 26b-3p; 487b-3p; 148a-3p; 21-3p; 29b-1-5p; 129-1-3p; 656-3p; 378c; 30c-5p; 1248; 30b-5p; 99a-5p; 628-5p; 144-3p; 654-3p; 101-5p; 329-3p; 132-3p; 484; 130a-3p; 3613-5p; 134-5p; 98-5p; 149-5p; 625-5p; 378i; 495-3p; 15a-5p; 376a-5p; 95-3p; 551b-3p; 361-3p; 194-5p; 411-5p; 129-2-3p; 26a-5p; 296-5p; 139-5p; 423-3p; 425-3p; 320c; 1-3p; 132-5p; 335-3p; 144-5p; 33b-5p; 181d-5p; 18b-5p; 378a-3p; 590-5p; 432-5p; 7-5p; 320d; 127-3p; 532-3p; 409-3p; 382-5p; 885-5p; 27b-3p; 340-3p; 204-5p; 27a-3p; 383-5p; 769-3p; 494-3p; 369-3p; 502-3p; 139-3p; 32-5p; hsa-let-7b-5p; 21-5p; 20b-5p; 136-5p; 30a-3p; 340-5p; 378d; 99b-3p; 454-3p; 23b-3p; 211-5p; 145-5p; 370-3p; 125b-5p; 221-5p; 99b-5p; 126-3p; 92b-3p; 770-5p; 1296-5p; 1249-3p

11	colon	0.0404429	147	4488; 92a-3p; 17-5p; 20a-5p; 155-5p; 96-5p; 18a-5p; 1246; 19a-3p; hsa-let-7c-5p; 17-3p; 1290; 3960; 142-5p; hsa-let-7e-5p; 142-3p; 1260b; 1260a; 16-2-3p; 93-5p; 200c-3p; 19b-3p; 582-5p; 497-5p; 146a-5p; 766-3p; 181c-5p; 106b-5p; 15b-5p; 4449; 130b-3p; 25-3p; 210-3p; 9-5p; 486-5p; 425-5p; 16-5p; 125b-2-3p; 154-5p; 192-5p; 30d-5p; 381-3p; 376a-3p; 376c-3p; 628-3p; 1285-3p; 124-3p; 30a-5p; 410-3p; 186-5p; 9-3p; 299-5p; 31-5p; 625-3p; 451a; 539-5p; 769-5p; 505-3p; 136-3p; 125a-5p; 195-5p; 203a-3p; 128-3p; 143-3p; hsa-let-7a-5p; 218-5p; 4286; 423-5p; 487b-3p; 148a-3p; 21-3p; 129-5p; 29b-1-5p; 129-1-3p; 378c; 30c-5p; 30b-5p; 99a-5p; 144-3p; 654-3p; 101-5p; 132-3p; 484; 130a-3p; 134-5p; 98-5p; 149-5p; 625-5p; 378i; 495-3p; 15a-5p; 95-3p; 551b-3p; 361-3p; 194-5p; 129-2-3p; 26a-5p; 296-5p; 139-5p; 423-3p; 425-3p; 320c; 1-3p; 132-5p; 335-3p; 181d-5p; 18b-5p; 378a-3p; 590-5p; 432-5p; 7-5p; 320d; 127-3p; 532-3p; 409-3p; 382-5p; 885-5p; 27b-3p; 204-5p; 27a-3p; 383-5p; 769-3p; 494-3p; 502-3p; 139-3p; 32-5p; hsa-let-7b-5p; 21-5p; 20b-5p; 136-5p; 30a-3p; 340-5p; 378d; 99b-3p; 338-5p; 454-3p; 23b-3p; 28-3p; 211-5p; 145-5p; 370-3p; 125b-5p; 99b-5p; 126-3p; 92b-3p; 770-5p; 1249-3p

*p-value after the Benjamini-Hochberg adjustment
