# Supplementary material for: A Set of 17 microRNAs Common for Brain and Cerebrospinal Fluid Differentiates Primary Central Nervous System Lymphoma from Non-Malignant Brain Tumors
Source: Biomolecules. 2021 Sep 21;11(9):1395. doi: 10.3390/biom11091395 (PMC8466839; doi:10.3390/biom11091395)
Supplement: Supplementary file 1 [file biomolecules-11-01395-s001.zip › Table S15.pdf]

Supplementary Table S15

Molecular functions of CNS DLBCL-specific tumor microRNAs.

GO Annotations indirect				
**No**	**subcategory**	**q-value***	**number of miRs observed**	**hsa-miRs**
1	negative regulation of anoikis	2.19534E-23	91	92a-3p; 17-5p; 182-5p; 20a-5p; 155-5p; 96-5p; 18a-5p; 19a-3p; hsa-let-7c-5p; 17-3p; 4521; 183-5p; hsa-let-7e-5p; 873-5p; 93-5p; 200c-3p; 582-5p; 497-5p; 146a-5p; 181c-5p; 106b-5p; 15b-5p; 130b-3p; 25-3p; 9-5p; 16-5p; 192-5p; 30d-5p; 376c-3p; 124-3p; 30a-5p; 410-3p; 186-5p; 9-3p; 31-5p; 451a; 769-5p; 191-5p; 125a-5p; 195-5p; 203a-3p; 379-5p; 128-3p; 485-5p; 143-3p; hsa-let-7a-5p; 218-5p; 135a-5p; 423-5p; 543; 148a-3p; 129-5p; 30c-5p; 30b-5p; 144-3p; 484; 134-5p; 935; 98-5p; 495-3p; 15a-5p; 194-5p; 26a-5p; 296-5p; 106a-5p; 139-5p; 423-3p; 320c; 1-3p; 335-3p; 33b-5p; 181d-5p; 7-5p; 320d; 138-5p; 27b-3p; 204-5p; 494-3p; 32-5p; 21-5p; 20b-5p; 136-5p; 30a-3p; 153-3p; 338-5p; 23b-3p; 211-5p; 125b-5p; 221-5p; 126-3p; 92b-3p
2	epithelial to mesenchymal transition	1.07061E-22	138	92a-3p; 106b-3p; 17-5p; 182-5p; 20a-5p; 155-5p; 96-5p; 18a-5p; 1246; 19a-3p; 19b-1-5p; hsa-let-7c-5p; 17-3p; 142-5p; 183-5p; hsa-let-7e-5p; 142-3p; 1260a; 744-5p; 93-5p; 200c-3p; 19b-3p; 582-5p; 130b-5p; 497-5p; 146a-5p; 181c-5p; 106b-5p; 15b-5p; 130b-3p; 25-3p; 3615; 210-3p; 9-5p; 486-5p; 425-5p; 16-5p; 154-5p; 192-5p; 30d-5p; 376c-3p; 1285-3p; 124-3p; 30a-5p; 410-3p; 186-5p; 9-3p; 299-5p; 31-5p; 625-3p; 769-5p; 505-3p; 136-3p; 154-3p; 491-5p; 195-5p; 203a-3p; 369-5p; 379-5p; 128-3p; 485-5p; hsa-let-7a-5p; 218-5p; 423-5p; 543; 487b-3p; 148a-3p; 129-5p; 29b-1-5p; 129-1-3p; 378c; 30c-5p; 30b-5p; 144-3p; 101-5p; 93-3p; 329-3p; 484; 130a-3p; 3613-5p; 935; 98-5p; 149-5p; 382-3p; 625-5p; 378i; 409-5p; 495-3p; 15a-5p; 376a-5p; 194-5p; 433-3p; 129-2-3p; 26a-5p; 296-5p; 106a-5p; 139-5p; 320c; 1-3p; 335-3p; 33b-5p; 181d-5p; 18b-5p; 378a-3p; 590-5p; 432-5p; 7-5p; 320d; 138-5p; 532-3p; 187-3p; 409-3p; 885-5p; 27b-3p; 204-5p; 27a-3p; 494-3p; 369-3p; 32-5p; hsa-let-7b-5p; 21-5p; 590-3p; 20b-5p; 136-5p; 30a-3p; 340-5p; 378d; 153-3p; 99b-3p; 338-5p; 454-3p; 23b-3p; 211-5p; 145-5p; 370-3p; 504-5p; 125b-5p; 92b-3p
3	pore complex	2.90052E-22	62	92a-3p; 17-5p; 182-5p; 20a-5p; 155-5p; 96-5p; 18a-5p; 142-3p; 92a-1-5p; 744-5p; 93-5p; 200c-3p; 19b-3p; 497-5p; 766-3p; 181c-5p; 106b-5p; 15b-5p; 130b-3p; 25-3p; 9-5p; 16-5p; 192-5p; 376c-3p; 124-3p; 30a-5p; 655-3p; 451a; 125a-5p; 195-5p; 128-3p; 143-3p; hsa-let-7a-5p; 135a-5p; 423-5p; 148a-3p; 30b-5p; 484; 130a-3p; 98-5p; 15a-5p; 361-3p; 106a-5p; 139-5p; 320c; 1-3p; 33b-5p; 181d-5p; 7-5p; 204-5p; 494-3p; 32-5p; 21-5p; 20b-5p; 136-5p; 153-3p; 454-3p; 211-5p; 125b-5p; 221-5p; 126-3p; 92b-3p
4	positive regulation of cytokine secretion	1.5948E-21	56	92a-3p; 106b-3p; 17-5p; 182-5p; 20a-5p; 96-5p; 19a-3p; hsa-let-7c-5p; 17-3p; 142-3p; 92a-1-5p; 744-5p; 93-5p; 146a-5p; 766-3p; 181c-5p; 106b-5p; 15b-5p; 25-3p; 486-5p; 16-5p; 192-5p; 30d-5p; 30a-5p; 451a; 769-5p; 191-5p; 195-5p; 203a-3p; 143-3p; 218-5p; 423-5p; 30c-5p; 30b-5p; 144-3p; 484; 130a-3p; 98-5p; 149-5p; 15a-5p; 361-3p; 194-5p; 106a-5p; 181d-5p; 432-5p; 187-3p; 409-3p; 769-3p; 32-5p; hsa-let-7b-5p; 21-5p; 20b-5p; 23b-3p; 125b-5p; 126-3p; 92b-3p
5	negative regulation of fat cell differentiation	3.84427E-20	114	92a-3p; 106b-3p; 17-5p; 182-5p; 20a-5p; 155-5p; 96-5p; 18a-5p; 1246; 19a-3p; hsa-let-7c-5p; 17-3p; 1290; 142-5p; 183-5p; hsa-let-7e-5p; 142-3p; 744-5p; 873-5p; 93-5p; 200c-3p; 19b-3p; 582-5p; 130b-5p; 497-5p; 146a-5p; 766-3p; 181c-5p; 106b-5p; 15b-5p; 130b-3p; 25-3p; 210-3p; 9-5p; 486-5p; 16-5p; 154-5p; 192-5p; 381-3p; 376a-3p; 376c-3p; 124-3p; 30a-5p; 186-5p; 9-3p; 127-5p; 31-5p; 451a; 769-5p; 154-3p; 491-5p; 195-5p; 203a-3p; 128-3p; 143-3p; hsa-let-7a-5p; 218-5p; 135a-5p; 543; 485-3p; 487b-3p; 148a-3p; 21-3p; 129-5p; 29b-1-5p; 656-3p; 99a-5p; 144-3p; 93-3p; 329-3p; 132-3p; 484; 130a-3p; 3613-5p; 935; 98-5p; 149-5p; 758-3p; 15a-5p; 487a-3p; 26a-5p; 106a-5p; 139-5p; 323a-3p; 423-3p; 1-3p; 132-5p; 33b-5p; 181d-5p; 18b-5p; 590-5p; 7-5p; 138-5p; 187-3p; 885-5p; 27b-3p; 204-5p; 27a-3p; 494-3p; 32-5p; hsa-let-7b-5p; 21-5p; 20b-5p; 136-5p; 153-3p; 23b-3p; 211-5p; 145-5p; 370-3p; 504-5p; 125b-5p; 221-5p; 126-3p; 92b-3p

6	cerebral cortex development	1.13937E-19	88	4488; 92a-3p; 17-5p; 20a-5p; 155-5p; 18a-5p; 19a-3p; 183-5p; hsa-let-7e-5p; 1260b; 744-5p; 873-5p; 93-5p; 200c-3p; 19b-3p; 130b-5p; 181c-5p; 106b-5p; 15b-5p; 130b-3p; 25-3p; 9-5p; 425-5p; 16-5p; 192-5p; 376c-3p; 628-3p; 124-3p; 30a-5p; 410-3p; 186-5p; 127-5p; 769-5p; 491-5p; 125a-5p; 195-5p; 143-3p; 218-5p; 135a-5p; 423-5p; 148a-3p; 129-5p; 29b-1-5p; 30c-5p; 99a-5p; 380-3p; 329-3p; 132-3p; 130a-3p; 98-5p; 149-5p; 758-3p; 495-3p; 15a-5p; 194-5p; 433-3p; 26a-5p; 423-3p; 378a-3p; 432-5p; 7-5p; 532-3p; 339-5p; 382-5p; 885-5p; 27b-3p; 204-5p; 27a-3p; 769-3p; 369-3p; 411-3p; 502-3p; 32-5p; hsa-let-7b-5p; 21-5p; 20b-5p; 136-5p; 454-3p; 331-5p; 211-5p; 145-5p; 370-3p; 125b-5p; 92b-5p; 99b-5p; 126-3p; 92b-3p; 770-5p
7	negative regulation of stress-activated MAPK cascade	1.73582E-19	63	92a-3p; 106b-3p; 17-5p; 182-5p; 20a-5p; 155-5p; 96-5p; 19a-3p; 19b-1-5p; hsa-let-7c-5p; 183-5p; hsa-let-7e-5p; 92a-1-5p; 744-5p; 16-2-3p; 93-5p; 200c-3p; 130b-5p; 106b-5p; 15b-5p; 25-3p; 9-5p; 486-5p; 16-5p; 30d-5p; 376a-3p; 186-5p; 9-3p; 451a; 125a-5p; hsa-let-7a-5p; 135a-5p; 423-5p; 487b-3p; 148a-3p; 30c-5p; 1248; 329-3p; 132-3p; 130a-3p; 134-5p; 935; 98-5p; 149-5p; 15a-5p; 194-5p; 129-2-3p; 323a-3p; 132-5p; 33b-5p; 378a-3p; 7-5p; 27b-3p; 204-5p; 27a-3p; 494-3p; hsa-let-7b-5p; 21-5p; 30a-3p; 153-3p; 145-5p; 370-3p; 92b-3p
8	positive regulation of mesenchymal cell proliferation	5.0906E-19	83	92a-3p; 17-5p; 20a-5p; 155-5p; 18a-5p; 19a-3p; 19b-1-5p; hsa-let-7c-5p; 142-5p; hsa-let-7e-5p; 92a-1-5p; 744-5p; 16-2-3p; 93-5p; 200c-3p; 19b-3p; 582-5p; 130b-5p; 497-5p; 146a-5p; 181c-5p; 106b-5p; 15b-5p; 130b-3p; 25-3p; 9-5p; 16-5p; 30d-5p; 124-3p; 410-3p; 186-5p; 655-3p; 451a; 505-3p; 125a-5p; 195-5p; 203a-3p; hsa-let-7a-5p; 135a-5p; 423-5p; 487b-3p; 148a-3p; 30c-5p; 1248; 101-5p; 329-3p; 484; 130a-3p; 134-5p; 98-5p; 495-3p; 15a-5p; 129-2-3p; 296-5p; 106a-5p; 323a-3p; 423-3p; 1-3p; 3065-5p; 33b-5p; 378a-3p; 590-5p; 7-5p; 138-5p; 409-3p; 204-5p; 383-5p; 494-3p; 369-3p; hsa-let-7b-5p; 21-5p; 20b-5p; 136-5p; 30a-3p; 338-5p; 454-3p; 211-5p; 145-5p; 370-3p; 504-5p; 125b-5p; 126-3p; 92b-3p
9	negative regulation of cell proliferation involved in heart valve morphogenesis	1.44197E-18	41	92a-3p; 17-5p; 20a-5p; 19a-3p; 93-5p; 200c-3p; 19b-3p; 146a-5p; 181c-5p; 106b-5p; 3615; 9-5p; 192-5p; 30d-5p; 30a-5p; 410-3p; 128-3p; 135a-5p; 129-5p; 30c-5p; 30b-5p; 144-3p; 93-3p; 130a-3p; 935; 296-5p; 139-5p; 335-3p; 181d-5p; 7-5p; 27b-3p; 204-5p; 27a-3p; 32-5p; 21-5p; 20b-5p; 30a-3p; 153-3p; 23b-3p; 211-5p; 92b-3p
10	negative regulation of mitochondrial depolarization	1.44197E-18	49	17-5p; 182-5p; 20a-5p; 96-5p; 18a-5p; hsa-let-7c-5p; hsa-let-7e-5p; 93-5p; 200c-3p; 497-5p; 181c-5p; 106b-5p; 15b-5p; 9-5p; 16-5p; 154-5p; 192-5p; 376c-3p; 31-5p; 451a; 125a-5p; 195-5p; 203a-3p; 128-3p; 143-3p; hsa-let-7a-5p; 135a-5p; 148a-3p; 30b-5p; 98-5p; 15a-5p; 106a-5p; 139-5p; 33b-5p; 181d-5p; 7-5p; 27b-3p; 204-5p; 27a-3p; 494-3p; hsa-let-7b-5p; 21-5p; 20b-5p; 136-5p; 153-3p; 23b-3p; 211-5p; 125b-5p; 126-3p
11	channel activity	1.62726E-18	43	17-5p; 182-5p; 20a-5p; 96-5p; 18a-5p; 17-3p; 200c-3p; 497-5p; 766-3p; 181c-5p; 15b-5p; 9-5p; 16-5p; 192-5p; 4524a-3p; 376c-3p; 30a-5p; 451a; 125a-5p; 195-5p; 128-3p; 143-3p; hsa-let-7a-5p; 135a-5p; 148a-3p; 30b-5p; 98-5p; 15a-5p; 139-5p; 1-3p; 33b-5p; 181d-5p; 7-5p; 204-5p; 494-3p; 21-5p; 590-3p; 136-5p; 153-3p; 23b-3p; 211-5p; 125b-5p; 126-3p
12	channel inhibitor activity	1.64933E-18	35	17-5p; 182-5p; 20a-5p; 96-5p; 18a-5p; 200c-3p; 497-5p; 181c-5p; 15b-5p; 9-5p; 16-5p; 192-5p; 376c-3p; 451a; 125a-5p; 195-5p; 143-3p; hsa-let-7a-5p; 135a-5p; 148a-3p; 30b-5p; 98-5p; 15a-5p; 139-5p; 33b-5p; 181d-5p; 7-5p; 204-5p; 494-3p; 21-5p; 136-5p; 153-3p; 211-5p; 125b-5p; 126-3p
13	negative regulation of cellular pH reduction	1.64933E-18	35	17-5p; 182-5p; 20a-5p; 96-5p; 18a-5p; 200c-3p; 497-5p; 181c-5p; 15b-5p; 9-5p; 16-5p; 192-5p; 376c-3p; 451a; 125a-5p; 195-5p; 143-3p; hsa-let-7a-5p; 135a-5p; 148a-3p; 30b-5p; 98-5p; 15a-5p; 139-5p; 33b-5p; 181d-5p; 7-5p; 204-5p; 494-3p; 21-5p; 136-5p; 153-3p; 211-5p; 125b-5p; 126-3p
14	neuron apoptotic process	1.64933E-18	81	92a-3p; 106b-3p; 17-5p; 182-5p; 20a-5p; 155-5p; 96-5p; 18a-5p; 19a-3p; hsa-let-7c-5p; 17-3p; hsa-let-7e-5p; 1260b; 16-2-3p; 93-5p; 200c-3p; 19b-3p; 497-5p; 766-3p; 181c-5p; 106b-5p; 15b-5p; 130b-3p; 25-3p; 210-3p; 9-5p; 486-5p; 16-5p; 154-5p; 192-5p; 376c-3p; 124-3p; 451a; 125a-5p; 195-5p; 203a-3p; 143-3p; hsa-let-7a-5p; 218-5p; 135a-5p; 423-5p; 148a-3p; 129-5p; 30b-5p; 144-3p; 93-3p; 329-3p; 132-3p; 484; 98-5p; 149-5p; 625-5p; 15a-5p; 361-3p; 26a-5p; 296-5p; 106a-5p; 139-5p; 423-3p; 3065-5p; 33b-5p; 181d-5p; 7-5p; 532-3p; 27b-3p; 204-5p; 27a-3p; 769-3p; 494-3p; hsa-let-7b-5p; 21-5p; 20b-5p; 136-5p; 153-3p; 23b-3p; 211-5p; 145-5p; 125b-5p; 126-3p; 323b-3p; 92b-3p

15	R-SMAD binding	1.93271E-18	99	4488; 92a-3p; 17-5p; 182-5p; 20a-5p; 155-5p; 18a-5p; 19a-3p; 19b-1-5p; hsa-let-7c-5p; 142-5p; 183-5p; 1260b; 744-5p; 16-2-3p; 93-5p; 200c-3p; 19b-3p; 497-5p; 146a-5p; 106b-5p; 15b-5p; 130b-3p; 25-3p; 486-5p; 425-5p; 16-5p; 154-5p; 192-5p; 30d-5p; 381-3p; 376a-3p; 124-3p; 30a-5p; 410-3p; 486-3p; 186-5p; 31-5p; 655-3p; 505-3p; 154-3p; 491-5p; 125a-5p; 195-5p; 203a-3p; 128-3p; 143-3p; 218-5p; 543; 148a-3p; 29b-1-5p; 99a-5p; 144-3p; 654-3p; 101-5p; 31-3p; 93-3p; 329-3p; 132-3p; 484; 130a-3p; 134-5p; 149-5p; 625-5p; 15a-5p; 487a-3p; 361-3p; 26a-5p; 106a-5p; 139-5p; 323a-3p; 1-3p; 335-3p; 144-5p; 18b-5p; 629-5p; 590-5p; 7-5p; 532-3p; 382-5p; 27b-3p; 204-5p; 27a-3p; 369-3p; 376b-3p; 32-5p; 21-5p; 20b-5p; 340-5p; 338-5p; 454-3p; 23b-3p; 211-5p; 145-5p; 370-3p; 125b-5p; 323b-3p; 92b-3p; 770-5p
16	I-SMAD binding	2.31579E-18	75	92a-3p; 17-5p; 182-5p; 20a-5p; 155-5p; 18a-5p; 19a-3p; hsa-let-7c-5p; 183-5p; 142-3p; 1260b; 744-5p; 93-5p; 19b-3p; 497-5p; 146a-5p; 181c-5p; 106b-5p; 15b-5p; 130b-3p; 25-3p; 486-5p; 425-5p; 16-5p; 154-5p; 30d-5p; 381-3p; 376a-3p; 376c-3p; 124-3p; 30a-5p; 186-5p; 31-5p; 505-3p; 125a-5p; 195-5p; 203a-3p; 379-5p; 128-3p; 218-5p; 148a-3p; 21-3p; 30c-5p; 30b-5p; 144-3p; 329-3p; 132-3p; 484; 130a-3p; 134-5p; 98-5p; 15a-5p; 26a-5p; 106a-5p; 323a-3p; 320c; 144-5p; 18b-5p; 590-5p; 532-3p; 885-5p; 27b-3p; 204-5p; 27a-3p; 769-3p; 32-5p; hsa-let-7b-5p; 21-5p; 20b-5p; 454-3p; 211-5p; 145-5p; 370-3p; 125b-5p; 92b-3p
17	aortic valve morphogenesis	2.31579E-18	99	92a-3p; 17-5p; 182-5p; 20a-5p; 155-5p; 96-5p; hsa-let-7c-5p; 142-5p; 183-5p; 142-3p; 744-5p; 873-5p; 93-5p; 200c-3p; 582-5p; 497-5p; 146a-5p; 106b-5p; 15b-5p; 130b-3p; 25-3p; 210-3p; 9-5p; 486-5p; 16-5p; 192-5p; 30d-5p; 381-3p; 1185-5p; 124-3p; 30a-5p; 410-3p; 186-5p; 299-5p; 625-3p; 539-5p; 491-5p; 125a-5p; 195-5p; 203a-3p; 128-3p; 485-5p; 143-3p; 218-5p; 135a-5p; 543; 148a-3p; 129-5p; 29b-1-5p; 30c-5p; 30b-5p; 99a-5p; 380-3p; 144-3p; 101-5p; 93-3p; 329-3p; 132-3p; 130a-3p; 134-5p; 935; 98-5p; 149-5p; 495-3p; 15a-5p; 26a-5p; 296-5p; 106a-5p; 139-5p; 423-3p; 1-3p; 3065-5p; 335-3p; 144-5p; 33b-5p; 590-5p; 7-5p; 138-5p; 27b-3p; 204-5p; 27a-3p; 769-3p; 494-3p; 411-3p; 139-3p; 32-5p; hsa-let-7b-5p; 21-5p; 20b-5p; 136-5p; 30a-3p; 340-5p; 153-3p; 338-5p; 23b-3p; 211-5p; 145-5p; 126-3p; 92b-3p
18	interleukin-7-mediated signaling pathway	2.31579E-18	93	92a-3p; 106b-3p; 17-5p; 182-5p; 20a-5p; 155-5p; 96-5p; 19a-3p; hsa-let-7c-5p; 1290; 142-5p; 183-5p; hsa-let-7e-5p; 92a-1-5p; 93-5p; 200c-3p; 19b-3p; 130b-5p; 497-5p; 146a-5p; 181c-5p; 106b-5p; 15b-5p; 130b-3p; 9-5p; 16-5p; 192-5p; 30d-5p; 376a-3p; 124-3p; 30a-5p; 410-3p; 186-5p; 299-5p; 539-5p; 125a-5p; 203a-3p; 369-5p; 128-3p; hsa-let-7a-5p; 135a-5p; 423-5p; 148a-3p; 21-3p; 29b-1-5p; 30c-5p; 30b-5p; 380-3p; 628-5p; 144-3p; 31-3p; 93-3p; 329-3p; 132-3p; 484; 130a-3p; 134-5p; 98-5p; 15a-5p; 361-3p; 194-5p; 433-3p; 106a-5p; 139-5p; 323a-3p; 423-3p; 1-3p; 335-3p; 181d-5p; 378a-3p; 629-5p; 487a-5p; 7-5p; 124-5p; 382-5p; 27b-3p; 340-3p; 769-3p; 369-3p; 411-3p; 376b-3p; hsa-let-7b-5p; 21-5p; 20b-5p; 340-5p; 454-3p; 23b-3p; 331-5p; 28-3p; 145-5p; 504-5p; 125b-5p; 126-3p
19	SMAD protein complex assembly	2.50025E-18	70	92a-3p; 17-5p; 182-5p; 20a-5p; 155-5p; 18a-5p; 19a-3p; 142-5p; 183-5p; 1260b; 744-5p; 93-5p; 19b-3p; 497-5p; 146a-5p; 181c-5p; 106b-5p; 15b-5p; 130b-3p; 25-3p; 486-5p; 425-5p; 16-5p; 154-5p; 30d-5p; 381-3p; 30a-5p; 410-3p; 186-5p; 31-5p; 505-3p; 491-5p; 125a-5p; 195-5p; 203a-3p; 128-3p; 143-3p; 218-5p; 543; 148a-3p; 21-3p; 29b-1-5p; 30c-5p; 30b-5p; 144-3p; 329-3p; 132-3p; 484; 130a-3p; 15a-5p; 433-3p; 26a-5p; 106a-5p; 323a-3p; 144-5p; 181d-5p; 18b-5p; 590-5p; 27b-3p; 204-5p; 27a-3p; 369-3p; 32-5p; 21-5p; 20b-5p; 454-3p; 211-5p; 145-5p; 125b-5p; 92b-3p
20	positive regulation of protein import into nucleus	3.48691E-18	90	92a-3p; 17-5p; 20a-5p; 155-5p; 96-5p; 18a-5p; 19a-3p; 142-5p; 183-5p; 142-3p; 1260b; 744-5p; 93-5p; 200c-3p; 19b-3p; 497-5p; 146a-5p; 766-3p; 181c-5p; 106b-5p; 15b-5p; 130b-3p; 25-3p; 9-5p; 486-5p; 16-5p; 192-5p; 30d-5p; 376a-3p; 1285-3p; 124-3p; 30a-5p; 410-3p; 486-3p; 186-5p; 9-3p; 31-5p; 655-3p; 505-3p; 491-5p; 125a-5p; 195-5p; 203a-3p; 128-3p; 143-3p; hsa-let-7a-5p; 218-5p; 423-5p; 129-5p; 29b-1-5p; 30c-5p; 144-3p; 654-3p; 101-5p; 31-3p; 93-3p; 329-3p; 132-3p; 484; 130a-3p; 935; 149-5p; 15a-5p; 487a-3p; 26a-5p; 106a-5p; 323a-3p; 1-3p; 181d-5p; 378a-3p; 590-5p; 7-5p; 138-5p; 409-3p; 339-5p; 27b-3p; 204-5p; 27a-3p; 369-3p; hsa-let-7b-5p; 21-5p; 20b-5p; 30a-3p; 454-3p; 23b-3p; 211-5p; 145-5p; 126-3p; 92b-3p; 1296-5p
21	endocardial cell differentiation	4.93865E-18	52	17-5p; 182-5p; 20a-5p; 155-5p; 18a-5p; 19a-3p; 183-5p; 1260b; 93-5p; 200c-3p; 19b-3p; 146a-5p; 106b-5p; 130b-3p; 9-5p; 154-5p; 30d-5p; 124-3p; 30a-5p; 410-3p; 186-5p; 31-5p; 125a-5p; 203a-3p; 218-5p; 129-5p; 30c-5p; 30b-5p; 144-3p; 132-3p; 130a-3p; 134-5p; 935; 758-3p; 26a-5p; 296-5p; 106a-5p; 139-5p; 1-3p; 335-3p; 144-5p; 27b-3p; 204-5p; 27a-3p; 769-3p; 20b-5p; 30a-3p; 454-3p; 23b-3p; 211-5p; 145-5p; 125b-5p

22	necroptotic signaling pathway	7.1073E-18	32	92a-3p; 17-5p; 155-5p; 18a-5p; 19a-3p; hsa-let-7e-5p; 92a-1-5p; 19b-3p; 146a-5p; 106b-5p; 15b-5p; 25-3p; 124-3p; 203a-3p; 128-3p; 143-3p; 485-3p; 21-3p; 329-3p; 130a-3p; 98-5p; 149-5p; 758-3p; 106a-5p; 1-3p; 7-5p; 187-3p; 32-5p; 21-5p; 504-5p; 125b-5p; 92b-3p
23	COPI vesicle coat	2.4429E-17	55	92a-3p; 106b-3p; 17-5p; 20a-5p; 155-5p; 183-5p; hsa-let-7e-5p; 142-3p; 1260b; 1260a; 744-5p; 93-5p; 582-5p; 130b-5p; 146a-5p; 106b-5p; 15b-5p; 130b-3p; 16-5p; 381-3p; 1185-5p; 376a-3p; 124-3p; 30a-5p; 486-3p; 127-5p; 451a; 539-5p; 125a-5p; 195-5p; hsa-let-7a-5p; 423-5p; 148a-3p; 30c-5p; 101-5p; 93-3p; 484; 98-5p; 15a-5p; 376a-5p; 26a-5p; 139-5p; 423-3p; 1-3p; 27b-3p; 27a-3p; 376b-3p; hsa-let-7b-5p; 20b-5p; 23b-3p; 28-3p; 125b-5p; 92b-3p; 770-5p; 1296-5p

MiRPathDB GO Molecular function

No	subcategory	q-value	number of miRs observed	hsa-miRs
1	enzyme binding	6.946E-23	69	92a-3p; 106b-3p; 17-5p; 182-5p; 20a-5p; 155-5p; 96-5p; 18a-5p; 19a-3p; hsa-let-7c-5p; 183-5p; 142-3p; 92a-1-5p; 744-5p; 93-5p; 200c-3p; 19b-3p; 497-5p; 146a-5p; 106b-5p; 15b-5p; 130b-3p; 9-5p; 16-5p; 30d-5p; 30a-5p; 486-3p; 31-5p; 625-3p; 451a; 505-3p; 191-5p; 491-5p; 125a-5p; 195-5p; 203a-3p; 128-3p; hsa-let-7a-5p; 218-5p; 135a-5p; 423-5p; 148a-3p; 30c-5p; 30b-5p; 144-3p; 93-3p; 484; 149-5p; 625-5p; 15a-5p; 106a-5p; 423-3p; 1-3p; 378a-3p; 138-5p; 27b-3p; 204-5p; 27a-3p; 494-3p; hsa-let-7b-5p; 21-5p; 20b-5p; 23b-3p; 211-5p; 145-5p; 125b-5p; 126-3p; 92b-3p; 1296-5p
2	protein binding	6.56347E-18	71	17-5p; 182-5p; 20a-5p; 155-5p; 96-5p; 18a-5p; 19a-3p; hsa-let-7c-5p; 17-3p; 92a-1-5p; 744-5p; 93-5p; 200c-3p; 19b-3p; 497-5p; 106b-5p; 15b-5p; 130b-3p; 25-3p; 9-5p; 192-5p; 30d-5p; 124-3p; 30a-5p; 186-5p; 31-5p; 625-3p; 451a; 505-3p; 191-5p; 125a-5p; 195-5p; 203a-3p; 128-3p; hsa-let-7a-5p; 218-5p; 423-5p; 148a-3p; 656-3p; 30c-5p; 30b-5p; 99a-5p; 93-3p; 484; 130a-3p; 98-5p; 149-5p; 625-5p; 15a-5p; 26a-5p; 106a-5p; 139-5p; 423-3p; 320c; 1-3p; 378a-3p; 7-5p; 320d; 138-5p; 27b-3p; 27a-3p; 494-3p; hsa-let-7b-5p; 21-5p; 20b-5p; 23b-3p; 145-5p; 125b-5p; 126-3p; 92b-3p; 1296-5p
3	protein serinethreonine kinase activity	1.01174E-16	51	17-5p; 182-5p; 20a-5p; 155-5p; 96-5p; 1246; 19a-3p; hsa-let-7c-5p; 744-5p; 93-5p; 200c-3p; 19b-3p; 582-5p; 497-5p; 146a-5p; 181c-5p; 15b-5p; 9-5p; 16-5p; 192-5p; 376c-3p; 410-3p; 451a; 125a-5p; 195-5p; 203a-3p; 128-3p; hsa-let-7a-5p; 218-5p; 135a-5p; 148a-3p; 99a-5p; 93-3p; 130a-3p; 625-5p; 15a-5p; 26a-5p; 106a-5p; 1-3p; 33b-5p; 378a-3p; 138-5p; 27b-3p; 27a-3p; 494-3p; hsa-let-7b-5p; 21-5p; 153-3p; 145-5p; 125b-5p; 126-3p
4	transferase activity	1.11151E-15	46	92a-3p; 17-5p; 20a-5p; 155-5p; 96-5p; 19a-3p; 17-3p; 183-5p; 744-5p; 93-5p; 200c-3p; 19b-3p; 497-5p; 106b-5p; 15b-5p; 130b-3p; 16-5p; 192-5p; 376c-3p; 186-5p; 451a; 125a-5p; 195-5p; 203a-3p; 128-3p; hsa-let-7a-5p; 218-5p; 135a-5p; 148a-3p; 30c-5p; 101-5p; 93-3p; 484; 130a-3p; 98-5p; 15a-5p; 26a-5p; 106a-5p; 27b-3p; 27a-3p; hsa-let-7b-5p; 21-5p; 20b-5p; 145-5p; 125b-5p; 126-3p
5	organic cyclic compound binding	1.87526E-14	85	92a-3p; 106b-3p; 17-5p; 182-5p; 20a-5p; 155-5p; 96-5p; 18a-5p; 1246; 19a-3p; hsa-let-7c-5p; 17-3p; 142-5p; 183-5p; hsa-let-7e-5p; 1260b; 92a-1-5p; 744-5p; 93-5p; 200c-3p; 19b-3p; 582-5p; 497-5p; 181c-5p; 106b-5p; 15b-5p; 25-3p; 210-3p; 9-5p; 425-5p; 16-5p; 381-3p; 376c-3p; 30a-5p; 410-3p; 186-5p; 31-5p; 539-5p; 505-3p; 491-5p; 125a-5p; 195-5p; 203a-3p; hsa-let-7a-5p; 218-5p; 135a-5p; 423-5p; 148a-3p; 30c-5p; 30b-5p; 99a-5p; 628-5p; 144-3p; 654-3p; 93-3p; 484; 130a-3p; 935; 149-5p; 15a-5p; 26a-5p; 106a-5p; 139-5p; 323a-3p; 423-3p; 320c; 1-3p; 33b-5p; 181d-5p; 378a-3p; 320d; 138-5p; 27b-3p; 27a-3p; 494-3p; hsa-let-7b-5p; 21-5p; 340-5p; 23b-3p; 145-5p; 125b-5p; 99b-5p; 92b-3p; 770-5p; 1296-5p
6	protein kinase binding	7.40314E-14	44	92a-3p; 17-5p; 182-5p; 20a-5p; 155-5p; 96-5p; 18a-5p; 19a-3p; 142-5p; 744-5p; 200c-3p; 497-5p; 15b-5p; 16-5p; 124-3p; 30a-5p; 486-3p; 186-5p; 31-5p; 625-3p; 451a; 505-3p; 491-5p; 125a-5p; 195-5p; 218-5p; 135a-5p; 148a-3p; 30c-5p; 30b-5p; 484; 149-5p; 15a-5p; 106a-5p; 323a-3p; 378a-3p; 138-5p; 27a-3p; 369-3p; hsa-let-7b-5p; 21-5p; 145-5p; 126-3p; 92b-3p

7	double-stranded DNA binding	2.5096E-13	61	106b-3p; 17-5p; 182-5p; 20a-5p; 155-5p; 96-5p; 18a-5p; 19a-3p; hsa-let-7c-5p; 142-5p; 92a-1-5p; 93-5p; 200c-3p; 19b-3p; 582-5p; 497-5p; 146a-5p; 106b-5p; 15b-5p; 130b-3p; 210-3p; 9-5p; 30d-5p; 381-3p; 410-3p; 9-3p; 31-5p; 539-5p; 191-5p; 491-5p; 125a-5p; 195-5p; 203a-3p; 128-3p; 218-5p; 135a-5p; 423-5p; 543; 485-3p; 30c-5p; 144-3p; 130a-3p; 149-5p; 625-5p; 15a-5p; 26a-5p; 106a-5p; 139-5p; 320c; 33b-5p; 378a-3p; 138-5p; 885-5p; 27b-3p; 204-5p; 27a-3p; 21-5p; 211-5p; 145-5p; 125b-5p; 126-3p
8	nucleic acid binding	2.5096E-13	86	92a-3p; 106b-3p; 17-5p; 182-5p; 20a-5p; 155-5p; 96-5p; 18a-5p; 19a-3p; hsa-let-7c-5p; 17-3p; 142-5p; 183-5p; hsa-let-7e-5p; 1260b; 92a-1-5p; 744-5p; 93-5p; 200c-3p; 19b-3p; 582-5p; 497-5p; 766-3p; 181c-5p; 106b-5p; 15b-5p; 25-3p; 210-3p; 425-5p; 16-5p; 30d-5p; 381-3p; 30a-5p; 410-3p; 186-5p; 31-5p; 655-3p; 539-5p; 505-3p; 491-5p; 125a-5p; 195-5p; 203a-3p; hsa-let-7a-5p; 218-5p; 135a-5p; 423-5p; 543; 485-3p; 148a-3p; 30c-5p; 30b-5p; 99a-5p; 144-3p; 93-3p; 484; 130a-3p; 935; 149-5p; 15a-5p; 26a-5p; 106a-5p; 139-5p; 323a-3p; 423-3p; 425-3p; 320c; 1-3p; 335-3p; 33b-5p; 181d-5p; 378a-3p; 590-5p; 320d; 138-5p; 27b-3p; 27a-3p; hsa-let-7b-5p; 21-5p; 340-5p; 23b-3p; 145-5p; 125b-5p; 92b-3p; 770-5p; 1296-5p
9	molecular function	5.31932E-13	47	106b-3p; 182-5p; 155-5p; 96-5p; 18a-5p; 19a-3p; hsa-let-7c-5p; 17-3p; 744-5p; 200c-3p; 19b-3p; 497-5p; 15b-5p; 130b-3p; 25-3p; 9-5p; 30d-5p; 30a-5p; 186-5p; 31-5p; 125a-5p; 195-5p; 203a-3p; 128-3p; hsa-let-7a-5p; 218-5p; 423-5p; 148a-3p; 30c-5p; 30b-5p; 99a-5p; 484; 130a-3p; 98-5p; 149-5p; 15a-5p; 26a-5p; 139-5p; 1-3p; 27b-3p; 27a-3p; 21-5p; 23b-3p; 145-5p; 125b-5p; 126-3p; 92b-3p
10	catalytic activity	9.55184E-13	33	92a-3p; 106b-3p; 17-5p; 155-5p; 96-5p; 19a-3p; 183-5p; 744-5p; 200c-3p; 146a-5p; 210-3p; 16-5p; 192-5p; 30a-5p; 186-5p; 451a; 125a-5p; 203a-3p; 218-5p; 487b-3p; 148a-3p; 30c-5p; 99a-5p; 484; 15a-5p; 26a-5p; 1-3p; 494-3p; hsa-let-7b-5p; 21-5p; 145-5p; 125b-5p; 126-3p
11	transcription regulatory region sequence-specific DNA binding	9.55184E-13	57	17-5p; 182-5p; 20a-5p; 155-5p; 18a-5p; 19a-3p; hsa-let-7c-5p; 142-5p; 92a-1-5p; 93-5p; 200c-3p; 19b-3p; 582-5p; 497-5p; 106b-5p; 130b-3p; 210-3p; 9-5p; 30d-5p; 381-3p; 410-3p; 9-3p; 31-5p; 655-3p; 539-5p; 191-5p; 491-5p; 125a-5p; 195-5p; 203a-3p; 128-3p; 218-5p; 135a-5p; 423-5p; 543; 485-3p; 30c-5p; 30b-5p; 144-3p; 130a-3p; 149-5p; 625-5p; 15a-5p; 106a-5p; 139-5p; 323a-3p; 320c; 33b-5p; 378a-3p; 138-5p; 204-5p; 27a-3p; 21-5p; 211-5p; 145-5p; 125b-5p; 126-3p
12	protein domain specific binding	1.08813E-12	44	92a-3p; 106b-3p; 17-5p; 20a-5p; 155-5p; 18a-5p; 744-5p; 200c-3p; 582-5p; 497-5p; 146a-5p; 15b-5p; 130b-3p; 486-5p; 16-5p; 124-3p; 30a-5p; 31-5p; 655-3p; 491-5p; 125a-5p; 195-5p; 203a-3p; 218-5p; 135a-5p; 423-5p; 543; 148a-3p; 656-3p; 30c-5p; 329-3p; 484; 149-5p; 15a-5p; 323a-3p; 1-3p; 378a-3p; 27a-3p; hsa-let-7b-5p; 21-5p; 23b-3p; 145-5p; 126-3p; 1296-5p
13	transcription regulatory region DNA binding	1.81144E-12	61	17-5p; 182-5p; 20a-5p; 155-5p; 18a-5p; 19a-3p; hsa-let-7c-5p; 142-5p; 92a-1-5p; 93-5p; 200c-3p; 19b-3p; 497-5p; 146a-5p; 106b-5p; 15b-5p; 130b-3p; 210-3p; 9-5p; 30d-5p; 381-3p; 410-3p; 9-3p; 31-5p; 655-3p; 539-5p; 191-5p; 491-5p; 125a-5p; 195-5p; 203a-3p; 128-3p; 218-5p; 135a-5p; 543; 485-3p; 30c-5p; 99a-5p; 144-3p; 130a-3p; 149-5p; 625-5p; 15a-5p; 26a-5p; 106a-5p; 139-5p; 320c; 33b-5p; 378a-3p; 590-5p; 138-5p; 885-5p; 27b-3p; 204-5p; 27a-3p; 494-3p; 21-5p; 211-5p; 145-5p; 125b-5p; 126-3p
14	RNA binding	3.38632E-12	51	92a-3p; 106b-3p; 182-5p; 155-5p; 18a-5p; 17-3p; 183-5p; hsa-let-7e-5p; 1260b; 92a-1-5p; 744-5p; 582-5p; 497-5p; 15b-5p; 25-3p; 425-5p; 16-5p; 628-3p; 30a-5p; 186-5p; 31-5p; 539-5p; 505-3p; 491-5p; 195-5p; hsa-let-7a-5p; 218-5p; 423-5p; 148a-3p; 30c-5p; 99a-5p; 93-3p; 484; 935; 149-5p; 15a-5p; 26a-5p; 323a-3p; 423-3p; 1-3p; 378a-3p; 27b-3p; 27a-3p; hsa-let-7b-5p; 30a-3p; 99b-3p; 338-5p; 23b-3p; 125b-5p; 92b-3p; 1296-5p

MiRPathDB KEGG

No	subcategory	q-value	number of miRs observed	hsa-miRs

1	miRNAs in cancer	1.31513E-18	90	92a-3p; 17-5p; 182-5p; 20a-5p; 155-5p; 96-5p; 18a-5p; 19a-3p; 19b-1-5p; hsa-let-7c-5p; 17-3p; 142-5p; 183-5p; hsa-let-7e-5p; 744-5p; 16-2-3p; 93-5p; 200c-3p; 497-5p; 146a-5p; 181c-5p; 106b-5p; 15b-5p; 25-3p; 9-5p; 425-5p; 16-5p; 376c-3p; 628-3p; 124-3p; 30a-5p; 9-3p; 31-5p; 451a; 191-5p; 491-5p; 125a-5p; 195-5p; 203a-3p; 369-5p; 128-3p; 143-3p; hsa-let-7a-5p; 218-5p; 135a-5p; 543; 487b-3p; 148a-3p; 21-3p; 29b-1-5p; 30c-5p; 144-3p; 31-3p; 132-3p; 130a-3p; 134-5p; 98-5p; 15a-5p; 95-3p; 194-5p; 433-3p; 129-2-3p; 26a-5p; 106a-5p; 139-5p; 323a-3p; 1-3p; 144-5p; 33b-5p; 18b-5p; 378a-3p; 7-5p; 138-5p; 27b-3p; 204-5p; 27a-3p; 494-3p; 32-5p; hsa-let-7b-5p; 21-5p; 20b-5p; 30a-3p; 340-5p; 153-3p; 23b-3p; 211-5p; 145-5p; 504-5p; 125b-5p; 126-3p
2	proteoglycans in cancer	9.5499E-15	63	17-5p; 155-5p; 18a-5p; 19a-3p; hsa-let-7c-5p; 142-5p; 744-5p; 200c-3p; 582-5p; 497-5p; 146a-5p; 106b-5p; 15b-5p; 16-5p; 376c-3p; 124-3p; 30a-5p; 9-3p; 31-5p; 625-3p; 451a; 125a-5p; 195-5p; 203a-3p; 143-3p; hsa-let-7a-5p; 218-5p; 135a-5p; 487b-3p; 148a-3p; 21-3p; 30c-5p; 329-3p; 132-3p; 935; 98-5p; 95-3p; 194-5p; 433-3p; 26a-5p; 106a-5p; 139-5p; 323a-3p; 1-3p; 144-5p; 18b-5p; 378a-3p; 590-5p; 7-5p; 138-5p; 187-3p; 885-5p; 204-5p; 27a-3p; 494-3p; 21-5p; 30a-3p; 340-5p; 23b-3p; 145-5p; 125b-5p; 126-3p; 1249-3p
3	hepatitis B	3.62524E-14	64	17-5p; 182-5p; 20a-5p; 155-5p; 18a-5p; 19a-3p; hsa-let-7c-5p; 142-5p; 92a-1-5p; 93-5p; 200c-3p; 497-5p; 146a-5p; 106b-5p; 15b-5p; 25-3p; 425-5p; 16-5p; 124-3p; 9-3p; 451a; 125a-5p; 195-5p; 203a-3p; 143-3p; hsa-let-7a-5p; 135a-5p; 543; 487b-3p; 148a-3p; 21-3p; 29b-1-5p; 30c-5p; 132-3p; 130a-3p; 98-5p; 15a-5p; 95-3p; 433-3p; 26a-5p; 106a-5p; 139-5p; 323a-3p; 144-5p; 33b-5p; 378a-3p; 590-5p; 7-5p; 138-5p; 204-5p; 27a-3p; 494-3p; 32-5p; hsa-let-7b-5p; 21-5p; 20b-5p; 30a-3p; 23b-3p; 28-3p; 211-5p; 145-5p; 125b-5p; 126-3p; 323b-3p
4	FoxO signaling pathway	6.75084E-13	62	17-5p; 182-5p; 155-5p; 96-5p; 18a-5p; 19a-3p; hsa-let-7c-5p; 142-5p; hsa-let-7e-5p; 92a-1-5p; 200c-3p; 497-5p; 146a-5p; 15b-5p; 9-5p; 486-5p; 425-5p; 16-5p; 376a-3p; 376c-3p; 124-3p; 30a-5p; 451a; 125a-5p; 195-5p; 203a-3p; 128-3p; 143-3p; hsa-let-7a-5p; 543; 148a-3p; 21-3p; 29b-1-5p; 132-3p; 130a-3p; 98-5p; 15a-5p; 95-3p; 194-5p; 26a-5p; 106a-5p; 139-5p; 323a-3p; 144-5p; 18b-5p; 378a-3p; 590-5p; 432-5p; 7-5p; 127-3p; 885-5p; 27b-3p; 27a-3p; 494-3p; hsa-let-7b-5p; 21-5p; 340-5p; 23b-3p; 145-5p; 370-3p; 125b-5p; 126-3p
5	pathways in cancer	6.45929E-12	70	92a-3p; 17-5p; 182-5p; 20a-5p; 155-5p; 96-5p; 18a-5p; 1246; 19a-3p; hsa-let-7c-5p; 142-5p; 93-5p; 200c-3p; 582-5p; 497-5p; 146a-5p; 106b-5p; 15b-5p; 9-5p; 486-5p; 425-5p; 16-5p; 376c-3p; 124-3p; 9-3p; 451a; 491-5p; 125a-5p; 195-5p; 203a-3p; 128-3p; 143-3p; hsa-let-7a-5p; 218-5p; 135a-5p; 543; 487b-3p; 148a-3p; 29b-1-5p; 329-3p; 132-3p; 130a-3p; 98-5p; 15a-5p; 95-3p; 194-5p; 433-3p; 26a-5p; 106a-5p; 139-5p; 1-3p; 144-5p; 378a-3p; 590-5p; 7-5p; 138-5p; 885-5p; 204-5p; 27a-3p; 494-3p; 21-5p; 20b-5p; 30a-3p; 23b-3p; 28-3p; 145-5p; 370-3p; 125b-5p; 126-3p; 1249-3p
6	HTLV-I infection	1.49081E-11	48	17-5p; 182-5p; 155-5p; 96-5p; 18a-5p; 19a-3p; 93-5p; 200c-3p; 497-5p; 146a-5p; 106b-5p; 15b-5p; 9-5p; 16-5p; 376c-3p; 124-3p; 30a-5p; 451a; 125a-5p; 195-5p; 203a-3p; 143-3p; 543; 487b-3p; 148a-3p; 29b-1-5p; 132-3p; 130a-3p; 935; 15a-5p; 95-3p; 26a-5p; 106a-5p; 139-5p; 323a-3p; 590-5p; 7-5p; 138-5p; 27b-3p; 27a-3p; 494-3p; 21-5p; 30a-3p; 23b-3p; 28-3p; 145-5p; 125b-5p; 126-3p
7	bladder cancer	8.14716E-11	49	17-5p; 20a-5p; 155-5p; 19a-3p; hsa-let-7c-5p; 142-5p; 93-5p; 200c-3p; 497-5p; 146a-5p; 106b-5p; 15b-5p; 130b-3p; 25-3p; 425-5p; 16-5p; 376c-3p; 9-3p; 451a; 491-5p; 125a-5p; 195-5p; 203a-3p; 143-3p; hsa-let-7a-5p; 135a-5p; 487b-3p; 148a-3p; 132-3p; 130a-3p; 134-5p; 98-5p; 95-3p; 106a-5p; 1-3p; 18b-5p; 378a-3p; 590-5p; 7-5p; 27a-3p; 494-3p; hsa-let-7b-5p; 21-5p; 20b-5p; 30a-3p; 28-3p; 145-5p; 125b-5p; 126-3p
8	colorectal cancer	5.88032E-10	49	17-5p; 20a-5p; 155-5p; 96-5p; 18a-5p; 1246; 19a-3p; 142-5p; 93-5p; 200c-3p; 582-5p; 497-5p; 146a-5p; 106b-5p; 15b-5p; 425-5p; 16-5p; 30a-5p; 451a; 125a-5p; 195-5p; 203a-3p; 143-3p; 135a-5p; 487b-3p; 148a-3p; 29b-1-5p; 30c-5p; 130a-3p; 378i; 15a-5p; 106a-5p; 139-5p; 323a-3p; 33b-5p; 378a-3p; 590-5p; 7-5p; 138-5p; 409-3p; 885-5p; 27a-3p; 494-3p; 21-5p; 378d; 211-5p; 145-5p; 125b-5p; 126-3p
9	prostate cancer	1.06413E-09	62	92a-3p; 17-5p; 182-5p; 20a-5p; 155-5p; 96-5p; 19a-3p; 744-5p; 93-5p; 200c-3p; 582-5p; 497-5p; 146a-5p; 106b-5p; 15b-5p; 25-3p; 9-5p; 486-5p; 425-5p; 16-5p; 376a-3p; 376c-3p; 124-3p; 9-3p; 451a; 125a-5p; 195-5p; 203a-3p; 128-3p; 143-3p; hsa-let-7a-5p; 218-5p; 135a-5p; 543; 148a-3p; 132-3p; 98-5p; 15a-5p; 95-3p; 194-5p; 433-3p; 26a-5p; 106a-5p; 139-5p; 144-5p; 378a-3p; 590-5p; 7-5p; 885-5p; 204-5p; 27a-3p; 494-3p; hsa-let-7b-5p; 21-5p; 20b-5p; 153-3p; 23b-3p; 28-3p; 145-5p; 370-3p; 125b-5p; 126-3p

10	chronic myeloid leukemia	2.35454E-09	57	17-5p; 20a-5p; 155-5p; 18a-5p; 19a-3p; hsa-let-7c-5p; 142-5p; 93-5p; 200c-3p; 497-5p; 146a-5p; 106b-5p; 15b-5p; 425-5p; 16-5p; 376c-3p; 124-3p; 9-3p; 451a; 125a-5p; 195-5p; 203a-3p; 143-3p; hsa-let-7a-5p; 543; 487b-3p; 148a-3p; 29b-1-5p; 132-3p; 130a-3p; 98-5p; 378i; 15a-5p; 95-3p; 26a-5p; 106a-5p; 139-5p; 323a-3p; 144-5p; 378a-3p; 590-5p; 7-5p; 138-5p; 27a-3p; 494-3p; hsa-let-7b-5p; 21-5p; 20b-5p; 30a-3p; 340-5p; 378d; 23b-3p; 28-3p; 145-5p; 370-3p; 125b-5p; 126-3p
11	renal cell carcinoma	2.57674E-09	39	155-5p; 19a-3p; 142-5p; 93-5p; 200c-3p; 497-5p; 15b-5p; 16-5p; 9-3p; 625-3p; 451a; 125a-5p; 195-5p; 203a-3p; 143-3p; 543; 378c; 30c-5p; 329-3p; 132-3p; 134-5p; 378i; 15a-5p; 194-5p; 433-3p; 106a-5p; 139-5p; 378a-3p; 7-5p; 409-3p; 27a-3p; 494-3p; 21-5p; 30a-3p; 378d; 23b-3p; 145-5p; 125b-5p; 126-3p
12	toxoplasmosis	2.85519E-09	26	17-5p; 155-5p; 19a-3p; 497-5p; 146a-5p; 15b-5p; 30d-5p; 124-3p; 30a-5p; 9-3p; 625-3p; 451a; 125a-5p; 203a-3p; 143-3p; 148a-3p; 29b-1-5p; 132-3p; 15a-5p; 106a-5p; 7-5p; 138-5p; 494-3p; 21-5p; 125b-5p; 126-3p
13	pancreatic cancer	4.68161E-09	49	17-5p; 20a-5p; 155-5p; 18a-5p; 19a-3p; 142-5p; 93-5p; 200c-3p; 582-5p; 497-5p; 146a-5p; 106b-5p; 15b-5p; 486-5p; 16-5p; 30a-5p; 9-3p; 451a; 491-5p; 125a-5p; 195-5p; 203a-3p; 128-3p; 143-3p; hsa-let-7a-5p; 148a-3p; 21-3p; 29b-1-5p; 30c-5p; 329-3p; 132-3p; 378i; 15a-5p; 106a-5p; 323a-3p; 378a-3p; 590-5p; 7-5p; 138-5p; 27a-3p; 494-3p; 21-5p; 20b-5p; 378d; 23b-3p; 28-3p; 145-5p; 125b-5p; 126-3p
14	HIF-1 signaling pathway	2.07991E-08	37	182-5p; 155-5p; 96-5p; hsa-let-7c-5p; 1260b; 200c-3p; 497-5p; 15b-5p; 486-5p; 9-3p; 625-3p; 451a; 125a-5p; 203a-3p; 143-3p; hsa-let-7a-5p; 148a-3p; 132-3p; 134-5p; 935; 98-5p; 194-5p; 433-3p; 106a-5p; 139-5p; 378a-3p; 7-5p; 138-5p; 27a-3p; 494-3p; hsa-let-7b-5p; 21-5p; 145-5p; 125b-5p; 99b-5p; 126-3p; 1296-5p
15	PI3K-Akt signaling pathway	2.09564E-08	48	17-5p; 182-5p; 155-5p; 96-5p; 19a-3p; hsa-let-7c-5p; 200c-3p; 582-5p; 497-5p; 146a-5p; 15b-5p; 425-5p; 16-5p; 124-3p; 30a-5p; 451a; 125a-5p; 195-5p; 203a-3p; 143-3p; hsa-let-7a-5p; 218-5p; 135a-5p; 487b-3p; 148a-3p; 29b-1-5p; 98-5p; 15a-5p; 95-3p; 433-3p; 26a-5p; 106a-5p; 139-5p; 1-3p; 144-5p; 33b-5p; 378a-3p; 7-5p; 138-5p; 27a-3p; 494-3p; 32-5p; 21-5p; 30a-3p; 153-3p; 145-5p; 125b-5p; 126-3p
16	Epstein-Barr virus infection	3.35926E-08	38	92a-3p; 155-5p; 19a-3p; hsa-let-7e-5p; 744-5p; 200c-3p; 497-5p; 146a-5p; 15b-5p; 16-5p; 30d-5p; 30a-5p; 625-3p; 451a; 125a-5p; 195-5p; 203a-3p; 143-3p; hsa-let-7a-5p; 148a-3p; 29b-1-5p; 30c-5p; 31-3p; 935; 98-5p; 149-5p; 15a-5p; 423-3p; 33b-5p; 378a-3p; 138-5p; hsa-let-7b-5p; 21-5p; 30a-3p; 23b-3p; 145-5p; 125b-5p; 126-3p
17	influenza A	3.35926E-08	24	17-5p; 155-5p; 19a-3p; 93-5p; 497-5p; 146a-5p; 106b-5p; 15b-5p; 16-5p; 30a-5p; 625-3p; 451a; 125a-5p; 203a-3p; 143-3p; 15a-5p; 106a-5p; 139-5p; 7-5p; 494-3p; 21-5p; 20b-5p; 125b-5p; 126-3p
18	TGF-beta signaling pathway	6.72617E-08	30	17-5p; 20a-5p; 19a-3p; hsa-let-7c-5p; 93-5p; 200c-3p; 146a-5p; 106b-5p; 15b-5p; 451a; 125a-5p; 203a-3p; 135a-5p; 487b-3p; 148a-3p; 29b-1-5p; 31-3p; 130a-3p; 98-5p; 194-5p; 323a-3p; 144-5p; 378a-3p; 590-5p; 27b-3p; 27a-3p; 21-5p; 20b-5p; 145-5p; 126-3p
19	mTOR signaling pathway	1.92112E-07	28	17-5p; 155-5p; 19a-3p; 142-5p; 744-5p; 200c-3p; 497-5p; 15b-5p; 9-3p; 625-3p; 451a; 125a-5p; 195-5p; 203a-3p; 128-3p; 143-3p; 378i; 106a-5p; 378a-3p; 7-5p; 138-5p; 27a-3p; 494-3p; 21-5p; 378d; 145-5p; 125b-5p; 126-3p
20	ErbB signaling pathway	2.04104E-07	38	155-5p; 19a-3p; hsa-let-7c-5p; 744-5p; 200c-3p; 497-5p; 15b-5p; 16-5p; 124-3p; 9-3p; 625-3p; 451a; 125a-5p; 195-5p; 203a-3p; 143-3p; hsa-let-7a-5p; 135a-5p; 543; 487b-3p; 132-3p; 134-5p; 98-5p; 15a-5p; 433-3p; 139-5p; 323a-3p; 33b-5p; 378a-3p; 7-5p; 138-5p; 27a-3p; 494-3p; 30a-3p; 23b-3p; 145-5p; 125b-5p; 126-3p
21	measles	2.04104E-07	32	17-5p; 155-5p; 18a-5p; 19a-3p; 200c-3p; 497-5p; 146a-5p; 106b-5p; 15b-5p; 16-5p; 625-3p; 451a; 125a-5p; 195-5p; 203a-3p; 143-3p; hsa-let-7a-5p; 29b-1-5p; 98-5p; 15a-5p; 26a-5p; 106a-5p; 144-5p; 7-5p; 138-5p; 21-5p; 20b-5p; 340-5p; 23b-3p; 504-5p; 125b-5p; 126-3p
22	focal adhesion	3.07512E-07	43	182-5p; 155-5p; 96-5p; 19a-3p; hsa-let-7c-5p; 142-5p; 744-5p; 200c-3p; 497-5p; 146a-5p; 15b-5p; 486-5p; 16-5p; 376c-3p; 124-3p; 30a-5p; 625-3p; 451a; 125a-5p; 195-5p; 203a-3p; 143-3p; 218-5p; 135a-5p; 148a-3p; 29b-1-5p; 15a-5p; 194-5p; 106a-5p; 139-5p; 1-3p; 144-5p; 33b-5p; 378a-3p; 7-5p; 138-5p; 885-5p; 27b-3p; 494-3p; 23b-3p; 145-5p; 125b-5p; 126-3p
23	p53 signaling pathway	3.16611E-07	47	17-5p; 182-5p; 20a-5p; 155-5p; 96-5p; 18a-5p; 19a-3p; hsa-let-7c-5p; hsa-let-7e-5p; 92a-1-5p; 93-5p; 200c-3p; 19b-3p; 582-5p; 497-5p; 146a-5p; 106b-5p; 15b-5p; 486-5p; 425-5p; 16-5p; 376c-3p; 30a-5p; 195-5p; hsa-let-7a-5p; 543; 148a-3p; 21-3p; 30c-5p; 98-5p; 15a-5p; 95-3p; 26a-5p; 106a-5p; 323a-3p; 144-5p; 18b-5p; 138-5p; 494-3p; hsa-let-7b-5p; 21-5p; 20b-5p; 340-5p; 23b-3p; 145-5p; 504-5p; 125b-5p

24	prolactin signaling pathway	4.67698E-07	34	155-5p; 96-5p; 19a-3p; 19b-1-5p; 142-5p; 19b-3p; 497-5p; 146a-5p; 15b-5p; 124-3p; 9-3p; 451a; 125a-5p; 195-5p; 203a-3p; 143-3p; hsa-let-7a-5p; 543; 29b-1-5p; 30c-5p; 433-3p; 26a-5p; 139-5p; 378a-3p; 7-5p; 138-5p; 27a-3p; 494-3p; 21-5p; 340-5p; 23b-3p; 145-5p; 125b-5p; 126-3p
25	Chagas disease American trypanosomiasis	5.27049E-07	22	155-5p; 18a-5p; 19a-3p; 146a-5p; 15b-5p; 486-5p; 9-3p; 451a; 125a-5p; 203a-3p; 143-3p; 29b-1-5p; 106a-5p; 139-5p; 1-3p; 590-5p; 7-5p; 138-5p; 409-3p; 21-5p; 145-5p; 126-3p
26	neurotrophin signaling pathway	9.15591E-07	36	155-5p; 96-5p; 19a-3p; 142-5p; 744-5p; 200c-3p; 497-5p; 146a-5p; 15b-5p; 16-5p; 376c-3p; 124-3p; 30a-5p; 9-3p; 451a; 125a-5p; 195-5p; 203a-3p; 143-3p; hsa-let-7a-5p; 148a-3p; 30c-5p; 31-3p; 132-3p; 15a-5p; 139-5p; 1-3p; 7-5p; 204-5p; 27a-3p; 494-3p; 369-3p; 21-5p; 340-5p; 125b-5p; 126-3p
27	glioma	9.39666E-07	48	17-5p; 20a-5p; 155-5p; 19a-3p; hsa-let-7c-5p; 142-5p; 93-5p; 200c-3p; 497-5p; 106b-5p; 15b-5p; 486-5p; 425-5p; 16-5p; 376a-3p; 376c-3p; 124-3p; 9-3p; 451a; 125a-5p; 195-5p; 203a-3p; 128-3p; 143-3p; hsa-let-7a-5p; 543; 21-3p; 132-3p; 625-5p; 95-3p; 26a-5p; 106a-5p; 139-5p; 1-3p; 378a-3p; 7-5p; 27a-3p; 494-3p; hsa-let-7b-5p; 21-5p; 20b-5p; 340-5p; 23b-3p; 28-3p; 211-5p; 145-5p; 125b-5p; 126-3p
28	chemokine signaling pathway	1.12757E-06	24	155-5p; 200c-3p; 497-5p; 146a-5p; 124-3p; 9-3p; 451a; 125a-5p; 195-5p; 203a-3p; 143-3p; hsa-let-7a-5p; 135a-5p; 148a-3p; 409-5p; 139-5p; 1-3p; 7-5p; 138-5p; 127-3p; 27a-3p; 494-3p; 23b-3p; 126-3p
29	cell cycle	1.38212E-06	48	92a-3p; 17-5p; 182-5p; 20a-5p; 155-5p; 18a-5p; 92a-1-5p; 93-5p; 200c-3p; 497-5p; 146a-5p; 106b-5p; 15b-5p; 425-5p; 16-5p; 192-5p; 376c-3p; 628-3p; 30a-5p; 451a; 125a-5p; 195-5p; 203a-3p; hsa-let-7a-5p; 423-5p; 148a-3p; 98-5p; 149-5p; 409-5p; 15a-5p; 95-3p; 26a-5p; 106a-5p; 323a-3p; 1-3p; 144-5p; 378a-3p; 590-5p; 885-5p; 27a-3p; 494-3p; hsa-let-7b-5p; 21-5p; 30a-3p; 340-5p; 23b-3p; 145-5p; 125b-5p
30	apoptosis	1.38232E-06	23	17-5p; 155-5p; 96-5p; 19a-3p; 92a-1-5p; 146a-5p; 106b-5p; 30d-5p; 376c-3p; 30a-5p; 451a; 125a-5p; 203a-3p; 143-3p; 30c-5p; 106a-5p; 139-5p; 7-5p; 204-5p; 494-3p; 21-5p; 125b-5p; 126-3p
31	non-small cell lung cancer	1.38232E-06	40	155-5p; 96-5p; 19a-3p; 142-5p; 744-5p; 93-5p; 200c-3p; 497-5p; 146a-5p; 15b-5p; 16-5p; 376c-3p; 124-3p; 9-3p; 451a; 125a-5p; 195-5p; 203a-3p; 128-3p; 143-3p; hsa-let-7a-5p; 30c-5p; 132-3p; 15a-5p; 26a-5p; 106a-5p; 1-3p; 378a-3p; 590-5p; 7-5p; 27a-3p; 494-3p; hsa-let-7b-5p; 21-5p; 20b-5p; 23b-3p; 28-3p; 145-5p; 125b-5p; 126-3p
32	endometrial cancer	1.52526E-06	39	182-5p; 155-5p; 96-5p; 1246; 19a-3p; 142-5p; 92a-1-5p; 744-5p; 200c-3p; 497-5p; 146a-5p; 15b-5p; 25-3p; 425-5p; 30a-5p; 9-3p; 451a; 125a-5p; 195-5p; 203a-3p; 128-3p; 143-3p; hsa-let-7a-5p; 135a-5p; 543; 487b-3p; 30c-5p; 132-3p; 15a-5p; 106a-5p; 378a-3p; 7-5p; 138-5p; 27a-3p; 494-3p; 21-5p; 145-5p; 125b-5p; 126-3p
33	small cell lung cancer	4.4107E-06	37	17-5p; 20a-5p; 155-5p; 19a-3p; 200c-3p; 497-5p; 146a-5p; 15b-5p; 486-5p; 425-5p; 16-5p; 124-3p; 9-3p; 451a; 125a-5p; 195-5p; 203a-3p; 143-3p; 135a-5p; 148a-3p; 98-5p; 15a-5p; 26a-5p; 106a-5p; 139-5p; 144-5p; 378a-3p; 7-5p; 138-5p; 494-3p; 21-5p; 20b-5p; 153-3p; 23b-3p; 28-3p; 125b-5p; 126-3p

MiRPathDB Reactome

No	subcategory	q-value	number of miRs observed	hsa-miRs
1	signal transduction	1.32716E-17	62	17-5p; 182-5p; 20a-5p; 155-5p; 96-5p; 19a-3p; 744-5p; 93-5p; 200c-3p; 19b-3p; 497-5p; 146a-5p; 106b-5p; 15b-5p; 130b-3p; 9-5p; 16-5p; 376c-3p; 628-3p; 124-3p; 30a-5p; 410-3p; 9-3p; 31-5p; 451a; 125a-5p; 195-5p; 203a-3p; 218-5p; 135a-5p; 487b-3p; 148a-3p; 30c-5p; 31-3p; 132-3p; 130a-3p; 98-5p; 149-5p; 15a-5p; 194-5p; 26a-5p; 296-5p; 139-5p; 323a-3p; 423-3p; 1-3p; 144-5p; 33b-5p; 378a-3p; 138-5p; 27b-3p; 204-5p; 27a-3p; 494-3p; 21-5p; 30a-3p; 99b-3p; 454-3p; 23b-3p; 145-5p; 125b-5p; 126-3p
2	intracellular signaling by second messengers	1.04511E-14	55	92a-3p; 17-5p; 182-5p; 20a-5p; 155-5p; 96-5p; 19a-3p; 92a-1-5p; 744-5p; 93-5p; 200c-3p; 19b-3p; 582-5p; 146a-5p; 106b-5p; 15b-5p; 130b-3p; 16-5p; 30d-5p; 376c-3p; 628-3p; 30a-5p; 186-5p; 9-3p; 125a-5p; 195-5p; 203a-3p; 143-3p; 135a-5p; 148a-3p; 30c-5p; 30b-5p; 99a-5p; 101-5p; 31-3p; 132-3p; 130a-3p; 382-3p; 15a-5p; 194-5p; 433-3p; 26a-5p; 106a-5p; 139-5p; 323a-3p; 423-3p; 378a-3p; 138-5p; 494-3p; 21-5p; 20b-5p; 454-3p; 23b-3p; 145-5p; 126-3p

3	PIP3 activates AKT signaling	4.32689E-14	54	92a-3p; 17-5p; 182-5p; 20a-5p; 155-5p; 19a-3p; 744-5p; 93-5p; 200c-3p; 19b-3p; 582-5p; 146a-5p; 106b-5p; 15b-5p; 130b-3p; 425-5p; 16-5p; 30d-5p; 376c-3p; 628-3p; 30a-5p; 186-5p; 9-3p; 125a-5p; 195-5p; 203a-3p; 143-3p; 135a-5p; 148a-3p; 30c-5p; 30b-5p; 99a-5p; 101-5p; 31-3p; 132-3p; 484; 130a-3p; 15a-5p; 194-5p; 433-3p; 26a-5p; 106a-5p; 139-5p; 423-3p; 378a-3p; 138-5p; 27a-3p; 494-3p; 21-5p; 20b-5p; 454-3p; 145-5p; 125b-5p; 126-3p
4	generic transcription pathway	3.46932E-12	84	92a-3p; 17-5p; 182-5p; 20a-5p; 155-5p; 96-5p; 18a-5p; 19a-3p; hsa-let-7c-5p; 17-3p; hsa-let-7e-5p; 92a-1-5p; 744-5p; 93-5p; 200c-3p; 19b-3p; 497-5p; 146a-5p; 766-3p; 181c-5p; 106b-5p; 15b-5p; 130b-3p; 425-5p; 16-5p; 154-5p; 376a-3p; 376c-3p; 628-3p; 30a-5p; 186-5p; 31-5p; 491-5p; 125a-5p; 195-5p; 203a-3p; 143-3p; hsa-let-7a-5p; 135a-5p; 543; 485-3p; 487b-3p; 148a-3p; 21-3p; 30c-5p; 30b-5p; 654-3p; 101-5p; 484; 130a-3p; 134-5p; 98-5p; 149-5p; 382-3p; 15a-5p; 194-5p; 26a-5p; 106a-5p; 323a-3p; 423-3p; 425-3p; 320c; 144-5p; 33b-5p; 378a-3p; 629-5p; 590-5p; 138-5p; 27b-3p; 27a-3p; 494-3p; 376b-3p; hsa-let-7b-5p; 21-5p; 20b-5p; 30a-3p; 454-3p; 23b-3p; 331-5p; 145-5p; 504-5p; 125b-5p; 126-3p; 770-5p
5	gene expression transcription	6.46613E-12	88	92a-3p; 17-5p; 182-5p; 20a-5p; 155-5p; 96-5p; 18a-5p; 1246; 19a-3p; hsa-let-7c-5p; 17-3p; 183-5p; hsa-let-7e-5p; 92a-1-5p; 744-5p; 93-5p; 200c-3p; 19b-3p; 497-5p; 146a-5p; 766-3p; 181c-5p; 106b-5p; 15b-5p; 130b-3p; 425-5p; 16-5p; 154-5p; 30d-5p; 376a-3p; 628-3p; 30a-5p; 410-3p; 186-5p; 127-5p; 31-5p; 491-5p; 125a-5p; 195-5p; 203a-3p; 143-3p; hsa-let-7a-5p; 218-5p; 543; 485-3p; 148a-3p; 21-3p; 30c-5p; 30b-5p; 101-5p; 31-3p; 484; 130a-3p; 134-5p; 98-5p; 149-5p; 382-3p; 15a-5p; 194-5p; 26a-5p; 106a-5p; 323a-3p; 423-3p; 425-3p; 320c; 144-5p; 33b-5p; 378a-3p; 629-5p; 590-5p; 138-5p; 27b-3p; 27a-3p; 494-3p; 376b-3p; hsa-let-7b-5p; 21-5p; 20b-5p; 30a-3p; 454-3p; 23b-3p; 331-5p; 145-5p; 504-5p; 125b-5p; 126-3p; 770-5p
6	signaling by TGF-beta receptor complex	2.66736E-11	37	17-5p; 20a-5p; 18a-5p; 19a-3p; 92a-1-5p; 93-5p; 200c-3p; 19b-3p; 497-5p; 146a-5p; 106b-5p; 15b-5p; 130b-3p; 16-5p; 154-5p; 376c-3p; 31-5p; 125a-5p; 195-5p; 148a-3p; 30c-5p; 99a-5p; 484; 130a-3p; 98-5p; 15a-5p; 323a-3p; 144-5p; 629-5p; 590-5p; 27b-3p; 27a-3p; 369-3p; 21-5p; 454-3p; 23b-3p; 145-5p
7	PTEN regulation	6.42009E-11	37	92a-3p; 17-5p; 20a-5p; 19a-3p; 92a-1-5p; 744-5p; 93-5p; 200c-3p; 19b-3p; 106b-5p; 130b-3p; 16-5p; 628-3p; 186-5p; 9-3p; 451a; 143-3p; 487b-3p; 148a-3p; 30c-5p; 30b-5p; 99a-5p; 484; 130a-3p; 382-3p; 15a-5p; 26a-5p; 106a-5p; 423-3p; 378a-3p; 138-5p; 494-3p; 21-5p; 20b-5p; 454-3p; 92b-5p; 126-3p
8	vesicle-mediated transport	1.02977E-10	29	92a-3p; 17-5p; 20a-5p; 155-5p; 19a-3p; 93-5p; 106b-5p; 15b-5p; 130b-3p; 99a-3p; 16-5p; 124-3p; 30a-5p; 186-5p; 299-5p; 451a; 195-5p; 148a-3p; 30c-5p; 130a-3p; 149-5p; 382-3p; 15a-5p; 1-3p; 132-5p; 432-5p; 20b-5p; 99b-3p; 1296-5p
9	RNA polymerase II transcription	1.77504E-10	82	92a-3p; 17-5p; 182-5p; 20a-5p; 155-5p; 96-5p; 18a-5p; 19a-3p; hsa-let-7c-5p; 17-3p; 183-5p; hsa-let-7e-5p; 92a-1-5p; 744-5p; 93-5p; 200c-3p; 19b-3p; 497-5p; 146a-5p; 766-3p; 181c-5p; 106b-5p; 15b-5p; 130b-3p; 425-5p; 16-5p; 154-5p; 376a-3p; 376c-3p; 628-3p; 30a-5p; 410-3p; 186-5p; 31-5p; 491-5p; 125a-5p; 195-5p; 203a-3p; 143-3p; hsa-let-7a-5p; 135a-5p; 543; 485-3p; 148a-3p; 21-3p; 30c-5p; 30b-5p; 654-3p; 101-5p; 484; 130a-3p; 134-5p; 98-5p; 149-5p; 15a-5p; 26a-5p; 106a-5p; 323a-3p; 423-3p; 320c; 1-3p; 144-5p; 33b-5p; 378a-3p; 629-5p; 590-5p; 138-5p; 27b-3p; 27a-3p; 494-3p; 376b-3p; hsa-let-7b-5p; 21-5p; 20b-5p; 30a-3p; 454-3p; 23b-3p; 145-5p; 504-5p; 125b-5p; 126-3p; 770-5p
10	transcriptional activity of SMAD2 SMAD3 SMAD4 heterotrimer	1.83271E-10	33	17-5p; 20a-5p; 18a-5p; 19a-3p; 92a-1-5p; 15b-5p; 130b-3p; 16-5p; 376c-3p; 31-5p; 125a-5p; 195-5p; 148a-3p; 30c-5p; 30b-5p; 99a-5p; 484; 130a-3p; 98-5p; 15a-5p; 129-2-3p; 323a-3p; 144-5p; 378a-3p; 629-5p; 590-5p; 27b-3p; 27a-3p; 369-3p; 21-5p; 454-3p; 23b-3p; 145-5p
11	signaling by TGF-beta family members	2.82943E-10	37	92a-3p; 17-5p; 20a-5p; 155-5p; 18a-5p; 19a-3p; 93-5p; 200c-3p; 19b-3p; 497-5p; 106b-5p; 15b-5p; 16-5p; 154-5p; 376c-3p; 31-5p; 195-5p; 203a-3p; 135a-5p; 148a-3p; 29b-1-5p; 99a-5p; 31-3p; 130a-3p; 15a-5p; 323a-3p; 144-5p; 629-5p; 590-5p; 27b-3p; 27a-3p; 369-3p; 21-5p; 20b-5p; 454-3p; 23b-3p; 145-5p
12	immune system	3.9251E-10	41	17-5p; 20a-5p; 155-5p; 96-5p; 18a-5p; 19a-3p; 17-3p; 93-5p; 200c-3p; 582-5p; 497-5p; 146a-5p; 106b-5p; 15b-5p; 16-5p; 30a-5p; 9-3p; 451a; 125a-5p; 195-5p; 203a-3p; 143-3p; 218-5p; 543; 148a-3p; 30c-5p; 31-3p; 132-3p; 130a-3p; 98-5p; 15a-5p; 95-3p; 139-5p; 423-3p; 1-3p; 21-5p; 23b-3p; 145-5p; 125b-5p; 126-3p; 1296-5p

13	transcriptional regulation by TP53	5.53929E-10	47	92a-3p; 17-5p; 182-5p; 20a-5p; 155-5p; 96-5p; 19a-3p; hsa-let-7c-5p; 17-3p; hsa-let-7e-5p; 92a-1-5p; 744-5p; 93-5p; 200c-3p; 19b-3p; 497-5p; 106b-5p; 15b-5p; 130b-3p; 16-5p; 628-3p; 30a-5p; 186-5p; 625-3p; 125a-5p; 195-5p; hsa-let-7a-5p; 148a-3p; 99a-5p; 484; 130a-3p; 149-5p; 382-3p; 15a-5p; 26a-5p; 106a-5p; 323a-3p; 423-3p; 494-3p; hsa-let-7b-5p; 21-5p; 20b-5p; 30a-3p; 454-3p; 23b-3p; 504-5p; 125b-5p
14	cellular senescence	1.00738E-09	54	17-5p; 20a-5p; 155-5p; hsa-let-7c-5p; hsa-let-7e-5p; 744-5p; 93-5p; 200c-3p; 497-5p; 146a-5p; 181c-5p; 106b-5p; 15b-5p; 130b-3p; 16-5p; 30d-5p; 376c-3p; 124-3p; 186-5p; 9-3p; 299-5p; 31-5p; 451a; 125a-5p; 195-5p; 203a-3p; hsa-let-7a-5p; 218-5p; 423-5p; 543; 487b-3p; 148a-3p; 30c-5p; 30b-5p; 654-3p; 130a-3p; 98-5p; 15a-5p; 194-5p; 26a-5p; 106a-5p; 323a-3p; 320c; 144-5p; 629-5p; 138-5p; 27a-3p; 494-3p; hsa-let-7b-5p; 21-5p; 20b-5p; 454-3p; 145-5p; 125b-5p
15	ESR-mediated signaling	1.00738E-09	53	92a-3p; 17-5p; 182-5p; 155-5p; 96-5p; 19a-3p; hsa-let-7c-5p; 17-3p; 744-5p; 93-5p; 200c-3p; 497-5p; 15b-5p; 130b-3p; 9-5p; 16-5p; 376c-3p; 30a-5p; 186-5p; 299-5p; 31-5p; 451a; 195-5p; 143-3p; hsa-let-7a-5p; 135a-5p; 543; 148a-3p; 656-3p; 30c-5p; 99a-5p; 654-3p; 132-3p; 98-5p; 382-3p; 15a-5p; 194-5p; 26a-5p; 139-5p; 323a-3p; 1-3p; 33b-5p; 378a-3p; 138-5p; 27b-3p; 27a-3p; 494-3p; hsa-let-7b-5p; 21-5p; 30a-3p; 145-5p; 92b-5p; 126-3p
16	disease	1.09641E-09	39	92a-3p; 182-5p; 155-5p; 19a-3p; 744-5p; 200c-3p; 15b-5p; 425-5p; 16-5p; 186-5p; 31-5p; 125a-5p; 195-5p; 143-3p; hsa-let-7a-5p; 218-5p; 485-3p; 148a-3p; 30c-5p; 31-3p; 132-3p; 484; 130a-3p; 409-5p; 15a-5p; 26a-5p; 296-5p; 139-5p; 423-3p; 27b-3p; 27a-3p; 494-3p; 30a-3p; 23b-3p; 145-5p; 125b-5p; 126-3p; 92b-3p; 1296-5p
17	membrane trafficking	1.43308E-09	30	92a-3p; 17-5p; 20a-5p; 155-5p; 19a-3p; 142-3p; 93-5p; 106b-5p; 15b-5p; 130b-3p; 99a-3p; 16-5p; 124-3p; 30a-5p; 186-5p; 299-5p; 451a; 195-5p; 148a-3p; 30c-5p; 130a-3p; 149-5p; 382-3p; 15a-5p; 1-3p; 132-5p; 432-5p; 20b-5p; 99b-3p; 1296-5p
18	diseases of signal transduction	4.21323E-09	40	92a-3p; 182-5p; 155-5p; 19a-3p; hsa-let-7c-5p; 744-5p; 200c-3p; 19b-3p; 582-5p; 15b-5p; 130b-3p; 425-5p; 376c-3p; 451a; 125a-5p; 195-5p; 143-3p; 218-5p; 135a-5p; 148a-3p; 656-3p; 30c-5p; 144-3p; 31-3p; 132-3p; 130a-3p; 194-5p; 433-3p; 26a-5p; 106a-5p; 139-5p; 323a-3p; 378a-3p; 27a-3p; 21-5p; 30a-3p; 454-3p; 145-5p; 125b-5p; 126-3p
19	interleukin-4 and interleukin-13 signaling	4.49125E-09	42	17-5p; 155-5p; 96-5p; 19a-3p; hsa-let-7c-5p; 93-5p; 200c-3p; 582-5p; 497-5p; 146a-5p; 15b-5p; 9-5p; 16-5p; 186-5p; 625-3p; 451a; 125a-5p; 195-5p; 203a-3p; 143-3p; 135a-5p; 148a-3p; 29b-1-5p; 30c-5p; 654-3p; 134-5p; 98-5p; 495-3p; 15a-5p; 95-3p; 26a-5p; 106a-5p; 139-5p; 323a-3p; 33b-5p; 378a-3p; 138-5p; 494-3p; 21-5p; 145-5p; 125b-5p; 126-3p
20	signaling by nuclear receptors	5.81788E-09	49	92a-3p; 17-5p; 182-5p; 155-5p; 19a-3p; hsa-let-7c-5p; 744-5p; 93-5p; 200c-3p; 582-5p; 497-5p; 15b-5p; 9-5p; 16-5p; 376c-3p; 30a-5p; 299-5p; 31-5p; 451a; 195-5p; 143-3p; hsa-let-7a-5p; 135a-5p; 543; 148a-3p; 656-3p; 30c-5p; 99a-5p; 132-3p; 98-5p; 382-3p; 15a-5p; 194-5p; 106a-5p; 139-5p; 320c; 1-3p; 33b-5p; 378a-3p; 138-5p; 27b-3p; 27a-3p; 494-3p; hsa-let-7b-5p; 21-5p; 30a-3p; 145-5p; 92b-5p; 126-3p
21	TP53 regulates metabolic genes	7.36013E-09	34	17-5p; 20a-5p; 19a-3p; 17-3p; hsa-let-7e-5p; 744-5p; 93-5p; 497-5p; 106b-5p; 130b-3p; 16-5p; 628-3p; 186-5p; 31-5p; 451a; 143-3p; hsa-let-7a-5p; 148a-3p; 99a-5p; 484; 130a-3p; 382-3p; 26a-5p; 106a-5p; 320c; 409-3p; 494-3p; hsa-let-7b-5p; 21-5p; 20b-5p; 454-3p; 23b-3p; 92b-5p; 126-3p
22	oncogene induced senescence	1.07326E-08	42	17-5p; 20a-5p; 155-5p; 18a-5p; 19a-3p; 93-5p; 200c-3p; 582-5p; 497-5p; 766-3p; 106b-5p; 15b-5p; 130b-3p; 410-3p; 9-3p; 299-5p; 31-5p; 451a; 195-5p; hsa-let-7a-5p; 218-5p; 543; 148a-3p; 30c-5p; 99a-5p; 654-3p; 130a-3p; 149-5p; 382-3p; 15a-5p; 26a-5p; 106a-5p; 378a-3p; 590-5p; 494-3p; hsa-let-7b-5p; 21-5p; 20b-5p; 454-3p; 23b-3p; 145-5p; 125b-5p
23	cytokine signaling in immune system	1.42651E-08	42	155-5p; 96-5p; hsa-let-7c-5p; hsa-let-7e-5p; 200c-3p; 497-5p; 146a-5p; 15b-5p; 16-5p; 124-3p; 186-5p; 9-3p; 451a; 125a-5p; 195-5p; 203a-3p; 143-3p; hsa-let-7a-5p; 218-5p; 135a-5p; 148a-3p; 30c-5p; 132-3p; 98-5p; 15a-5p; 95-3p; 194-5p; 433-3p; 26a-5p; 139-5p; 423-3p; 1-3p; 33b-5p; 378a-3p; 138-5p; 494-3p; 21-5p; 30a-3p; 23b-3p; 145-5p; 125b-5p; 126-3p

MiRPathDB WikiPathways

No	subcategory	q-value	number of miRs observed	hsa-miRs

1	integrated breast cancer pathway	5.03182E-20	69	92a-3p; 17-5p; 182-5p; 20a-5p; 155-5p; 96-5p; 18a-5p; 19a-3p; hsa-let-7c-5p; 142-5p; 92a-1-5p; 744-5p; 16-2-3p; 93-5p; 200c-3p; 582-5p; 497-5p; 146a-5p; 106b-5p; 15b-5p; 9-5p; 486-5p; 425-5p; 30d-5p; 381-3p; 376c-3p; 30a-5p; 9-3p; 451a; 125a-5p; 203a-3p; 128-3p; 143-3p; hsa-let-7a-5p; 135a-5p; 543; 487b-3p; 148a-3p; 21-3p; 30c-5p; 132-3p; 98-5p; 149-5p; 758-3p; 15a-5p; 26a-5p; 296-5p; 106a-5p; 139-5p; 33b-5p; 18b-5p; 378a-3p; 590-5p; 7-5p; 138-5p; 885-5p; 27b-3p; 204-5p; 27a-3p; 383-5p; 494-3p; 21-5p; 20b-5p; 153-3p; 23b-3p; 145-5p; 370-3p; 99b-5p; 126-3p
2	TGF-beta Signaling Pathway	5.03182E-20	68	17-5p; 20a-5p; 155-5p; 18a-5p; 19a-3p; 19b-1-5p; hsa-let-7c-5p; 142-5p; 92a-1-5p; 93-5p; 200c-3p; 19b-3p; 582-5p; 497-5p; 146a-5p; 106b-5p; 15b-5p; 16-5p; 154-5p; 376c-3p; 628-3p; 30a-5p; 410-3p; 31-5p; 625-3p; 655-3p; 451a; 491-5p; 125a-5p; 195-5p; 203a-3p; 143-3p; hsa-let-7a-5p; 218-5p; 135a-5p; 543; 487b-3p; 148a-3p; 29b-1-5p; 30c-5p; 144-3p; 654-3p; 130a-3p; 15a-5p; 95-3p; 433-3p; 129-2-3p; 106a-5p; 139-5p; 323a-3p; 144-5p; 33b-5p; 378a-3p; 590-5p; 138-5p; 127-3p; 27b-3p; 27a-3p; 494-3p; 369-3p; hsa-let-7b-5p; 21-5p; 20b-5p; 30a-3p; 23b-3p; 145-5p; 370-3p; 126-3p
3	VEGFA-VEGFR2 Signaling Pathway	8.92E-17	63	17-5p; 182-5p; 20a-5p; 155-5p; 96-5p; 18a-5p; 19a-3p; hsa-let-7c-5p; 142-5p; 93-5p; 200c-3p; 497-5p; 146a-5p; 106b-5p; 15b-5p; 9-5p; 486-5p; 425-5p; 381-3p; 376c-3p; 124-3p; 30a-5p; 9-3p; 625-3p; 451a; 125a-5p; 195-5p; 203a-3p; 128-3p; 143-3p; 218-5p; 135a-5p; 543; 148a-3p; 378c; 30c-5p; 654-3p; 31-3p; 132-3p; 134-5p; 378i; 15a-5p; 487a-3p; 296-5p; 106a-5p; 139-5p; 1-3p; 144-5p; 33b-5p; 18b-5p; 378a-3p; 138-5p; 409-3p; 204-5p; 27a-3p; 494-3p; 369-3p; 21-5p; 340-5p; 378d; 23b-3p; 145-5p; 126-3p
4	DNA damage response only ATM dependent	1.44131E-16	73	92a-3p; 17-5p; 182-5p; 20a-5p; 155-5p; 96-5p; 18a-5p; 19a-3p; 142-5p; 92a-1-5p; 744-5p; 93-5p; 200c-3p; 19b-3p; 582-5p; 497-5p; 146a-5p; 106b-5p; 15b-5p; 9-5p; 425-5p; 16-5p; 154-5p; 376c-3p; 124-3p; 30a-5p; 451a; 125a-5p; 195-5p; 203a-3p; 143-3p; hsa-let-7a-5p; 218-5p; 4286; 135a-5p; 543; 485-3p; 487b-3p; 148a-3p; 21-3p; 29b-1-5p; 30c-5p; 144-3p; 101-5p; 130a-3p; 98-5p; 409-5p; 15a-5p; 95-3p; 433-3p; 26a-5p; 106a-5p; 139-5p; 323a-3p; 144-5p; 33b-5p; 378a-3p; 590-5p; 7-5p; 138-5p; 127-3p; 885-5p; 27b-3p; 27a-3p; 494-3p; hsa-let-7b-5p; 21-5p; 30a-3p; 340-5p; 153-3p; 145-5p; 125b-5p; 126-3p
5	chromosomal and microsatellite instability in colorectal cancer	3.85426E-15	63	17-5p; 20a-5p; 155-5p; 96-5p; 18a-5p; 1246; 19a-3p; 142-5p; 92a-1-5p; 93-5p; 200c-3p; 582-5p; 497-5p; 146a-5p; 106b-5p; 15b-5p; 425-5p; 16-5p; 381-3p; 30a-5p; 655-3p; 451a; 125a-5p; 195-5p; 203a-3p; 143-3p; hsa-let-7a-5p; 4286; 135a-5p; 487b-3p; 148a-3p; 29b-1-5p; 378c; 30c-5p; 144-3p; 101-5p; 132-3p; 130a-3p; 378i; 15a-5p; 95-3p; 106a-5p; 139-5p; 323a-3p; 423-3p; 33b-5p; 378a-3p; 590-5p; 7-5p; 138-5p; 409-3p; 885-5p; 27a-3p; 494-3p; 21-5p; 20b-5p; 378d; 145-5p; 370-3p; 504-5p; 125b-5p; 126-3p; 323b-3p
6	epithelial to mesenchymal transition in colorectal cancer	3.85426E-15	48	155-5p; 18a-5p; 19a-3p; 142-5p; 200c-3p; 497-5p; 146a-5p; 15b-5p; 9-5p; 486-5p; 154-5p; 376c-3p; 124-3p; 30a-5p; 410-3p; 9-3p; 625-3p; 451a; 491-5p; 125a-5p; 195-5p; 203a-3p; 128-3p; 143-3p; 543; 487b-3p; 148a-3p; 29b-1-5p; 30c-5p; 144-3p; 132-3p; 378i; 15a-5p; 433-3p; 139-5p; 378a-3p; 590-5p; 7-5p; 138-5p; 27b-3p; 204-5p; 27a-3p; 494-3p; 21-5p; 378d; 145-5p; 125b-5p; 126-3p
7	hepatitis C and hepatocellular carcinoma	3.85426E-15	54	17-5p; 20a-5p; 155-5p; 18a-5p; 19a-3p; hsa-let-7c-5p; 142-5p; 93-5p; 582-5p; 497-5p; 146a-5p; 106b-5p; 15b-5p; 425-5p; 16-5p; 30d-5p; 124-3p; 30a-5p; 299-5p; 625-3p; 451a; 125a-5p; 195-5p; 203a-3p; 128-3p; 143-3p; hsa-let-7a-5p; 218-5p; 135a-5p; 148a-3p; 21-3p; 29b-1-5p; 30c-5p; 144-3p; 98-5p; 15a-5p; 95-3p; 433-3p; 26a-5p; 106a-5p; 323a-3p; 378a-3p; 590-5p; 138-5p; 885-5p; 27a-3p; 494-3p; hsa-let-7b-5p; 21-5p; 20b-5p; 28-3p; 145-5p; 125b-5p; 126-3p
8	TGF-beta Receptor Signaling	9.75275E-15	42	17-5p; 20a-5p; 155-5p; 19a-3p; 93-5p; 200c-3p; 19b-3p; 582-5p; 497-5p; 146a-5p; 106b-5p; 15b-5p; 486-5p; 154-5p; 376c-3p; 628-3p; 125a-5p; 203a-3p; 143-3p; hsa-let-7a-5p; 135a-5p; 543; 148a-3p; 29b-1-5p; 30c-5p; 144-3p; 130a-3p; 15a-5p; 106a-5p; 139-5p; 323a-3p; 144-5p; 590-5p; 138-5p; 27b-3p; 27a-3p; 21-5p; 20b-5p; 30a-3p; 145-5p; 370-3p; 125b-5p
9	leptin signaling pathway	1.35016E-14	54	106b-3p; 182-5p; 155-5p; 19a-3p; hsa-let-7c-5p; 142-5p; 200c-3p; 582-5p; 497-5p; 146a-5p; 15b-5p; 9-5p; 486-5p; 425-5p; 16-5p; 124-3p; 30a-5p; 9-3p; 625-3p; 451a; 125a-5p; 195-5p; 203a-3p; 128-3p; 143-3p; hsa-let-7a-5p; 135a-5p; 543; 148a-3p; 21-3p; 378c; 30c-5p; 329-3p; 378i; 15a-5p; 487a-3p; 433-3p; 106a-5p; 139-5p; 144-5p; 33b-5p; 378a-3p; 7-5p; 138-5p; 204-5p; 27a-3p; 494-3p; 369-3p; 21-5p; 378d; 23b-3p; 145-5p; 125b-5p; 126-3p

10	extracellular vesicle-mediated signaling in recipient cells	2.11888E-14	42	155-5p; 18a-5p; 19a-3p; 142-5p; 744-5p; 497-5p; 146a-5p; 425-5p; 16-5p; 154-5p; 376c-3p; 30a-5p; 491-5p; 125a-5p; 128-3p; 143-3p; hsa-let-7a-5p; 218-5p; 135a-5p; 543; 487b-3p; 148a-3p; 29b-1-5p; 378c; 144-3p; 132-3p; 130a-3p; 378i; 433-3p; 26a-5p; 139-5p; 323a-3p; 144-5p; 590-5p; 409-3p; 27b-3p; 27a-3p; 21-5p; 378d; 145-5p; 125b-5p; 126-3p
11	RAC1 PAK1 p38 MMP2 pathway	2.57742E-14	45	106b-3p; 155-5p; 96-5p; 19a-3p; 142-5p; 92a-1-5p; 200c-3p; 497-5p; 146a-5p; 124-3p; 9-3p; 625-3p; 451a; 125a-5p; 203a-3p; 143-3p; hsa-let-7a-5p; 135a-5p; 543; 487b-3p; 148a-3p; 30c-5p; 132-3p; 134-5p; 15a-5p; 487a-3p; 433-3p; 106a-5p; 139-5p; 323a-3p; 33b-5p; 378a-3p; 7-5p; 138-5p; 885-5p; 27a-3p; 494-3p; 21-5p; 30a-3p; 23b-3p; 28-3p; 145-5p; 370-3p; 125b-5p; 126-3p
12	bladder cancer	6.04679E-14	54	17-5p; 20a-5p; 155-5p; 96-5p; 19a-3p; hsa-let-7c-5p; 142-5p; 92a-1-5p; 16-2-3p; 93-5p; 497-5p; 146a-5p; 106b-5p; 15b-5p; 130b-3p; 25-3p; 486-5p; 425-5p; 16-5p; 376c-3p; 30a-5p; 9-3p; 451a; 491-5p; 125a-5p; 195-5p; 203a-3p; 143-3p; hsa-let-7a-5p; 135a-5p; 487b-3p; 148a-3p; 30c-5p; 132-3p; 130a-3p; 134-5p; 98-5p; 487a-3p; 95-3p; 106a-5p; 1-3p; 33b-5p; 18b-5p; 378a-3p; 138-5p; 27a-3p; 494-3p; 21-5p; 20b-5p; 30a-3p; 23b-3p; 145-5p; 125b-5p; 126-3p
13	EGF EGFR signaling pathway	1.5309E-13	50	106b-3p; 17-5p; 182-5p; 20a-5p; 155-5p; 18a-5p; 19a-3p; 142-5p; 744-5p; 93-5p; 200c-3p; 497-5p; 146a-5p; 181c-5p; 106b-5p; 15b-5p; 486-5p; 376c-3p; 124-3p; 30a-5p; 9-3p; 451a; 125a-5p; 203a-3p; 128-3p; 143-3p; 135a-5p; 543; 148a-3p; 30c-5p; 99a-5p; 654-3p; 31-3p; 132-3p; 433-3p; 106a-5p; 139-5p; 33b-5p; 181d-5p; 378a-3p; 138-5p; 27a-3p; 494-3p; 21-5p; 20b-5p; 153-3p; 23b-3p; 145-5p; 125b-5p; 126-3p
14	pancreatic adenocarcinoma pathway	1.54919E-13	58	17-5p; 20a-5p; 155-5p; 96-5p; 18a-5p; 19a-3p; 142-5p; 92a-1-5p; 744-5p; 200c-3p; 582-5p; 497-5p; 146a-5p; 106b-5p; 15b-5p; 486-5p; 16-5p; 124-3p; 30a-5p; 9-3p; 625-3p; 451a; 491-5p; 125a-5p; 195-5p; 203a-3p; 128-3p; 143-3p; hsa-let-7a-5p; 148a-3p; 21-3p; 29b-1-5p; 378c; 30c-5p; 654-3p; 132-3p; 134-5p; 378i; 15a-5p; 95-3p; 106a-5p; 323a-3p; 378a-3p; 590-5p; 7-5p; 138-5p; 27a-3p; 494-3p; 369-3p; 21-5p; 20b-5p; 378d; 23b-3p; 145-5p; 370-3p; 504-5p; 125b-5p; 126-3p
15	IL-5 signaling pathway	1.64454E-13	40	182-5p; 155-5p; 96-5p; 19a-3p; 744-5p; 200c-3p; 497-5p; 146a-5p; 15b-5p; 425-5p; 16-5p; 376c-3p; 9-3p; 451a; 125a-5p; 195-5p; 203a-3p; 143-3p; hsa-let-7a-5p; 135a-5p; 487b-3p; 148a-3p; 30c-5p; 15a-5p; 487a-3p; 433-3p; 139-5p; 323a-3p; 33b-5p; 378a-3p; 7-5p; 124-5p; 27a-3p; 494-3p; 21-5p; 99b-3p; 23b-3p; 145-5p; 125b-5p; 126-3p
16	viral acute myocarditis	1.06889E-12	36	155-5p; 96-5p; 19a-3p; hsa-let-7c-5p; 744-5p; 582-5p; 146a-5p; 106b-5p; 15b-5p; 9-5p; 30a-5p; 9-3p; 451a; 125a-5p; 203a-3p; 143-3p; hsa-let-7a-5p; 148a-3p; 21-3p; 29b-1-5p; 132-3p; 487a-3p; 194-5p; 26a-5p; 106a-5p; 139-5p; 33b-5p; 138-5p; 409-3p; 885-5p; 204-5p; 494-3p; 21-5p; 125b-5p; 126-3p; 323b-3p
17	oncostatin M signaling pathway	2.56324E-12	46	17-5p; 155-5p; 19a-3p; 142-5p; 200c-3p; 497-5p; 146a-5p; 16-5p; 30d-5p; 381-3p; 124-3p; 30a-5p; 9-3p; 299-5p; 625-3p; 451a; 125a-5p; 203a-3p; 128-3p; 143-3p; 4286; 148a-3p; 29b-1-5p; 30c-5p; 30b-5p; 132-3p; 134-5p; 487a-3p; 433-3p; 106a-5p; 139-5p; 33b-5p; 378a-3p; 7-5p; 138-5p; 27b-3p; 27a-3p; 494-3p; 21-5p; 20b-5p; 23b-3p; 28-3p; 145-5p; 504-5p; 125b-5p; 126-3p

*p-value after the Benjamini-Hochberg adjustment
