# Supplementary material for: A Set of 17 microRNAs Common for Brain and Cerebrospinal Fluid Differentiates Primary Central Nervous System Lymphoma from Non-Malignant Brain Tumors
Source: Biomolecules. 2021 Sep 21;11(9):1395. doi: 10.3390/biom11091395 (PMC8466839; doi:10.3390/biom11091395)
Supplement: Supplementary file 1 [file biomolecules-11-01395-s001.zip › Table S2.pdf]

Supplementary Table S2

Databases used for the microRNA enrichment analysis and by the annotation tool (miEAA)

Database	Description categories
Gene Ontology	Annotations derived over miRTarBase (indirect)
Gene Ontology	Annotations
KEGG	Pathways
miRandola	exRNA forms
miRBase	Chromosomal location
miRBase	Conservation
miRBase	Confidence
miRBase	Seed family
miRPathDB	GO Biological process
miRPathDB	GO Cellular component
miRPathDB	GO Molecular function
iRPathDB	KEGG
miRPathDB	Reactome
miRPathDB	WikiPathways
miRTarBase	Target genes
miRWalk	Diseases
miRWalk	Gene Ontology
miRWalk	Organs
miRWalk	Pathways
MNDR	Diseases
NPInter	Interactions
Published age/gender	Gender/Age
Published cell specific	Cell-type specific
Published diseases	Published Diseases
Published Immune Cells	Immune cells
RNALocate	Localization
SM2miR	Drugs
TissueAtlas	Expressed in tissue
