# Supplementary material for: A Set of 17 microRNAs Common for Brain and Cerebrospinal Fluid Differentiates Primary Central Nervous System Lymphoma from Non-Malignant Brain Tumors
Source: Biomolecules. 2021 Sep 21;11(9):1395. doi: 10.3390/biom11091395 (PMC8466839; doi:10.3390/biom11091395)
Supplement: Supplementary file 1 [file biomolecules-11-01395-s001.zip › Table S3.pdf]

Supplementary Table S3

A list of CSF and tumor miRs significantly differentiating CNS DLBCL and n-ML diseases.

CSF miRs			
**miRs**	**q-value***	**FC**	**AUC**
hsa-miR-9-3p	0.0165	0.14	0.84
hsa-miR-9-5p	0.0299	0.15	0.79
hsa-miR-15a-5p	0.0095	5.31	0.58
hsa-miR-15b-5p	0.0198	5.24	0.79
hsa-miR-16-5p	0.0067	4.34	0.72
hsa-miR-17-3p	0.0198	16.11	0.73
hsa-miR-19a-3p	8.56E-04	4.79	0.96
hsa-miR-19b-3p	0.0095	3.16	0.73
hsa-miR-20a-5p	0.0334	5.71	0.68
hsa-miR-21-5p	0.0165	3.40	0.76
hsa-miR-25-3p	0.0260	12.46	0.37
hsa-miR-26a-5p	0.0337	0.40	0.76
hsa-miR-92a-3p	0.0219	6.08	0.61
hsa-miR-106b-3p	0.0253	19.51	0.32
hsa-miR-148a-3p	0.0095	9.45	0.70
hsa-miR-210-3p	0.0198	10.48	0.68
hsa-miR-221-3p	9.46E-03	4,75	0.73
hsa-miR-423-5p	0.0115	4.15	0.68
**Tumor miRs**			
hsa-miR-1-3p	1.36E-02	0.29	0.76
hsa-miR-7-5p	8.24E-03	0.36	0.82
hsa-let-7a-5p	1.95E-03	0.53	0.9
hsa-let-7b-5p	2.32E-02	0.55	0.84
hsa-let-7c-5p	1.17E-07	0.21	1
hsa-let-7e-5p	7.85E-07	0.28	0.98
hsa-miR-9-3p	9.33E-04	0.24	0.86
hsa-miR-9-5p	8.62E-05	0.22	0.89
hsa-miR-15a-5p	1.32E-02	2.05	0.81
hsa-miR-15b-3p	1.64E-06	6.83	0.87
hsa-miR-15b-5p	8.62E-05	5.23	0.87
hsa-miR-16-2-3p	2.89E-06	4.87	0.95
hsa-miR-16-5p	4.93E-04	2.57	0.93
hsa-miR-17-3p	8.01E-07	5.37	0.9
hsa-miR-17-5p	1.50E-09	6.70	0.99
hsa-miR-18a-5p	3.96E-09	5.87	0.99
hsa-miR-18b-5p	1.99E-02	2.45	0.715
hsa-miR-19a-3p	1.16E-07	6.61	0.96
hsa-miR-19b-1-5p	2.97E-07	9.96	0.94
hsa-miR-19b-3p	1.10E-05	5.06	0.95
hsa-miR-20a-5p	1.50E-09	8.96	0.98
hsa-miR-21-3p	4.83E-03	4.65	0.905
hsa-miR-21-5p	3.11E-02	2.78	0.83
hsa-miR-25-3p	4.49E-04	3.45	0.83
hsa-miR-26a-5p	9.68E-03	0.65	0.89
hsa-miR-26b-3p	9.77E-03	2.22	0.89
hsa-miR-27a-3p	2.73E-02	1.84	0.79

hsa-miR-27b-3p	4.04E-02	0.60	0.8
hsa-miR-30a-3p	1.97E-02	0.38	0.8
hsa-miR-30a-5p	5.87E-04	0.36	0.91
hsa-miR-30b-5p	1.80E-02	2.77	0.58
hsa-miR-30c-5p	3.01E-03	0.58	0.84
hsa-miR-30d-5p	1.36E-03	3.79	0.75
hsa-miR-31-3p	5.44E-03	0.31	0.88
hsa-miR-31-5p	5.50E-04	0.33	0.91
hsa-miR-32-5p	2.41E-02	2.37	0.81
hsa-miR-92a-1-5p	6.70E-06	18.41	0.72
hsa-miR-92a-3p	1.52E-08	5.77	0.98
hsa-miR-92b-3p	3.09E-02	0.44	0.76
hsa-miR-92b-5p	2.48E-02	0.44	0.74
hsa-miR-93-3p	2.73E-02	2.14	0.78
hsa-miR-93-5p	2.00E-05	3.34	1
hsa-miR-95-3p	6.73E-03	0.36	0.87
hsa-miR-96-5p	9.36E-10	12.18	0.9
hsa-miR-98-5p	3.20E-03	0.54	0.9
hsa-miR-99a-3p	1.75E-04	0.29	0.97
hsa-miR-99a-5p	5.30E-03	0.32	0.87
hsa-miR-99b-3p	1.99E-02	0.51	0.9
hsa-miR-99b-5p	3.99E-02	0.56	0.78
hsa-miR-101-5p	4.16E-03	0.40	0.89
hsa-miR-106a-5p	3.22E-02	2.17	0.71
hsa-miR-106b-3p	8.02E-10	7.09	1
hsa-miR-106b-5p	1.16E-04	3.39	0.91
hsa-miR-124-3p	3.81E-04	0.12	0.9
hsa-miR-124-5p	1.97E-02	0.23	0.825
hsa-miR-125a-5p	1.36E-03	0.43	0.92
hsa-miR-125b-2-3p	5.02E-04	0.30	0.96
hsa-miR-125b-5p	4.47E-02	0.53	0.84
hsa-miR-126-3p	3.11E-02	0.58	0.8
hsa-miR-127-3p	1.21E-02	0.38	0.85
hsa-miR-127-5p	6.18E-04	0.29	0.93
hsa-miR-128-3p	1.36E-03	0.27	0.86
hsa-miR-129-1-3p	2.81E-03	0.22	0.85
hsa-miR-129-2-3p	7.36E-03	0.30	0.84
hsa-miR-129-5p	2.48E-03	0.29	0.88
hsa-miR-130a-3p	4.16E-03	0.52	0.91
hsa-miR-130b-3p	1.75E-04	3.62	0.82
hsa-miR-130b-5p	1.75E-04	4.13	0.87
hsa-miR-132-3p	4.27E-03	0.40	0.86
hsa-miR-132-5p	8.43E-03	0.38	0.82
hsa-miR-134-5p	4.85E-03	0.35	0.91
hsa-miR-135a-5p	2.55E-03	0.26	0.88
hsa-miR-136-3p	1.26E-03	0.24	0.82
hsa-miR-136-5p	2.37E-02	0.42	0.73
hsa-miR-137	4.83E-03	0.23	0.85
hsa-miR-138-5p	2.13E-02	0.34	0.77
hsa-miR-139-3p	2.54E-02	0.27	0.835
hsa-miR-139-5p	9.30E-03	0.25	0.86
hsa-miR-142-3p	7.76E-07	14.27	0.97
hsa-miR-142-5p	1.16E-07	11.28	0.97
hsa-miR-143-3p	1.04E-03	0.43	0.83
hsa-miR-144-3p	1.32E-02	4.30	0.78

hsa-miR-144-5p	2.48E-02	3.09	0.75
hsa-miR-145-5p	1.97E-02	0.54	0.86
hsa-miR-146a-5p	1.22E-05	6.86	0.91
hsa-miR-148a-3p	1.04E-03	6.21	0.89
hsa-miR-149-5p	5.48E-03	0.29	0.87
hsa-miR-153-3p	2.64E-02	0.27	0.825
hsa-miR-154-3p	1.65E-03	0.25	0.85
hsa-miR-154-5p	1.31E-04	0.25	0.96
hsa-miR-155-5p	1.92E-10	46.56	0.94
hsa-miR-181c-3p	1.77E-03	0.31	0.88
hsa-miR-181c-5p	1.46E-05	0.21	0.87
hsa-miR-181d-5p	1.25E-02	0.35	0.76
hsa-miR-182-5p	1.92E-10	12.85	1
hsa-miR-183-5p	6.91E-07	10.08	0.96
hsa-miR-186-5p	1.32E-03	2.07	0.87
hsa-miR-187-3p	1.59E-02	0.34	0.815
hsa-miR-191-5p	4.83E-03	1.96	0.88
hsa-miR-192-5p	1.07E-03	2.70	0.895
hsa-miR-194-5p	4.28E-02	1.67	0.71
hsa-miR-195-5p	1.93E-03	0.36	0.89
hsa-miR-200c-3p	1.06E-05	15.38	0.79
hsa-miR-203a-3p	1.77E-03	0.19	0.88
hsa-miR-204-5p	1.70E-02	0.35	0.8
hsa-miR-210-3p	2.27E-04	4.17	0.84
hsa-miR-218-5p	1.95E-03	0.22	0.88
hsa-miR-296-5p	3.22E-02	2.53	0.71
hsa-miR-299-5p	8.72E-04	0.26	0.88
hsa-miR-320c	3.45E-02	2.60	0.58
hsa-miR-320d	3.99E-02	2.70	0.61
hsa-miR-323a-3p	9.36E-03	0.29	0.84
hsa-miR-323b-3p	4.13E-02	0.31	0.75
hsa-miR-328-3p	2.53E-02	0.53	0.86
hsa-miR-329-3p	5.53E-03	0.29	0.87
hsa-miR-335-3p	1.56E-02	1.98	0.76
hsa-miR-339-5p	3.15E-02	1.91	0.8
hsa-miR-33b-5p	2.57E-02	0.44	0.81
hsa-miR-361-3p	1.85E-02	1.88	0.84
hsa-miR-369-3p	3.15E-02	0.37	0.695
hsa-miR-369-5p	1.15E-03	0.29	0.9
hsa-miR-370-3p	2.69E-02	0.40	0.81
hsa-miR-376a-3p	3.09E-04	0.28	0.92
hsa-miR-376a-5p	8.43E-03	0.25	0.845
hsa-miR-376b-3p	3.16E-02	0.44	0.75
hsa-miR-376c-3p	3.09E-04	0.25	0.86
hsa-miR-378a-3p	2.91E-02	2.29	0.8
hsa-miR-378c	6.31E-03	3.91	0.78
hsa-miR-378i	1.57E-02	3.39	0.82
hsa-miR-379-5p	1.32E-03	0.24	0.88
hsa-miR-380-3p	3.53E-03	0.26	0.83
hsa-miR-381-3p	1.50E-04	0.27	0.93
hsa-miR-382-3p	6.61E-03	0.30	0.79
hsa-miR-382-5p	1.50E-02	0.40	0.86
hsa-miR-383-5p	1.82E-02	0.29	0.85
hsa-miR-409-3p	1.36E-02	0.41	0.87
hsa-miR-409-5p	5.89E-03	0.35	0.87

hsa-miR-410-3p	3.66E-04	0.17	0.87
hsa-miR-411-3p	1.81E-02	0.33	0.815
hsa-miR-411-5p	7.36E-03	0.29	0.82
hsa-miR-423-3p	3.75E-02	1.50	0.85
hsa-miR-423-5p	1.25E-02	1.80	0.85
hsa-miR-425-3p	3.99E-02	1.71	0.82
hsa-miR-425-5p	5.50E-04	3.17	0.78
hsa-miR-432-5p	1.12E-02	0.37	0.88
hsa-miR-433-3p	7.60E-03	0.26	0.83
hsa-miR-451a	3.61E-03	3.85	0.78
hsa-miR-484	1.50E-02	1.60	0.86
hsa-miR-485-3p	1.95E-03	0.23	0.87
hsa-miR-485-5p	1.32E-03	0.23	0.92
hsa-miR-486-3p	4.27E-03	3.76	0.75
hsa-miR-486-5p	7.53E-04	4.32	0.63
hsa-miR-487a-3p	7.86E-03	0.29	0.82
hsa-miR-487a-5p	1.21E-02	0.28	0.835
hsa-miR-487b-3p	2.54E-03	0.23	0.85
hsa-miR-491-5p	1.04E-03	0.25	0.93
hsa-miR-494-3p	1.82E-02	0.43	0.79
hsa-miR-495-3p	7.36E-03	0.33	0.79
hsa-miR-497-5p	1.00E-05	0.34	0.96
hsa-miR-504-5p	3.00E-02	0.26	0.83
hsa-miR-505-3p	1.04E-02	2.15	0.83
hsa-miR-532-3p	4.96E-02	2.04	0.83
hsa-miR-539-5p	1.03E-03	0.29	0.87
hsa-miR-543	1.95E-03	0.31	0.87
hsa-miR-551b-3p	6.61E-03	0.38	0.91
hsa-miR-582-5p	1.61E-06	0.17	0.93
hsa-miR-590-3p	4.18E-02	2.22	0.74
hsa-miR-590-5p	1.66E-02	2.16	0.86
hsa-miR-6087	6.40E-04	9.57	0.775
hsa-miR-625-3p	4.70E-03	3.37	0.76
hsa-miR-625-5p	1.99E-02	1.97	0.82
hsa-miR-628-3p	2.35E-04	0.35	0.96
hsa-miR-628-5p	6.76E-03	0.37	0.82
hsa-miR-629-5p	3.18E-02	2.04	0.68
hsa-miR-654-3p	2.71E-03	0.38	0.855
hsa-miR-655-3p	1.19E-03	0.28	0.875
hsa-miR-656-3p	2.60E-03	0.25	0.87
hsa-miR-744-5p	1.16E-07	0.34	0.97
hsa-miR-758-3p	5.35E-03	0.29	0.87
hsa-miR-766-3p	2.92E-04	3.17	0.89
hsa-miR-769-3p	1.44E-02	0.44	0.85
hsa-miR-769-5p	1.04E-03	0.35	0.85
hsa-miR-770-5p	4.06E-02	0.30	0.81
hsa-miR-873-5p	4.46E-07	0.10	0.89
hsa-miR-885-5p	1.81E-02	0.29	0.84
hsa-miR-935	6.31E-03	0.22	0.89
hsa-miR-1185-5p	2.92E-04	0.18	0.9
hsa-miR-1246	3.32E-08	419.95	0.81
hsa-miR-1248	2.13E-02	2.75	0.71
hsa-miR-1249-3p	3.11E-02	0.42	0.74
hsa-miR-1260a	4.12E-06	6.97	0.89
hsa-miR-1260b	4.12E-06	7.03	0.89

hsa-miR-1285-3p	4.90E-04	3.32	0.84
hsa-miR-1290	1.33E-07	679.51	0.82
hsa-miR-1296-5p	3.73E-02	0.45	0.87
hsa-miR-3065-5p	1.36E-02	0.34	0.84
hsa-miR-3613-5p	1.04E-02	2.66	0.81
hsa-miR-3615	9.33E-04	4.17	0.8
hsa-miR-4286	1.25E-02	2.77	0.71
hsa-miR-4449	3.68E-04	12.44	0.675
hsa-miR-4488	1.92E-10	114.60	0.885
hsa-miR-4521	1.33E-07	21.63	0.87
hsa-miR-4524a-3p	2.35E-04	7.50	0.705
hsa-miR-4792	4.85E-03	9.11	0.54
hsa-miR-7641	6.31E-03	4.92	0.695

CNS DLBCL, primary diffuse large B-cell lymphoma of the central nervous system; n-ML, non-malignant brain lesions; q-value, p-value after the Benjamini-Hochberg adjustment; FC, fold change (CNS DLBCL/n-ML); AUC, Area under the curve obtained by Receiver Operator Characteristic
*p-value after the Benjamini-Hochberg adjustment
