# Supplementary material for: A Set of 17 microRNAs Common for Brain and Cerebrospinal Fluid Differentiates Primary Central Nervous System Lymphoma from Non-Malignant Brain Tumors
Source: Biomolecules. 2021 Sep 21;11(9):1395. doi: 10.3390/biom11091395 (PMC8466839; doi:10.3390/biom11091395)
Supplement: Supplementary file 1 [file biomolecules-11-01395-s001.zip › Table S4.pdf]

Supplementary Table S4

Newly identified miRNAs (miRBase v.22)

	CSF		tumor			
**miRNA locus**	**CNS DLBCL n/9**	**n-ML n/12**	**CNS DLBCL n/10**	**n-ML n/10**	**Sequence**	**Precursor**
chr5:174790685..174790764:-	1	1	10	9	auccugccgacuaugcca	auccugccgacuaugccaauugucaggucccaaccugggcugggguccgaggggaguugguagaugggguggcagguagu
chr6:56187641..56187683:+	1	1	9	10	aaccauccucugcuauca	auaacccagaggucgauggaucaaaaccauccucugcuauca
chr7:2516409..2516482:-	0	2	0	0	gguucgagucccuggaggg	gguucgagucccuggaggggcccgugaucccggaggaagagccgggccagggucccugccuagaccuugccgu
chr12:95841363..95841423:+	0	0	7	8	uccucacacggggcacca	ggcuccaacugugggggucuguuuccgcagaccgcugacuuuccucacacggggcacca
chr17:65822071..65822137:-	2	0	10	10	ggggccggcggcggcggcgg	gcccccgccuccucgcccccccggcccggggcgcuggugcugggggggccggcggcggcggcgg

CNS DLBCL, central nervous system primary diffuse large B-cell lymphoma; n-ML, non-malignant brain lesions; CSF, cerebrospinal fluid; n, number of samples where the presence of the miRNA molecules was identified
