# Supplementary material for: A Set of 17 microRNAs Common for Brain and Cerebrospinal Fluid Differentiates Primary Central Nervous System Lymphoma from Non-Malignant Brain Tumors
Source: Biomolecules. 2021 Sep 21;11(9):1395. doi: 10.3390/biom11091395 (PMC8466839; doi:10.3390/biom11091395)
Supplement: Supplementary file 1 [file biomolecules-11-01395-s001.zip › Table S5.pdf]

Supplementary Table S5

CNS DLBCL-specific CSF microRNAs and biological processes; bold indicates processes linked to metabolism.

GO Biological process (miRPathDB)				
**No**	**subcategory**	**q-value***	**number of miRs observed**	**hsa-miRs**
**1**	**positive regulation of cellular metabolic process**	**1.05817E-09**	**18**	**19a-3p; 16-5p; 148a-3p; 15a-5p; 19b-3p; 221-3p; 423-5p; 21-5p; 9-3p; 15b-5p; 17-3p; 210-3p; 92a-3p; 106b-3p; 25-3p; 9-5p; 20a-5p; 26a-5p**
2	organelle organization	1.95906E-09	15	19a-3p; 16-5p; 148a-3p; 15a-5p; 19b-3p; 221-3p; 423-5p; 21-5p; 15b-5p; 92a-3p; 106b-3p; 25-3p; 9-5p; 20a-5p; 26a-5p
3	positive regulation of cellular biosynthetic process	1.95906E-09	17	19a-3p; 16-5p; 148a-3p; 15a-5p; 19b-3p; 221-3p; 423-5p; 21-5p; 9-3p; 15b-5p; 17-3p; 210-3p; 92a-3p; 25-3p; 9-5p; 20a-5p; 26a-5p
4	regulation of organelle organization	5.45239E-09	14	19a-3p; 16-5p; 148a-3p; 15a-5p; 221-3p; 21-5p; 9-3p; 15b-5p; 210-3p; 92a-3p; 25-3p; 9-5p; 20a-5p; 26a-5p
**5**	**protein metabolic process**	**6.92324E-09**	**15**	**19a-3p; 16-5p; 148a-3p; 15a-5p; 19b-3p; 221-3p; 423-5p; 21-5p; 15b-5p; 17-3p; 92a-3p; 106b-3p; 25-3p; 20a-5p; 26a-5p**
**6**	**nitrogen compound metabolic process**	**1.0157E-08**	**17**	**19a-3p; 16-5p; 148a-3p; 15a-5p; 19b-3p; 221-3p; 423-5p; 21-5p; 15b-5p; 17-3p; 210-3p; 92a-3p; 106b-3p; 25-3p; 9-5p; 20a-5p; 26a-5p**
7	cellular protein modification process	1.36762E-08	15	19a-3p; 16-5p; 148a-3p; 15a-5p; 19b-3p; 221-3p; 21-5p; 9-3p; 15b-5p; 17-3p; 210-3p; 92a-3p; 9-5p; 20a-5p; 26a-5p
8	protein modification process	1.36762E-08	15	19a-3p; 16-5p; 148a-3p; 15a-5p; 19b-3p; 221-3p; 21-5p; 9-3p; 15b-5p; 17-3p; 210-3p; 92a-3p; 9-5p; 20a-5p; 26a-5p
**9**	**catabolic process**	**2.50187E-08**	**12**	**19a-3p; 16-5p; 148a-3p; 15a-5p; 221-3p; 423-5p; 21-5p; 15b-5p; 17-3p; 92a-3p; 20a-5p; 26a-5p**
10	cytoskeleton-dependent intracellular transport	2.50187E-08	7	16-5p; 15a-5p; 19b-3p; 15b-5p; 92a-3p; 9-5p; 26a-5p
**11**	**macromolecule metabolic process**	**2.50187E-08**	**17**	**19a-3p; 16-5p; 148a-3p; 15a-5p; 19b-3p; 221-3p; 423-5p; 21-5p; 15b-5p; 17-3p; 210-3p; 92a-3p; 106b-3p; 25-3p; 9-5p; 20a-5p; 26a-5p**
12	positive regulation of macromolecule biosynthetic process	2.50187E-08	16	19a-3p; 16-5p; 148a-3p; 15a-5p; 19b-3p; 221-3p; 423-5p; 21-5p; 9-3p; 15b-5p; 17-3p; 92a-3p; 25-3p; 9-5p; 20a-5p; 26a-5p
**13**	**positive regulation of metabolic process**	**2.50187E-08**	**17**	**19a-3p; 16-5p; 148a-3p; 15a-5p; 19b-3p; 221-3p; 423-5p; 21-5p; 9-3p; 15b-5p; 17-3p; 210-3p; 92a-3p; 106b-3p; 9-5p; 20a-5p; 26a-5p**
14	regulation of cell death	2.50187E-08	14	19a-3p; 16-5p; 148a-3p; 15a-5p; 19b-3p; 221-3p; 423-5p; 21-5p; 9-3p; 15b-5p; 210-3p; 92a-3p; 9-5p; 26a-5p
**15**	**nuclear-transcribed mRNA catabolic process**	**4.50392E-08**	**10**	**16-5p; 148a-3p; 221-3p; 423-5p; 15b-5p; 17-3p; 92a-3p; 106b-3p; 25-3p; 26a-5p**
16	regulation of programmed cell death	6.16964E-08	14	19a-3p; 16-5p; 148a-3p; 15a-5p; 221-3p; 423-5p; 21-5p; 9-3p; 15b-5p; 210-3p; 92a-3p; 9-5p; 20a-5p; 26a-5p

17	cell death	7.2243E-08	14	19a-3p; 16-5p; 148a-3p; 15a-5p; 19b-3p; 221-3p; 21-5p; 9-3p; 15b-5p; 210-3p; 92a-3p; 9-5p; 20a-5p; 26a-5p
18	miRNA loading onto RISC involved in gene silencing by miRNA	8.69552E-08	9	19a-3p; 16-5p; 148a-3p; 15a-5p; 19b-3p; 221-3p; 21-5p; 15b-5p; 20a-5p
19	immune system process	8.7533E-08	12	19a-3p; 16-5p; 148a-3p; 15a-5p; 221-3p; 21-5p; 9-3p; 15b-5p; 92a-3p; 25-3p; 9-5p; 20a-5p
20	apoptotic process	9.33689E-08	14	19a-3p; 16-5p; 148a-3p; 15a-5p; 19b-3p; 221-3p; 21-5p; 9-3p; 15b-5p; 210-3p; 92a-3p; 9-5p; 20a-5p; 26a-5p
**21**	**regulation of protein metabolic process**	**9.33689E-08**	**15**	**19a-3p; 16-5p; 148a-3p; 15a-5p; 19b-3p; 221-3p; 423-5p; 21-5p; 9-3p; 15b-5p; 17-3p; 92a-3p; 25-3p; 9-5p; 26a-5p**
**22**	**cellular metabolic process**	**9.3441E-08**	**16**	**19a-3p; 148a-3p; 15a-5p; 19b-3p; 221-3p; 21-5p; 9-3p; 15b-5p; 17-3p; 210-3p; 92a-3p; 106b-3p; 25-3p; 9-5p; 20a-5p; 26a-5p**
**23**	**cellular protein metabolic process**	**9.3441E-08**	**14**	**19a-3p; 16-5p; 148a-3p; 15a-5p; 19b-3p; 221-3p; 423-5p; 21-5p; 15b-5p; 17-3p; 92a-3p; 25-3p; 20a-5p; 26a-5p**
24	membrane organization	9.3441E-08	10	19a-3p; 16-5p; 148a-3p; 15a-5p; 221-3p; 423-5p; 15b-5p; 92a-3p; 9-5p; 20a-5p
25	negative regulation of transcription DNA-templated	9.3441E-08	16	19a-3p; 16-5p; 148a-3p; 15a-5p; 19b-3p; 221-3p; 423-5p; 21-5p; 9-3p; 15b-5p; 210-3p; 92a-3p; 25-3p; 9-5p; 20a-5p; 26a-5p
26	positive regulation of gene expression	9.3441E-08	16	19a-3p; 16-5p; 148a-3p; 15a-5p; 19b-3p; 221-3p; 423-5p; 21-5p; 9-3p; 15b-5p; 17-3p; 92a-3p; 25-3p; 9-5p; 20a-5p; 26a-5p
27	programmed cell death	9.3441E-08	14	19a-3p; 16-5p; 148a-3p; 15a-5p; 19b-3p; 221-3p; 21-5p; 9-3p; 15b-5p; 210-3p; 92a-3p; 9-5p; 20a-5p; 26a-5p
**28**	**regulation of metabolic process**	**9.3441E-08**	**18**	**19a-3p; 16-5p; 148a-3p; 15a-5p; 19b-3p; 221-3p; 423-5p; 21-5p; 9-3p; 15b-5p; 17-3p; 210-3p; 92a-3p; 106b-3p; 25-3p; 9-5p; 20a-5p; 26a-5p**
29	posttranscriptional regulation of gene expression	9.59062E-08	13	19a-3p; 16-5p; 148a-3p; 15a-5p; 19b-3p; 221-3p; 21-5p; 15b-5p; 17-3p; 92a-3p; 25-3p; 20a-5p; 26a-5p
30	regulation of gene expression	9.59062E-08	18	19a-3p; 16-5p; 148a-3p; 15a-5p; 19b-3p; 221-3p; 423-5p; 21-5p; 9-3p; 15b-5p; 17-3p; 210-3p; 92a-3p; 106b-3p; 25-3p; 9-5p; 20a-5p; 26a-5p
31	regulation of gene expression epigenetic	9.59062E-08	12	19a-3p; 16-5p; 148a-3p; 15a-5p; 19b-3p; 221-3p; 21-5p; 15b-5p; 92a-3p; 9-5p; 20a-5p; 26a-5p

*p-value after the Benjamini-Hochberg adjustment
