# Supplementary material for: A Set of 17 microRNAs Common for Brain and Cerebrospinal Fluid Differentiates Primary Central Nervous System Lymphoma from Non-Malignant Brain Tumors
Source: Biomolecules. 2021 Sep 21;11(9):1395. doi: 10.3390/biom11091395 (PMC8466839; doi:10.3390/biom11091395)
Supplement: Supplementary file 1 [file biomolecules-11-01395-s001.zip › Table S6.pdf]

Supplementary Table S6
CNS DLBCL-specific CSF microRNAs and diseases.

miRWalk_Diseases

No	subcategory	q-value*	number of miRs observed	hsa-miRs
1	Hypertension Pulmonary	0.0035763	4	19a-3p; 19b-3p; 92a-3p; 20a-5p
2	Hypertension	0.0225845	4	19a-3p; 19b-3p; 92a-3p; 20a-5p
3	Adenocarcinoma	0.0286493	7	16-5p; 148a-3p; 15a-5p; 21-5p; 210-3p; 9-5p; 20a-5p
4	Aneuploidy	0.0286493	2	16-5p; 15a-5p
5	Arthritis	0.0286493	3	19a-3p; 19b-3p; 21-5p
6	Arthritis Rheumatoid	0.0286493	2	19a-3p; 19b-3p
7	Carcinoma Non-Small-Cell Lung	0.0286493	4	16-5p; 15a-5p; 221-3p; 21-5p
8	Glioblastoma	0.0286493	6	19a-3p; 221-3p; 21-5p; 92a-3p; 20a-5p; 26a-5p
9	Lymphoma	0.0286493	6	16-5p; 15a-5p; 21-5p; 9-5p; 20a-5p; 26a-5p
10	Monoclonal Gammopathy of Undetermined Significance	0.0286493	4	19a-3p; 19b-3p; 92a-3p; 25-3p
11	Multiple Myeloma	0.0286493	4	19a-3p; 19b-3p; 92a-3p; 25-3p
12	Paraproteinemias	0.0286493	4	19a-3p; 19b-3p; 92a-3p; 25-3p
13	Pheochromocytoma	0.0286493	2	16-5p; 15a-5p
14	Stomach Neoplasms	0.0286493	8	16-5p; 148a-3p; 221-3p; 21-5p; 15b-5p; 106b-3p; 25-3p; 9-5p
15	Infection	0.0365215	5	16-5p; 15a-5p; 221-3p; 15b-5p; 26a-5p
16	Leukemia	0.0365215	8	16-5p; 15a-5p; 19b-3p; 221-3p; 21-5p; 92a-3p; 9-5p; 26a-5p
17	Anoxia	0.0411627	5	16-5p; 21-5p; 15b-5p; 210-3p; 20a-5p

Diseases (MNDR)

No	subcategory	q-value	number of miRs observed	hsa-miRs
1	Behcets disease	2.28413E-19	14	19a-3p; 16-5p; 148a-3p; 15a-5p; 19b-3p; 221-3p; 21-5p; 15b-5p; 210-3p; 92a-3p; 106b-3p; 9-5p; 20a-5p; 26a-5p
2	HIV-1	2.28413E-19	14	19a-3p; 16-5p; 148a-3p; 15a-5p; 19b-3p; 221-3p; 21-5p; 15b-5p; 210-3p; 92a-3p; 106b-3p; 9-5p; 20a-5p; 26a-5p
3	Leukemia. Biphenotypic. Acute	2.28413E-19	14	19a-3p; 16-5p; 148a-3p; 15a-5p; 19b-3p; 221-3p; 21-5p; 15b-5p; 210-3p; 92a-3p; 106b-3p; 9-5p; 20a-5p; 26a-5p
4	Lymphoma. Extranodal NK-T-Cell	2.28413E-19	14	19a-3p; 16-5p; 148a-3p; 15a-5p; 19b-3p; 221-3p; 21-5p; 15b-5p; 210-3p; 92a-3p; 106b-3p; 9-5p; 20a-5p; 26a-5p
5	Sjogrens syndrome	2.28413E-19	14	19a-3p; 16-5p; 148a-3p; 15a-5p; 19b-3p; 221-3p; 21-5p; 15b-5p; 210-3p; 92a-3p; 106b-3p; 9-5p; 20a-5p; 26a-5p
6	Endomyocardial fibrosis	2.28413E-19	14	19a-3p; 16-5p; 148a-3p; 15a-5p; 19b-3p; 221-3p; 21-5p; 210-3p; 92a-3p; 106b-3p; 25-3p; 9-5p; 20a-5p; 26a-5p
7	Nervous system disease	2.28413E-19	14	19a-3p; 16-5p; 148a-3p; 15a-5p; 19b-3p; 221-3p; 21-5p; 15b-5p; 210-3p; 92a-3p; 106b-3p; 9-5p; 20a-5p; 26a-5p
8	Prion disease	2.28413E-19	14	19a-3p; 16-5p; 148a-3p; 15a-5p; 19b-3p; 221-3p; 21-5p; 15b-5p; 210-3p; 92a-3p; 106b-3p; 9-5p; 20a-5p; 26a-5p
9	Psoriatic arthritis	2.28413E-19	14	19a-3p; 16-5p; 148a-3p; 15a-5p; 19b-3p; 221-3p; 21-5p; 15b-5p; 210-3p; 92a-3p; 106b-3p; 9-5p; 20a-5p; 26a-5p
10	Stomach disease	2.28413E-19	14	19a-3p; 16-5p; 148a-3p; 15a-5p; 19b-3p; 221-3p; 21-5p; 15b-5p; 210-3p; 92a-3p; 106b-3p; 9-5p; 20a-5p; 26a-5p

11	Esophagus	2.38383E-19	14	19a-3p; 16-5p; 148a-3p; 15a-5p; 19b-3p; 221-3p; 21-5p; 15b-5p; 210-3p; 92a-3p; 106b-3p; 9-5p; 20a-5p; 26a-5p
12	Myocardial Reperfusion Injury	2.38383E-19	14	19a-3p; 16-5p; 148a-3p; 15a-5p; 19b-3p; 221-3p; 21-5p; 15b-5p; 210-3p; 92a-3p; 106b-3p; 9-5p; 20a-5p; 26a-5p
13	SARS Virus	2.38383E-19	14	19a-3p; 16-5p; 148a-3p; 15a-5p; 19b-3p; 221-3p; 21-5p; 15b-5p; 210-3p; 92a-3p; 106b-3p; 9-5p; 20a-5p; 26a-5p
14	Cowden disease	2.61046E-19	14	19a-3p; 16-5p; 148a-3p; 15a-5p; 19b-3p; 221-3p; 21-5p; 15b-5p; 210-3p; 92a-3p; 106b-3p; 9-5p; 20a-5p; 26a-5p
15	Intermediate coronary syndrome	2.61046E-19	14	19a-3p; 16-5p; 148a-3p; 15a-5p; 19b-3p; 221-3p; 21-5p; 15b-5p; 210-3p; 92a-3p; 106b-3p; 9-5p; 20a-5p; 26a-5p
16	Neuroendocrine carcinoma	2.61046E-19	14	19a-3p; 16-5p; 148a-3p; 15a-5p; 19b-3p; 221-3p; 21-5p; 15b-5p; 210-3p; 92a-3p; 106b-3p; 9-5p; 20a-5p; 26a-5p
17	Endometrial carcinoma	3.91897E-19	14	19a-3p; 16-5p; 148a-3p; 15a-5p; 19b-3p; 221-3p; 21-5p; 15b-5p; 210-3p; 92a-3p; 106b-3p; 9-5p; 20a-5p; 26a-5p
18	Neutropenia	3.91897E-19	14	19a-3p; 16-5p; 148a-3p; 15a-5p; 19b-3p; 221-3p; 21-5p; 15b-5p; 210-3p; 92a-3p; 106b-3p; 9-5p; 20a-5p; 26a-5p
19	Pulmonary fibrosis	3.91897E-19	14	19a-3p; 16-5p; 148a-3p; 15a-5p; 19b-3p; 221-3p; 21-5p; 15b-5p; 210-3p; 92a-3p; 106b-3p; 9-5p; 20a-5p; 26a-5p
20	Lymphoma. Non-Hodgkin	7.31138E-19	16	19a-3p; 16-5p; 148a-3p; 15a-5p; 19b-3p; 221-3p; 21-5p; 9-3p; 15b-5p; 17-3p; 210-3p; 92a-3p; 25-3p; 9-5p; 20a-5p; 26a-5p
21	Atopic dermatitis	8.08309E-19	14	19a-3p; 16-5p; 148a-3p; 15a-5p; 19b-3p; 221-3p; 21-5p; 15b-5p; 210-3p; 92a-3p; 106b-3p; 9-5p; 20a-5p; 26a-5p
22	AIDS Dementia Complex	9.93779E-19	15	19a-3p; 16-5p; 148a-3p; 15a-5p; 19b-3p; 221-3p; 21-5p; 9-3p; 15b-5p; 210-3p; 92a-3p; 106b-3p; 9-5p; 20a-5p; 26a-5p
23	Brain Injuries	1.24526E-18	14	19a-3p; 16-5p; 148a-3p; 15a-5p; 19b-3p; 221-3p; 21-5p; 15b-5p; 210-3p; 92a-3p; 106b-3p; 9-5p; 20a-5p; 26a-5p
24	Cardiovascular Diseases	1.5389E-18	14	19a-3p; 16-5p; 148a-3p; 15a-5p; 19b-3p; 221-3p; 21-5p; 15b-5p; 210-3p; 92a-3p; 106b-3p; 9-5p; 20a-5p; 26a-5p

CNS DLBCL-specific CSF microRNAs in other selected CNS disorders

	subcategory	q-value	number of miRs observed	hsa-miRs
	Lymphoproliferative Disorders	6.641E-18	13	19a-3p; 148a-3p; 15a-5p; 19b-3p; 221-3p; 21-5p; 15b-5p; 210-3p; 92a-3p; 106b-3p; 9-5p; 20a-5p; 26a-5p
	Disease of metabolism	1.26073E-17	13	19a-3p; 16-5p; 148a-3p; 15a-5p; 19b-3p; 221-3p; 21-5p; 15b-5p; 210-3p; 106b-3p; 9-5p; 20a-5p; 26a-5p
	Precursor Cell Lymphoblastic Leukemia-Lymphoma	5.72176E-16	16	19a-3p; 16-5p; 148a-3p; 15a-5p; 19b-3p; 221-3p; 21-5p; 9-3p; 15b-5p; 17-3p; 210-3p; 92a-3p; 106b-3p; 9-5p; 20a-5p; 26a-5p
	B-cell lymphoma	2.71217E-15	17	19a-3p; 16-5p; 148a-3p; 15a-5p; 19b-3p; 221-3p; 21-5p; 9-3p; 15b-5p; 17-3p; 210-3p; 92a-3p; 106b-3p; 25-3p; 9-5p; 20a-5p; 26a-5p
	Burkitt lymphoma	3.73951E-15	16	19a-3p; 16-5p; 148a-3p; 15a-5p; 19b-3p; 221-3p; 21-5p; 15b-5p; 17-3p; 210-3p; 92a-3p; 106b-3p; 25-3p; 9-5p; 20a-5p; 26a-5p
	Infection	8.38443E-15	14	19a-3p; 16-5p; 148a-3p; 15a-5p; 19b-3p; 221-3p; 21-5p; 15b-5p; 210-3p; 92a-3p; 106b-3p; 9-5p; 20a-5p; 26a-5p
	Cerebral Hemorrhage	1.8654E-14	11	19a-3p; 16-5p; 148a-3p; 15a-5p; 19b-3p; 221-3p; 21-5p; 210-3p; 92a-3p; 9-5p; 20a-5p

	Brain ischemia	1.8654E-14	11	16-5p; 148a-3p; 15a-5p; 19b-3p; 221-3p; 21-5p; 15b-5p; 106b-3p; 9-5p; 20a-5p; 26a-5p
	Central nervous system disease	1.8654E-14	11	16-5p; 148a-3p; 15a-5p; 221-3p; 21-5p; 15b-5p; 210-3p; 106b-3p; 9-5p; 20a-5p; 26a-5p
	Hypoxia-Ischemia. Brain	2.73846E-14	11	16-5p; 148a-3p; 15a-5p; 221-3p; 21-5p; 15b-5p; 210-3p; 106b-3p; 9-5p; 20a-5p; 26a-5p
	Lymphoma. Primary Effusion	2.73846E-14	11	19a-3p; 16-5p; 148a-3p; 15a-5p; 19b-3p; 221-3p; 21-5p; 210-3p; 92a-3p; 9-5p; 20a-5p
	Cerebral infarction	2.73846E-14	11	19a-3p; 16-5p; 148a-3p; 15a-5p; 19b-3p; 221-3p; 21-5p; 210-3p; 92a-3p; 9-5p; 20a-5p
	Lipid metabolism disorder	4.90958E-14	11	16-5p; 148a-3p; 15a-5p; 221-3p; 21-5p; 15b-5p; 210-3p; 106b-3p; 9-5p; 20a-5p; 26a-5p
	Diffuse large B-cell lymphoma	1.53287E-13	12	19a-3p; 16-5p; 148a-3p; 15a-5p; 19b-3p; 221-3p; 21-5p; 210-3p; 92a-3p; 9-5p; 20a-5p; 26a-5p
	Inflammation	2.13822E-13	11	19a-3p; 16-5p; 148a-3p; 15a-5p; 19b-3p; 221-3p; 21-5p; 92a-3p; 106b-3p; 9-5p; 20a-5p
	Brain cancer	8.02872E-13	11	19a-3p; 16-5p; 148a-3p; 15a-5p; 19b-3p; 221-3p; 21-5p; 210-3p; 92a-3p; 106b-3p; 20a-5p
	Precursor B-Cell Lymphoblastic Leukemia-Lymphoma	1.49627E-12	10	19a-3p; 16-5p; 148a-3p; 19b-3p; 221-3p; 21-5p; 210-3p; 92a-3p; 9-5p; 20a-5p
	Leukemia. B-Cell	2.84269E-09	8	19a-3p; 148a-3p; 221-3p; 21-5p; 210-3p; 106b-3p; 9-5p; 20a-5p
	lymphoma	3.56791E-09	16	19a-3p; 16-5p; 148a-3p; 15a-5p; 19b-3p; 221-3p; 21-5p; 9-3p; 15b-5p; 17-3p; 92a-3p; 106b-3p; 25-3p; 9-5p; 20a-5p; 26a-5p
	Lymphoblastoid	7.0076E-09	9	16-5p; 15a-5p; 21-5p; 9-3p; 92a-3p; 25-3p; 9-5p; 20a-5p; 26a-5p
	Brain disease	2.20671E-08	17	19a-3p; 16-5p; 148a-3p; 15a-5p; 19b-3p; 221-3p; 423-5p; 21-5p; 15b-5p; 17-3p; 210-3p; 92a-3p; 106b-3p; 25-3p; 9-5p; 20a-5p; 26a-5p
	Central nervous system lymphoma	2.38101E-08	6	148a-3p; 19b-3p; 21-5p; 92a-3p; 9-5p; 20a-5p

*p-value after the Benjamini-Hochberg adjustment
