# Supplementary material for: A Set of 17 microRNAs Common for Brain and Cerebrospinal Fluid Differentiates Primary Central Nervous System Lymphoma from Non-Malignant Brain Tumors
Source: Biomolecules. 2021 Sep 21;11(9):1395. doi: 10.3390/biom11091395 (PMC8466839; doi:10.3390/biom11091395)
Supplement: Supplementary file 1 [file biomolecules-11-01395-s001.zip › Table S7.pdf]

Supplementary Table S7

Drugs potentially affecting CNS DLBCL-specific CSF/tumor miRNAs.

Drugs (SM2miR)

Drugs potentially affecting CNS DLBCL-specific CSF miRNAs

No	subcategory	type of substance	q-value*	number of miRs observed	hsa-miRs
1	Curcumin	extract from rhizomes of the plant *Curcuma longa*	2.31637E-08	12	19a-3p; 16-5p; 148a-3p; 15a-5p; 21-5p; 15b-5p; 210-3p; 92a-3p; 106b-3p; 25-3p; 20a-5p; 26a-5p
2	Formaldehyde	organic compound potential. occupational carcinogen	5.94307E-05	11	19a-3p; 16-5p; 15a-5p; 19b-3p; 21-5p; 15b-5p; 17-3p; 25-3p; 9-5p; 20a-5p; 26a-5p
3	Hydroxamic acid HDACi LAQ824	HDACi	5.94307E-05	7	19a-3p; 148a-3p; 19b-3p; 21-5p; 92a-3p; 20a-5p; 26a-5p
4	Benzoapyrene	carcinogen	0.000579369	4	16-5p; 15a-5p; 92a-3p; 25-3p
5	Morphine	opiate pain medication	0.0012018	6	16-5p; 221-3p; 423-5p; 21-5p; 15b-5p; 26a-5p
6	5-aza-2-deoxycytidine 5-Aza-CdR (Decitabine)	epigenetic drug. demethylating agent. analog of cytidine	0.0015511	7	19a-3p; 16-5p; 15a-5p; 21-5p; 210-3p; 25-3p; 20a-5p
7	Bisphenol A	organic synthetic compound. xenoestrogen	0.0016742	5	16-5p; 19b-3p; 21-5p; 15b-5p; 20a-5p
8	Diethylstilbestrol	nonsteroidal estrogen	0.0036876	8	16-5p; 15a-5p; 9-3p; 15b-5p; 25-3p; 9-5p; 20a-5p; 26a-5p
9	Marine fungal metabolite 1386A	bioactive compound from the mangrove fungus 1386A	0.0042302	6	16-5p; 21-5p; 15b-5p; 25-3p; 20a-5p; 26a-5p
10	3.3-diindolylmethane BR-DIM	bioactive compounds from cruciferes	0.0049726	2	21-5p; 92a-3p
11	Ginsenoside Rh2	bioactive compound from Panax ginseng	0.0049726	8	16-5p; 148a-3p; 221-3p; 21-5p; 15b-5p; 210-3p; 92a-3p; 25-3p
12	Histone deacetylase inhibitor ITF2357	HDACi	0.0049726	2	19a-3p; 19b-3p
13	Melphalan	chemotherapy drug (alkylating agent)	0.0049726	2	16-5p; 15a-5p
14	5-azacytidine 5-AzaC (Azacitidine)	epigenetic drug. demethylating agent. analog of cytidine	0.005687	3	19a-3p; 19b-3p; 20a-5p
15	Temozolomide	epigenetic drug. demethylating agent. analog of cytidine	0.005687	4	21-5p; 210-3p; 9-5p; 26a-5p
16	2.3.7.8-tetrachlorodibenzo-p-dioxin TCDD	dioxin	0.0095402	2	92a-3p; 25-3p
17	All-trans-retinoic acid ATRA	chemotherapy drug-naturally-occurring acid of retinol	0.0095402	4	16-5p; 15a-5p; 21-5p; 15b-5p
18	Arsenic trioxide	chemotherapy drug (natural product)	0.0095402	7	19a-3p; 16-5p; 148a-3p; 221-3p; 21-5p; 210-3p; 9-5p
19	Bortezomib	antineoplastic agents-proteosome inhibitor	0.0095402	2	16-5p; 15a-5p
20	Nicotine	stimulant drug -agonist at nicotinic acetylcholine receptors	0.0095402	2	16-5p; 21-5p
21	Goserelin	gonadotropin-releasing hormone (GnRH) agonists	0.0127184	4	148a-3p; 9-3p; 210-3p; 9-5p
22	Activin A	dimeric glycoprotein -TGF-β family	0.0133869	3	19a-3p; 19b-3p; 221-3p
23	Sulindac sulfide	nonsteroidal anti-inflammatory drug (NSAID)	0.0156444	2	21-5p; 9-5p

24	Sunitinib	chemotherapy drug-vascular endothelial growth factor (VEGF) receptor inhibitor	0.0156444	2	221-3p; 21-5p
25	Vitamin D3	Vitamin D3	0.0192094	4	19a-3p; 221-3p; 15b-5p; 26a-5p
26	Trichostatin A TSA	hydroxamic acids HDACi	0.0245804	6	19a-3p; 16-5p; 221-3p; 21-5p; 25-3p; 20a-5p
27	5-aza-2-deoxycytidine 5-Aza-CdR + trichostatin ATSA	Decitabine +Trichostatin A	0.0291463	4	19a-3p; 16-5p; 25-3p; 20a-5p
28	5-Fluorouracil	chemotherapy drug - antimetabolites	0.0293139	11	19a-3p; 16-5p; 15a-5p; 423-5p; 21-5p; 9-3p; 15b-5p; 210-3p; 92a-3p; 25-3p; 20a-5p
29	O.p-dichlorodiphenyltrichloroethane DDT	insecticide	0.0293139	2	21-5p; 15b-5p
30	Glucocorticoid	corticosteroids	0.0414114	4	16-5p; 19b-3p; 21-5p; 15b-5p

Drugs potentially affecting CNS DLBCL-specific tumor miRNAs

No	subcategory	type of substance	q-value	number of miRs observed	hsa-miRs
1	Arsenic trioxide	chemotherapy drug (natural product)	5.10584E-07	40	17-5p; 155-5p; 96-5p; 18a-5p; 19a-3p; 183-5p; 200c-3p; 146a-5p; 181c-5p; 210-3p; 9-5p; 16-5p; 124-3p; 30a-5p; 191-5p; 491-5p; 125a-5p; 203a-3p; 128-3p; 143-3p; hsa-let-7a-5p; 218-5p; 148a-3p; 30c-5p; 144-3p; 132-3p; 98-5p; 149-5p; 1-3p; 181d-5p; 378a-3p; 7-5p; 27b-3p; 27a-3p; 32-5p; 21-5p; 20b-5p; 23b-3p; 125b-5p; 126-3p
2	Hydroxamic acid HDACi LAQ824	HDACi	3.28965E-06	20	92a-3p; 20a-5p; 19a-3p; hsa-let-7c-5p; 183-5p; hsa-let-7e-5p; 200c-3p; 19b-3p; 106b-5p; 191-5p; hsa-let-7a-5p; 148a-3p; 30c-5p; 98-5p; 26a-5p; 27b-3p; 27a-3p; 21-5p; 23b-3p; 125b-5p
3	Aidi injection	extracts from Radix Ginseng. Astragaloside. Eleutherococcus senticosus. and Cantharidin	5.71033E-05	11	155-5p; 18a-5p; 210-3p; 191-5p; 195-5p; hsa-let-7a-5p; 181d-5p; 7-5p; 27a-3p; 125b-5p; 126-3p
4	Formaldehyde	organic compound potential. occupational carcinogen	5.71033E-05	40	17-5p; 182-5p; 20a-5p; 18a-5p; 19a-3p; hsa-let-7c-5p; 17-3p; hsa-let-7e-5p; 93-5p; 19b-3p; 582-5p; 106b-5p; 15b-5p; 130b-3p; 25-3p; 9-5p; 425-5p; 16-5p; 192-5p; 186-5p; hsa-let-7a-5p; 487b-3p; 99a-5p; 130a-3p; 98-5p; 15a-5p; 194-5p; 26a-5p; 106a-5p; 181d-5p; 378a-3p; 590-5p; 27b-3p; 27a-3p; hsa-let-7b-5p; 21-5p; 20b-5p; 454-3p; 23b-3p; 126-3p
5	5-aza-2-deoxycytidine 5-Aza-CdR (Decitabine)	epigenetic drug. demethylating agent. analog of cytidine	7.89999E-05	26	17-5p; 182-5p; 20a-5p; 155-5p; 19a-3p; 93-5p; 106b-5p; 25-3p; 210-3p; 16-5p; 31-5p; 191-5p; 30b-5p; 130a-3p; 15a-5p; 181d-5p; 378a-3p; 27b-3p; 27a-3p; 494-3p; 21-5p; 20b-5p; 23b-3p; 145-5p; 125b-5p; 126-3p

6	5-azacytidine 5-AzaC (Azacitidine)	epigenetic drug. demethylating agent. analog of cytidine	0.000274924	8	17-5p; 20a-5p; 18a-5p; 19a-3p; 19b-3p; 203a-3p; 145-5p; 125b-5p
7	Bicalutamide	nonsteroidal antiandrogen	0.000274924	21	17-5p; hsa-let-7e-5p; 210-3p; 9-5p; 30d-5p; 381-3p; 376a-3p; 376c-3p; 9-3p; 218-5p; 30b-5p; 99a-5p; 144-3p; 149-5p; 551b-3p; 106a-5p; 1-3p; 204-5p; 32-5p; 23b-3p; 125b-5p
8	Ginsenoside Rh2	bioactive compound from Panax ginseng	0.000274924	37	92a-3p; 155-5p; 18a-5p; hsa-let-7c-5p; 1290; hsa-let-7e-5p; 181c-5p; 106b-5p; 15b-5p; 130b-3p; 25-3p; 210-3p; 486-5p; 16-5p; 30a-5p; 186-5p; 31-5p; 128-3p; 218-5p; 135a-5p; 148a-3p; 21-3p; 31-3p; 98-5p; 361-3p; 129-2-3p; 296-5p; 323a-3p; 18b-5p; 590-5p; 138-5p; 27b-3p; 32-5p; 21-5p; 23b-3p; 370-3p; 125b-5p
9	Vorinostat SAHA	HDACi	0.000274924	28	92a-3p; 182-5p; 155-5p; 96-5p; 17-3p; 183-5p; 425-5p; 381-3p; 376a-3p; 124-3p; 9-3p; 769-5p; 379-5p; 487b-3p; 654-3p; 132-3p; 134-5p; 129-2-3p; 106a-5p; 629-5p; 432-5p; 127-3p; 409-3p; 382-5p; 383-5p; 502-3p; 99b-5p; 126-3p
10	1.2.6-Tri-O-galloyl-beta-D-glucopyranose	gallotonina from cell cultures of Cornus officinalis.	0.000459038	42	92a-3p; 17-5p; 182-5p; 18a-5p; 19a-3p; hsa-let-7c-5p; 19b-3p; 25-3p; 9-5p; 154-5p; 30d-5p; 381-3p; 376a-3p; 30a-5p; 186-5p; 451a; 191-5p; 195-5p; 379-5p; 485-5p; 143-3p; hsa-let-7a-5p; 218-5p; 30b-5p; 144-3p; 132-3p; 484; 134-5p; 98-5p; 149-5p; 409-5p; 194-5p; 26a-5p; 296-5p; 423-3p; 18b-5p; 432-5p; 7-5p; 27b-3p; 27a-3p; 125b-5p; 99b-5p
11	Marine fungal metabolite 1386A	bioactive compound from the mangrove fungus 1386A	0.000465703	22	182-5p; 20a-5p; 1246; hsa-let-7c-5p; hsa-let-7e-5p; 93-5p; 200c-3p; 15b-5p; 25-3p; 16-5p; 125a-5p; 203a-3p; hsa-let-7a-5p; 26a-5p; 320c; 7-5p; 320d; 27b-3p; hsa-let-7b-5p; 21-5p; 125b-5p; 99b-5p
12	Curcumin	extract from rhizomes of the plant *Curcuma longa*	0.00061173	26	92a-3p; 106b-3p; 20a-5p; 19a-3p; 92a-1-5p; 93-5p; 200c-3p; 15b-5p; 25-3p; 210-3p; 16-5p; 125a-5p; 195-5p; 203a-3p; hsa-let-7a-5p; 148a-3p; 98-5p; 15a-5p; 26a-5p; 181d-5p; 7-5p; 124-5p; 204-5p; 27a-3p; 21-5p; 23b-3p
13	5-aza-2-deoxycytidine 5-Aza-CdR + trichostatin ATSA	Decitabine +Trichostatin A	0.000985262	18	182-5p; 20a-5p; 19a-3p; 93-5p; 25-3p; 16-5p; 31-5p; 191-5p; 30b-5p; 409-5p; 129-2-3p; 181d-5p; 378a-3p; 127-3p; 27a-3p; 494-3p; 20b-5p; 23b-3p

14	Trichostatin A TSA	hydroxamic acids HDACi	0.0022961	30	182-5p; 20a-5p; 155-5p; 19a-3p; hsa-let-7c-5p; 93-5p; 200c-3p; 146a-5p; 106b-5p; 25-3p; 486-5p; 16-5p; 31-5p; 191-5p; 143-3p; hsa-let-7a-5p; 129-5p; 30b-5p; 144-3p; 194-5p; 139-5p; 1-3p; 181d-5p; 27a-3p; 494-3p; 21-5p; 20b-5p; 153-3p; 23b-3p; 126-3p
15	Gemcitabine	chemotherapy drug (antimetabolite)	0.0023399	24	155-5p; hsa-let-7c-5p; 16-5p; 154-5p; 186-5p; 128-3p; hsa-let-7a-5p; 329-3p; 132-3p; 149-5p; 409-5p; 194-5p; 129-2-3p; 26a-5p; 139-5p; 323a-3p; 1-3p; 187-3p; 339-5p; 204-5p; 27a-3p; hsa-let-7b-5p; 21-5p; 211-5p
16	17beta-estradiol E2	estrogen steroid hormone	0.0032036	24	17-5p; hsa-let-7c-5p; hsa-let-7e-5p; 200c-3p; 146a-5p; 15b-5p; 9-5p; 124-3p; 203a-3p; 128-3p; 143-3p; hsa-let-7a-5p; 30c-5p; 30b-5p; 98-5p; 409-5p; 1-3p; 27b-3p; 27a-3p; hsa-let-7b-5p; 21-5p; 23b-3p; 211-5p; 126-3p
17	Morphine	opiate pain medication	0.0044981	16	155-5p; 146a-5p; 15b-5p; 16-5p; 191-5p; 128-3p; 423-5p; 30c-5p; 132-3p; 26a-5p; 320c; 382-5p; 21-5p; 23b-3p; 125b-5p; 99b-5p
18	Dexamethasone	corticosteroids	0.0073903	5	451a; 148a-3p; 323a-3p; 27b-3p; 27a-3p
19	Glucocorticoid	corticosteroids	0.0073903	18	155-5p; 142-3p; 93-5p; 19b-3p; 146a-5p; 15b-5p; 16-5p; 125a-5p; 195-5p; 203a-3p; 99a-5p; 27a-3p; 502-3p; 139-3p; 21-5p; 145-5p; 99b-5p; 126-3p
20	Paclitaxel	chemotherapy drugs. Plant alkaloids from the periwinkle plant (catharanthus rosea)(vinca alkaloids), the bark of the Pacific Yew tree (taxus) (the taxanes).	0.0073903	8	30a-5p; 451a; 148a-3p; 323a-3p; 27b-3p; 27a-3p; 99b-5p; 126-3p
21	Trastuzumab	antineoplastics, anti-HER2; monoclonal antibody	0.0074885	14	18a-5p; 1246; 210-3p; 425-5p; 30d-5p; 125a-5p; 195-5p; 30c-5p; 30b-5p; 98-5p; 26a-5p; 296-5p; 494-3p; 21-5p
22	Bisphenol A	organic synthetic compound. xenoestrogen	0.0082327	12	20a-5p; 155-5p; hsa-let-7c-5p; 19b-3p; 146a-5p; 106b-5p; 15b-5p; 16-5p; 376c-3p; 106a-5p; 27b-3p; 21-5p
23	Diethylstilbestrol	nonsteroidal estrogen	0.0082327	30	17-5p; 20a-5p; 92a-1-5p; 93-5p; 497-5p; 106b-5p; 15b-5p; 130b-3p; 25-3p; 9-5p; 16-5p; 9-3p; 195-5p; 203a-3p; 218-5p; 135a-5p; 625-5p; 409-5p; 758-3p; 15a-5p; 194-5p; 26a-5p; 106a-5p; 181d-5p; 138-5p; 20b-5p; 454-3p; 370-3p; 126-3p; 92b-3p
24	Goserelin	gonadotropin-releasing hormone (GnRH) agonists	0.0093793	13	130b-3p; 210-3p; 9-5p; 9-3p; 203a-3p; 128-3p; 135a-5p; 148a-3p; 99a-5p; 134-5p; 149-5p; 7-5p; 125b-5p

25	Enoxacin	quinolone/fluoroquinolone antibiotic	0.0123216	17	96-5p; 146a-5p; 16-5p; 124-3p; 30a-5p; 31-5p; 128-3p; 143-3p; hsa-let-7a-5p; 99a-5p; 26a-5p; 139-5p; 7-5p; hsa-let-7b-5p; 21-5p; 125b-5p; 126-3p
26	5-aza-2-deoxycytidine 5-Aza-CdR + 4-phenylbutyric acid PBA	Decitabine +Phenylbutyric acid	0.0142215	10	182-5p; 183-5p; 192-5p; 376c-3p; 132-3p; 134-5p; 495-3p; 127-3p; 494-3p; 126-3p
27	Benzoapyrene	carcinogen	0.0193092	6	92a-3p; 146a-5p; 25-3p; 16-5p; 15a-5p; 125b-5p
28	Metformin	antihyperglycemic agent of the biguanide class	0.0193092	6	hsa-let-7c-5p; 200c-3p; 192-5p; hsa-let-7a-5p; 26a-5p; hsa-let-7b-5p
29	Temozolomide	chemotherapy drug (alkylating agent)	0.0193092	10	155-5p; 210-3p; 9-5p; 195-5p; 135a-5p; 26a-5p; 21-5p; 153-3p; 145-5p; 125b-5p
30	Vinblastine	chemotherapy drugs- vinca alkaloids	0.0193092	5	451a; 148a-3p; 323a-3p; 27b-3p; 27a-3p
31	XMD8-92	potent and selective inhibitor of BMK1/ERK5/MAPK7 and DCAMKL1 (DCLK1), kinases implicated in tumorigenesis	0.0193092	4	143-3p; hsa-let-7a-5p; 144-3p; 145-5p
32	Oxaliplatin L-OHP	chemotherapy drug (alkylating agent)	0.0267627	9	92a-3p; 93-5p; 25-3p; 30d-5p; 191-5p; 128-3p; 320d; hsa-let-7b-5p; 92b-3p
33	Vitamin D3	Vitamin D3	0.0359766	13	19a-3p; 146a-5p; 766-3p; 106b-5p; 15b-5p; 191-5p; 203a-3p; hsa-let-7a-5p; 543; 26a-5p; 532-3p; 339-5p; 99b-5p
34	Calcitriol	active form of vitamin D	0.0486089	5	181c-5p; 15a-5p; 411-5p; 20b-5p; 126-3p
35	5-methoxy-N.N-dimethyltryptamine 5-MeO-DMT	naturally-occurring psychoactive indolealkylamine drugs	0.0487295	3	18a-5p; 124-3p; 27b-3p
36	Camptothecin	chemotherapy drugs- alkaloid from the stem wood of the Chinese tree (*Camptotheca acuminata*)	0.0487295	3	17-5p; 155-5p; 125b-5p
37	Cisplatin	chemotherapy drug (alkylating agent)	0.0487295	18	182-5p; 142-5p; 425-5p; 16-5p; 192-5p; 30d-5p; 381-3p; 487b-3p; 98-5p; 106a-5p; 27b-3p; 204-5p; 411-3p; 21-5p; 340-5p; 454-3p; 23b-3p; 125b-5p
38	Gefitinib	chemotherapy drug- epidermal growth factor receptor (EGFR) tyrosine kinase inhibitor	0.0487295	3	655-3p; 487b-3p; 134-5p
39	Hydroxychloroquine	antimalarials drug	0.0487295	3	155-5p; hsa-let-7a-5p; 21-5p
40	Nicotine	stimulant drug -agonist at nicotinic acetylcholine receptors	0.0487295	3	146a-5p; 16-5p; 21-5p
41	Prednisone	corticosteroids	0.0487295	3	146a-5p; hsa-let-7a-5p; 21-5p
42	Vincristine	chemotherapy drug (alkylating agent)	0.0487295	3	93-5p; 210-3p; 494-3p

*p-value after the Benjamini-Hochberg adjustment
