# Supplementary material for: A Set of 17 microRNAs Common for Brain and Cerebrospinal Fluid Differentiates Primary Central Nervous System Lymphoma from Non-Malignant Brain Tumors
Source: Biomolecules. 2021 Sep 21;11(9):1395. doi: 10.3390/biom11091395 (PMC8466839; doi:10.3390/biom11091395)
Supplement: Supplementary file 1 [file biomolecules-11-01395-s001.zip › Table S8.pdf]

Supplementary Table S8

Sources of CNS DLBCL-specific CSF microRNAs.

GO Cellular component (miRPathDB)				
**No**	**subcategory**	**q-value***	**number of miRs observed**	**hsa-miRs**
1	vesicle	2.702E-07	10	19a-3p; 16-5p; 148a-3p; 15a-5p; 19b-3p; 423-5p; 15b-5p; 17-3p; 92a-3p; 9-5p
2	cytoplasm	8.538E-07	13	19a-3p; 148a-3p; 15a-5p; 19b-3p; 221-3p; 423-5p; 21-5p; 15b-5p; 17-3p; 92a-3p; 9-5p; 20a-5p; 26a-5p
3	nuclear body	3.948E-06	10	19a-3p; 16-5p; 15a-5p; 19b-3p; 221-3p; 21-5p; 15b-5p; 17-3p; 92a-3p; 26a-5p
4	protein-containing complex	5.438E-06	13	16-5p; 148a-3p; 15a-5p; 19b-3p; 221-3p; 423-5p; 21-5p; 15b-5p; 17-3p; 92a-3p; 25-3p; 20a-5p; 26a-5p
5	RISC-loading complex	1.637E-05	7	19a-3p; 16-5p; 148a-3p; 15a-5p; 19b-3p; 21-5p; 15b-5p
6	cytoplasmic vesicle	1.637E-05	8	19a-3p; 16-5p; 148a-3p; 15a-5p; 19b-3p; 423-5p; 15b-5p; 9-5p
7	cytosol	1.637E-05	12	19a-3p; 16-5p; 148a-3p; 15a-5p; 19b-3p; 221-3p; 21-5p; 15b-5p; 17-3p; 92a-3p; 20a-5p; 26a-5p
8	endomembrane system	1.637E-05	7	19a-3p; 16-5p; 148a-3p; 19b-3p; 15b-5p; 92a-3p; 9-5p
9	intracellular vesicle	1.637E-05	8	19a-3p; 16-5p; 148a-3p; 15a-5p; 19b-3p; 423-5p; 15b-5p; 9-5p
10	organelle membrane	1.637E-05	7	19a-3p; 16-5p; 148a-3p; 15a-5p; 19b-3p; 15b-5p; 92a-3p
11	phagophore assembly site	1.637E-05	5	19a-3p; 16-5p; 19b-3p; 423-5p; 20a-5p
12	microtubule cytoskeleton	3.566E-05	7	16-5p; 148a-3p; 15a-5p; 19b-3p; 15b-5p; 92a-3p; 26a-5p
13	transferase complex	9.786E-05	8	19a-3p; 16-5p; 15a-5p; 19b-3p; 21-5p; 15b-5p; 92a-3p; 26a-5p
14	micro-ribonucleoprotein complex	0.0001407	7	19a-3p; 148a-3p; 15a-5p; 19b-3p; 21-5p; 15b-5p; 20a-5p
15	transcriptional repressor complex	0.0001407	7	19a-3p; 16-5p; 15a-5p; 19b-3p; 15b-5p; 92a-3p; 9-5p
16	catalytic complex	0.0001419	9	19a-3p; 16-5p; 15a-5p; 19b-3p; 221-3p; 21-5p; 15b-5p; 92a-3p; 26a-5p

Organs (miRWalk)				
**No**	**subcategory**	**q-value**	**number of miRs observed**	**hsa-miRs**
1	lymphocytes	0.0007133	9	16-5p; 148a-3p; 15a-5p; 19b-3p; 221-3p; 15b-5p; 92a-3p; 20a-5p; 26a-5p
2	blood cells	0.0044112	4	148a-3p; 19b-3p; 92a-3p; 20a-5p
3	bodily secretions	0.0044112	8	19a-3p; 16-5p; 15a-5p; 19b-3p; 21-5p; 9-5p; 20a-5p; 26a-5p
4	bone and bones	0.0044112	10	19a-3p; 16-5p; 148a-3p; 19b-3p; 21-5p; 92a-3p; 25-3p; 9-5p; 20a-5p; 26a-5p
5	granulocytes	0.014233	4	148a-3p; 19b-3p; 92a-3p; 20a-5p
6	PC12 cells	0.014233	3	16-5p; 15a-5p; 221-3p
7	T-lymphocytes	0.014233	7	148a-3p; 19b-3p; 221-3p; 21-5p; 92a-3p; 20a-5p; 26a-5p
8	immune system	0.0211429	4	148a-3p; 19b-3p; 92a-3p; 20a-5p
9	plasma cells	0.0211429	4	19a-3p; 19b-3p; 92a-3p; 25-3p
10	umbilical cord	0.0211429	4	148a-3p; 19b-3p; 92a-3p; 20a-5p
11	blood	0.0230447	8	16-5p; 148a-3p; 15a-5p; 19b-3p; 221-3p; 92a-3p; 9-5p; 20a-5p
12	blood vessels	0.0230447	3	16-5p; 221-3p; 92a-3p

13	endothelial cells	0.0230447	7	19a-3p; 16-5p; 221-3p; 21-5p; 210-3p; 92a-3p; 20a-5p
14	erythroid precursor cells	0.0230447	2	16-5p; 15a-5p
15	joints	0.0230447	2	19a-3p; 19b-3p
16	killer cells natural	0.0230447	3	16-5p; 15a-5p; 15b-5p
17	muscle smooth	0.0230447	4	221-3p; 21-5p; 25-3p; 26a-5p
18	myocytes smooth muscle	0.0230447	4	221-3p; 21-5p; 25-3p; 26a-5p
19	stomach	0.0230447	3	21-5p; 20a-5p; 26a-5p
20	X Chromosome	0.0230447	3	19b-3p; 221-3p; 92a-3p
21	brain	0.0261338	9	15a-5p; 19b-3p; 221-3p; 21-5p; 9-3p; 15b-5p; 9-5p; 20a-5p; 26a-5p
22	hematopoietic stem cells	0.0391702	4	148a-3p; 19b-3p; 92a-3p; 20a-5p
23	hand	0.0491233	4	16-5p; 15a-5p; 21-5p; 20a-5p

*p-value after the Benjamini-Hochberg adjustment
