# Supplementary material for: A Set of 17 microRNAs Common for Brain and Cerebrospinal Fluid Differentiates Primary Central Nervous System Lymphoma from Non-Malignant Brain Tumors
Source: Biomolecules. 2021 Sep 21;11(9):1395. doi: 10.3390/biom11091395 (PMC8466839; doi:10.3390/biom11091395)
Supplement: Supplementary file 1 [file biomolecules-11-01395-s001.zip › Table S9.pdf]

Supplementary Table S9

Molecular functions of CNS DLBCL-specific CSF microRNAs.

GO Annotations

No	subcategory	q-value*	number of miRs observed	hsa-miRs
1	miRNA mediated inhibition of translation	0.0028406	11	16-5p; 148a-3p; 15a-5p; 19b-3p; 221-3p; 21-5p; 15b-5p; 210-3p; 92a-3p; 9-5p; 20a-5p
2	negative regulation of cell population proliferation	0.0028406	7	16-5p; 15a-5p; 221-3p; 21-5p; 15b-5p; 9-5p; 26a-5p
3	positive regulation of tyrosine phosphorylation of STAT protein	0.0062128	3	19a-3p; 19b-3p; 221-3p
4	extracellular exosome	0.0100212	6	19a-3p; 16-5p; 15a-5p; 21-5p; 9-3p; 9-5p
5	negative regulation of cell migration involved in sprouting angiogenesis	0.0100328	6	16-5p; 15a-5p; 19b-3p; 221-3p; 20a-5p; 26a-5p
6	negative regulation of vascular smooth muscle cell differentiation	0.0100328	3	221-3p; 15b-5p; 26a-5p
7	positive regulation of apoptotic process	0.0100328	5	16-5p; 15a-5p; 221-3p; 15b-5p; 92a-3p
8	positive regulation of vascular associated smooth muscle cell migration	0.0100328	4	221-3p; 21-5p; 20a-5p; 26a-5p
9	negative regulation of G1S transition of mitotic cell cycle	0.0159948	5	16-5p; 15a-5p; 21-5p; 15b-5p; 26a-5p
10	negative regulation of I-kappaB kinaseNF-kappaB signaling	0.0159948	3	16-5p; 15a-5p; 15b-5p
11	negative regulation of inflammatory response	0.0159948	6	19a-3p; 16-5p; 15a-5p; 221-3p; 92a-3p; 20a-5p

GO Annotations indirect

No	subcategory	q-value	number of miRs observed	hsa-miRs
1	neuron apoptotic process	1.66625E-10	17	19a-3p; 16-5p; 148a-3p; 15a-5p; 19b-3p; 221-3p; 423-5p; 21-5p; 15b-5p; 17-3p; 210-3p; 92a-3p; 106b-3p; 25-3p; 9-5p; 20a-5p; 26a-5p
2	anaphase-promoting complex binding	3.1119E-09	15	19a-3p; 16-5p; 15a-5p; 19b-3p; 221-3p; 423-5p; 21-5p; 15b-5p; 17-3p; 210-3p; 92a-3p; 106b-3p; 25-3p; 20a-5p; 26a-5p
3	maintenance of DNA methylation	4.28806E-09	10	19a-3p; 16-5p; 148a-3p; 19b-3p; 221-3p; 21-5p; 92a-3p; 9-5p; 20a-5p; 26a-5p
4	cellular response to epinephrine stimulus	9.01727E-09	10	19a-3p; 16-5p; 15a-5p; 19b-3p; 423-5p; 21-5p; 15b-5p; 92a-3p; 25-3p; 26a-5p
5	inositol phosphate dephosphorylation	9.01727E-09	14	19a-3p; 16-5p; 15a-5p; 19b-3p; 221-3p; 21-5p; 15b-5p; 17-3p; 210-3p; 92a-3p; 106b-3p; 25-3p; 20a-5p; 26a-5p
6	negative regulation of wound healing. spreading of epidermal cells	9.01727E-09	13	19a-3p; 16-5p; 148a-3p; 19b-3p; 221-3p; 21-5p; 17-3p; 210-3p; 92a-3p; 106b-3p; 25-3p; 20a-5p; 26a-5p
7	phosphatidylinositol-3-phosphatase activity	9.01727E-09	14	19a-3p; 16-5p; 15a-5p; 19b-3p; 221-3p; 423-5p; 21-5p; 15b-5p; 17-3p; 92a-3p; 106b-3p; 25-3p; 20a-5p; 26a-5p
8	positive regulation of TRAIL-activated apoptotic signaling pathway	1.60974E-08	12	19a-3p; 16-5p; 19b-3p; 221-3p; 423-5p; 21-5p; 17-3p; 92a-3p; 106b-3p; 25-3p; 20a-5p; 26a-5p
9	positive regulation of ubiquitin-dependent protein catabolic process	2.25879E-08	14	19a-3p; 16-5p; 15a-5p; 19b-3p; 221-3p; 423-5p; 21-5p; 15b-5p; 17-3p; 92a-3p; 106b-3p; 25-3p; 20a-5p; 26a-5p
10	regulation of transmembrane transporter activity	2.25879E-08	13	19a-3p; 16-5p; 148a-3p; 15a-5p; 19b-3p; 221-3p; 21-5p; 15b-5p; 210-3p; 92a-3p; 9-5p; 20a-5p; 26a-5p

11	negative regulation of stress-activated MAPK cascade	2.33102E-08	13	19a-3p; 16-5p; 148a-3p; 15a-5p; 423-5p; 21-5p; 9-3p; 15b-5p; 92a-3p; 106b-3p; 25-3p; 9-5p; 20a-5p
12	atrial septum morphogenesis	2.37975E-08	13	19a-3p; 16-5p; 148a-3p; 15a-5p; 19b-3p; 21-5p; 9-3p; 15b-5p; 92a-3p; 25-3p; 9-5p; 20a-5p; 26a-5p
13	central nervous system myelin maintenance	2.37975E-08	13	19a-3p; 16-5p; 15a-5p; 19b-3p; 221-3p; 21-5p; 15b-5p; 17-3p; 92a-3p; 106b-3p; 25-3p; 20a-5p; 26a-5p
14	positive regulation of ossification	3.81655E-08	13	19a-3p; 16-5p; 148a-3p; 15a-5p; 19b-3p; 221-3p; 21-5p; 15b-5p; 92a-3p; 106b-3p; 25-3p; 20a-5p; 26a-5p
15	nuclear-transcribed mRNA catabolic process. no-go decay	4.39091E-08	11	19a-3p; 16-5p; 15a-5p; 19b-3p; 221-3p; 423-5p; 15b-5p; 92a-3p; 25-3p; 9-5p; 20a-5p
16	prepulse inhibition	4.49503E-08	11	19a-3p; 19b-3p; 221-3p; 21-5p; 17-3p; 92a-3p; 106b-3p; 25-3p; 9-5p; 20a-5p; 26a-5p
17	rRNA adenine-N6.N6--dimethyltransferase activity	4.76441E-08	6	19a-3p; 16-5p; 15a-5p; 19b-3p; 15b-5p; 210-3p
18	synapse assembly	4.76441E-08	17	19a-3p; 16-5p; 148a-3p; 15a-5p; 19b-3p; 221-3p; 423-5p; 21-5p; 9-3p; 17-3p; 210-3p; 92a-3p; 106b-3p; 25-3p; 9-5p; 20a-5p; 26a-5p
19	central nervous system neuron axonogenesis	5.01429E-08	15	19a-3p; 16-5p; 148a-3p; 19b-3p; 221-3p; 21-5p; 9-3p; 15b-5p; 17-3p; 210-3p; 92a-3p; 106b-3p; 25-3p; 20a-5p; 26a-5p
20	motor behavior	5.01429E-08	9	19a-3p; 16-5p; 15a-5p; 19b-3p; 15b-5p; 17-3p; 25-3p; 9-5p; 26a-5p
21	targeting of mRNA for destruction involved in RNA interference	5.01429E-08	10	19a-3p; 16-5p; 148a-3p; 15a-5p; 19b-3p; 221-3p; 21-5p; 15b-5p; 25-3p; 9-5p
22	RNA stem-loop binding	5.44842E-08	13	19a-3p; 16-5p; 15a-5p; 19b-3p; 221-3p; 423-5p; 21-5p; 15b-5p; 92a-3p; 106b-3p; 25-3p; 9-5p; 26a-5p
23	negative regulation of cytoplasmic translation	5.44842E-08	13	16-5p; 15a-5p; 221-3p; 423-5p; 21-5p; 15b-5p; 17-3p; 210-3p; 92a-3p; 25-3p; 9-5p; 20a-5p; 26a-5p
24	nerve development	5.44842E-08	11	19a-3p; 16-5p; 148a-3p; 15a-5p; 19b-3p; 221-3p; 21-5p; 15b-5p; 210-3p; 9-5p; 26a-5p
25	neuron-neuron synaptic transmission	5.44842E-08	12	19a-3p; 16-5p; 19b-3p; 221-3p; 21-5p; 17-3p; 92a-3p; 106b-3p; 25-3p; 9-5p; 20a-5p; 26a-5p
26	brain morphogenesis	7.55843E-08	14	19a-3p; 16-5p; 15a-5p; 19b-3p; 221-3p; 423-5p; 21-5p; 15b-5p; 17-3p; 92a-3p; 106b-3p; 25-3p; 20a-5p; 26a-5p
27	negative regulation of T-helper 17 cell differentiation	7.59629E-08	10	16-5p; 15a-5p; 423-5p; 21-5p; 15b-5p; 210-3p; 92a-3p; 25-3p; 9-5p; 20a-5p
28	ARC complex	8.3917E-08	9	19a-3p; 16-5p; 148a-3p; 15a-5p; 19b-3p; 221-3p; 21-5p; 15b-5p; 9-5p
29	conversion of ds siRNA to ss siRNA	8.3917E-08	9	19a-3p; 16-5p; 148a-3p; 15a-5p; 19b-3p; 221-3p; 21-5p; 15b-5p; 9-5p
30	conversion of ds siRNA to ss siRNA involved in RNA interference	8.3917E-08	9	19a-3p; 16-5p; 148a-3p; 15a-5p; 19b-3p; 221-3p; 21-5p; 15b-5p; 9-5p
31	gamete generation	8.3917E-08	12	19a-3p; 16-5p; 15a-5p; 19b-3p; 221-3p; 21-5p; 15b-5p; 17-3p; 92a-3p; 25-3p; 9-5p; 26a-5p
32	negative regulation of insulin secretion	8.3917E-08	16	19a-3p; 16-5p; 148a-3p; 15a-5p; 19b-3p; 221-3p; 423-5p; 21-5p; 9-3p; 15b-5p; 210-3p; 92a-3p; 106b-3p; 25-3p; 9-5p; 20a-5p
33	positive regulation of Schwann cell differentiation	8.3917E-08	9	19a-3p; 16-5p; 148a-3p; 15a-5p; 19b-3p; 221-3p; 21-5p; 15b-5p; 9-5p

*p-value after the Benjamini-Hochberg adjustment
